# Pyrimidin‐6‐yl Trifluoroborate Salts as Versatile Templates for Heterocycle Synthesis

**DOI:** 10.1002/anie.202101297

**Published:** 2021-03-17

**Authors:** David L. Cousins, Prisca Fricero, Kenji P. M. Kopf, Elliot J. McColl, Werngard Czechtizky, Yee Hwee Lim, Joseph P. A. Harrity

**Affiliations:** ^1^ Department of Chemistry University of Sheffield Sheffield S3 7HF UK; ^2^ Integrated Drug discovery R&D Sanofi Aventis (Deutschland) GmbH Industriepark Höchst 65926 Frankfurt am Main Germany; ^3^ Present address: Department of Medicinal Chemistry Research and Early Development Respiratory & Immunology BioPharmaceuticals R&D AstraZeneca Pepparedsleden 1 43183 Mölndal Sweden; ^4^ Functional Molecules & Polymers Institute of Chemical and Engineering Sciences A*STAR, Biopolis Singapore Singapore

**Keywords:** chemoselective, cross-coupling, pyrimidine, trifluoroborate

## Abstract

We report a novel and general method to access a highly under‐studied privileged scaffold—pyrimidines bearing a trifluoroborate at C4, and highlight the broad utility of these intermediates in a rich array of downstream functionalization reactions. This chemistry is underpinned by the unique features of the trifluoroborate group; its robustness provides an opportunity to carry out chemoselective reactions at other positions on the pyrimidine while providing a pathway for elaboration at the C−B bond when suitably activated.

## Graphical abstract

Stable and enabling: Pyrimidine trifluoroborates are an under‐developed scaffold that have been rarely reported in the literature. These compounds participate in a rich array of downstream functionalization reactions that are underpinned by the unique features of the trifluoroborate group; its robustness provides an opportunity to carry out chemoselective reactions at other positions on the pyrimidine while providing a pathway for elaboration at the C−B bond when activated.

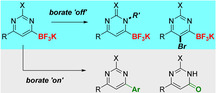

Pyrimidines are amongst the most widely represented class of heterocycles in biological systems. They are present in nucleic acids and countless other biologically relevant compounds, including numerous pharmaceutical and agrochemical products (Figure 1).^[1]^ Indeed, in 2020, 8 out of 35 small‐molecule drugs that gained FDA approval contained a pyrimidine heterocycle, demonstrating the continued demand for ways to access this privileged substructure. In addition to their ubiquity in the biological sciences, pyrimidine derivatives are also common building blocks in many functional materials such as supramolecular assemblies, non‐linear optics and organic electronics.^[2]^

**Figure 1:**
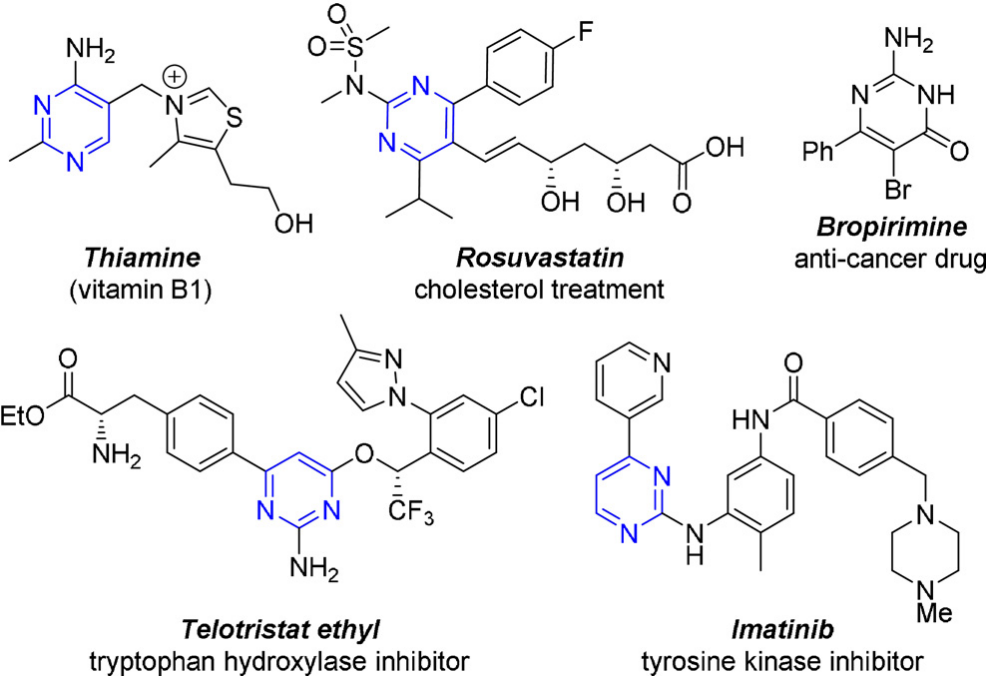
Prominent pyrimidine containing heterocycles.

The ring C–H metalation of pyrimidines offers a direct means to further functionalize these systems,^[3]^ and this has been successfully implemented at C2,^[4]^ C4,^[5]^ and C5.^[6]^ However, ring metalation requires strong bases and often leads to low yields of products due to the propensity of these intermediates to undergo dimerization. In recent years, there have been numerous reports of radical/homolytic functionalization of pyrimidines as a mild and functional group tolerant alternative to deprotonative metalation.^[7]^ Several notable examples of this strategy have allowed the predictable, selective and mild functionalization of pyrimidines under photoredox catalysis and Minisci conditions.^[8]^

While this approach is powerful for pyrimidine alkylation and (electron‐rich) arylation at C4 due to the innate electrophilicity of this ring position, metalation would prove complementary to this strategy and so more robust C4 metalated pyrimidine derivatives would make a welcome addition to the arsenal of methods available for pyrimidine functionalization. In this regard, boronic acid derivatives offer an intriguing alternative to main group organometallics as they are generally stable, easy to handle and enjoy a rich array of potential functionalization methods. We anticipated that pyrimidine boronic acid derivatives would offer an established route to a range of derivatization strategies. To our surprise however, they are remarkably under‐represented, with the 4‐borylated congener being the subject of only very limited studies to‐date. For catalytic transformations, this fragment is typically supplanted by a (pseudo)halide in Suzuki–Miyaura cross‐couplings or an organotin reagent in Stille cross couplings.^[9]^ Among the possible reasons for this may include the tendency for boronic acids and esters adjacent to ring heteroatoms to undergo undesired side‐reactions, such as protodeboronation and oxidation.^[10]^ Notably, attempts at catalytic borylation at the pyrimidine C6 have been unsuccessful.^[11]^ This adds to the incentive to develop alternative approaches to access this important class of intermediate.

Organotrifluoroborate salts offer a powerful means for the isolation and utility of otherwise unstable/sensitive boronic acids and esters.^[12]^ As shown in Figure 2, we recognized the opportunity to exploit ynone trifluoroborates **1** ^[13]^ to access novel pyrimidines that would provide a platform for a rich array of downstream manipulations. This scaffold would demonstrate the unique chemoselectivity of the trifluoroborate group that is available for derivatization when activated under appropriate conditions, but is otherwise unaffected during a range of other transformations.

**Figure 2:**
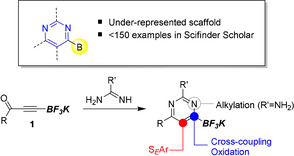
Design strategy for the synthesis and functionalization of pyrimidin‐6‐yl boronates.

We began our studies by investigating the condensation reaction of amidines and ynone trifluoroborate salts and our results are summarized in Figure 3. A brief optimization study revealed that refluxing toluene promoted the smooth condensation of benzaminidine free base with a range of ynone trifluoroborate salts to give the corresponding pyrimidine borates in good yield. Encouragingly, with regard to the ynone substituent, electron‐rich, electron‐poor, and halogenated aromatics were tolerated. In addition, pyrazole‐substituted ynone **1 i** underwent smooth condensation with benzamidine to afford **2 i** which was isolated in excellent yield. The procedure could also be applied to alkyl‐substituted ynone trifluoroborates, allowing the isolation of pyrimidines **2 f**–**h** in good yield. With regard to the amidine component, numerous benzamidines were tolerated in this process to give pyrimidines **2 j**–**l** with various substituted aromatics in the 2‐position. Heteroaromatic fragments, along with a cyclopropyl substituent could also be incorporated, providing the corresponding products in good yield.

**Figure 3:**
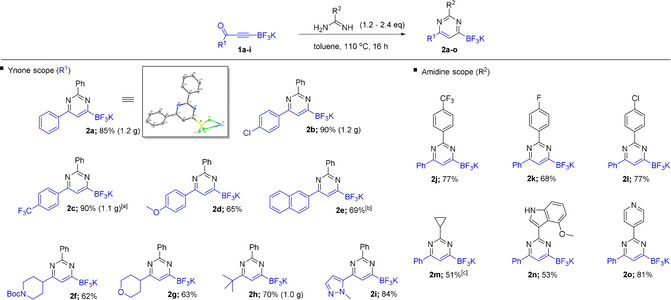
Scope of pyrimidin‐6‐yl trifluoroborate salts. [a] 3.8 equiv of benzamidine used. [b] Reaction mixture was heated for 48 hours. [c] 6.0 equiv of cyclopropylamidine used.

A significant proportion of pyrimidine‐containing drugs contain a 2‐amino substituent (cf. Figure 1), therefore, a priority for further scope investigation was to determine if this important pharmacophore could be incorporated in the structure of the product salts, via reaction of the appropriate guanidine with our ynone salts. Starting with *N*‐carbamimidoyl pivalamide as a non‐hygroscopic and crystalline equivalent of guanidine freebase, we were surprised to isolate free aminopyrimidine **4 a** with no trace of the corresponding amide (Figure 4).^[14]^ This process appeared to be quite general and delivered a small family of 2‐aminopyrimidine borate salts in good to excellent yields. We next turned our attention to the condensation of *N*‐substituted guanidines and again were pleased to find that we could generate a range of *N*‐substituted analogs. In this case the products were isolated with high regioselectivities after recrystallization, and the regiochemistry was assigned on the basis of ^1^H NMR spectroscopy, and by X‐ray crystallography in the case of **6**.^[15]^

**Figure 4:**
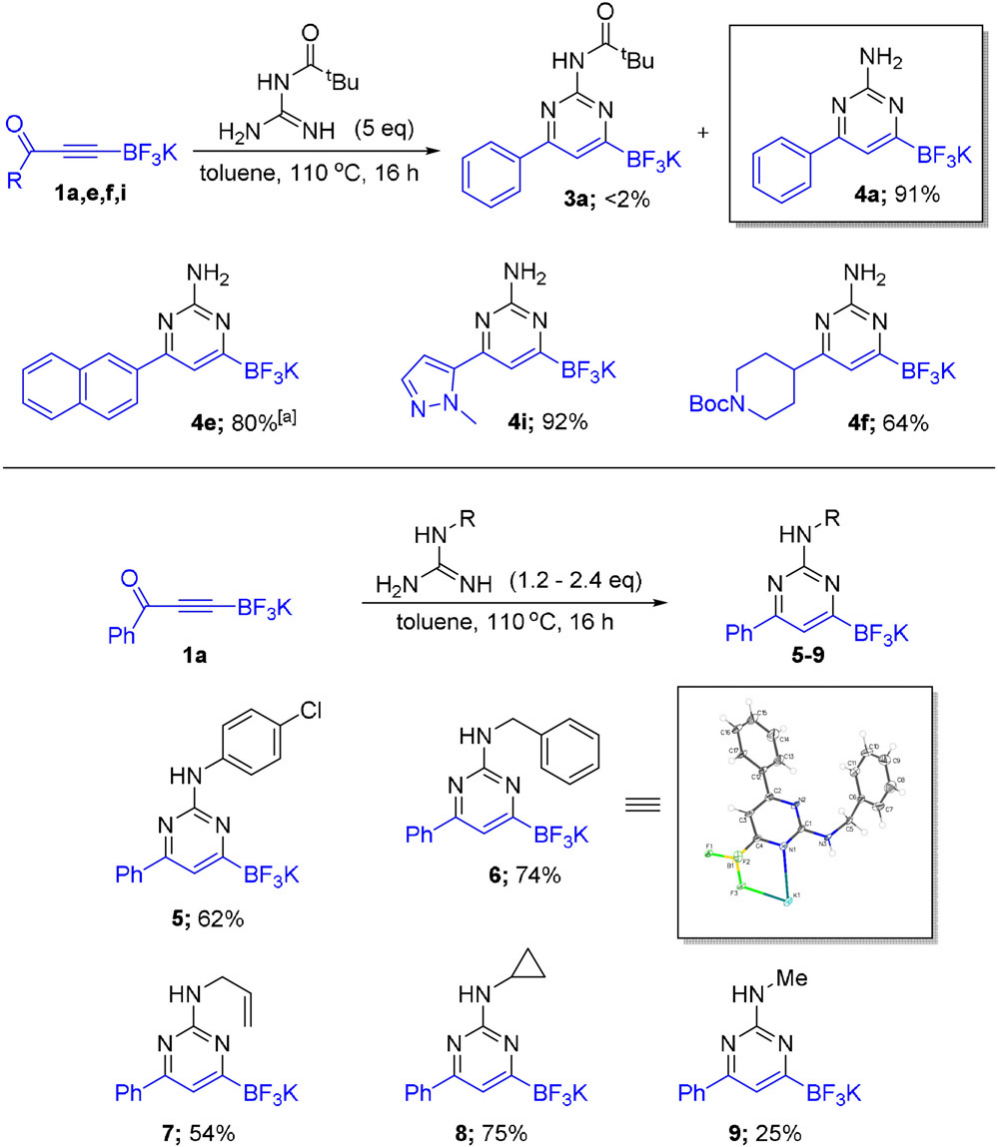
Synthesis of 2‐aminopyrimidine borate salts. [a] Reaction mixture was heated for 48 hours.

Finally, and with a range of pyrimidine trifluoroborate salts in hand, we decided to investigate the potential of these compounds for further organic synthesis, with an emphasis on highlighting chemoselective manipulations of the functionality present in these molecules. As shown in Figure 5, treatment of **4 a** with MeI and allyl bromide resulted in the formation of **10 a,b**. These compounds are isomers of **7** and **9** that were generated by direct condensation (cf Figure 4) which serves to highlight the diversity of substitution available in the generation of these heterocyclic products. These compounds were isolated as single regioisomers and the regioselectivity was assigned by X‐ray crystallography and HMBC spectroscopy (see Supporting Information).^[16]^ Turning to the trifluoroborate, we were pleased to find that this group proved to be amenable to promoting cross‐coupling with aryl iodides, furnishing biaryl products **11 a,b** in good yield. Finally, subjecting **4 a** to bromination results in clean electrophilic aromatic substitution at the pyrimidine C5 position, leaving the trifluoroborate group intact. Subsequent oxidation of the C−B bond provided the experimental anti‐cancer drug, bropirimine in high yield over two steps.

**Figure 5:**
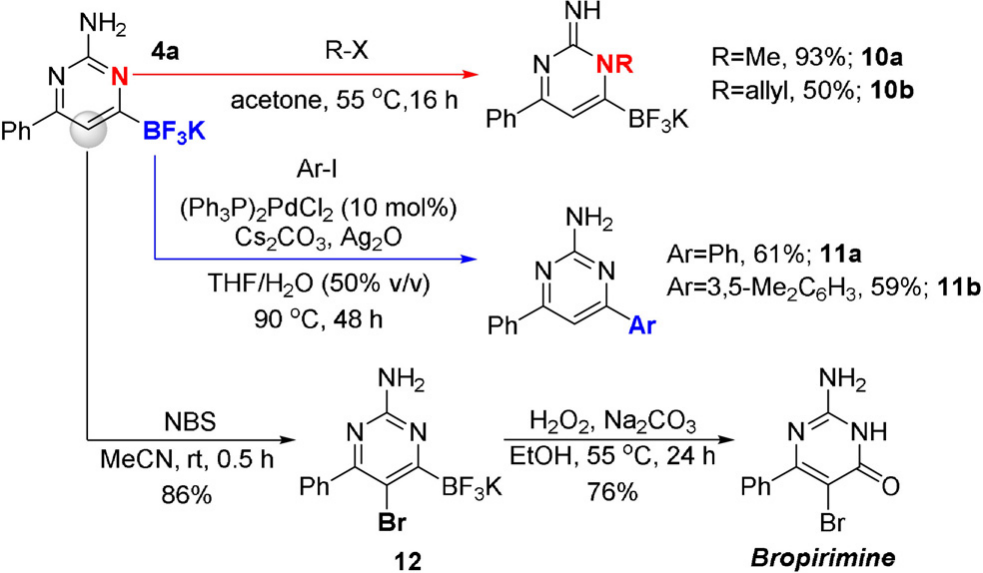
Elaboration of pyrimidin‐6‐yl trifluoroborate salts.

In conclusion, we report that C4‐borylated pyrimidine derivatives can be readily accessed by condensation reactions of ynone trifluoroborates, providing the first general method to access this class of heterocycles. This work serves to highlight the robustness of the potassium trifluoroborate handle; it is stable towards strongly nucleophilic amidines and guanidines, as well as alkylating agents and even bromine. However, it also provides a pathway for elaboration of the pyrimidines via Suzuki–Miyaura cross‐coupling and oxidation. The trifluoroborate salts presented herein are crystalline, bench‐stable materials that have been stored without precaution for more than 1 year, without noticeable degradation. It is hoped that these compounds will find application as robust intermediates in the synthesis of useful pyrimidines.

## Conflict of interest

The authors declare no conflict of interest.

## Supporting information

As a service to our authors and readers, this journal provides supporting information supplied by the authors. Such materials are peer reviewed and may be re‐organized for online delivery, but are not copy‐edited or typeset. Technical support issues arising from supporting information (other than missing files) should be addressed to the authors.

Supplementary
