# Supplementary material for: Pyrimidin‐6‐yl Trifluoroborate Salts as Versatile Templates for Heterocycle Synthesis
Source: Angew Chem Int Ed Engl. 2021 Mar 17;60(17):9412–5. doi: 10.1002/anie.202101297 (PMC8252621; doi:10.1002/anie.202101297)
Supplement: Supplementary file 1 — Supplementary [file ANIE-60-9412-s001.pdf]

Angewandte Chemie
Eine Zeitschrift der Gesellschaft Deutscher Chemiker

Supporting Information

Pyrimidin-6-yl Trifluoroborate Salts as Versatile Templates for Heterocycle Synthesis

*David L. Cousins, Prisca Fricero, Kenji P. M. Kopf, Elliot J. McColl, Werngard Czechtizky, Yee Hwee Lim, and Joseph P. A. Harrity**

anie_202101297_sm_miscellaneous_information.pdf

Contents

General considerations **S2**

Synthetic procedures and compound characterisation **S3**

HMBC Assignment of 10b **S15**

NMR spectra of Pyrimidines **S16**

References **S72**

X-ray data for 2a **S73**

X-ray data for 6 **S79**

X-ray data for 10a **S86**

General considerations

All reactions were conducted in flame-dried glassware under ambient conditions unless otherwise stated. THF and toluene were dried before use over an alumina column. All commercially available solvents and reagents were used as supplied or purified using standard laboratory techniques according to methods described by Perrin and Armarego.[1]

Thin layer chromatography was performed on aluminium-backed plates pre-coated with silica (Merck silica Kieselgel 60 F254), which were developed using standard visualizing agents: ultraviolet light or potassium permanganate. Flash chromatography was performed on silica gel (60 Å, mesh 40-63 µm). Melting points were obtained using a Stuart apparatus and are uncorrected.

^{1}H spectra were recorded on a Bruker AVIII HD-400 (400 MHz), Bruker AVI-400 (400 MHz), Bruker AMX-400 (400 MHz) or DPX-400 (400 MHz). Proton magnetic resonance chemical shifts are reported from tetramethylsilane with the residual protic solvent resonance as the internal standard (DMSO: δ = 2.50 ppm). Data are reported as follows: chemical shift (ppm), multiplicity (s = singlet, d = doublet, t = triplet, q = quartet, quint = quintet, br = broad, m = multiplet), normalised peak integral (arbitrary units) then coupling constant (Hz). ^{13}C NMR spectra were recorded on a Bruker AVIII HD-400 (101 MHz), Bruker AVI-400 (101 MHz), Bruker AMX-400 (101 MHz) or DPX-400 (101 MHz). Carbon magnetic resonance chemical shifts are reported from tetramethylsilane with the solvent as the internal reference (DMSO: δ = 2.50 ppm). ^{19}F NMR spectra were recorded on a Bruker AMX-400 (376 MHz) or Bruker AVIII HD-400 (376 MHz) and the chemical shifts are uncorrected. ^{11}B NMR spectra were recorded on a Bruker AVIII HD-400 (128 MHz), and the chemical shifts are uncorrected.

Infrared spectra were recorded on a Perkin-Elmer Paragon 100 FTIR spectrometer. Spectra were obtained from neat compounds through the use of a standard ATR attachment, and the most structurally relevant bands are quoted in cm^{-1}. Bands are characterized as broad (br), strong (s), medium (m) or weak (w). High-resolution mass spectra (HRMS) were recorded on a MicroMass LCT operating in Electrospray mode (TOF ES).

Synthetic procedures and compound characterisation

Compounds **1a-1i** were prepared according to published procedures.[2]

Note that the ^{13}C NMR signal for the carbon atom attached to boron is not observed due to quadrupolar relaxation broadening.[3]

General procedure A: Preparation of pyrimidin-6-yl trifluoroborates

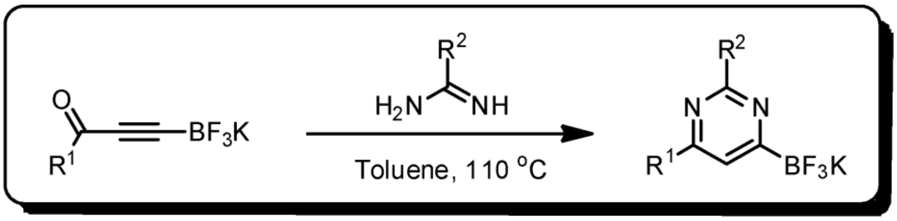

To a stirring suspension of ynone trifluoroborate salt **1** (1.0 eq) in toluene (0.07 M) was added amidine (1.2-6.0 eq) and the mixture heated at reflux for 18-48 hours. Upon completion (determined by ^{19}F NMR spectroscopy), the reaction mixture was cooled and concentrated *in* vacuo. The solid residue was re-dissolved in the minimum amount of acetone affording a saturated solution. The product was precipitated by adding diethyl ether and isolated by filtration or decantation followed by further washing with diethyl ether. The resulting solid was dried thoroughly *in vacuo*, to provide the desired pyrimidin-6-yl trifluoroborate **2**.

Potassium (2,6-diphenylpyrimidin-6-yl)trifluoroborate **2a**

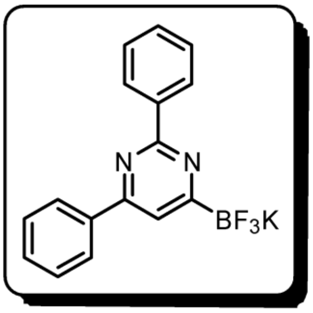

Following general procedure A using **1a** (1.00 g, 4.23 mmol) and benzamidine (1.23 g, 10.20 mmol). The reaction was complete within 16 hours, giving **2a** as a colourless solid (1.22 g, 85%). **M.p.** = 294 °C (dec); **^{1}H NMR (400 MHz, DMSO-d$_6$)** δ_H ppm 7.48 – 7.59 (m, 6H), 7.78 (s, 1H), 8.24 (dd, 2H, J = 8.0, 1.5 Hz), 8.54 (dd, 2H, J = 8.0, 1.5 Hz); **^{13}C NMR (101 MHz, DMSO-d^6)** δ_C ppm 116.7, 126.7, 127.8, 128.3, 128.9, 129.8, 130.1, 137.9, 139.1, 159.5, 161.8; **^{19}F NMR (376 MHz, DMSO-d^6)** δ_F ppm -142.6; **^{11}B NMR (128 MHz, DMSO-d^6)** δ_B ppm 1.5; **FTIR (neat)** ν_{max} / cm^{-1} 3000 (w), 1572 (s), 1514 (s); **HRMS (ESI-TOF)** *m/z* [M-K]$^-$ calculated for $C_{16}H_{11}{}^{11}BF_3N_2$ 299.0973, found 299.0985.

Potassium (2-phenyl-4-(4-chlorophenyl)pyrimidin-6-yl)trifluoroborate **2b**

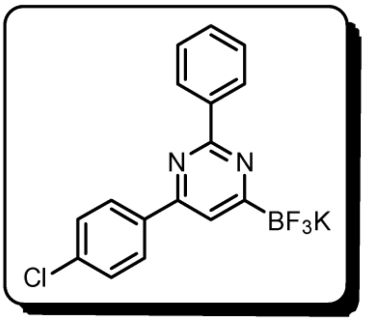

Following general procedure A using **1b** (1.00 g, 3.70 mmol) and benzamidine (1.07 g, 8.87 mmol). The reaction was complete within 24 hours, giving **2b** as a colourless solid (1.24 g, 90%). **M.p.** = >300 °C; **^{1}H NMR (400 MHz, DMSO-d$_6$)** δ_H ppm 7.45 – 7.57 (m, 3H), 7.60 (d, 2H, J = 8.5 Hz), 7.78 (s, 1H), 8.29 (d, 2H, J = 8.5 Hz), 8.53 (dd, 2H, J = 8.0, 1.5 Hz); **^{13}C NMR (101 MHz, DMSO-d^6)** δ_C ppm 116.6, 127.8, 128.3, 128.5, 128.9, 129.9, 134.9, 136.7, 138.9, 158.3, 161.8; **^{19}F NMR (376 MHz, DMSO-d^6)** δ_F ppm -142.6; **^{11}B NMR (128 MHz, DMSO-d^6)** δ_B ppm 1.4; **FTIR (neat)** ν_{max} / cm^{-1} 3096

(w), 1513 (m), 972 (s); **HRMS (ESI-TOF)** *m/z* [M-K]$^-$ calculated for $C_{16}H_{10}{}^{11}B^{35}ClF_3N_2$ 333.0583, found 333.0594.

Potassium (2-phenyl-4-(4-trifluoromethylphenyl)pyrimidin-6-yl)trifluoroborate **2c**

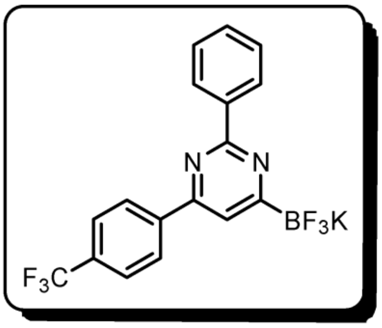

Following general procedure A using **1c** (900 mg, 2.96 mmol) and benzamidine (1.35 g, 11.24 mmol). The reaction was complete within 16 hours, giving **2c** as a colourless solid (1.08 g, 90%). **M.p.** = 297 °C (dec); **^{1}H NMR (400 MHz, DMSO-d^6)** δ_H ppm 7.49 – 7.57 (m, 3H), 7.88 (s, 1H), 7.91 (d, 2H, J = 8.0 Hz), 8.48 (d, 2H, J = 8.0 Hz), 8.56 (dd, 2H, J = 8.0, 1.5 Hz); **^{13}C NMR (101 MHz, DMSO-d^6)** δ_C ppm 117.3, 124.3 (q, J = 272.0 Hz), 125.8 (q, J = 3.5 Hz), 127.6, 127.8, 128.4, 130.0, 130.1 (q, J = 32.0 Hz), 138.8, 141.8, 158.1, 162.0; **^{19}F NMR (376 MHz, DMSO-d^6)** δ_F ppm -142.7, -61.2; **^{11}B NMR (128 MHz, DMSO-d^6)** δ_B ppm 1.7; **FTIR (neat)** ν_{max} / cm^{-1} 3393 (w), 1512 (m), 1328 (m), 978 (s); **HRMS (ESI-TOF)** *m/z* [M-K]$^-$ calculated for $C_{17}H_{10}{}^{11}BF_6N_2$ 367.0847, found 367.0855.

Potassium (2-phenyl-4-(4-methoxyphenyl)pyrimidin-6-yl)trifluoroborate **2d**

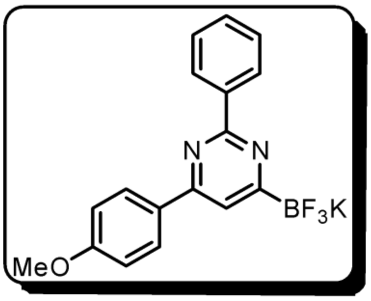

Following general procedure A using **1d** (115 mg, 0.49 mmol) and benzamidine (69 mg, 0.57 mmol). The reaction was complete within 16 hours, giving **2d** as a colourless solid (117 mg, 65%). **M.p.** = 257 °C (dec); **^{1}H NMR (400 MHz, DMSO-d^6)** δ_H ppm 3.84 (s, 3H), 7.10 (d, 2H, J = 9.0 Hz), 7.47 – 7.54 (m, 3H), 7.72 (s, 1H), 8.22 (d, 2H, J = 9.0 Hz), 8.53 (dd, 2H, J = 8.0, 1.5 Hz); **^{13}C NMR (101 MHz, DMSO-d^6)** δ_C ppm 55.3, 114.2, 115.9, 127.7, 128.1, 128.2, 129.6, 130.1, 139.2, 159.2, 161.0, 161.6; **^{19}F NMR (376 MHz, DMSO-d^6)** δ_F ppm -142.7; **^{11}B NMR (128 MHz, DMSO-d^6)** δ_B ppm 1.6; **FTIR (neat)** ν_{max} / cm^{-1} 3085 (w), 3047 (w), 2960 (w), 2936 (w), 2836 (w), 1610 (m), 1588 (m), 1572 (m), 1509 (s), 1370 (s), 1354 (s), 966 (s); **HRMS (ESI-TOF)** *m/z* [M-K]$^-$ calculated for $C_{17}H_{13}{}^{11}BF_3N_2O$ 329.1073, found 329.1089.

Potassium (2-phenyl-4-(naphthalen-2-yl)pyrimidin-6-yl)trifluoroborate **2e**

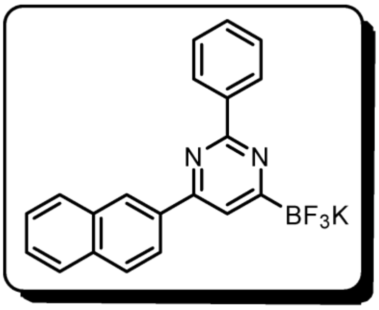

Following general procedure A using **1e** (100 mg, 0.35 mmol) and benzamidine (84 mg, 0.70 mmol). The reaction was complete within 40 hours, giving **2e** as a colourless solid (94 mg, 69%). **M.p.** = 255-256 °C (dec); **^{1}H NMR (400 MHz, DMSO-d^6)** δ_H ppm 7.48 – 7.64 (m, 5H), 7.95 – 8.03 (m, 2H), 8.09 (d, 1H, J = 8.5 Hz), 8.14 – 8.21 (m, 1H), 8.44 (d, 1H, J = 8.5 Hz), 8.61 (d, 2H, J = 7.0 Hz), 8.85 (s, 1H); **^{13}C NMR (101 MHz, DMSO-d^6)** δ_C ppm 117.5, 124.5, 126.9 (x2C), 127.5, 128.0, 128.3, 128.8, 128.9, 129.4, 130.2, 133.5, 134.3, 135.8, 139.5, 159.9, 162.3; **^{19}F NMR (376 MHz, DMSO-d^6)** δ_F ppm -142.6; **^{11}B NMR (128 MHz, DMSO-d^6)** δ_B ppm 1.8; **FTIR (neat)** ν_{max} / cm^{-1} 3064 (w), 1572 (w), 1519 (m), 1365 (m), 969 (s); **HRMS (ESI-TOF)** *m/z* [M-K]$^-$ calculated for $C_{20}H_{13}{}^{11}BF_3N_2$ 349.1129, found 349.1140.

Potassium (2-phenyl-4-((1-Boc)piperidin-4-yl)pyrimidin-6-yl)trifluoroborate **2f**

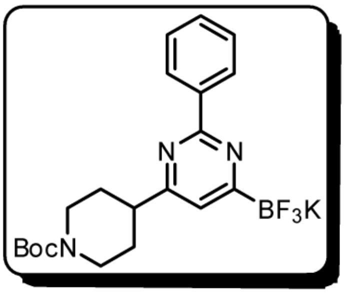

Following general procedure A using **1f** (149 mg, 0.43 mmol) and benzamidine (64 mg, 0.53 mmol). The reaction was complete within 16 hours, giving **2f** as a light yellow solid (119 mg, 62%). **M.p.** = 127-128 °C; **^{1}H NMR (400 MHz, DMSO-d^6)** δ_H ppm 1.43 (s, 9H), 1.61 (dq, 2H, J = 12.5, 4.0 Hz), 1.87 (dd, 2H, J = 12.5, 1.5 Hz), 2.73 – 2.98 (m, 3H), 4.07 (d, 2H, J = 11.0 Hz), 7.16 (s, 1H), 7.39 – 7.51 (m, 3H), 8.41 (dd, 2H, J = 8.0, 1.5 Hz); **^{13}C NMR (101 MHz, DMSO-d^6)** δ_C ppm 28.1, 30.7, 42.8, 64.9, 78.6, 119.0, 127.6, 128.2, 129.5, 139.2, 154.0, 161.2, 168.5; **^{19}F NMR (376 MHz, DMSO-d^6)** δ_F ppm -142.6; **^{11}B NMR (128 MHz, DMSO-d^6)** δ_B ppm 1.4; **FTIR (neat)** ν_{max} / cm^{-1} 2974 (w), 2931 (w), 2857 (w), 1671 (s), 1518 (s), 1427 (s), 1232 (s), 1166 (s); **HRMS (ESI-TOF)** *m/z* [M-K]$^-$ calculated for $C_{20}H_{24}{}^{11}BF_3N_3O_2$ 406.1914, found 406.1926.

Potassium (2-phenyl-4-(pyran-4-yl)pyrimidin-6-yl)trifluoroborate **2g**

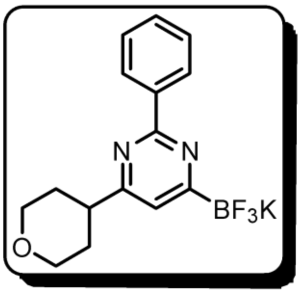

Following general procedure A using **1g** (111 mg, 0.45 mmol) and benzamidine (69 mg, 0.57 mmol). The reaction was complete within 16 hours, giving **2g** as a light brown solid (98 mg, 63%). **M.p.** 209 °C (dec); **^{1}H NMR (400 MHz, DMSO-d^6)** δ_H ppm 1.73 – 1.85 (m, 4H), 2.82 – 2.91 (m, 1H), 3.47 (dt, 2H, J = 11.0, 3.5 Hz), 3.92 – 4.01 (m, 2H), 7.18 (s, 1H), 7.44 – 7.49 (m, 3H), 8.42 (dd, 2H, J = 8.0, 1.5 Hz); **^{13}C NMR (101 MHz, DMSO-d^6)** δ_C ppm 31.4, 41.9, 67.0, 118.9, 127.7, 128.2, 129.5, 139.2, 161.3, 168.5; **^{19}F NMR (376 MHz, DMSO-d^6)** δ_F ppm -142.5; **^{11}B NMR (128 MHz, DMSO-d^6)** δ_B ppm 1.5; **FTIR (neat)** ν_{max} / cm^{-1} 2953 (w), 2858 (w), 1568 (m), 1516 (s), 1492 (w), 1463 (w), 1088 (m); **HRMS (ESI-TOF)** *m/z* [M-K]$^-$ calculated for $C_{15}H_{15}{}^{11}BF_3N_2O$ 307.1230, found 307.1246.

Potassium (2-phenyl-4-tert-butylpyrimidin-6-yl)trifluoroborate **2h**

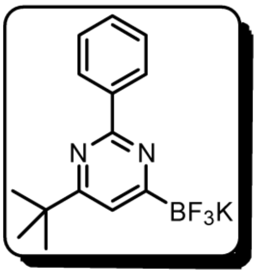

Following general procedure A using **1h** (1.00 g, 4.63 mmol) and benzamidine (1.30 g, 10.82 mmol). The reaction was complete within 16 hours, giving **2h** as a colourless solid (1.03 g, 70%). **M.p.** >300 °C; **^{1}H NMR (400 MHz, DMSO-d^6)** δ_H ppm 1.34 (s, 9H), 7.32 (s, 1H), 7.43 – 7.51 (m, 3H), 8.44 (dd, 2H, J = 8.0, 1.5 Hz); **^{13}C NMR (101 MHz, DMSO-d^6)** δ_C ppm 29.4, 64.9, 116.4, 127.6, 128.2, 129.4, 139.5, 160.8, 172.9; **^{19}F NMR (376 MHz, DMSO-d^6)** δ_F ppm -142.4; **^{11}B NMR (128 MHz, DMSO-d^6)** δ_B ppm 1.4; **FTIR (neat)** ν_{max} / cm^{-1} 2962 (w), 2922 (w), 2903 (w), 2870 (w), 1569 (w), 1516 (m); **HRMS (ESI-TOF)** *m/z* [M-K]$^-$ calculated for $C_{14}H_{15}{}^{11}BF_3N_2$ 279.1286, found 279.1288.

Potassium (2-phenyl-4-(1-methylpyrazol-5-yl)pyrimidin-6-yl)trifluoroborate **2i**

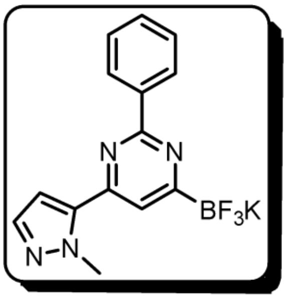

Following general procedure A using **1i** (108 mg, 0.45 mmol) and benzamidine (123 mg, 1.02 mmol). The reaction was complete within 16 hours, giving **2i** as a colourless solid (129 mg, 84%). **M.p.** 263-264 °C (dec); **^{1}H NMR (400 MHz, DMSO-d^6)** δ_H ppm 4.33 (s, 3H), 6.95 (d, 1H, J = 2.0 Hz), 7.46 – 7.56 (m, 4H), 7.59 (s, 1H), 8.47 (dd, 2H, J = 8.0, 1.5 Hz); **^{13}C NMR (101 MHz, DMSO-d^6)** δ_C ppm 40.1, 107.5, 118.8, 127.7, 128.5, 130.0, 137.9, 138.8, 139.7, 153.1, 161.5; **^{19}F NMR (376 MHz, DMSO-d^6)** δ_F ppm -142.8; **^{11}B NMR (128 MHz, DMSO-d^6)** δ_B ppm 1.6; **FTIR (neat)** ν_{max} / cm^{-1} 1570 (m), 1511 (m), 1454 (m), 1424 (w); **HRMS (ESI-TOF)** *m/z* [M-K]$^-$ calculated for $C_{14}H_{11}{}^{11}BF_3N_4$ 303.1034, found 303.1047.

Potassium 2-(4-trifluoromethylphenyl)-4-phenylpyrimidin-6-yl)trifluoroborate **2j**

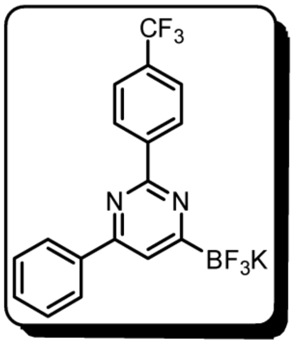

Following general procedure A using **1a** (50 mg, 0.21 mmol) and (4-trifluoromethyl)benzamidine (80 mg, 0.51 mmol). The reaction was complete within 16 hours, giving **2j** as a colourless solid (66 mg, 77%). **M.p.** 243-244 °C (dec); **^{1}H NMR (400 MHz, CD_3CN)** δ_H ppm 7.49 – 7.58 (m, 3H), 7.80 (d, 2H, J = 8.0 Hz), 7.96 (s, 1H), 8.26 – 8.33 (m, 2H), 8.70 (d, 2H, J = 8.0 Hz); **^{13}C NMR (101 MHz, CD_3CN)** δ_C ppm 118.6, 125.6 (q, J = 271.0 Hz), 126.3 (q, J = 3.5 Hz), 128.1, 129.5, 129.8, 131.3, 131.6 (q, J = 32.0 Hz), 139.0, 144.3, 162.0, 162.6; **^{19}F NMR (376 MHz, CD_3CN)** δ_F ppm -63.0, -145.6; **^{11}B NMR (128 MHz, CD_3CN)** δ_B ppm 1.6; **FTIR (neat)** ν_{max} / cm^{-1} 3145 (w), 1573 (m), 1513 (s), 1318 (s), 980 (s), 971 (s); **HRMS (ESI-TOF)** *m/z* [M-K]$^-$ calculated for $C_{17}H_{10}{}^{11}BF_6N_2$ 367.0841, found 367.0855.

Potassium 2-(4-fluorophenyl)-4-phenylpyrimidin-6-yl)trifluoroborate **2k**

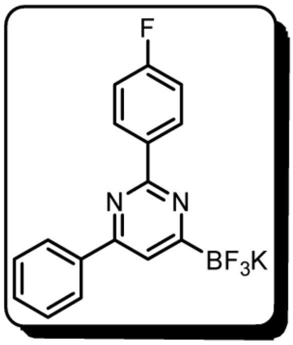

Following general procedure A using **1a** (83 mg, 0.35 mmol) and (4-fluoro)benzamidine (97 mg, 0.70 mmol). The reaction was complete within 24 hours giving **2k** as a colourless solid (88 mg, 68%). **M.p.** 292-293 °C (dec); **^{1}H NMR (400 MHz, DMSO-d^6)** δ_H ppm 7.28 – 7.40 (m, 2H), 7.49 – 7.61 (m, 3H), 7.79 (s, 1H), 8.24 (dd, 2H, J = 8.0, 1.0 Hz), 8.59 (dd, 2H, J = 8.5, 6.0 Hz); **^{13}C NMR (101 MHz, DMSO-d^6)** δ_C ppm 115.2 (d, J = 21.5 Hz), 116.7, 126.7, 128.9, 130.0 (d, J = 8.5 Hz), 130.2, 135.6 (d, J = 2.5 Hz), 137.8, 159.6, 161.0, 163.5 (d, J = 246.5 Hz); **^{19}F NMR (376 MHz, DMSO-d^6)** δ_F ppm -112.3, -142.7; **^{11}B NMR (128 MHz, DMSO-d^6)** δ_B ppm 1.6; **FTIR (neat)** ν_{max} / cm^{-1} 3596 (w), 3146 (w), 1668 (w), 1603 (w), 1574 (w), 1507 (w), 1349 (w), 997 (s); **HRMS (ESI-TOF)** *m/z* [M-K]$^-$ calculated for $C_{16}H_{10}{}^{11}BF_4N_2$ 317.0873, found 317.0887.

Potassium 2-(4-chlorophenyl)-4-phenylpyrimidin-6-yl)trifluoroborate **2l**

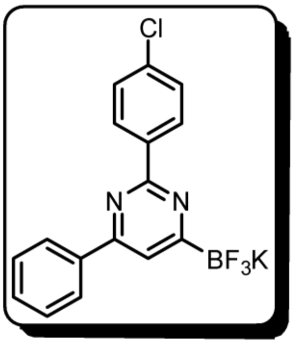

Following general procedure A using **1a** (83 mg, 0.35 mmol) and (4-chloro)benzamidine (110 mg, 0.70 mmol). The reaction was complete within 24 hours, giving **2l** as a colourless solid (100 mg, 77%). **M.p.** 280-281 °C (dec); **^{1}H NMR (400 MHz, DMSO-d^{6})** δ_H ppm 7.46 – 7.68 (m, 5H), 7.80 (s, 1H), 8.24 (dd, 2H, J = 7.5, 1.0 Hz), 8.56 (d, 2H, J = 8.5 Hz); **^{13}C NMR (101 MHz, DMSO-d^{6})** δ_C ppm 116.9, 126.7, 128.4, 128.9, 129.5, 130.2, 134.7, 137.7, 137.9, 159.6, 160.8; **^{19}F NMR (376 MHz, DMSO-d^{6})** δ_F ppm -142.7; **^{11}B NMR (128 MHz, DMSO-d^{6})** δ_B ppm 1.7; **FTIR (neat)** ν_{max} / cm^{-1} 3070 (w), 1591 (w), 1514 (s), 1350 (s), 974 (s); **HRMS (ESI-TOF)** *m/z* [M-K]$^-$ calculated for $C_{16}H_{10}{}^{11}B^{35}ClF_3N_2$ 333.0578, found 333.0594.

Potassium 2-cyclopropyl-4-phenylpyrimidin-6-yl)trifluoroborate **2m**

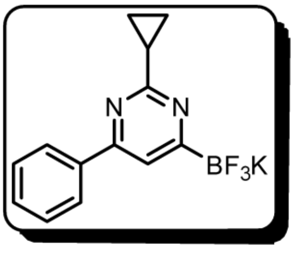

Following general procedure A using **1a** (250 mg, 1.06 mmol) and (4-cyclopropyl)benzamidine (535 mg, 6.36 mmol). The reaction was complete within 16 hours, giving **2m** as a tan solid (163 mg, 51%). **M.p.** 295 °C (dec); **^{1}H NMR (400 MHz, DMSO-d^{6})** δ_H ppm 0.93 – 0.99 (m, 2H), 1.02 – 1.07 (m, 2H), 2.22 (tt, 1H, J = 8.0, 5.0 Hz), 7.43 – 7.55 (m, 3H), 7.60 (s, 1H), 8.04 – 8.10 (m, 2H); **^{13}C NMR (101 MHz, DMSO-d^{6})** δ_C ppm 9.9, 18.5, 116.0, 127.0, 129.2, 130.3, 138.5, 159.6, 169.3; **^{19}F NMR (376 MHz, DMSO-d^{6})** δ_F ppm -142.6; **^{11}B NMR (128 MHz, DMSO-d^{6})** δ_B ppm 1.5; **FTIR (neat)** ν_{max} / cm^{-1} 3096 (w), 1575 (s), 1520 (s), 1009 (s), 981 (s); **HRMS (ESI-TOF)** *m/z* [M-K]$^-$ calculated for $C_{13}H_{11}{}^{11}BF_3N_2$ 263.0973, found 263.0981.

Potassium (2-(4-methoxy-1H-indol-3-yl)-4-phenylpyrimidin-6-yl)trifluoroborate **2n**

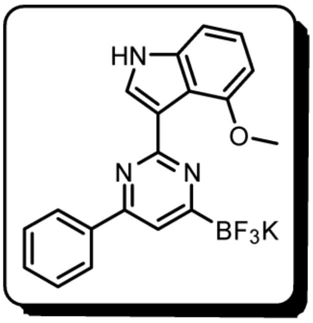

Following general procedure A using **1a** (83 mg, 0.35 mmol) and 4-methoxy-1*H*-indole-3-carboximidamide (80 mg, 0.42 mmol), added portion-wise. The reaction was complete within 16 hours, giving **2n** as a light brown solid (75 mg, 53%). **M.p** = 174-175 °C (dec); **^{1}H NMR (400 MHz, DMSO-d$_6$)** δ_H ppm 3.93 (s, 3H), 6.72 (d, 1H, J = 5.5 Hz), 7.09 – 7.18 (m, 2H), 7.51 – 7.60 (m, 3H), 7.73 – 7.82 (s, 1H), 8.18 (s, 1H), 8.30 – 8.38 (m, 2H); **^{13}C NMR (101 MHz, DMSO-d^{6})** δ_C ppm 55.7, 102.0, 103.4, 106.1, 114.3, 114.8, 123.3, 124.5, 127.5, 128.9, 131.0, 138.8, 139.1, 151.6, 153.1, 160.3; **^{19}F NMR (376 MHz, DMSO-d^{6})** δ_F ppm -143.3; **^{11}B NMR (128 MHz, DMSO-d^{6})** δ_B ppm 1.3; **FTIR (neat)** ν_{max} / cm^{-1} 3351 (w), 3069 (w), 1576 (s), 1525 (m), 1509 (s), 1485 (w), 1086 (s); **HRMS (ESI-TOF)** *m/z* [M-K]$^-$ calculated for $C_{19}H_{14}{}^{11}B^{19}F_3N_3O$ 368.1188, found 368.1196.

Potassium (2-(4-pyridyl)-4-phenylpyrimidin-6-yl)trifluoroborate **2o**

Following general procedure A using **1a** (100 mg, 0.42 mmol) and isonicotinamidine (103 mg, 0.85 mmol). The reaction was complete within 16 hours, giving **2o** as a colourless solid (117 mg, 81%). **M.p** = 272-273 °C (dec); **^{1}H NMR (400 MHz, DMSO-d$_6$)** δ_H ppm 7.51 – 7.61 (m, 3H), 7.90 (s, 1H), 8.27 (dd, 2H, J = 8.0, 1.5 Hz), 8.42 (dd, 2H, J = 4.5, 1.5 Hz), 8.76 (dd, 2H, J = 4.5, 1.5 Hz); **^{13}C NMR (101 MHz,**

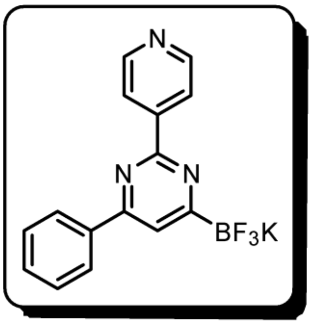

DMSO-d[6]) δ_C ppm 118.0, 121.8, 126.8, 129.0, 130.4, 137.4, 146.1, 150.2, 159.9, 160.1; **^{19}F NMR (376 MHz, DMSO-d[6])** δ_F ppm -142.7; **^{11}B NMR (128 MHz, DMSO-d[6])** δ_B ppm 1.7; **FTIR (neat)** ν_{max} / cm^{-1} 3042 (w), 2966 (w), 1555 (m), 1507 (s), 1004 (s), 963 (s); **HRMS (ESI-TOF)** *m/z* [M-K]$^-$ calculated for $C_{15}H_{10}{}^{11}B^{19}F_3N_3$ 300.0925, found 300.0932.

General procedure A: Preparation of 2-aminopyrimidin-6-yl trifluoroborates

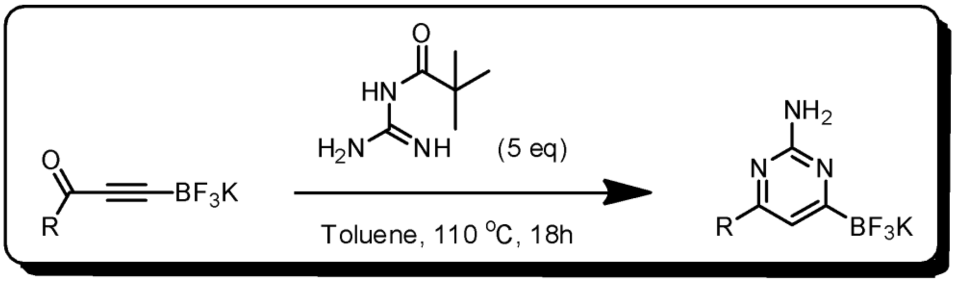

To a stirring suspension of ynone in toluene (0.07 M) at room temperature was added *N*-carbamimidoyl pivalate[4] (5 eq) in one portion. The mixture was heated at reflux until the reaction was complete, then cooled to room temperature and concentrated *in vacuo*. The residue was then suspended in acetone, and Et_2O was added slowly from a dropping funnel (typically ~10 times the volume of acetone). The mixture was then filtered and the resultant solid was washed with Et_2O then dried *in vacuo* to provide the corresponding potassium (aminopyrimidine)trifluoroborate salt **4**.

Potassium (2-amino-4-phenylpyrimidin-6-yl)trifluoroborate **4a**

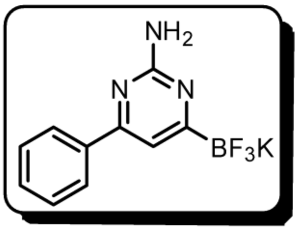

Following general procedure A using **1a** (500 mg, 2.12 mmol) and *N*-carbamimidoyl pivalate (1.52 g, 10.59 mmol). The reaction was complete within 18 hours, giving **4a** as a tan solid (534 mg, 91%). **M.p.** = 262-263 °C; **^{1}H NMR (400 MHz, DMSO-d6)** δ_H ppm 6.12 (s, 2H), 7.07 (s, 1H), 7.35 – 7.56 (m, 3H), 7.90 – 8.06 (m, 2H); **^{13}C NMR (101 MHz, DMSO-d[6])** δ_C ppm 108.7, 126.3, 128.5, 129.3, 138.8, 160.1, 163.2; **^{19}F NMR (376 MHz, DMSO-d[6])** δ_F ppm -142.7; **^{11}B NMR (128 MHz, DMSO-d[6])** δ_B ppm 1.4; **FTIR (neat)** ν_{max} / cm^{-1} 3474 (m), 3308 (m), 3193 (m), 3064 (m), 1619 (m), 1577 (s), 1527 (s), 969 (s); **HRMS (ESI-TOF)** *m/z* [M-K]$^-$ calculated for $C_{10}H_8{}^{11}BF_3N_3$ 238.0769, found 238.0772.

Potassium (2-amino-4-(naphthalen-2-yl)pyrimidin-6-yl)trifluoroborate **4b**

Following general procedure A using **1e** (100 mg, 0.35 mmol) and *N*-carbamimidoyl pivalate (250 mg, 1.75 mmol). The reaction was complete within 48 hours, giving **4b** as a tan solid (92 mg, 80%). **M.p.** = 235-236°C (dec); **^{1}H NMR (400 MHz, DMSO-d6)** δ_H ppm 6.16 (br, 2H), 7.27 (s, 1H), 7.55 (m, 2H), 7.87 – 8.12 (m, 3H), 8.16 (d, 1H, J = 7.5 Hz), 8.58 (s, 1H); **^{13}C NMR (101 MHz, DMSO-d[6])** δ_C ppm 109.0, 124.2,

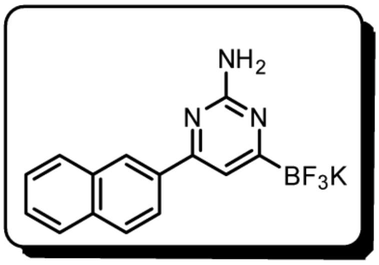

125.8, 126.4, 126.7, 127.5, 128.0, 128.7, 133.0, 133.6, 136.1, 160.1, 163.2; **^{19}F NMR (376 MHz, DMSO-d^6)** δ_F ppm -142.6; **^{11}B NMR (128 MHz, DMSO-d^6)** δ_B ppm 1.5; **FTIR (neat)** ν_{max} / cm^{-1} 3467 (w), 3305 (w), 3189 (w), 1526 (s), 954 (s); **HRMS (ESI-TOF)** *m/z* [M-K]$^-$ calculated for $C_{14}H_{10}{}^{11}BF_3N_3$ 288.0925, found 288.0930.

Potassium (2-amino-4-(1-methyl-1*H*-pyrazol-5-yl)pyrimidin-6-yl)trifluoroborate **4c**

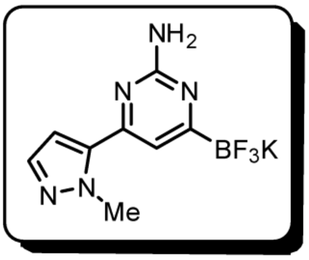

Following general procedure A using **1i** (400 mg, 1.67 mmol) and *N*-carbamimidoyl pivalate (1.20 g, 8.35 mmol). The reaction was complete within 18 hours, giving **4c** as a tan solid (427 mg, 92%). **M.p.** = 288-289 °C (dec); **^{1}H NMR (400 MHz, DMSO-d$_6$)** δ_H ppm 4.15 (s, 3H), 6.21 (br, 2H), 6.67 (d, 1H, J = 1.0 Hz), 6.85 (s, 1H), 7.43 (d, 1H, J = 1.0 Hz); **^{13}C NMR (101 MHz, DMSO-d^6)** δ_C ppm 39.4, 106.7, 110.7, 137.7, 140.5, 153.7, 162.5; **^{19}F NMR (376 MHz, DMSO-d^6)** δ_F ppm -142.9; **^{11}B NMR (128 MHz, DMSO-d^6)** δ_B ppm 1.4; **FTIR (neat)** ν_{max} / cm^{-1} 3469 (m), 3325 (m), 3212 (m), 3114 (w), 2951 (w), 1633 (m), 1526 (m), 974 (s); **HRMS (ESI-TOF)** *m/z* [M-K]$^-$ calculated for $C_8H_8{}^{11}BF_3N_5$ 242.0825, found 242.0832.

Potassium (2-amino-4-(1-Boc-piperidin-4-yl)pyrimidin-6-yl)trifluoroborate **4d**

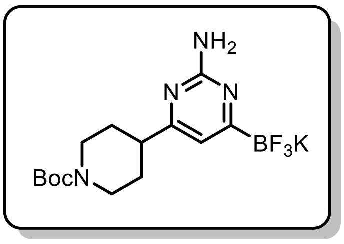

Following general procedure A using **1f** (160 mg, 0.47 mmol) and *N*-carbamimidoyl pivalate (334 mg, 2.33 mmol). The reaction was complete within 18 hours, giving **4d** as a tan solid (115 mg, 64%). **M.p.** = 207-208 °C (dec); **^{1}H NMR (400 MHz, DMSO-d$_6$)** δ_H ppm 1.26 – 1.52 (m, 11H), 1.71 (dd, 2H, J = 13.0, 2.0 Hz), 2.40 – 2.48 (m, 1H), 2.62 – 2.89 (m, 2H), 3.90 – 4.10 (m, 2H), 5.84 (br, 2H), 6.44 (s, 1H); **^{13}C NMR (101 MHz, DMSO-d^6)** δ_C ppm 28.1, 30.7, 42.9, 59.8, 78.5, 110.3, 153.9, 162.7, 169.1; **^{19}F NMR (376 MHz, DMSO-d^6)** δ_F ppm -142.7; **^{11}B NMR (128 MHz, DMSO-d^6)** δ_B ppm 1.5; **FTIR (neat)** ν_{max} / cm^{-1} 3461 (m), 3319 (m), 3174 (m), 2975 (m), 1628 (s), 1531 (s), 1428 (m), 963 (m); **HRMS (ESI-TOF)** *m/z* [M-K]$^-$ calculated for $C_{14}H_{21}{}^{11}BF_3N_4O_2$ 345.1715, found 345.1726.

Condensation of *N*-aryl/alkyl guanidines

Preparation of 2-((4-chorophenyl)amino)-4-phenylpyrimidin-6-yl trifluoroborate 5

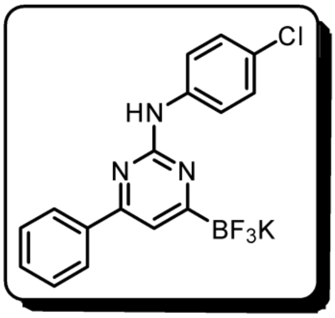

To a slurry of **1a** (174 mg, 0.74 mmol) in toluene (11 mL) was added (4-chlorophenyl)guanidine (150 mg, 0.88 mmol). The mixture was heated at reflux for 16 h then cooled to room temperature and concentrated *in vacuo*. The crude mixture was dissolved in acetone, and Et_2O was added. The resultant precipitate was collected. Cooling the mixture to 0 °C provided a second crop of product. The solids were combined, washed with Et_2O and dried *in vacuo* to give **5** as a yellow solid (178 mg, 62%). **M.p. =** 156-157 °C (dec); **^{1}H NMR (400 MHz, DMSO-d^6)** δ**H** ppm 7.31 (d, 2H, J = 9.0 Hz), 7.33 (s, 1H), 7.29 – 7.32 (m, 3H), 7.98 (d, 2H, J = 9.0 Hz), 8.08 (dd, 2H, J = 8.0, 1.0 Hz), 9.53 (br, 1H); **^{13}C NMR (101 MHz, DMSO-d^6)** δ_C ppm 110.9, 119.4, 123.4, 126.6, 128.2, 128.8, 129.9, 138.3, 140.9, 159.6, 160.0; **^{19}F NMR (376 MHz, DMSO-d^6)** δ_F ppm -142.7; **^{11}B NMR (128 MHz, DMSO-d^6)** δ_B ppm 1.5; **FTIR (neat)** ν_{max} / cm^{-1} 3414 (w), 3338 (br), 3338 (w), 1522 (s), 1490 (s), 1059 (m); **HRMS (ESI-TOF)** *m/z* [M-K]$^-$ calculated for $C_{16}H_{11}{}^{11}B^{35}ClF_3N_3$ 348.0692, found 348.0702.

General procedure C: Reaction of *N*-alkyl guanidine hydrochloride salts with 1a. Neutralisation of the salt using potassium carbonate prior to condensation.

To a stirring suspension of alkylguanidine hydrochloride (2.4-2.5 eq) in toluene was added potassium carbonate (2.4-2.5 eq). The mixture was heated at 80 °C for 2 h, then **1a** (1.0 eq) was added and the mixture was heated at reflux for 16 h. The mixture was cooled to room temperature and concentrated *in vacuo*, followed by dissolving in acetone and filtering to remove residual salts. The resultant acetone solution was concentrated to afford a saturated solution and Et_2O was added slowly. The aminopyrimidine trifluoroborate precipitate was then collected by filtration and dried thoroughly *in vacuo*.

Preparation of 2-(benzylamino)-4-phenylpyrimidin-6-yl trifluoroborate 6

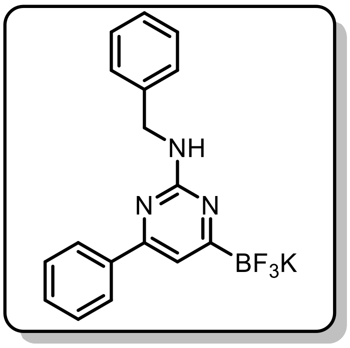

Following general procedure C using benzylguanidine hydrochloride (200 mg, 1.08 mmol), potassium carbonate (147 mg, 1.06 mmol), **1a** (100 mg, 0.42 mmol) and toluene (10 mL). The title compound was obtained as a colourless solid (114 mg, 74%). **M.p. =** 234-235 °C (dec); **^{1}H NMR (400 MHz, DMSO-d^6)** δ**H** ppm 4.61 (d, 2H, J = 6.0 Hz), 7.09 (s, 1H), 7.10 – 7.25 (m, 2H), 7.29 (t, 2H, J = 7.5 Hz), 7.37 – 7.47 (m, 5H), 7.99 (dd, 2H, J = 8.0, 1.5 Hz); **^{13}C NMR (101 MHz, DMSO-d^6)** δ_C ppm 44.2, 108.5, 126.3 (x2C), 127.3, 128.0, 128.5, 129.4, 138.7, 141.5, 159.6, 162.1; **^{19}F NMR (376 MHz, DMSO-d^6)** δ_F ppm -142.6; **^{11}B NMR (128 MHz, DMSO-d^6)** δ_B ppm 1.3; **FTIR (neat)** ν_{max} / cm^{-1} 3432 (m), 3063 (w), 3033 (w), 2945 (w), 1534 (s), 1026 (s), 953 (s); **HRMS (ESI-TOF)** *m/z* [M-K]$^-$ calculated for $C_{17}H_{14}{}^{11}BF_3N_3$ 328.1238, found 328.1251.

Preparation of 2-((*N*-allyl)amino)-4-phenylpyrimidin-6-yl trifluoroborate 7

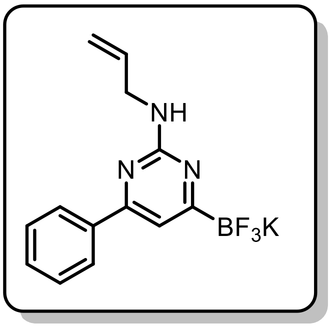

Following general procedure C using allylguanidine hydrochloride (115 mg, 0.85 mmol), potassium carbonate (117 mg, 0.85 mmol), **1a** (82 mg, 0.35 mmol) and toluene (6 mL). The title compound was obtained as a colourless solid (60 mg, 54%). **M.p.** = 204-205 °C (dec); **^{1}H NMR (400 MHz, DMSO-d^{6})** δ_H ppm 3.97 – 4.06 (m, 2H), 5.00 – 5.08 (m, 1H), 5.16 – 5.25 (m, 1H), 5.98 (ddt, 1H, J = 17.0, 10.5, 5.5 Hz), 6.69 (br, 1H), 7.08 (s, 1H), 7.40 – 7.51 (m, 3H), 8.02 (dd, 2H, J = 8.0, 1.5 Hz); **^{13}C NMR (101 MHz, DMSO-d^{6})** δ_C ppm 43.7, 109.0, 114.8, 116.4, 126.8, 129.0, 129.8, 139.1, 159.9, 162.3; **^{19}F NMR (377 MHz, DMSO-d^{6}):** δ_F ppm -142.6; **^{11}B NMR (128 MHz, DMSO-d^{6})** δ_B ppm 1.6; **FTIR (neat)** ν_{max} / cm^{-1} 3383, 1645, 1601, 1534, 1033; **HRMS (ESI-TOF)** [M-K]$^{-}$ calculated for $C_{13}H_{12}{}^{11}BF_3N_3$ 278.1082, found 278.1091.

Preparation of 2-((*N*-cyclopropyl)amino)-4-phenylpyrimidin-6-yl trifluoroborate 8

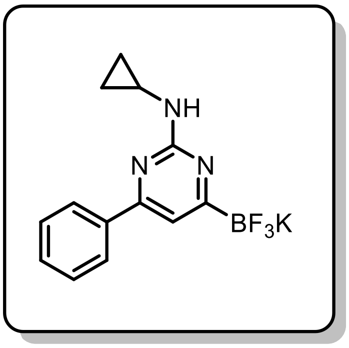

Following general procedure C using cyclopropylguanidine hydrochloride (145 mg, 1.07 mmol), potassium carbonate (147 mg, 1.06 mmol), **1a** (100 mg, 0.42 mmol) and toluene (10 mL). The title compound was obtained as a pale yellow solid (100 mg, 75%). **M.p.** = 168-170 °C (dec); **^{1}H NMR (400 MHz, DMSO-d^{6})** δ_H ppm 0.44 – 0.57 (m, 2H), 0.65 – 0.77 (m, 2H), 2.77 – 2.85 (m, 1H), 6.80 (s, 1H), 7.11 (s, 1H), 7.42 – 7.49 (m, 3H), 8.03 – 8.10 (m, 2H); **^{13}C NMR (101 MHz, DMSO-d^{6})** δ_C ppm 6.9, 24.4, 106.4, 127.2, 129.2, 131.0, 137.5, 159.3, 163.9; **^{19}F NMR (377 MHz, DMSO-d^{6})** δ_F ppm -142.6; **^{11}B NMR (128 MHz, DMSO-d^{6})** δ_B ppm 1.5; **FTIR (neat)** ν_{max} / cm^{-1} 3373, 1672, 1532, 1347, 1058; **HRMS**: **(ESI-TOF)** [M-K]$^{-}$ calculated for $C_{13}H_{12}{}^{11}BF_3N_3$ 278.1082, found 278.1094.

Preparation of 2-((*N*-methyl)amino)-4-phenylpyrimidin-6-yl trifluoroborate 9

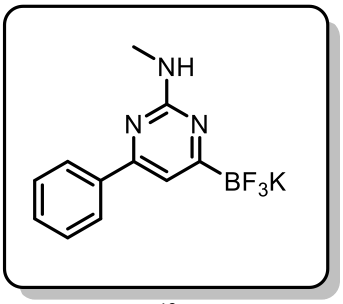

Following general procedure C using methylguanidine hydrochloride (116 mg, 1.06 mmol), potassium carbonate (147 mg, 1.06 mmol), **1a** (100 mg, 0.42 mmol) and toluene (10 mL). The title compound was obtained as a pale yellow solid (30 mg, 25%). **M.p.** = 154-155 °C (dec); **^{1}H NMR (400 MHz, DMSO-d^{6})** δ_H ppm 2.83 – 2.91 (m, 3H), 6.48 (br, 1H), 7.06 (s, 1H), 7.37 – 7.52 (m, 3H), 7.97 – 8.10 (m, 2H); **^{13}C NMR (101 MHz, DMSO-d^{6})** δ_C ppm 28.5, 108.7, 126.8, 129.0, 129.8, 139.3, 160.1, 163.2; **^{19}F NMR (377 MHz, DMSO-d^{6})** δ_F ppm -142.6; **^{11}B NMR (128 MHz, DMSO-d^{6})** δ_B ppm 1.8; **FTIR (neat)** ν_{max} / cm^{-1} 3357, 1662, 1542, 1308, 1080; **HRMS: (ESI-TOF)** [M-K]$^{-}$ calculated for $C_{11}H_{10}{}^{11}BF_3N_3$ 252.0925, found 252.0936.

General procedure D: Ring alkylation of aminopyrimidine 4a

To a stirring suspension of **4a** (1.0 eq) in acetone at room temperature was added alkyl halide (1.0 eq). The reaction mixture was heated at 50 °C for 16 h, then cooled to room temperature

and concentrated *in vacuo*, giving a saturated solution. Et_2O was added and the resultant precipitate was collected, washed with Et_2O and dried thoroughly *in vacuo* to afford the corresponding alkylated pyrimidine trifluoroborate salt.

Preparation of 1-methyl-2-(1*H*)-4-phenylpyrimidinimin-6-yl trifluoroborate 10a

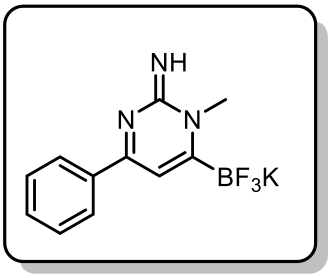

Following general procedure D, using **4a** (70 mg, 0.25 mmol), iodomethane (15 µL, 0.25 mmol) and acetone (8 mL) The title compound was obtained as a colourless solid (68 mg, 93%). **M.p** = 175-177 °C (dec); ^{1}**H NMR (400 MHz, DMSO-d^6)** δ_H ppm 3.77 (s, 3H), 6.90 (s, 1H), 7.45 (s, 1H), 7.55 – 7.67 (m, 3H), 8.11 – 8.21 (m, 2H); ^{13}**C NMR (101 MHz, DMSO-d^6)** δ_C ppm 38.6, 109.4, 127.8, 129.2, 132.5, 134.6, 156.5, 166.3; ^{19}**F NMR (377 MHz, DMSO-d^6)** δ_F ppm -141.5; ^{11}**B NMR (128 MHz, DMSO-d^6)** δ_B ppm 0.6; **FTIR (neat)** ν_{max} / cm^{-1} 3367, 1639, 1599, 1562, 1360; **HRMS: (ESI-TOF)** *m/z* [M-K]$^-$ calculated for $C_{11}H_{10}{}^{11}BF_3N_3$ 252.0925, found 252.0932.

Preparation of 1-allyl-2-(1*H*)-4-phenylpyrimidinimin-6-yl trifluoroborate 10b

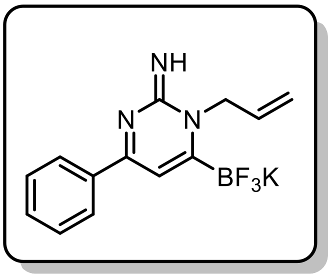

Following general procedure D, using **4a** (70 mg, 0.25 mmol), allyl bromide (22 µL, 0.25 mmol) and acetone (8 mL). The title compound was obtained as a colourless solid (40 mg, 50%). **M.p** = 173-174 °C (dec); ^{1}**H NMR (400 MHz, DMSO-d^6)** δ_H ppm 4.96 (d, 2H J = 3.0 Hz), 5.21 (d, 1H, J = 17.5 Hz), 5.26 (d, 1H, J = 11.0 Hz), 5.83 – 5.95 (m, 1H), 7.04 (s, 1H), 7.48 (s, 1H), 7.54 – 7.67 (m, 3H), 8.08 – 8.22 (m, 2H); ^{13}**C NMR (101 MHz, DMSO-d^6)** δ_C ppm 52.1, 109.9, 118.5, 128.0, 129.3, 130.4, 132.8, 134.5, 155.7, 166.8; ^{19}**F NMR (377 MHz, DMSO-d^6)** δ_F ppm -140.7; ^{11}**B NMR (128 MHz, DMSO-d^6)** δ_B ppm 0.6; **FTIR (neat)** ν_{max} / cm^{-1} 3367, 1636, 1597, 1556, 1362; **HRMS: (ESI-TOF)** *m/z* [M-K]$^-$ calculated for $C_{13}H_{12}{}^{11}BF_3N_3$. 278.1082, found 278.1096.

Bromination of 3a

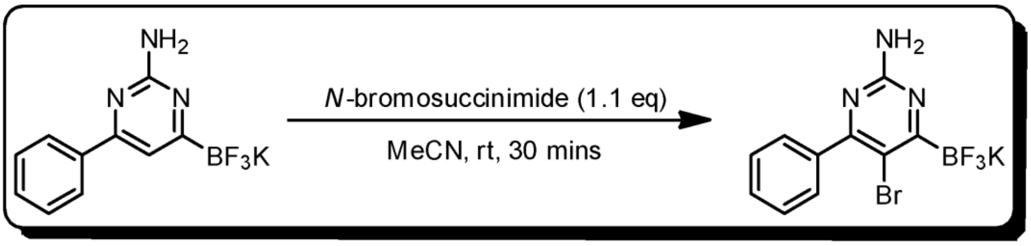

To a stirring slurry of **3a** (300 mg, 1.08 mmol) in MeCN (5 mL) in the absence of light was added *N*-bromosuccinimide (212 mg, 1.19 mmol) in one portion. The mixture was stirred rapidly in the dark at room temperature for 30 minutes, then Et_2O (20 mL) was added and the resulting suspension was allowed to stand undisturbed for 10 minutes. The mixture was filtered and the collected solid was

washed with Et_2O (3 x 10 mL) then dried *in vacuo* to give **5a** (329 mg, 86%) as a tan solid. **M.p.** 285-286 °C (dec); **^{1}H NMR (400 MHz, DMSO-d^6)** δ_H 6.28 (s, 2H), 7.29 – 7.58 (m, 5H); **^{13}C NMR (101 MHz, DMSO-d^6)** δ_C ppm 110.4, 127.5, 128.2, 128.8, 140.0, 161.1, 162.5; **^{19}F NMR (376 MHz, DMSO-d^6)** δ_F ppm -139.8; **^{11}B NMR (128 MHz, DMSO-d^6)** δ_B ppm 1.1; **FTIR (neat)** ν_{max} / cm^{-1} 3486 (m), 3330 (m), 3204 (w), 1626 (s), 1515 (s), 991 (s); **HRMS (ESI-TOF)** *m/z* [M-K]$^-$ calculated for $C_{10}H_7N_3{}^{11}B^{79}BrF_3$ 315.9874, found 315.9878.

Synthesis of Bropirimine *via* oxidation of 5a

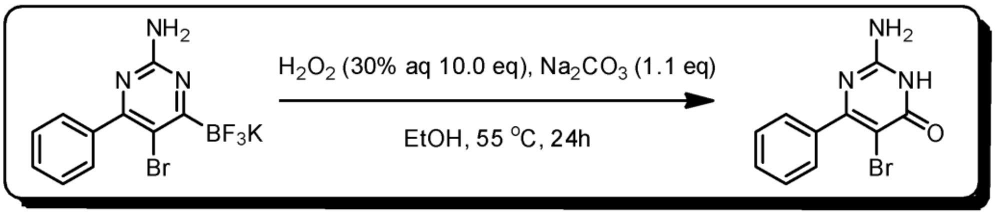

A solution of **5a** (220 mg, 0.62 mmol), H_2O_2 (30% aq, 631 µL, 6.18 mmol), Na_2CO_3 (72 mg, 0.68 mmol) in ethanol (7 mL) behind a precautionary blast shield was heated at 55 °C for 24 hours then cooled to room temperature and diluted with EtOAc (25 mL) then H_2O (10 mL). The phases were separated and the aqueous phase was extracted with EtOAc (5 x 20 mL). The combined organic phases were dried over anhydrous $MgSO_4$ then concentrated *in vacuo*. The crude product was purified by flash column chromatography on silica gel (eluting with EtOAc) to give bropirimine (125 mg, 76%) as a colourless solid. **M.p.** 269-270 °C (lit[5] = 275-277 °C); **^{1}H NMR (400 MHz, DMSO-d^6)** δ_H ppm 6.76 (s, 2H), 7.34 – 7.49 (m, 3H), 7.49 – 7.60 (m, 2H), 11.42 (s, 1H); **^{13}C NMR (101 MHz, DMSO-d^6)** δ_C ppm 96.0, 127.7, 128.5, 128.9, 139.1, 154.2, 159.2, 163.5.

General procedure E: Suzuki-Miyaura cross coupling of 4a

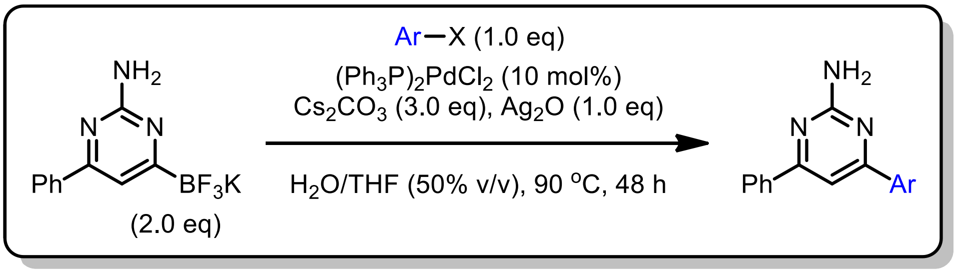

A 10 mL Biotage microwave vial was charged with **4a** (2.0 eq), $(Ph_3P)_2PdCl_2$ (10 mol%), Cs_2CO_3 (3.0 eq) and Ag_2O (1.0 eq). The tube was then capped and purged with argon before the aryl halide (1.0 eq) and then H_2O/THF (50% vv, diluting ArX to a concentration of 0.13 M) were added. The tube was sealed and the reaction mixture was heated with vigorous stirring for 48 h, then cooled to room temperature and concentrated *in vacuo*. The crude mixture was suspended in a small quantity of DMSO and subjected to flash column chromatography on silica gel (gradient elution, 0-50% EtOAc in petroleum ether).

Preparation of 4,6-diphenylpyrimidin-2-amine

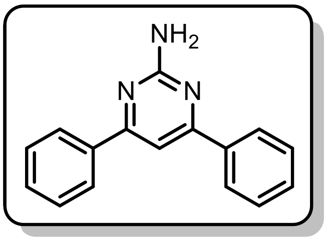

Following general procedure E, using **4a** (133 mg, 0.48 mmol), iodobenzene (27 µL, 0.24 mmol), $(Ph_3P)_2PdCl_2$ (17 mg, 0.02 mmol), Cs_2CO_3 (235 mg, 0.72 mmol), Ag_2O (56 mg, 0.24 mmol) and H_2O/THF (50% v/v, 1.8 mL). The title compound was obtained (36 mg, 61%) as a colourless solid. **M.p.** 135 °C (lit[6] = 134-136 °C); **^{1}H NMR (400 MHz, CDCl$_3$)** δ_H ppm 5.41 (s, 2H), 7.46 (s, 1H), 7.47 – 7.52 (m, 6H), 8.03 – 8.09 (m, 4H); **^{13}C NMR (101 MHz, CDCl$_3$)** δ_C ppm 104.4, 127.3, 128.9, 130.6, 137.9, 163.8, 166.4; (the data were in agreement with those published)[6]

Preparation of 4-(3,5-dimethylphenyl)-6-phenylpyrimidin-2-amine

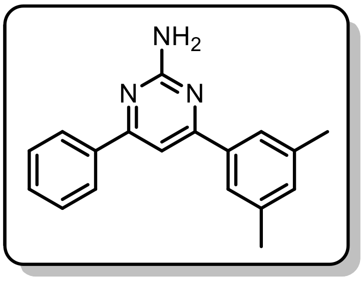

Following general procedure E, using **4a** (133 mg, 0.48 mmol), 1-iodo-3,5-dimethylbenzene (35 µL, 0.24 mmol), $(Ph_3P)_2PdCl_2$ (17 mg, 0.02 mmol), Cs_2CO_3 (235 mg, 0.72 mmol), Ag_2O (56 mg, 0.24 mmol) and H_2O/THF (50% v/v, 1.8 mL). The title compound was obtained (39 mg, 59%) as a colourless solid. **M.p.** 155 °C; **^{1}H NMR (400 MHz, CDCl$_3$)** δ_H ppm 2.42 (s, 6H), 5.37 (s, 2H), 7.13 (m, 1H), 7.44 (s, 1H), 7.47 – 7.52 (m, 3H), 7.66 (m, 2H), 8.03 – 8.09 (m, 2H); **^{13}C NMR (101 MHz, CDCl$_3$)** δ_C ppm 21.5, 104.6, 125.1, 127.3, 128.9, 130.5, 132.2, 137.9, 138.0, 138.5, 163.8, 166.2, 166.8; **FTIR (neat)** ν_{max} / cm^{-1} 3420(m), 3158(m), 2986(m), 1624(m), 1572(s), 1541(s); **HRMS: (ESI-TOF)** *m/z* [M+H]$^+$ calculated for $C_{18}H_{18}N_3$ 276.1495, found 276.1492.

HMBC Assignment of 10b:

Correlation of H3 & H6 with C5. Correlation of H8 and C7.

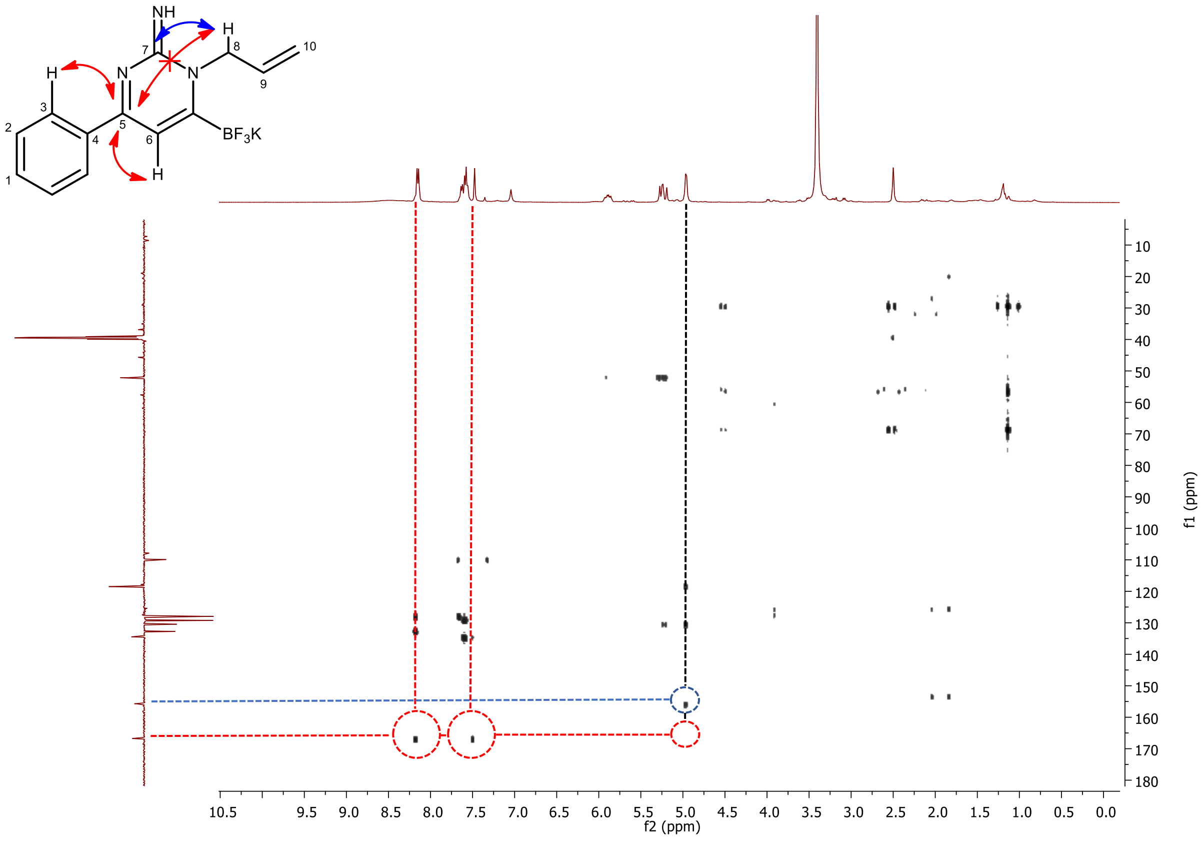

NMR spectra for all novel isolated compounds

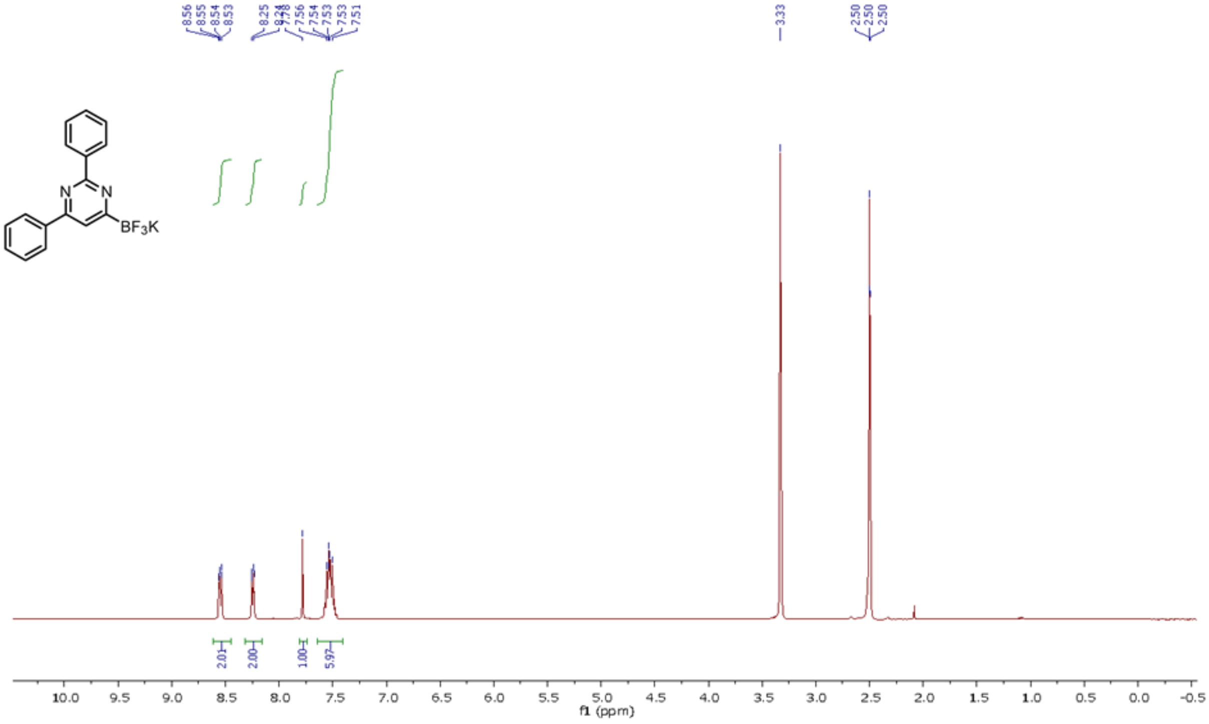

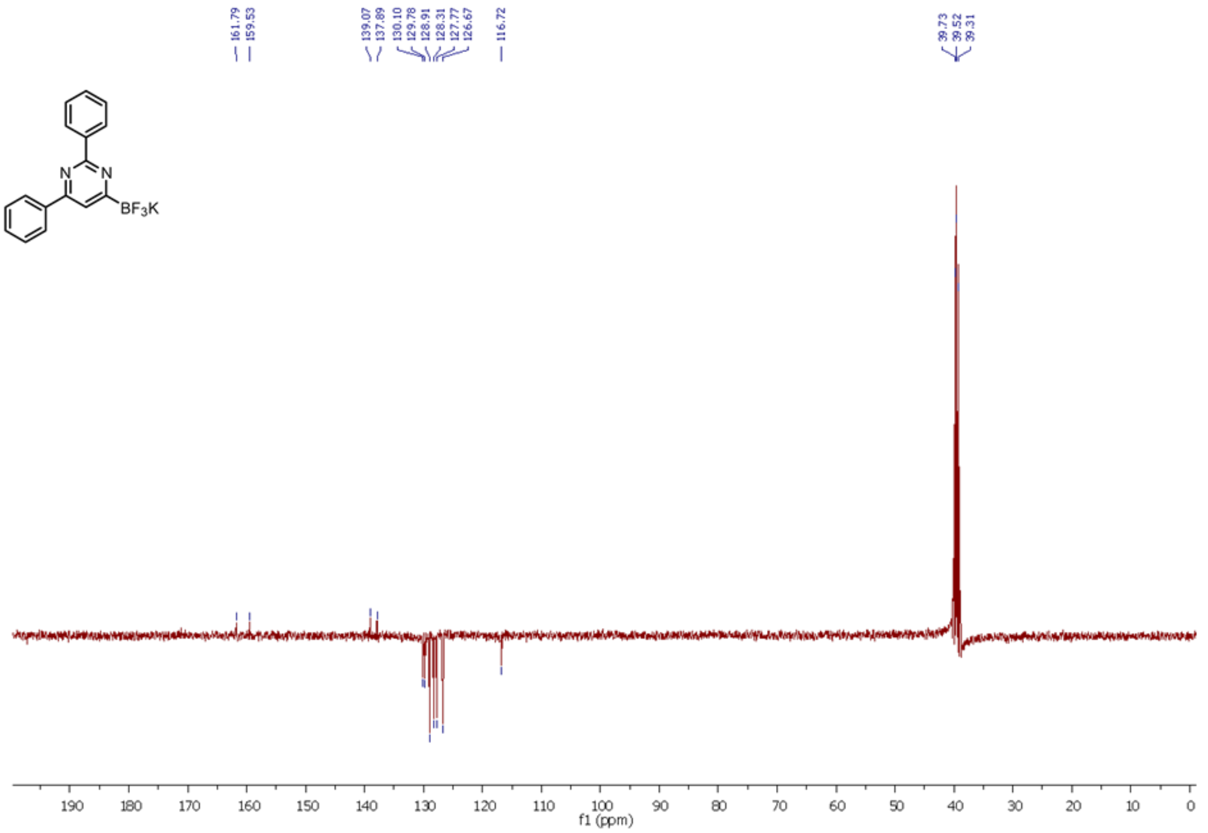

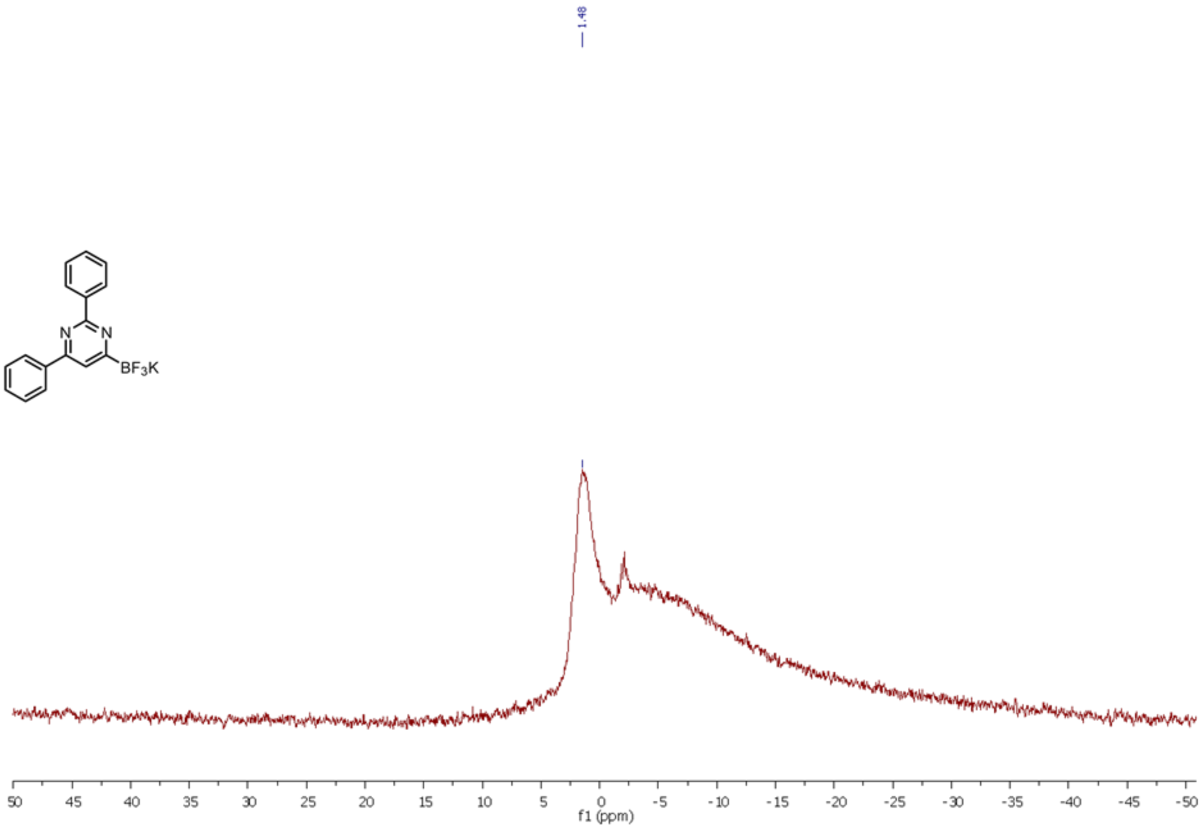
1.48
N
N
BF3K
f1 (ppm)

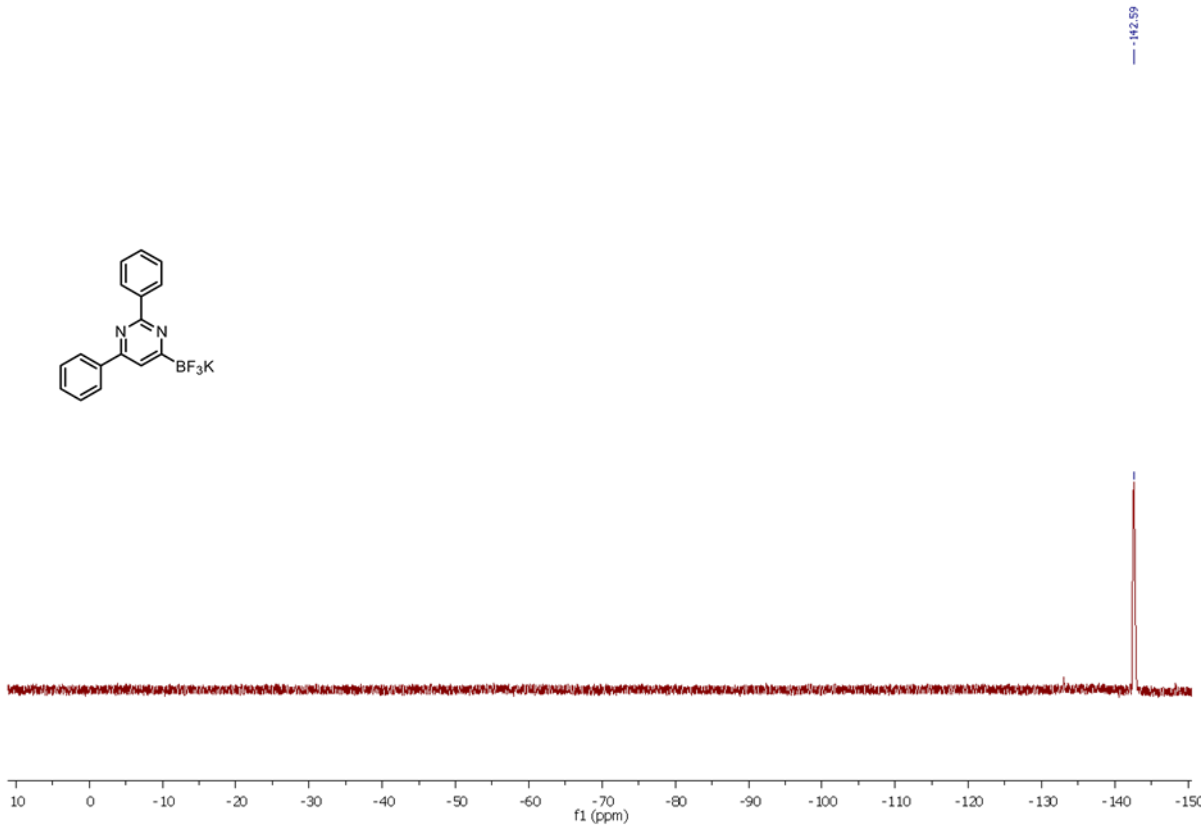
-142.59
N
N
BF3K
f1 (ppm)

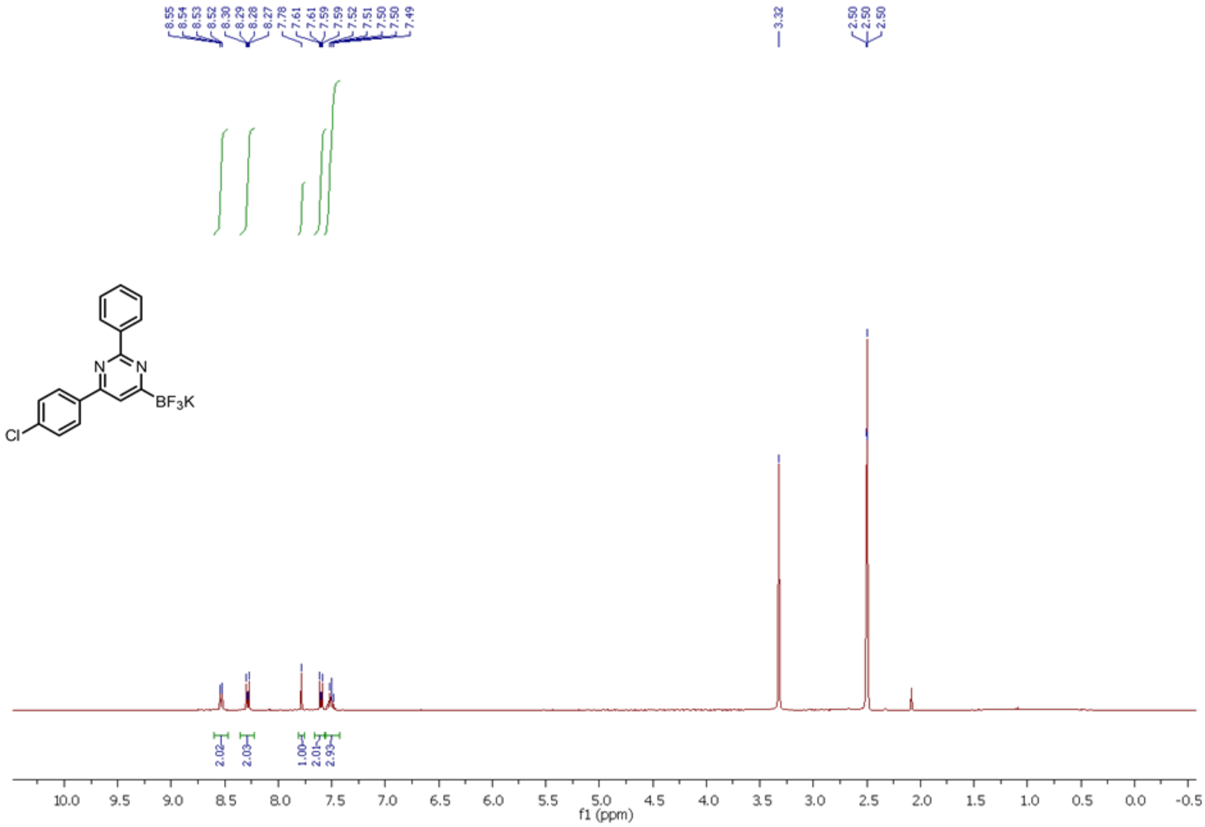
8.55
8.54
8.53
8.52
8.30
8.29
8.28
8.27
7.78
7.61
7.61
7.59
7.59
7.52
7.51
7.50
7.50
7.49
3.32
2.50
2.50
2.50
N
N
BF3K
Cl
2.02
2.03
1.00
2.01
2.93
f1 (ppm)

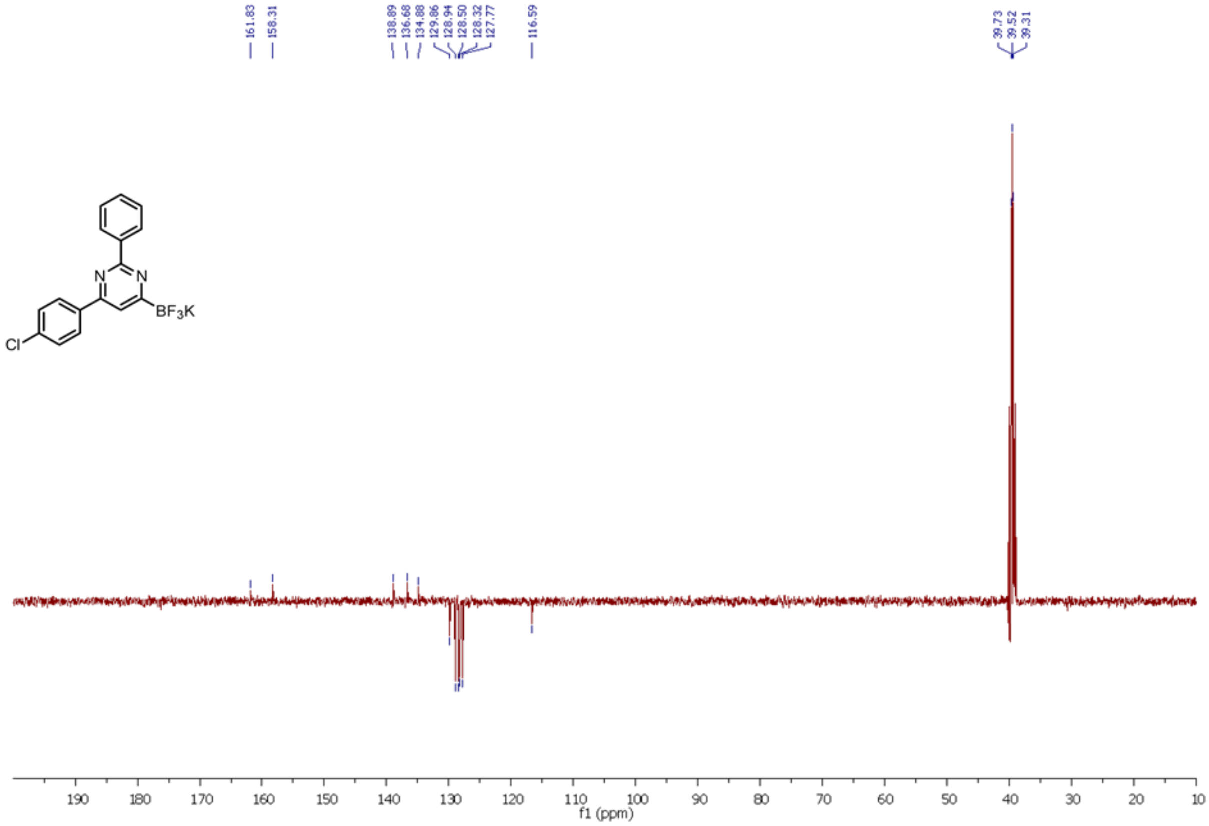
161.83
158.31
138.89
136.68
134.88
129.86
128.94
128.50
128.32
127.77
116.59
39.73
39.52
39.31
N
N
BF3K
Cl
f1 (ppm)

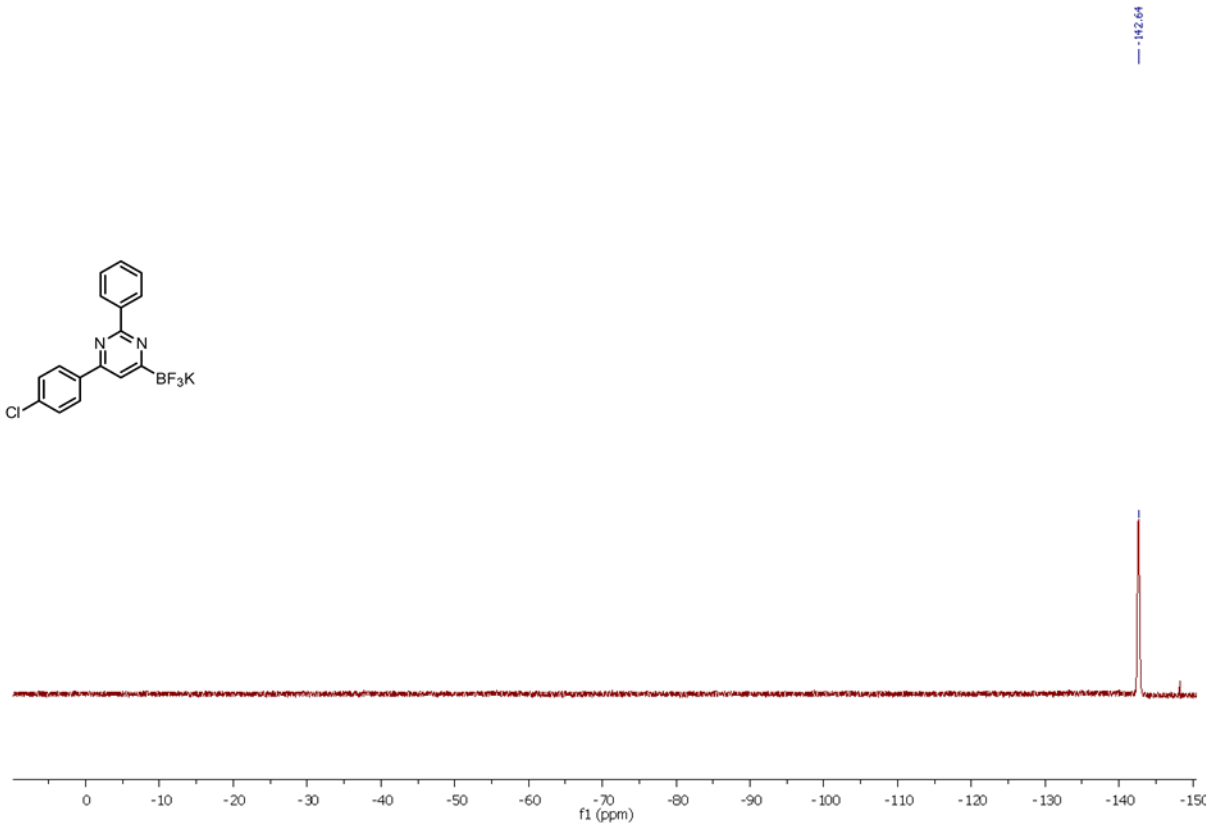
-142.64
N
N
BF3K
Cl
0 -10 -20 -30 -40 -50 -60 -70 -80 -90 -100 -110 -120 -130 -140 -150
f1 (ppm)

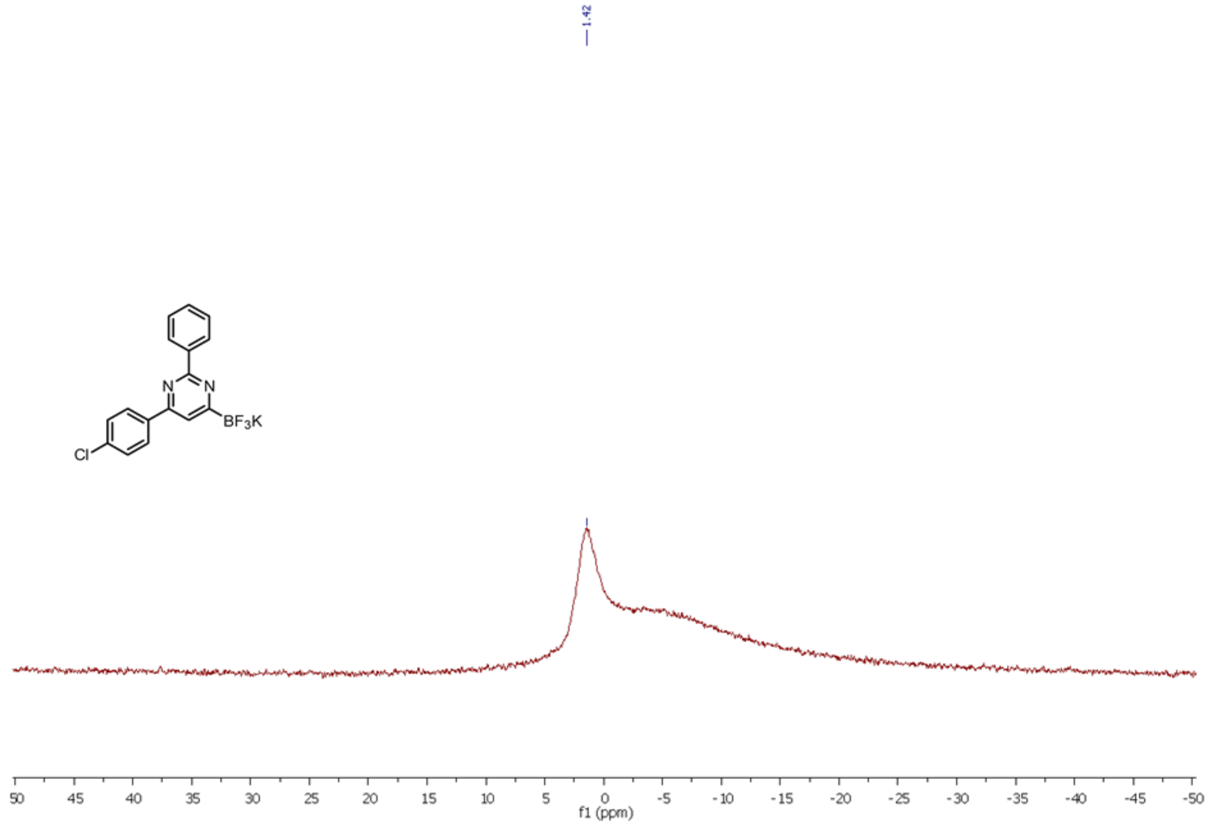
1.42
N
N
BF3K
Cl
50 45 40 35 30 25 20 15 10 5 0 -5 -10 -15 -20 -25 -30 -35 -40 -45 -50
f1 (ppm)

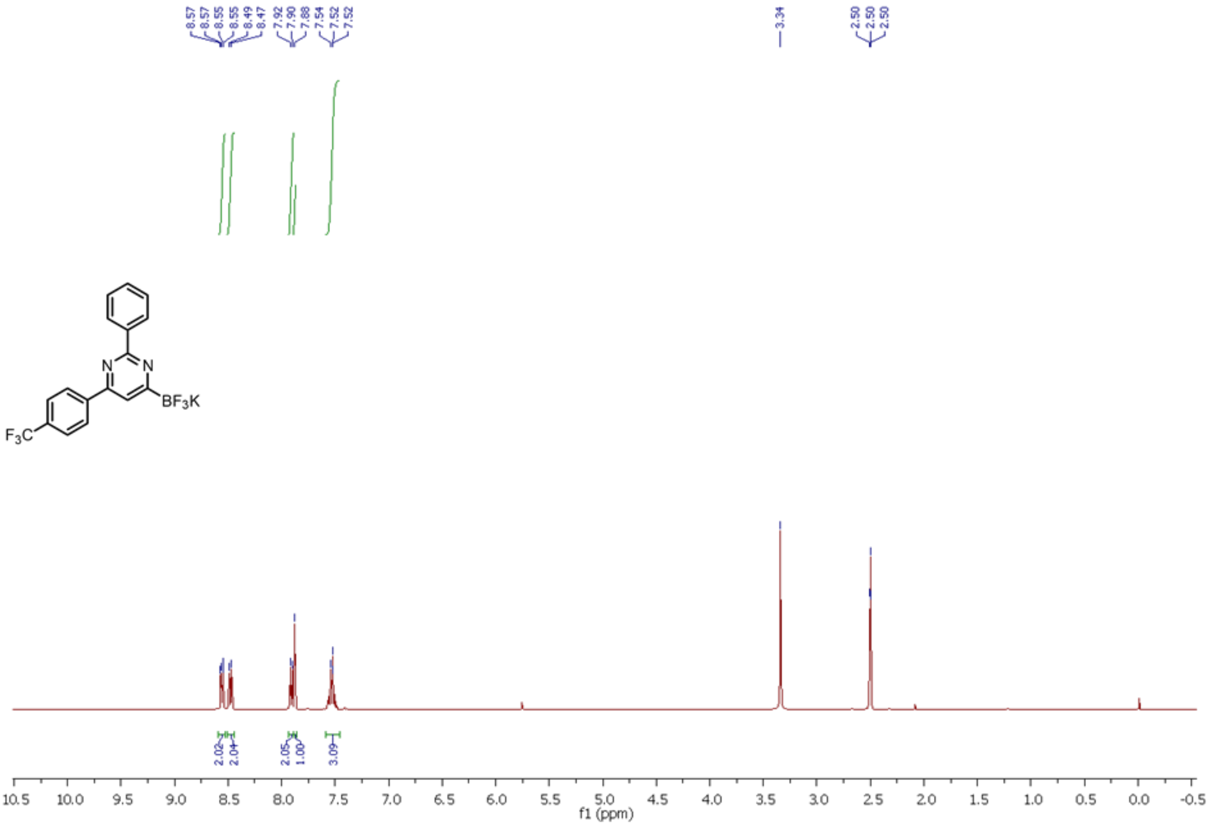

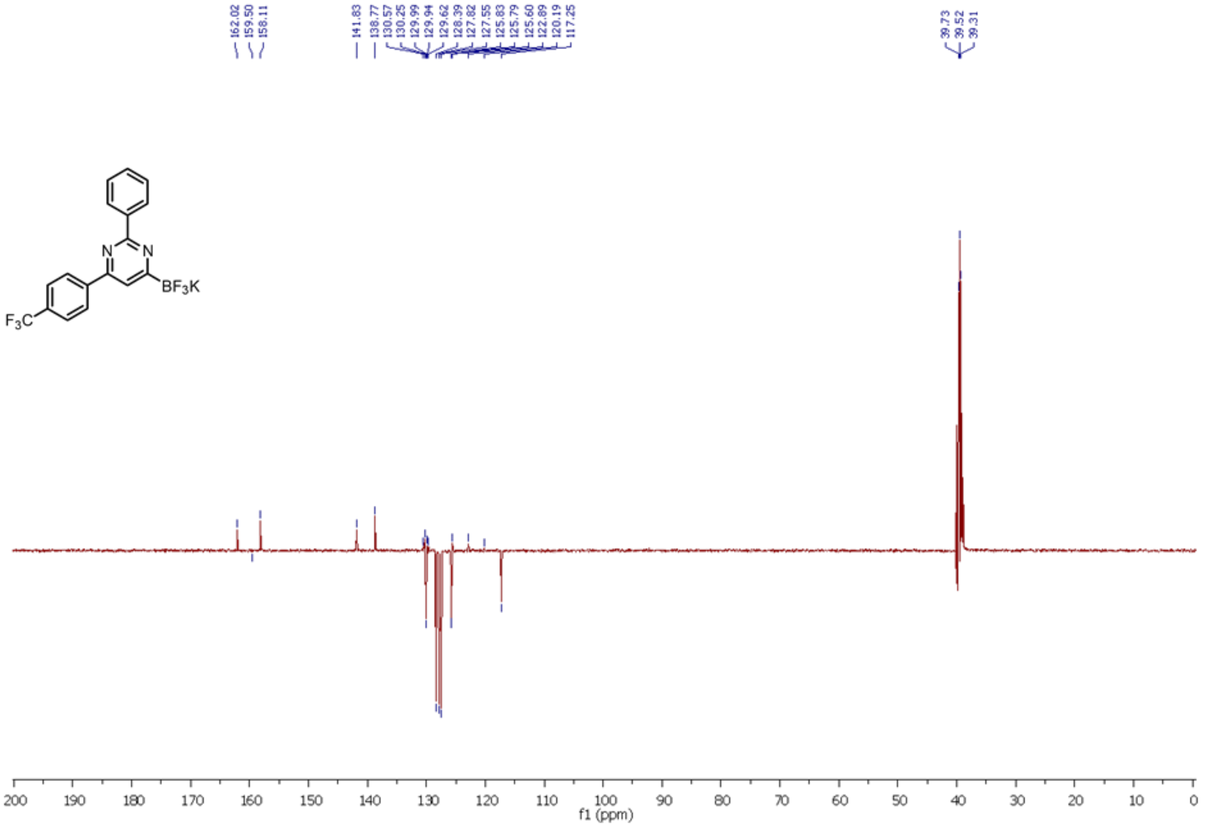

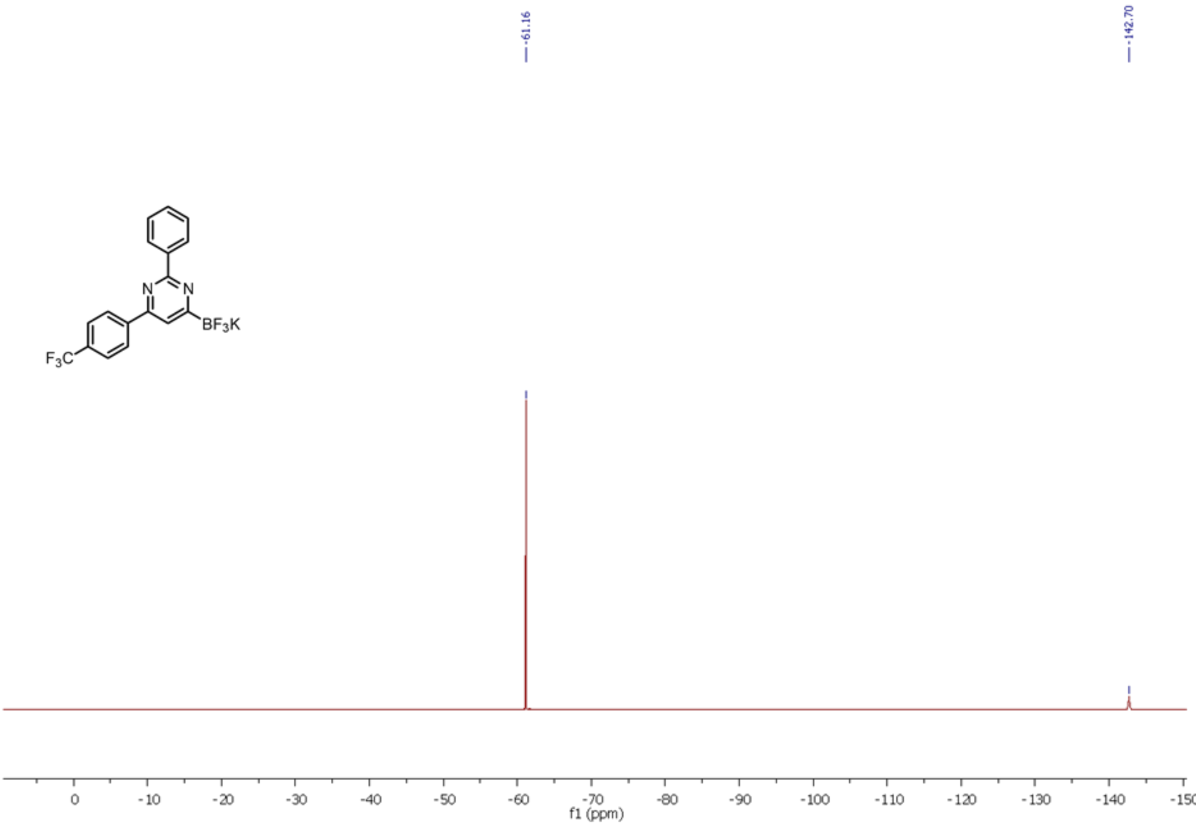
-61.16
-142.70
N
N
BF3K
F3C
0
-10
-20
-30
-40
-50
-60
-70
-80
-90
-100
-110
-120
-130
-140
f1 (ppm)

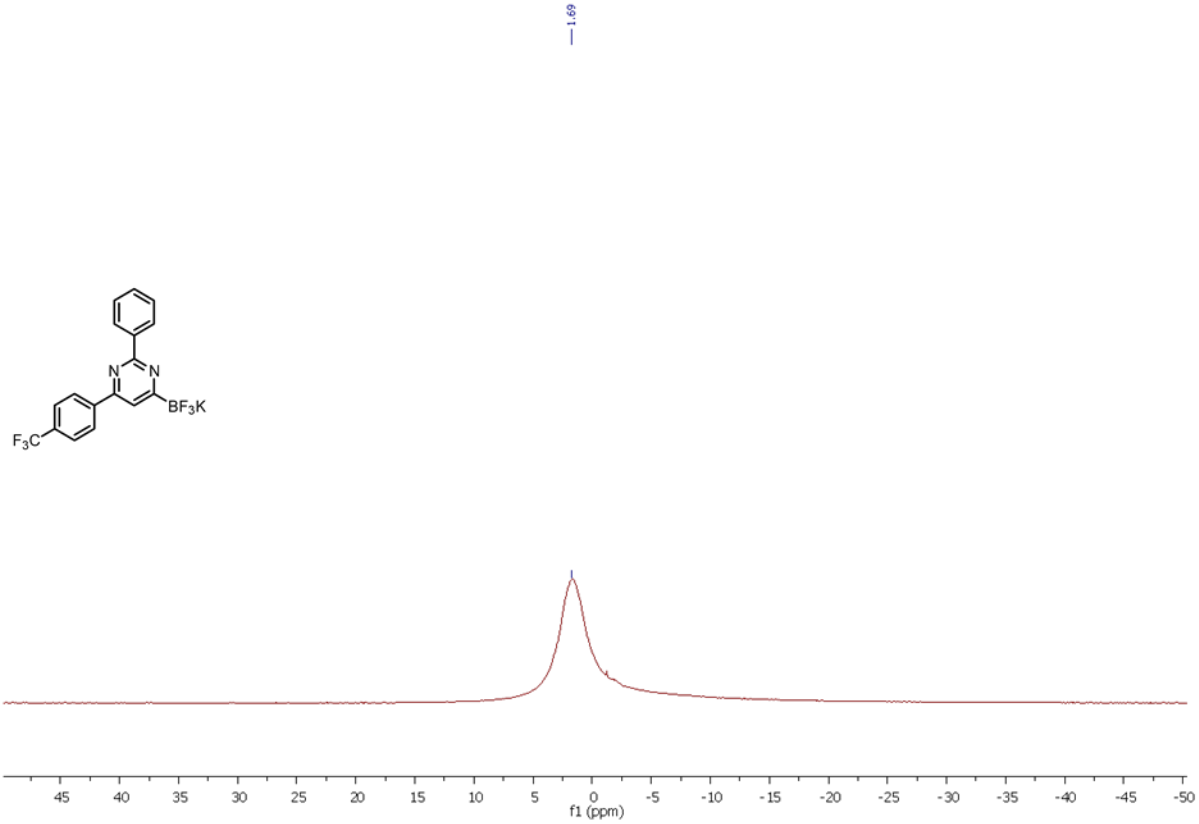
1.69
N
N
BF3K
F3C
45
40
35
30
25
20
15
10
5
0
-5
-10
-15
-20
-25
-30
-35
-40
-45
-50
f1 (ppm)

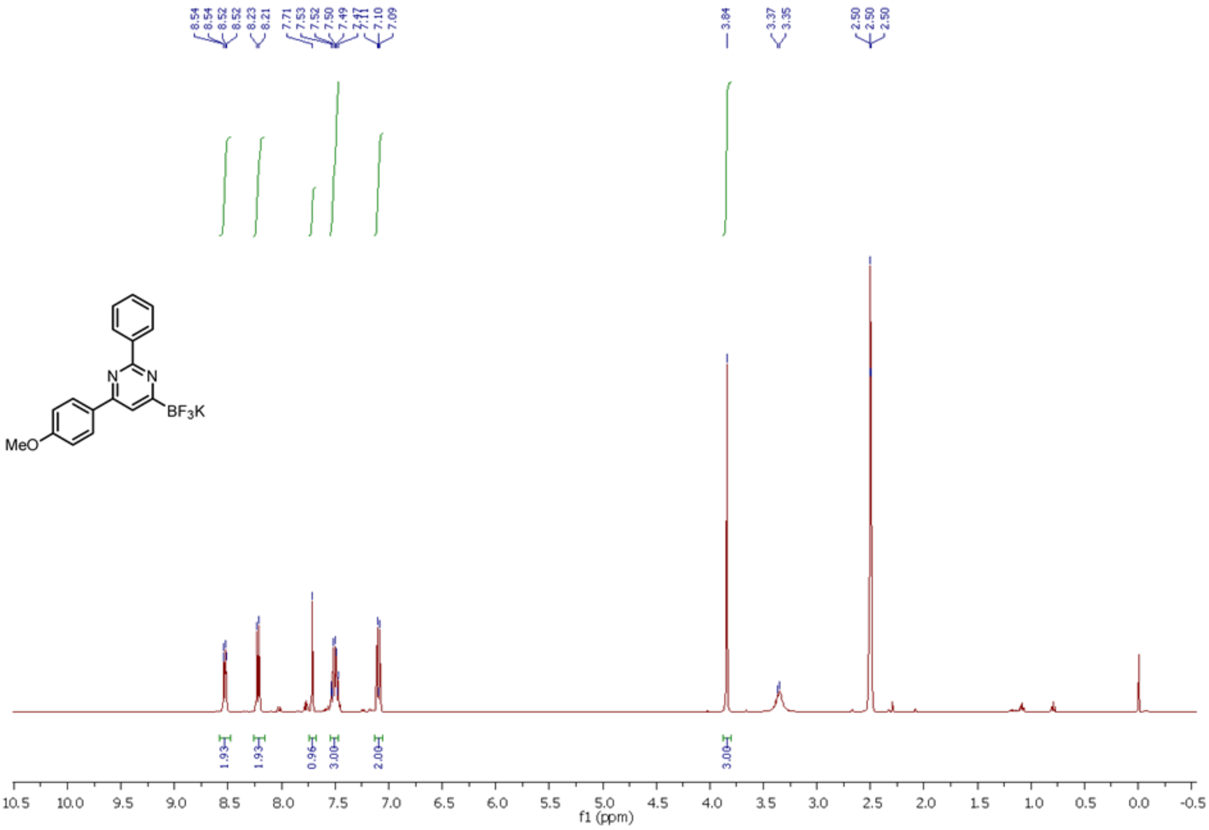

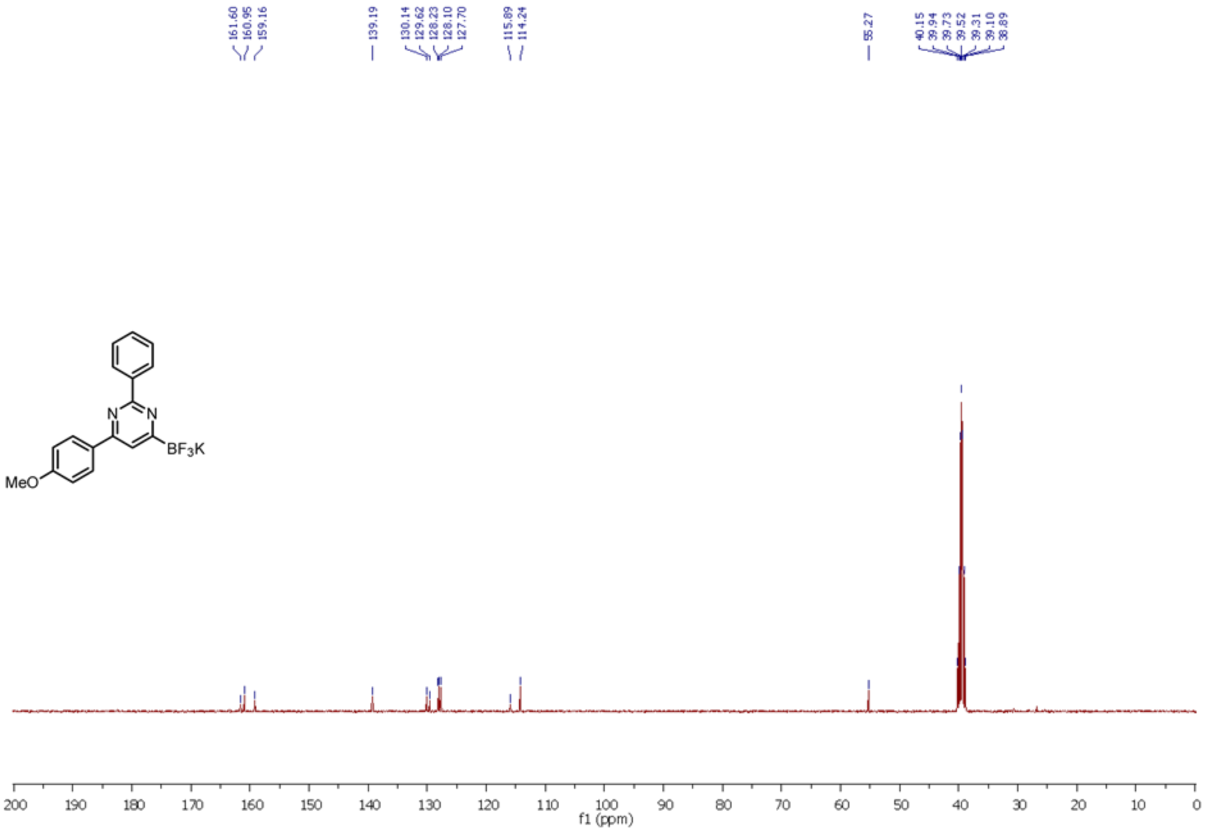

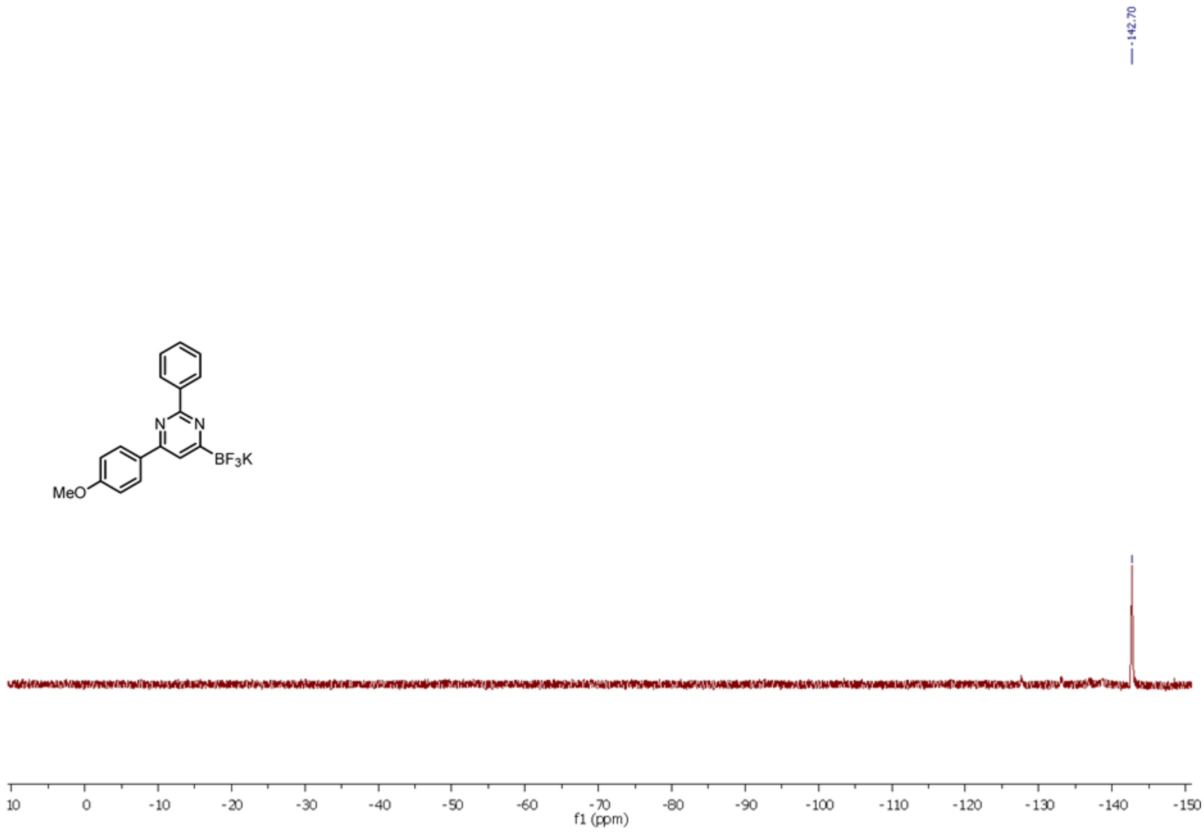
-142.70
N
N
BF3K
MeO
10
0
-10
-20
-30
-40
-50
-60
-70
-80
-90
-100
-110
-120
-130
-140
-150
f1 (ppm)

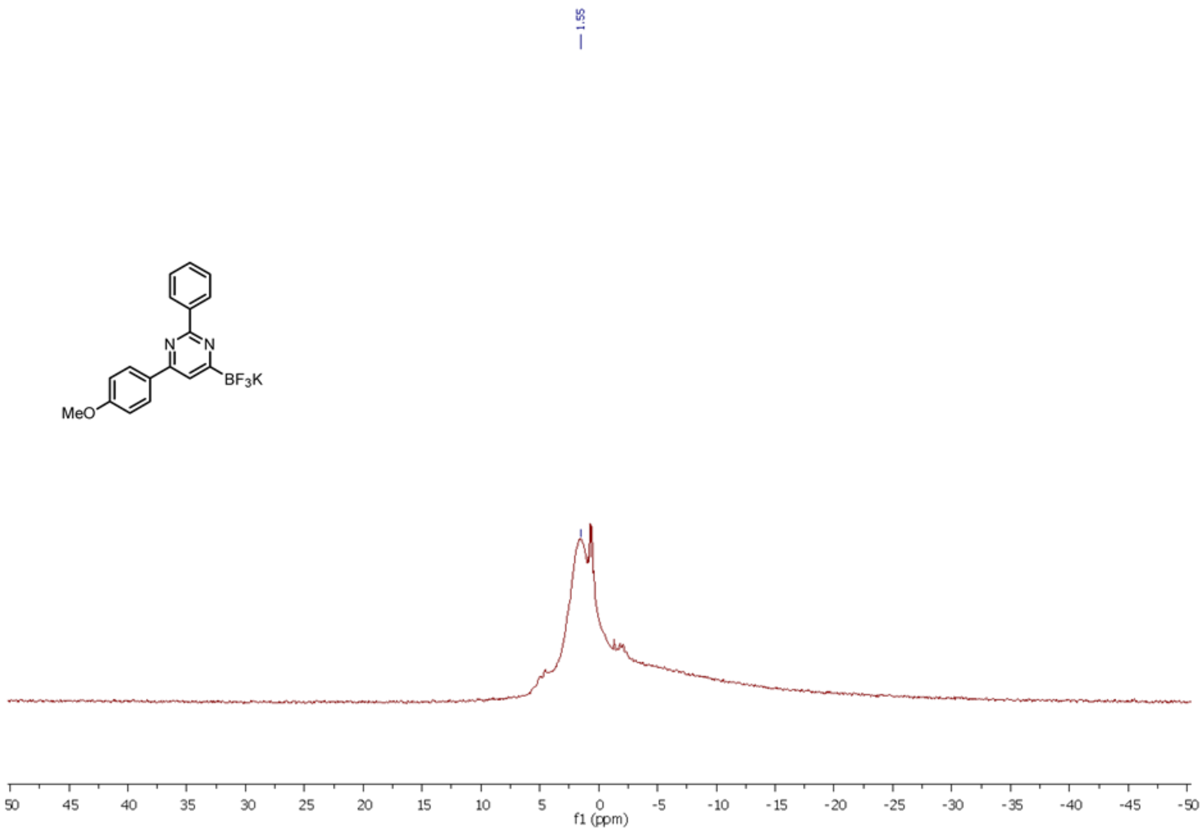
1.55
N
N
BF3K
MeO
50
45
40
35
30
25
20
15
10
5
0
-5
-10
-15
-20
-25
-30
-35
-40
-45
-50
f1 (ppm)

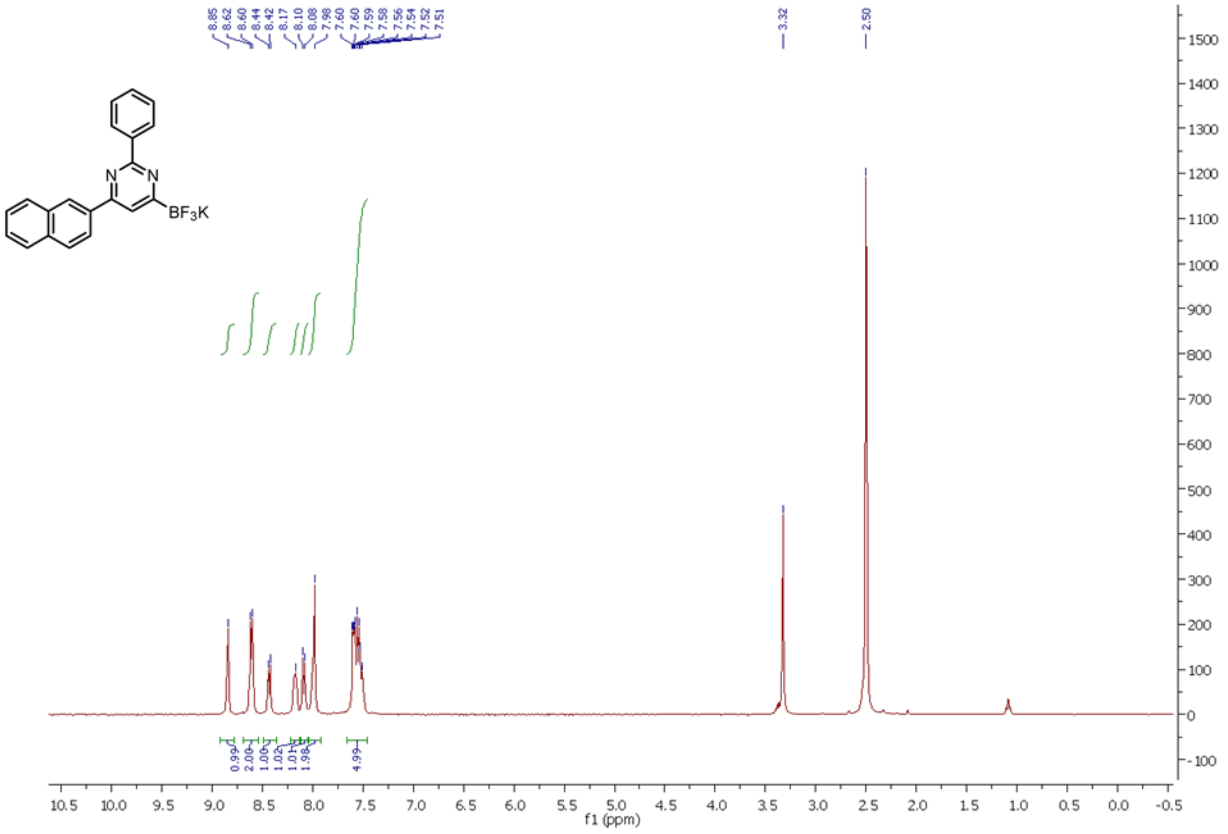

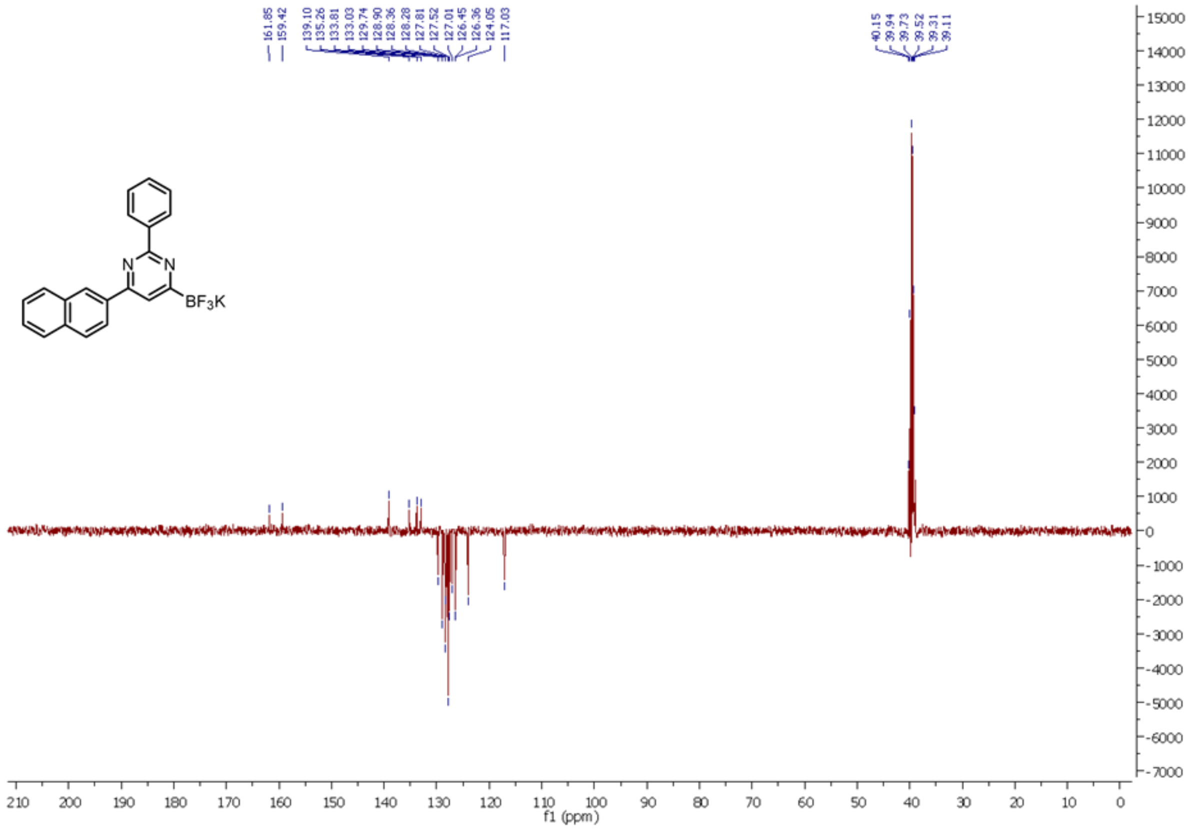

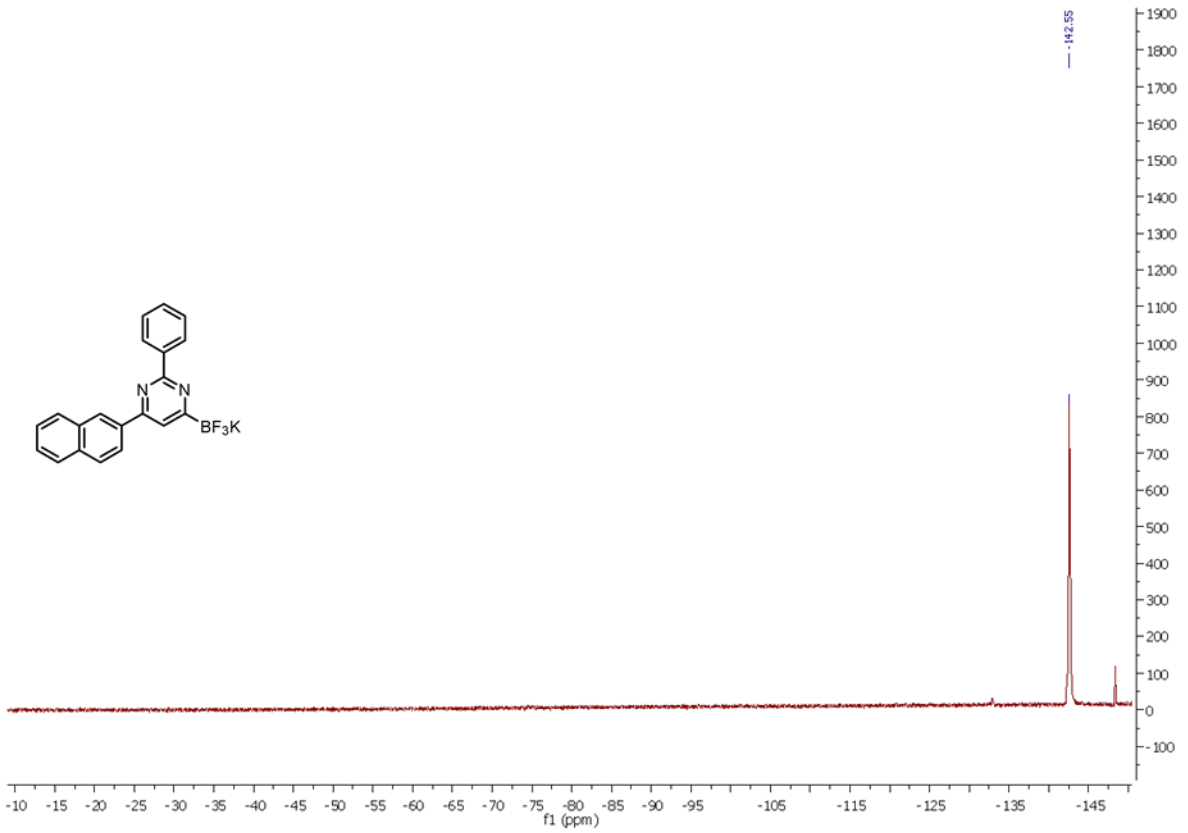
-142.55
f1 (ppm)

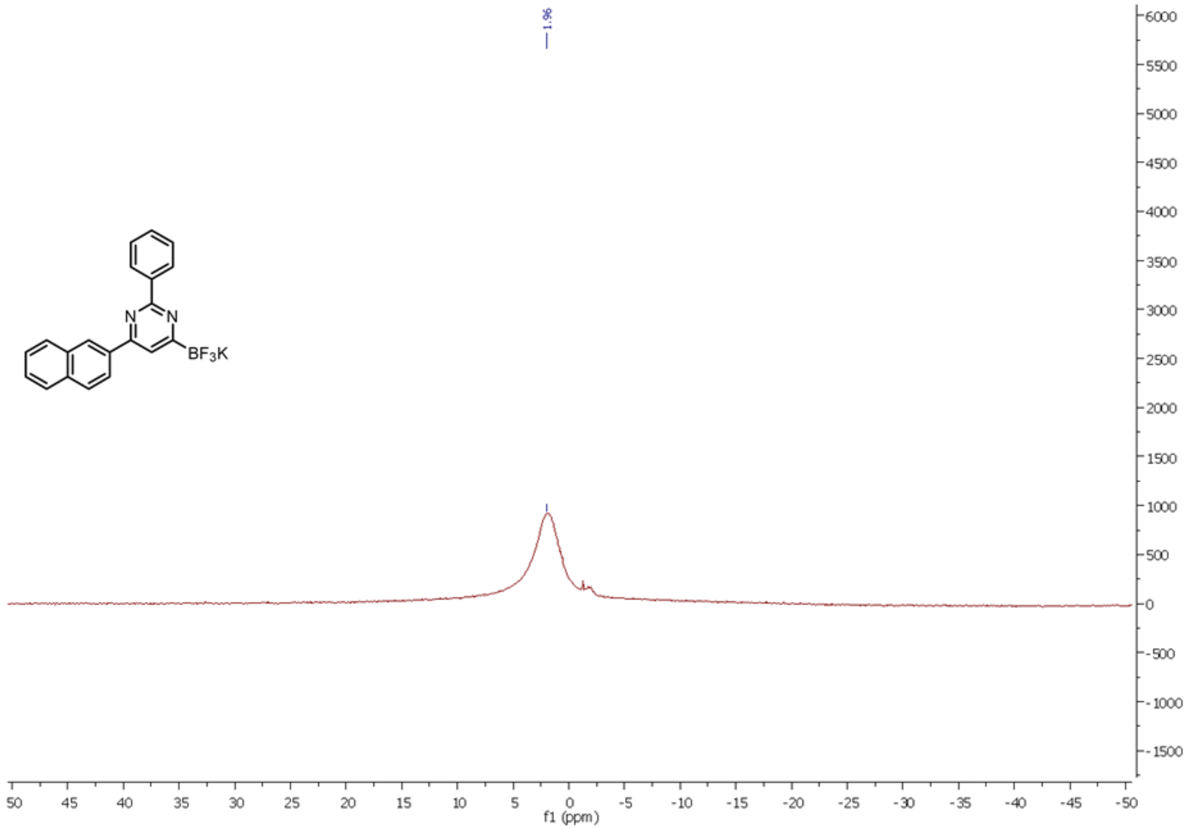
1.96
f1 (ppm)

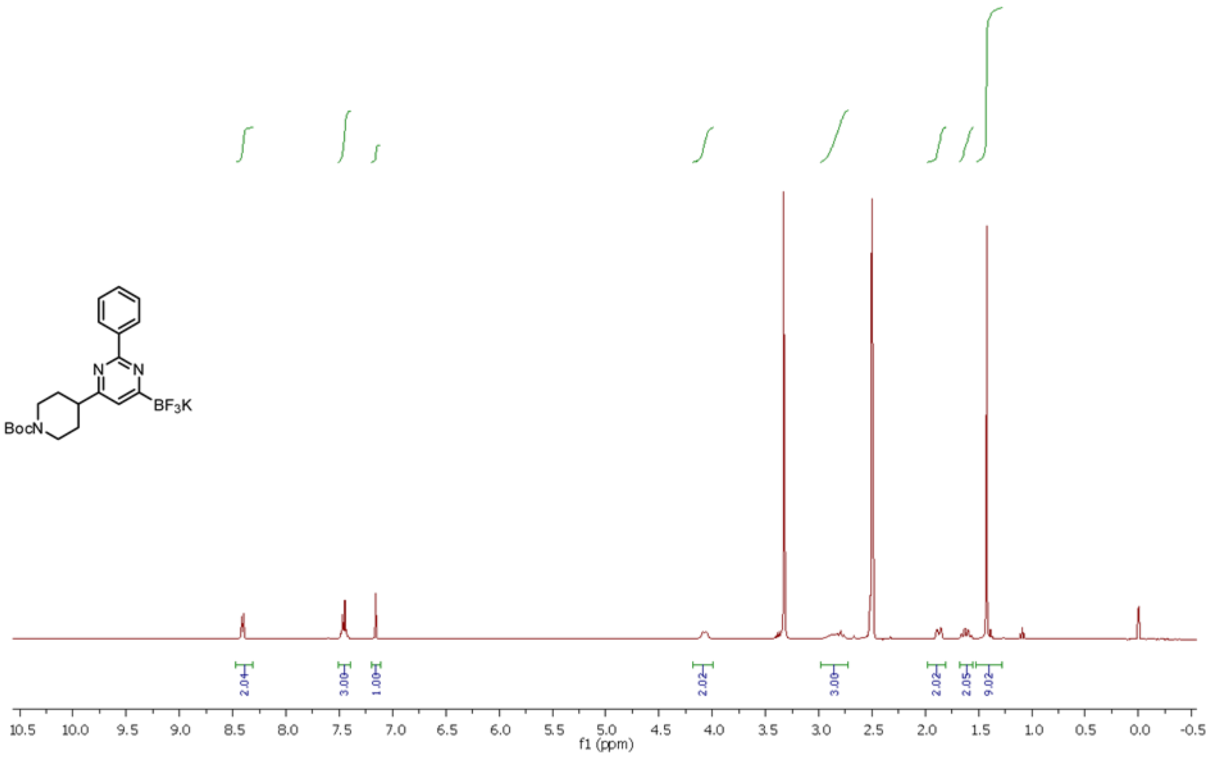
BocN
N
N
BF3K
2.04
3.00
1.00
2.02
3.00
2.02
2.05
9.02
10.5 10.0 9.5 9.0 8.5 8.0 7.5 7.0 6.5 6.0 5.5 5.0 4.5 4.0 3.5 3.0 2.5 2.0 1.5 1.0 0.5 0.0 -0.5
f1 (ppm)

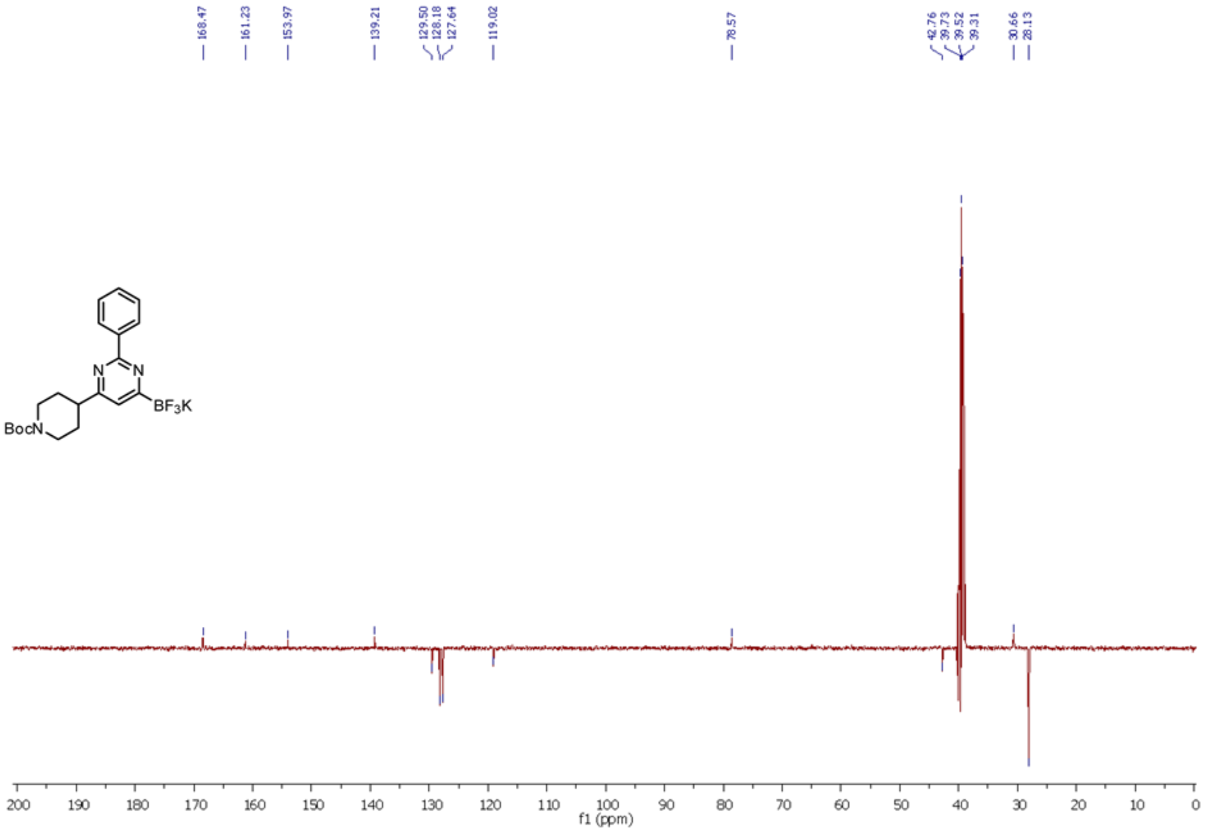
168.47
161.23
153.97
139.21
129.50
128.18
127.64
119.02
78.57
42.76
39.73
39.52
39.31
30.66
28.13
BocN
N
N
BF3K
200 190 180 170 160 150 140 130 120 110 100 90 80 70 60 50 40 30 20 10 0
f1 (ppm)

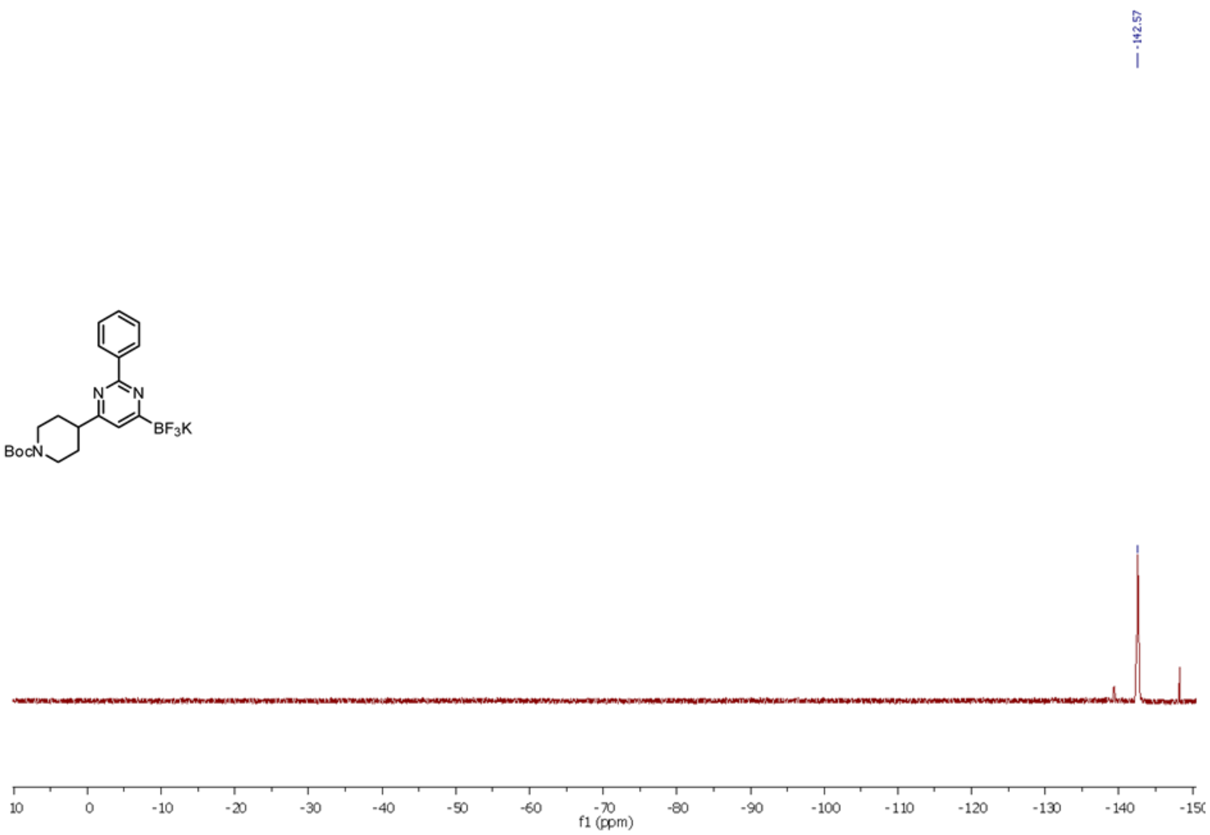
-142.57
BocN
N
N
BF3K
10 0 -10 -20 -30 -40 -50 -60 -70 -80 -90 -100 -110 -120 -130 -140 -150
f1 (ppm)

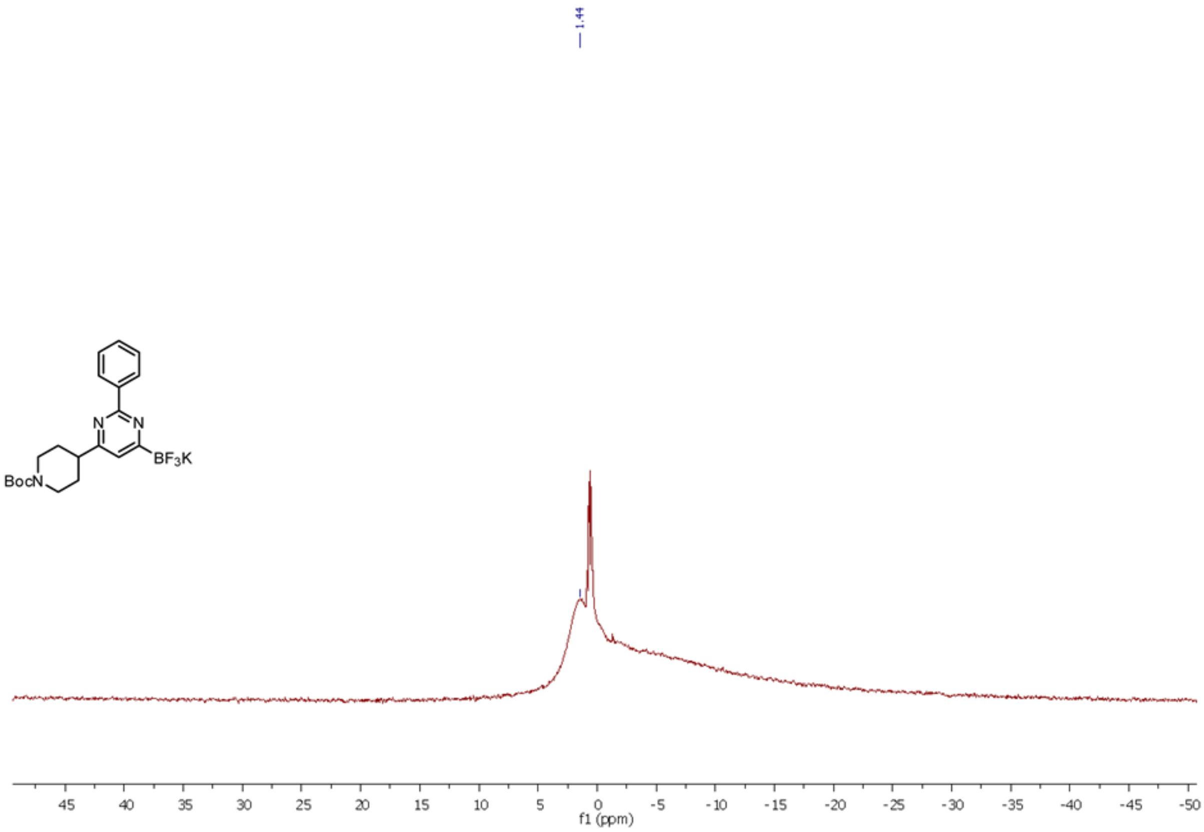
1.44
BocN
N
N
BF3K
45 40 35 30 25 20 15 10 5 0 -5 -10 -15 -20 -25 -30 -35 -40 -45 -50
f1 (ppm)

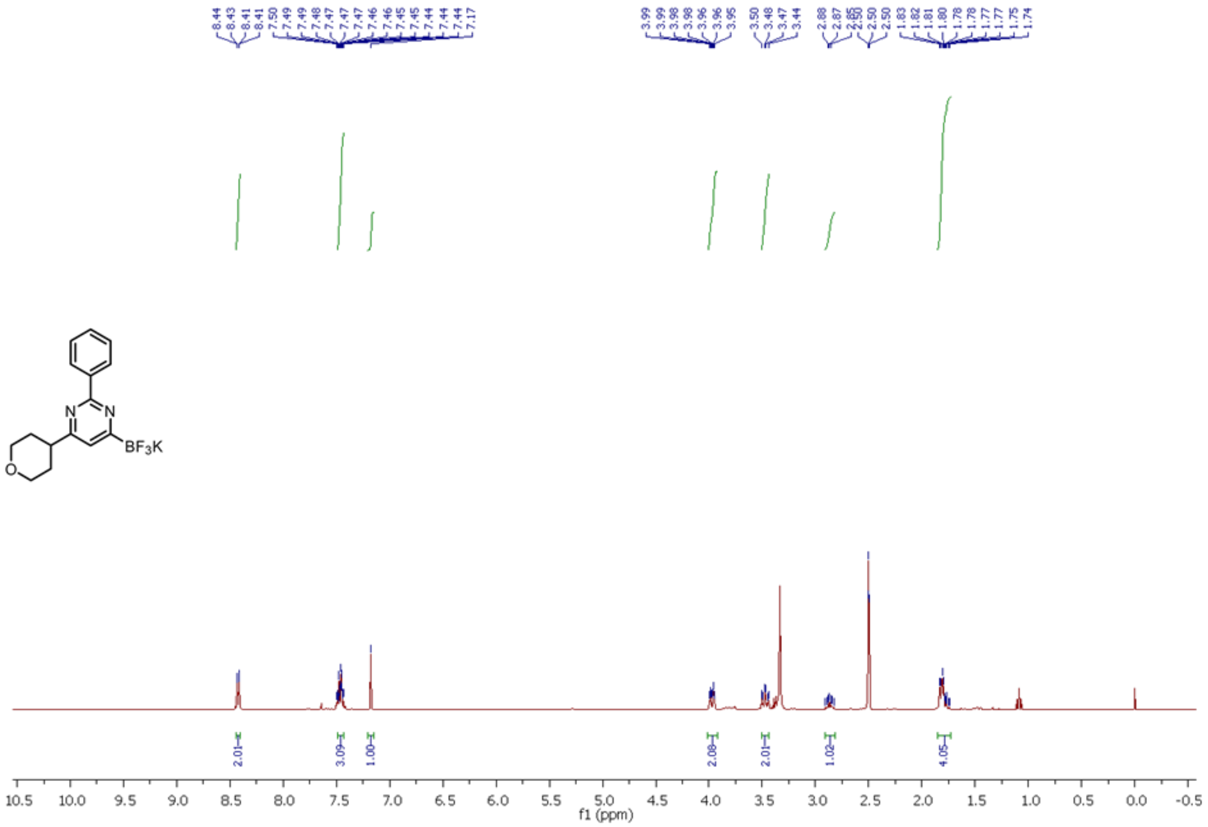

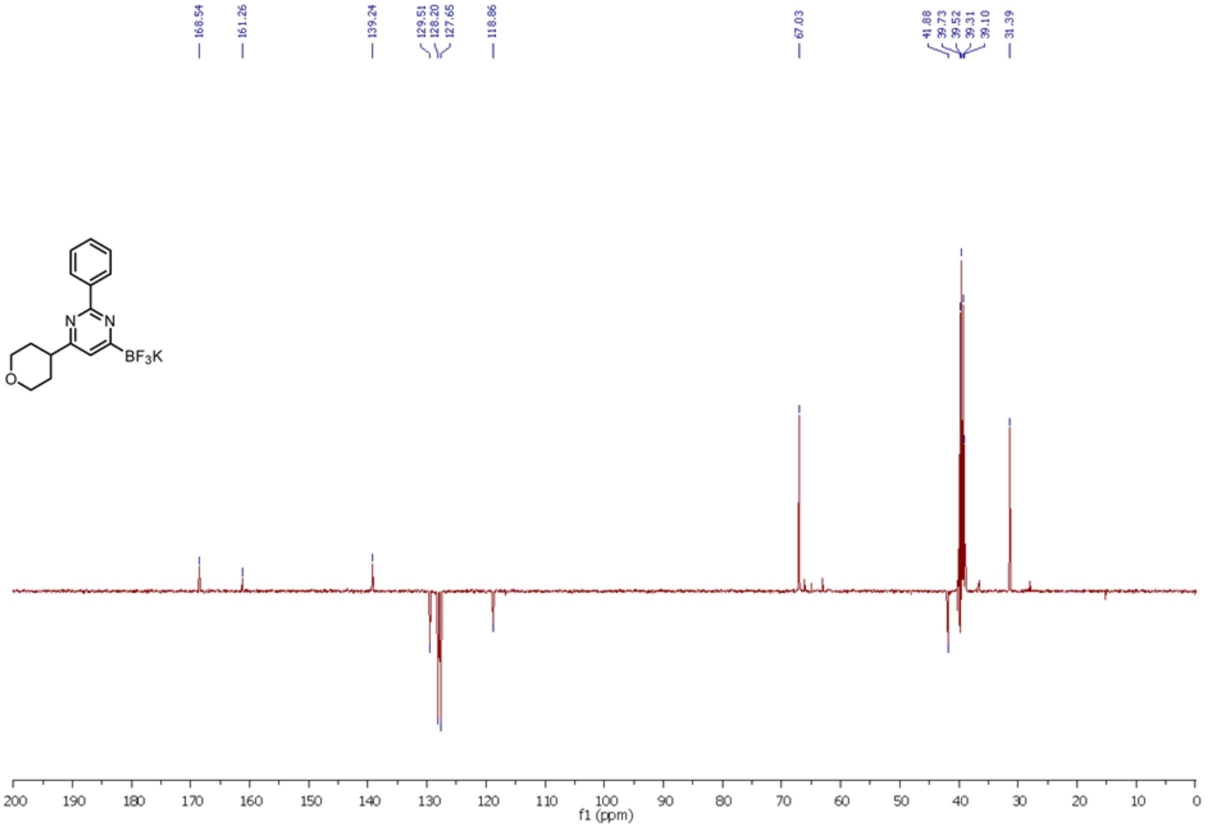

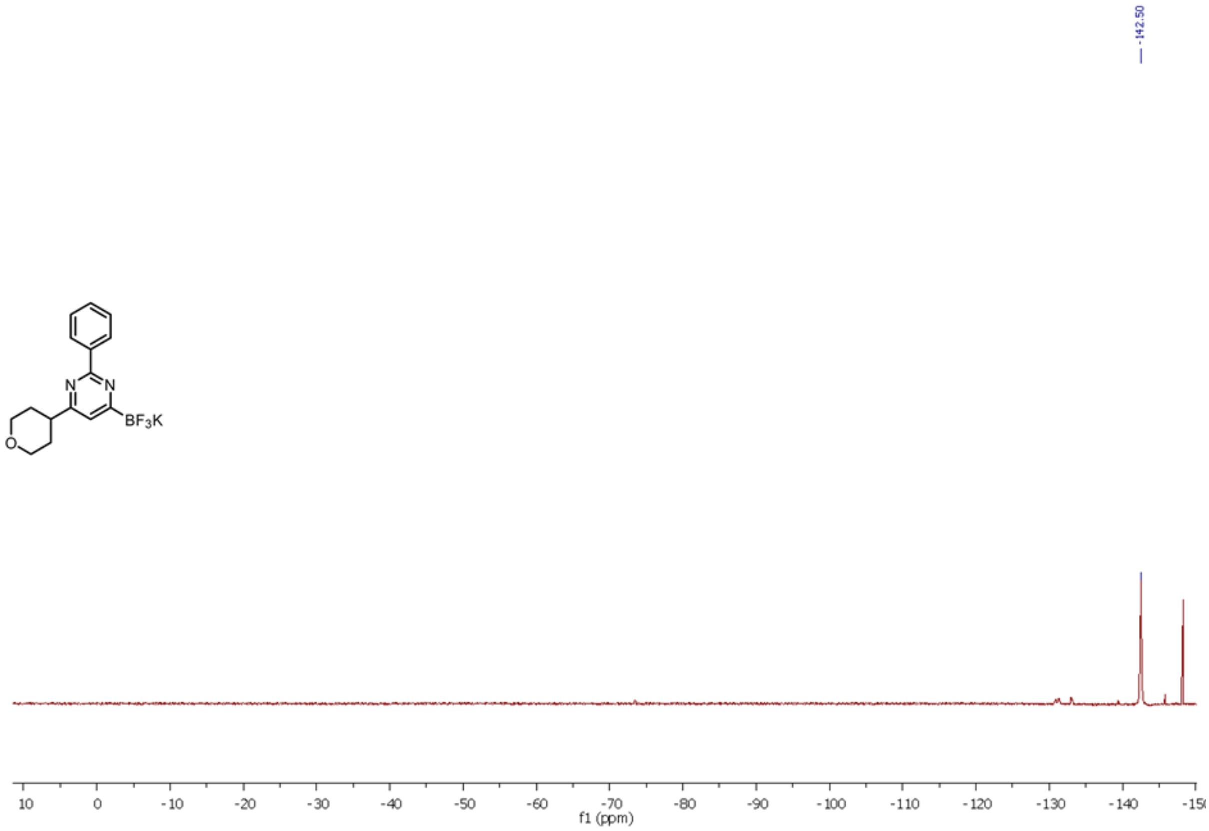
-142.50
N
N
BF3K
O
10
0
-10
-20
-30
-40
-50
-60
-70
-80
-90
-100
-110
-120
-130
-140
f1 (ppm)

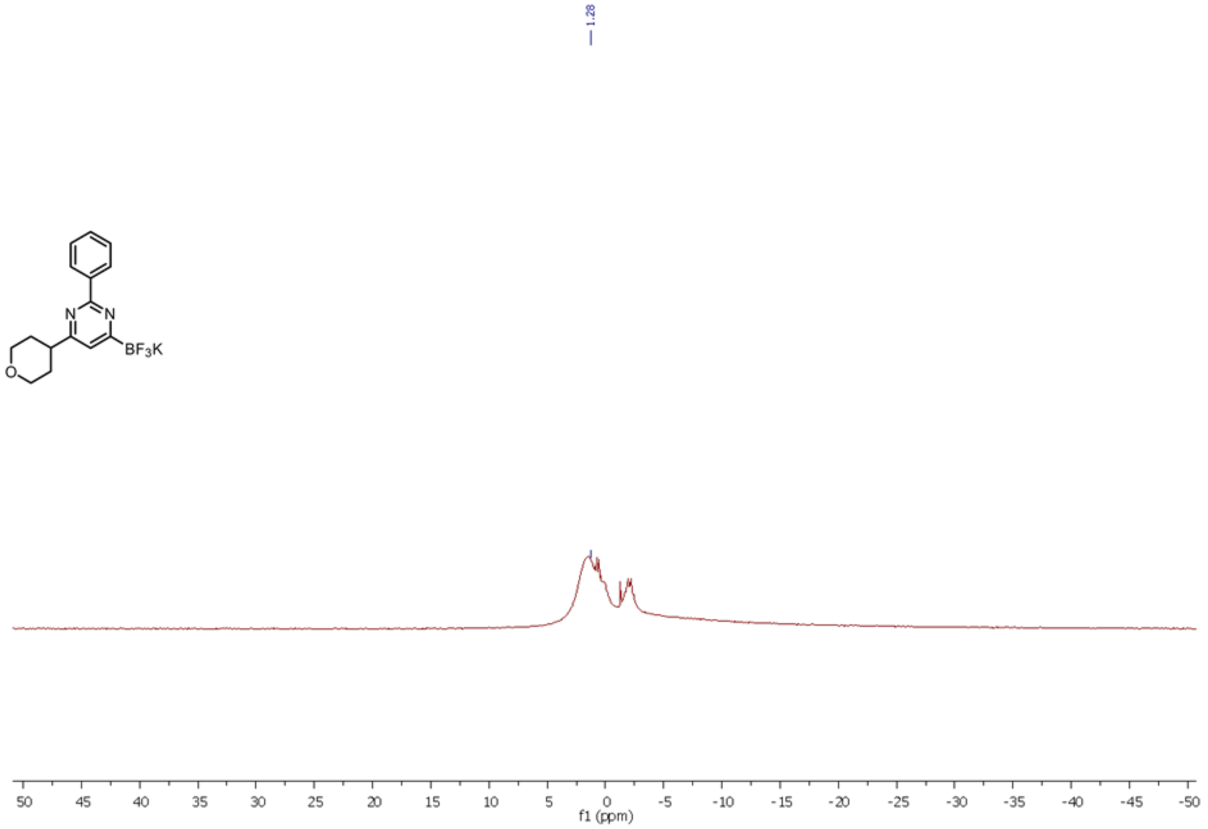
1.28
N
N
BF3K
O
50
45
40
35
30
25
20
15
10
5
0
-5
-10
-15
-20
-25
-30
-35
-40
-45
-50
f1 (ppm)

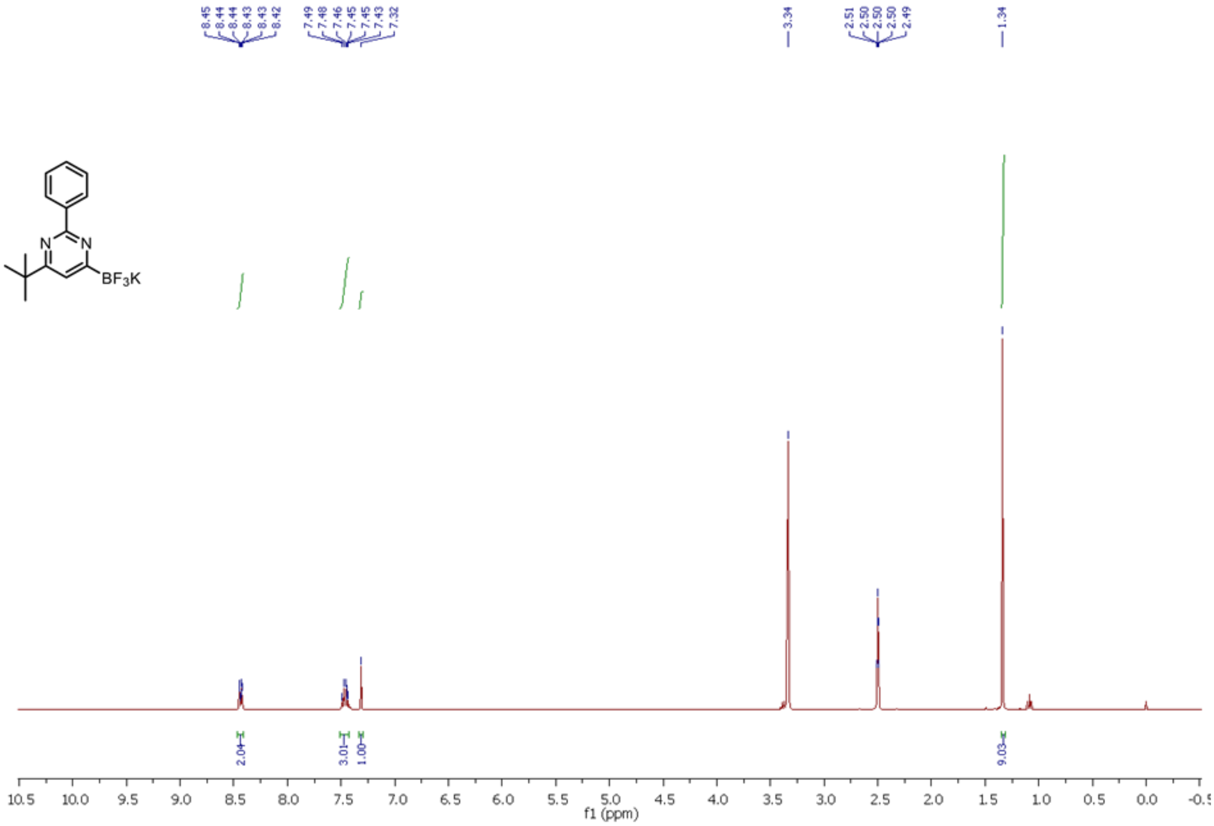

8.45
8.44
8.44
8.43
8.43
8.42
7.49
7.48
7.46
7.45
7.45
7.43
7.32
3.34
2.51
2.50
2.50
2.50
2.49
1.34
BF3K
N
N
2.04
3.01
1.00
9.03
f1 (ppm)

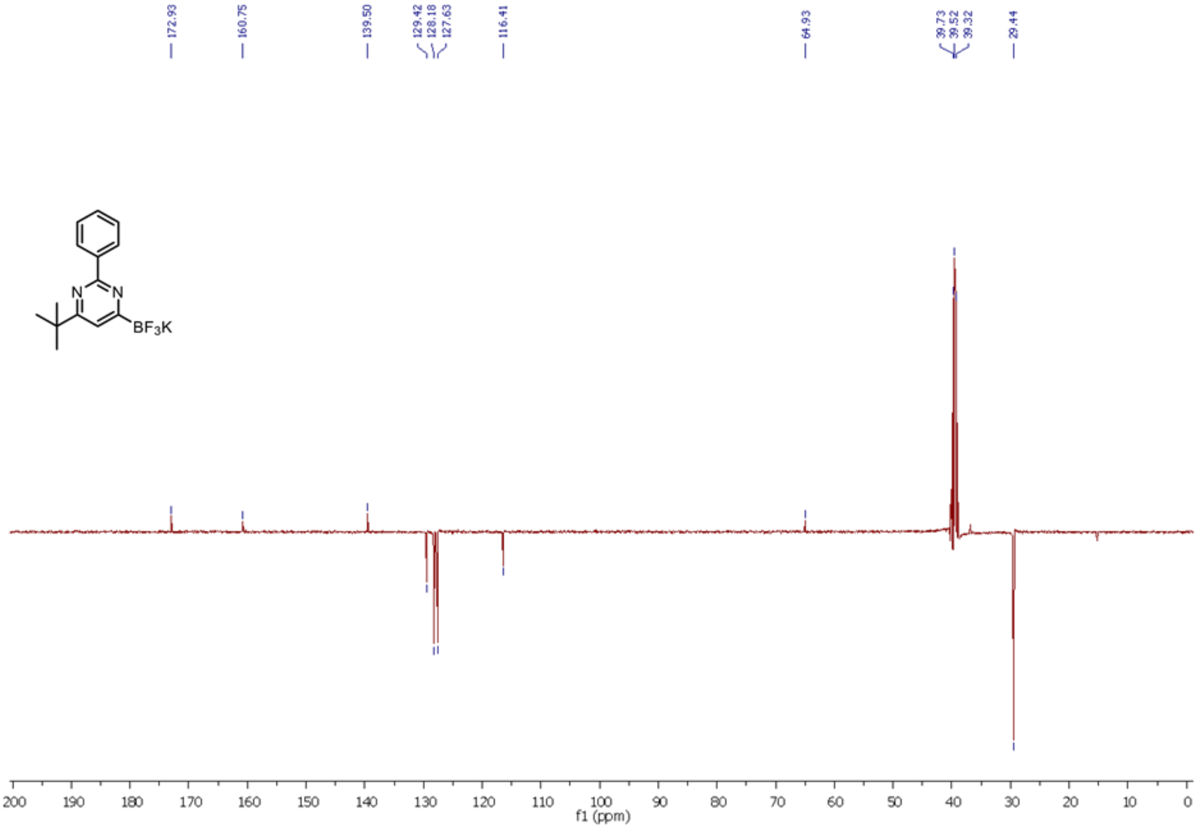

172.93
160.75
139.50
129.42
128.18
127.63
116.41
64.93
39.73
39.52
39.32
29.44
BF3K
N
N
f1 (ppm)

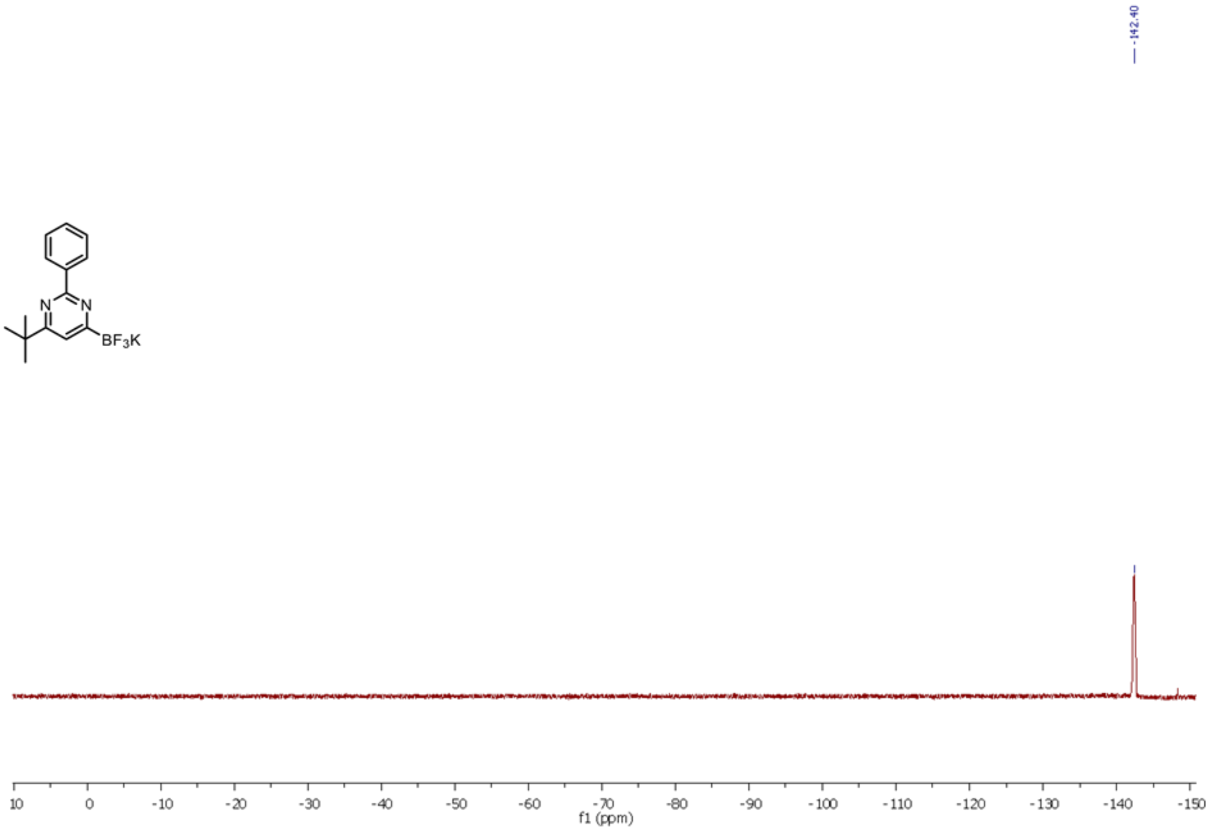
-142.40
BF3K
N
N
10
0
-10
-20
-30
-40
-50
-60
-70
-80
-90
-100
-110
-120
-130
-140
-150
f1 (ppm)

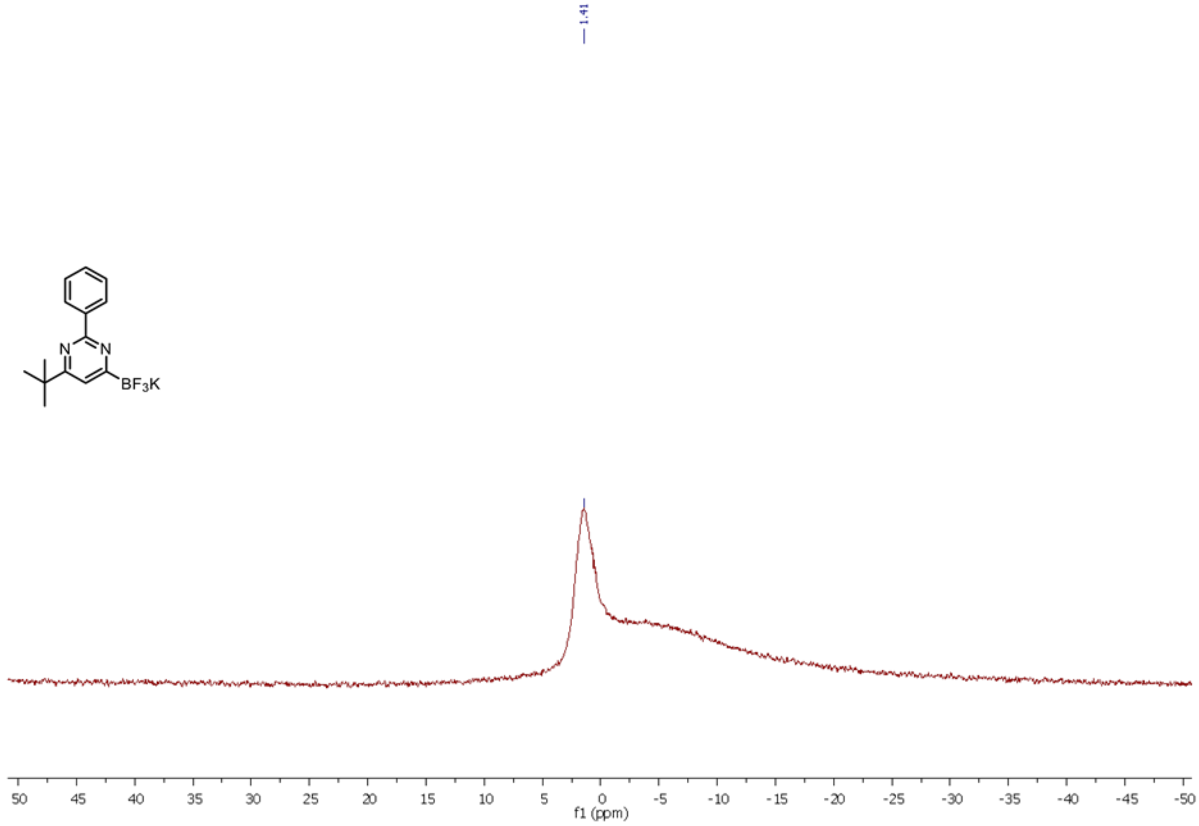
1.41
BF3K
N
N
50
45
40
35
30
25
20
15
10
5
0
-5
-10
-15
-20
-25
-30
-35
-40
-45
-50
f1 (ppm)

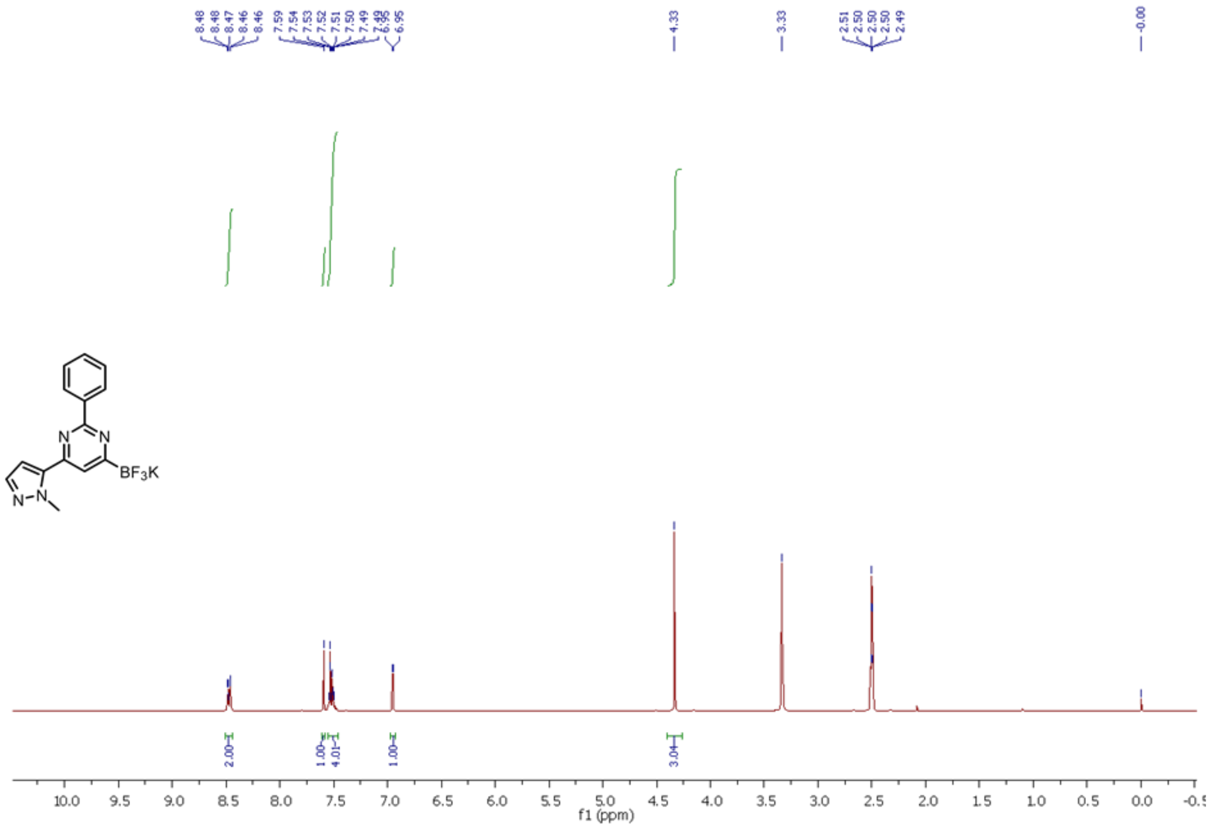
8.48
8.48
8.47
8.46
8.46
7.59
7.54
7.53
7.52
7.51
7.50
7.49
4.33
3.33
2.51
2.50
2.50
2.50
2.49
-0.00
BF3K
2.00
1.00
4.01
1.00
3.04
f1 (ppm)

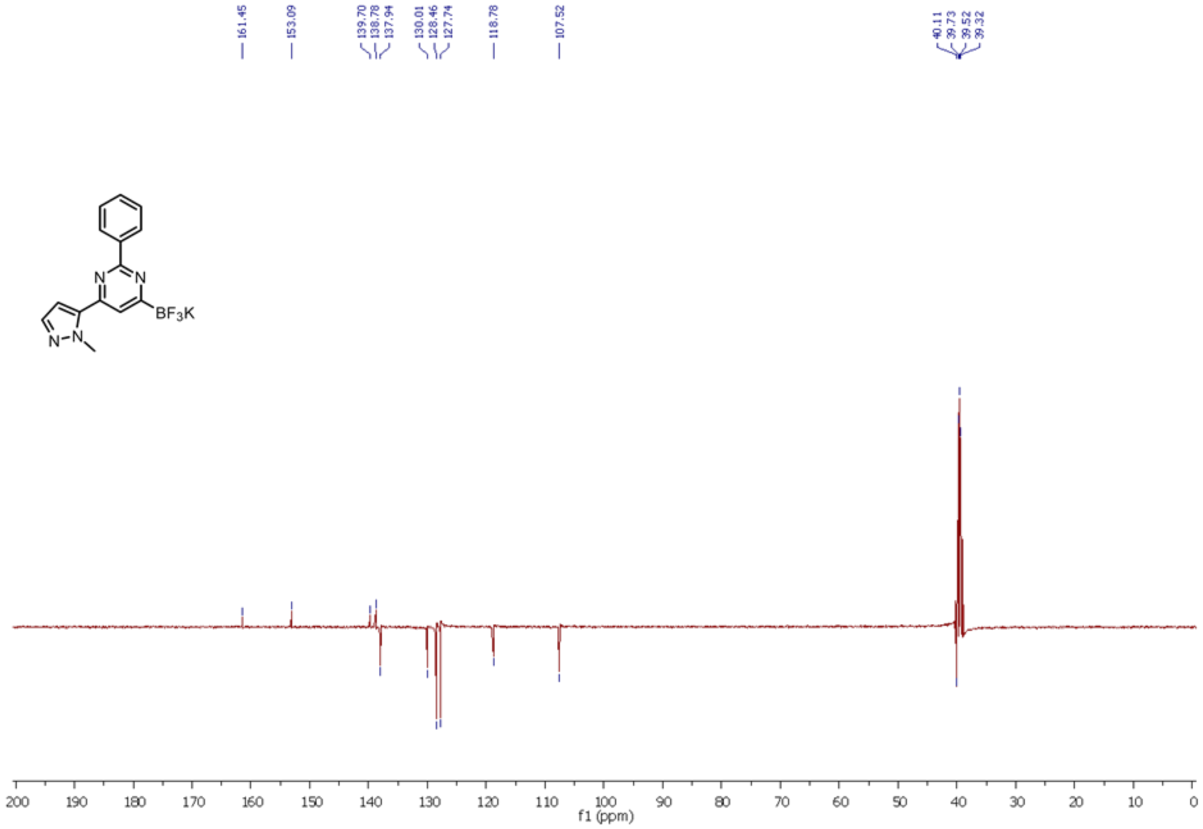
161.45
153.09
139.70
138.78
137.94
130.01
128.46
127.74
118.78
107.52
40.11
39.73
39.52
39.32
BF3K
f1 (ppm)

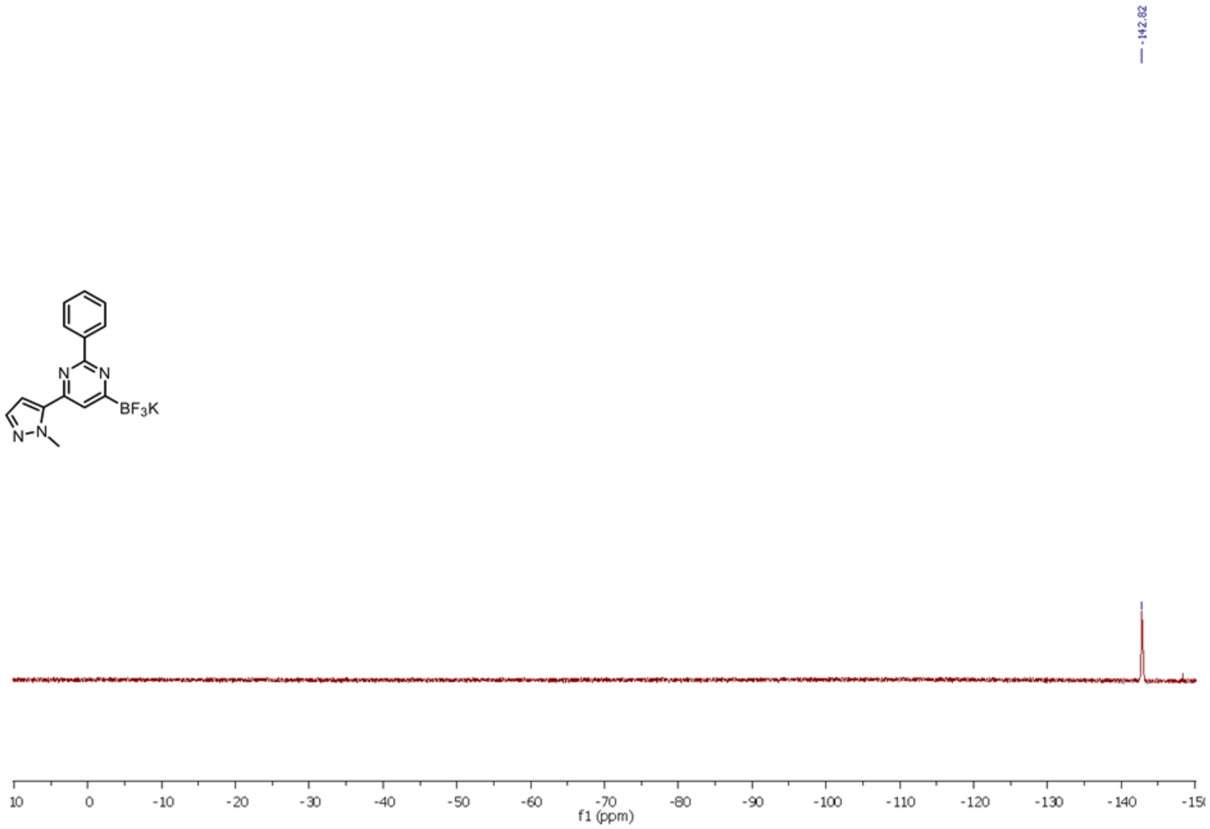
-142.82
BF3K
N
N
N
N
10
0
-10
-20
-30
-40
-50
-60
-70
-80
-90
-100
-110
-120
-130
-140
f1 (ppm)

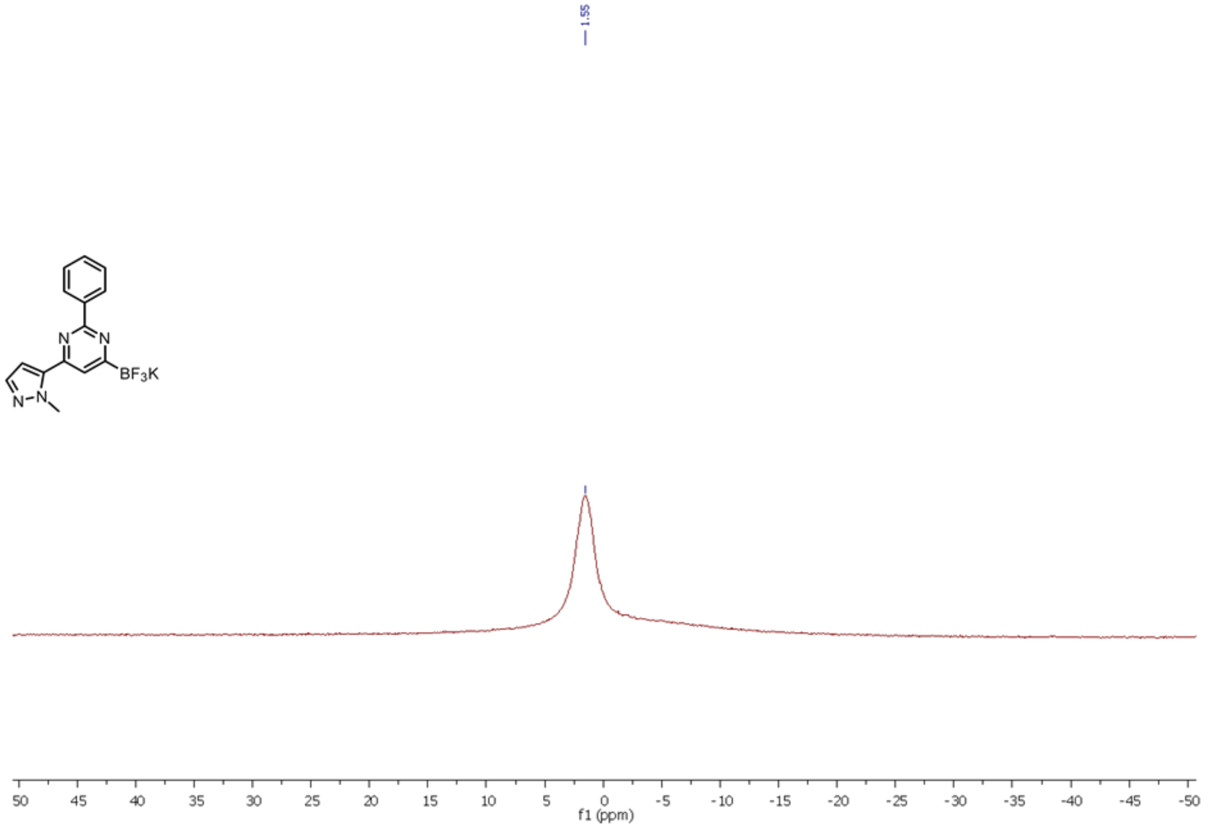
1.55
BF3K
N
N
N
N
50
45
40
35
30
25
20
15
10
5
0
-5
-10
-15
-20
-25
-30
-35
-40
-45
-50
f1 (ppm)

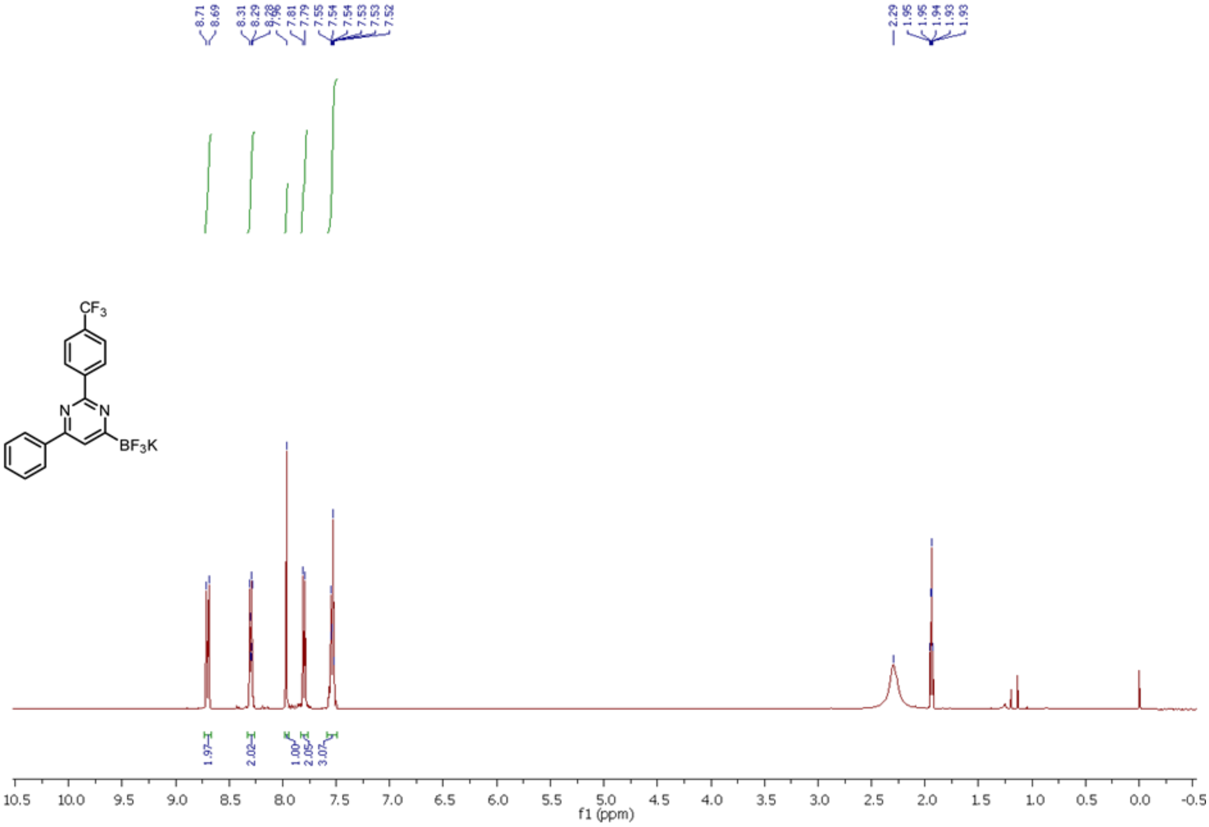

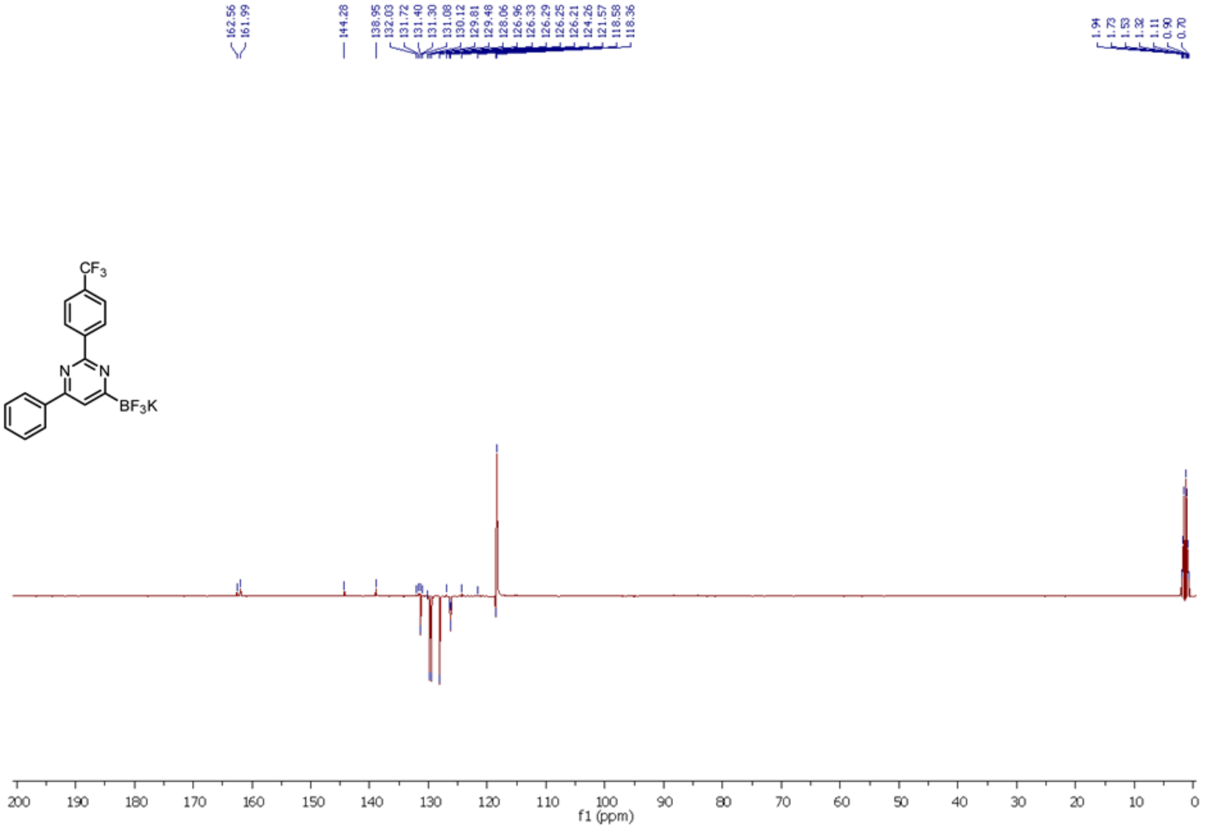

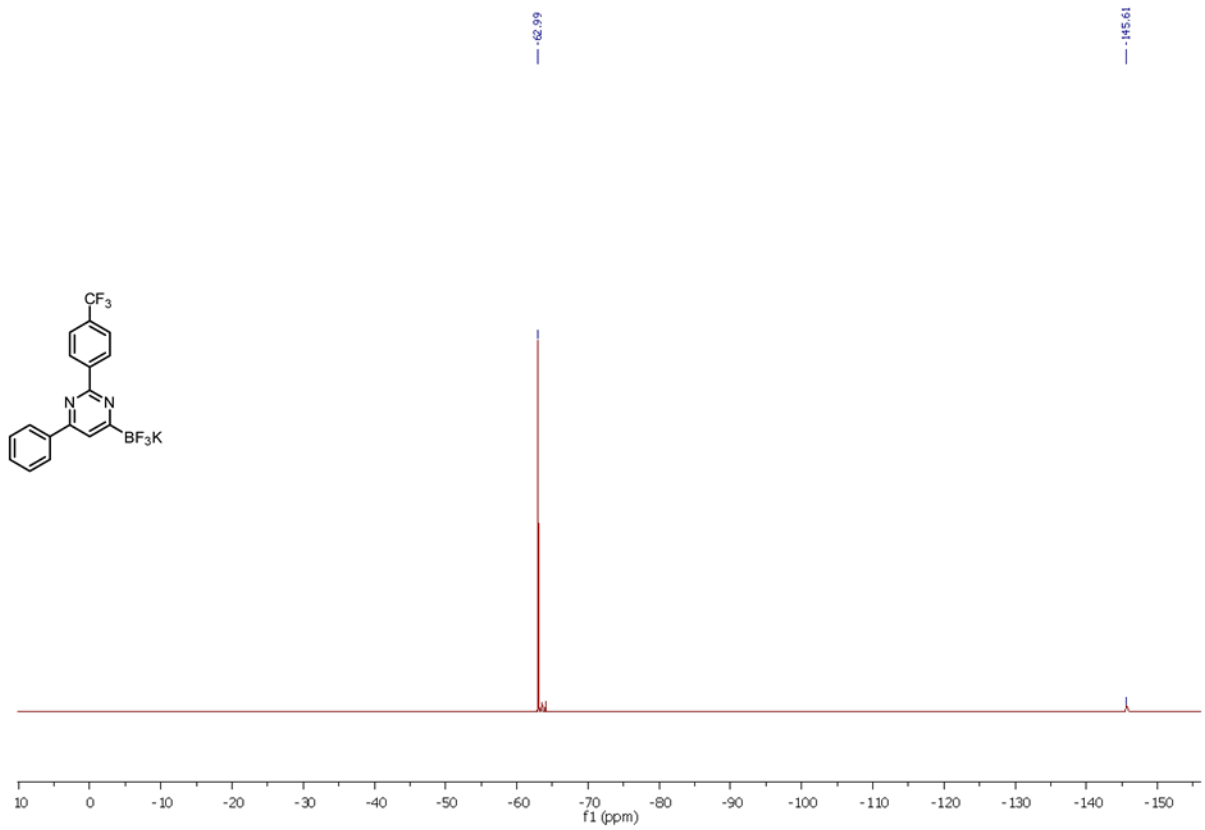
-62.99
-145.61
CF3
N
N
BF3K
10
0
-10
-20
-30
-40
-50
-60
-70
-80
-90
-100
-110
-120
-130
-140
-150
f1 (ppm)

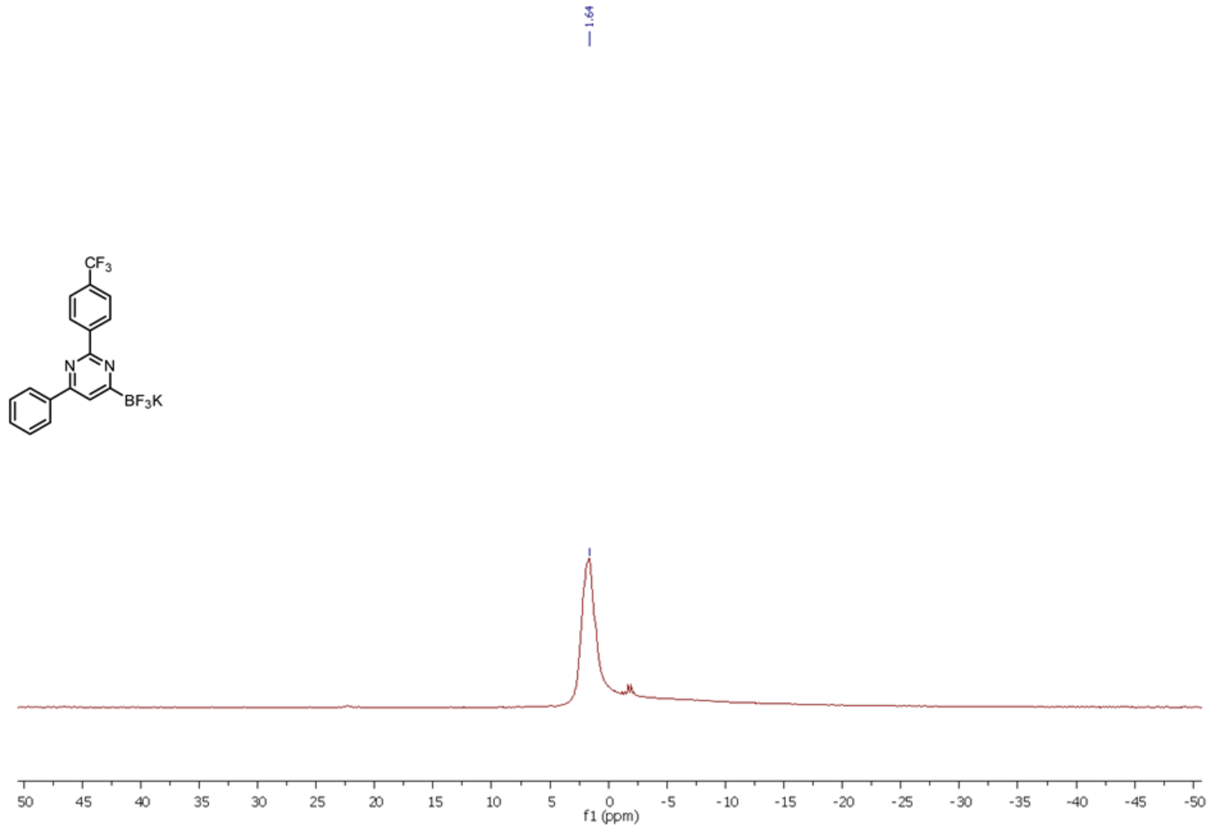
1.64
CF3
N
N
BF3K
50
45
40
35
30
25
20
15
10
5
0
-5
-10
-15
-20
-25
-30
-35
-40
-45
-50
f1 (ppm)

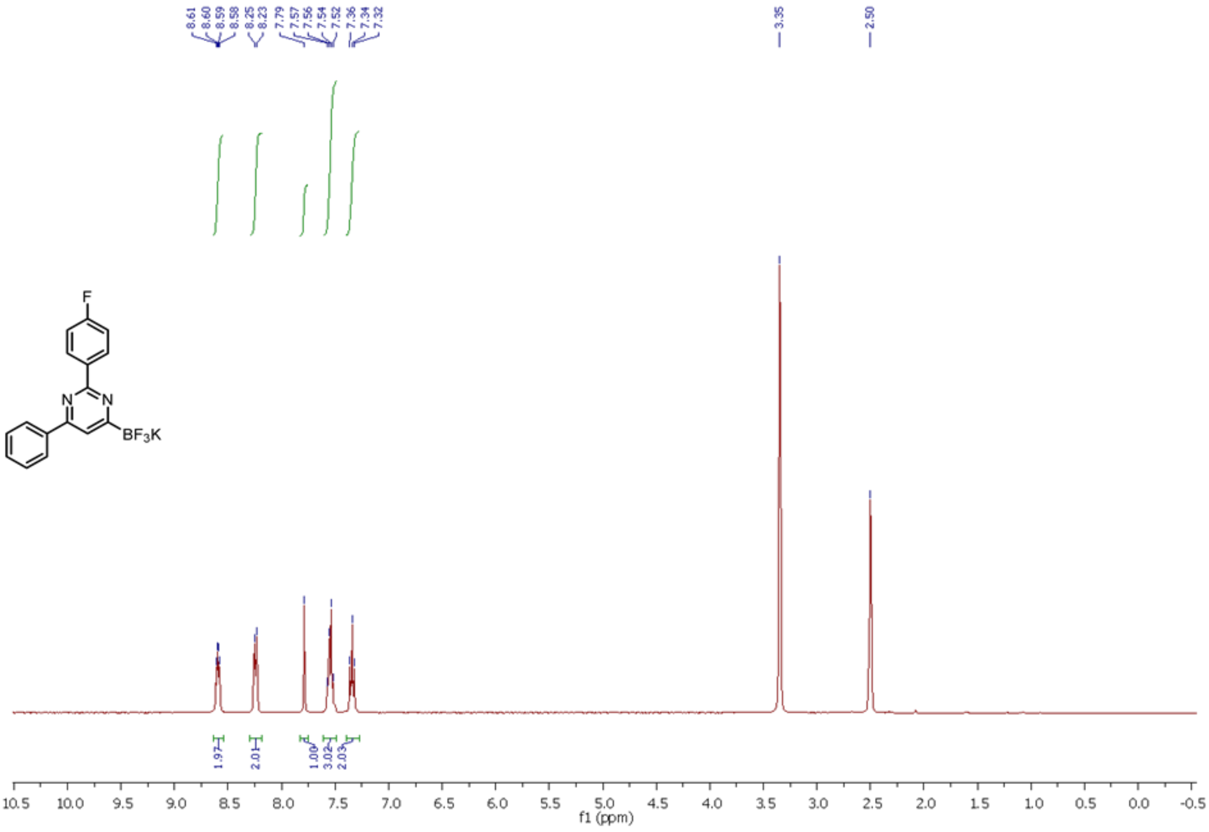

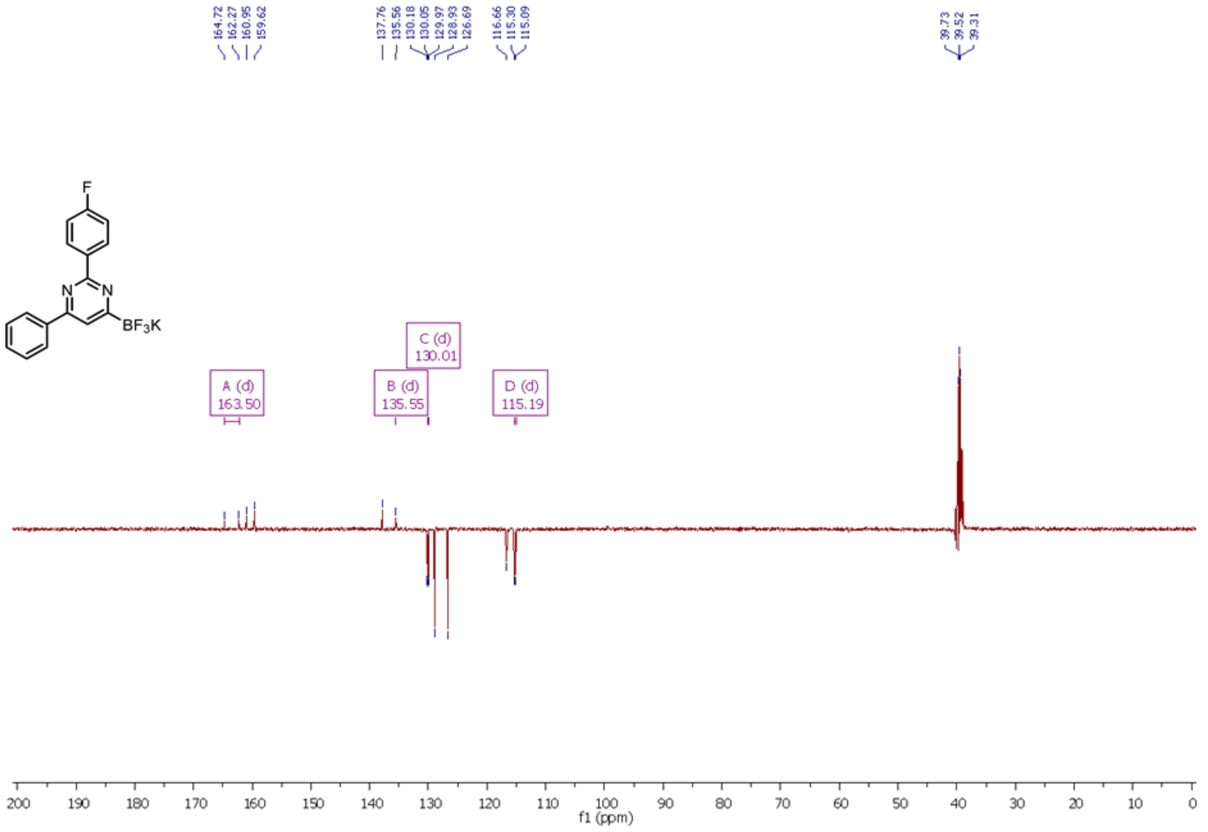

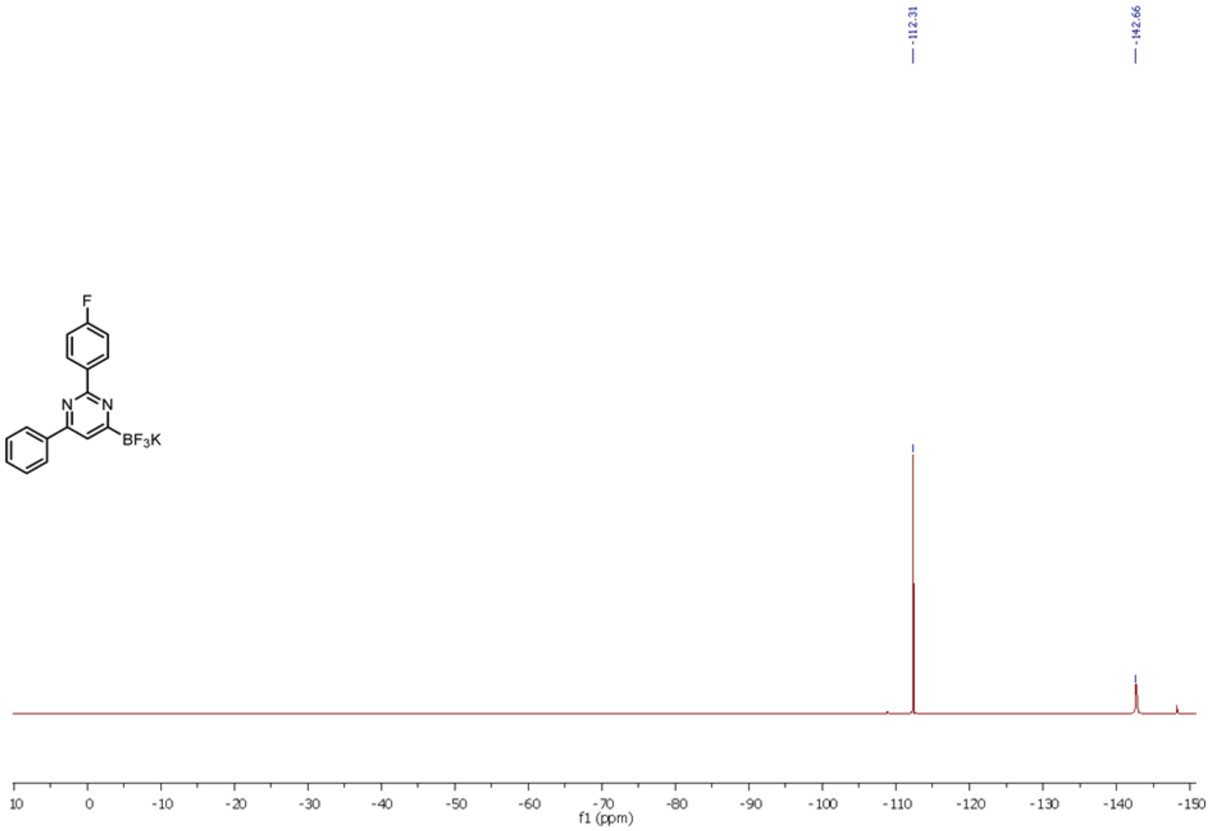

-112.31
-142.66
F
N
N
BF3K
10 0 -10 -20 -30 -40 -50 -60 -70 -80 -90 -100 -110 -120 -130 -140 -150
f1 (ppm)

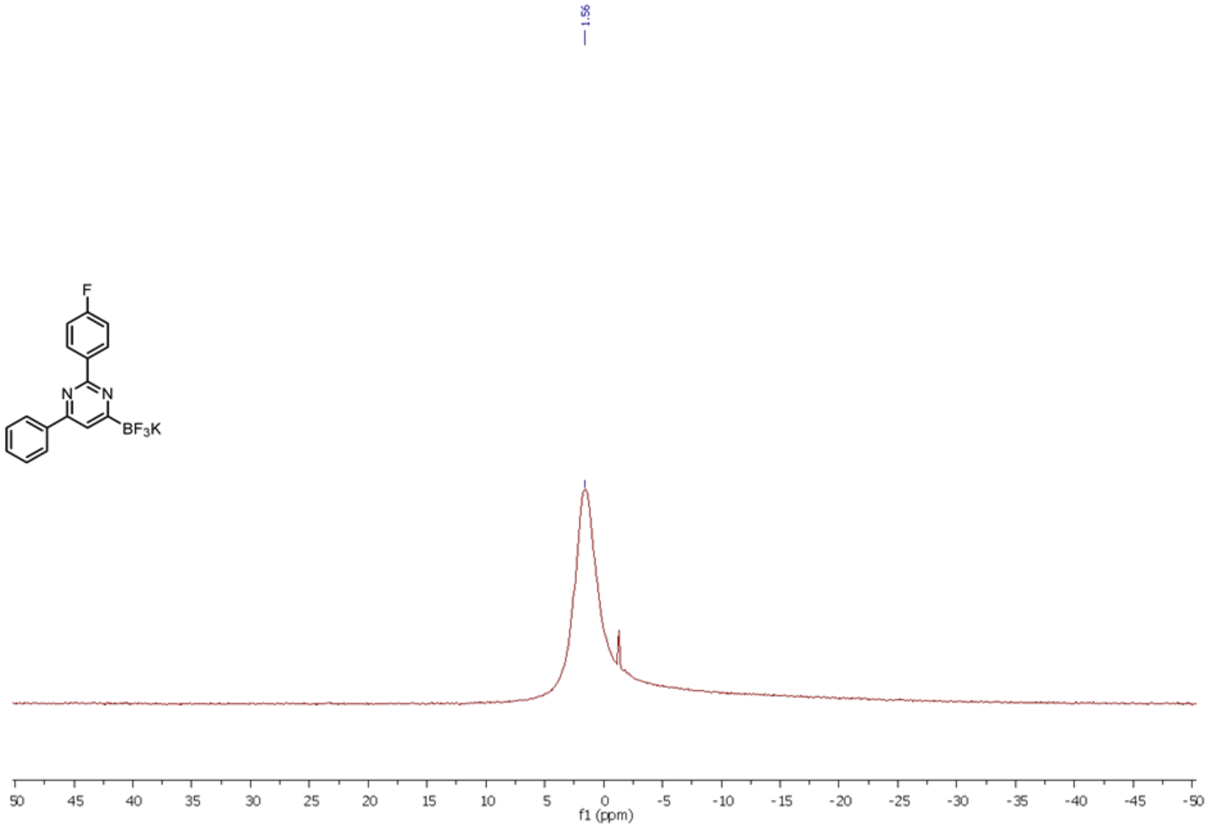

1.56
F
N
N
BF3K
50 45 40 35 30 25 20 15 10 5 0 -5 -10 -15 -20 -25 -30 -35 -40 -45 -50
f1 (ppm)

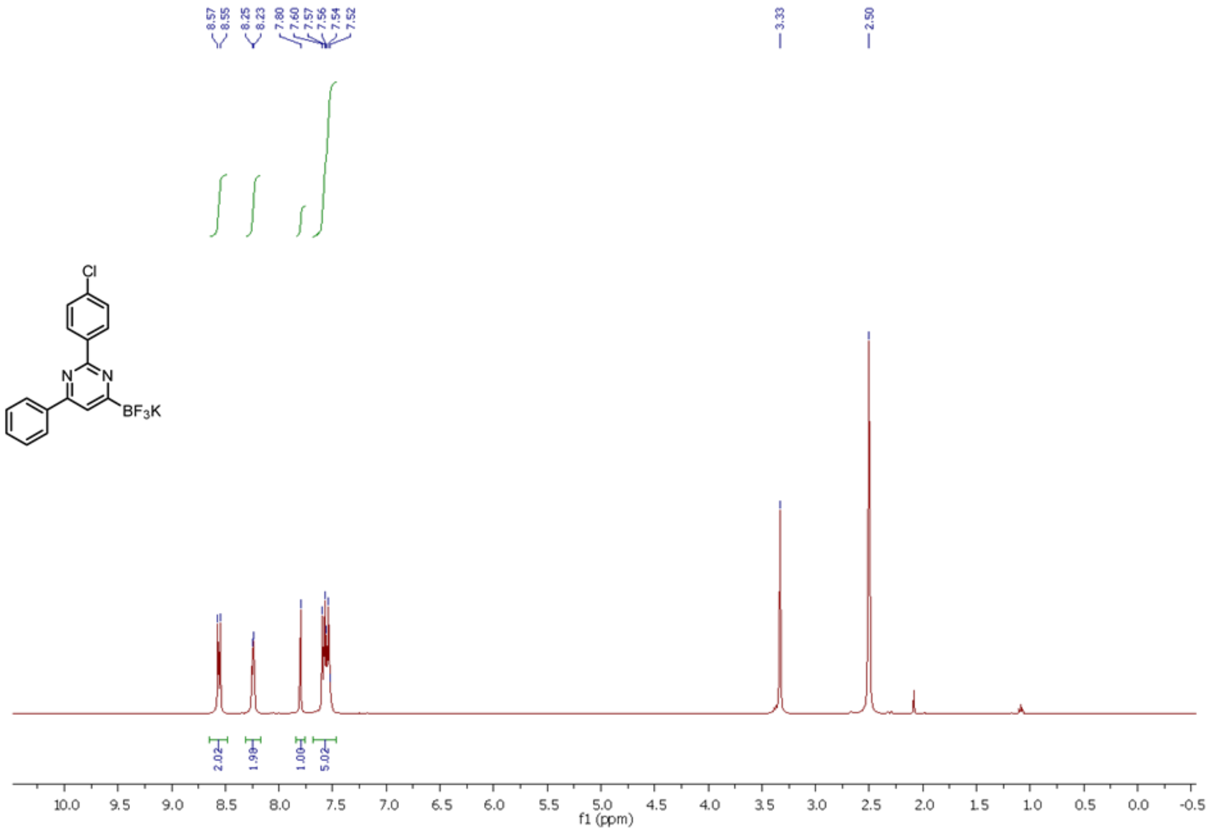
Cl
N
N
BF3K
8.57
8.55
8.25
8.23
7.80
7.60
7.57
7.56
7.54
7.52
3.33
2.50
2.02
1.98
1.00
5.02
f1 (ppm)

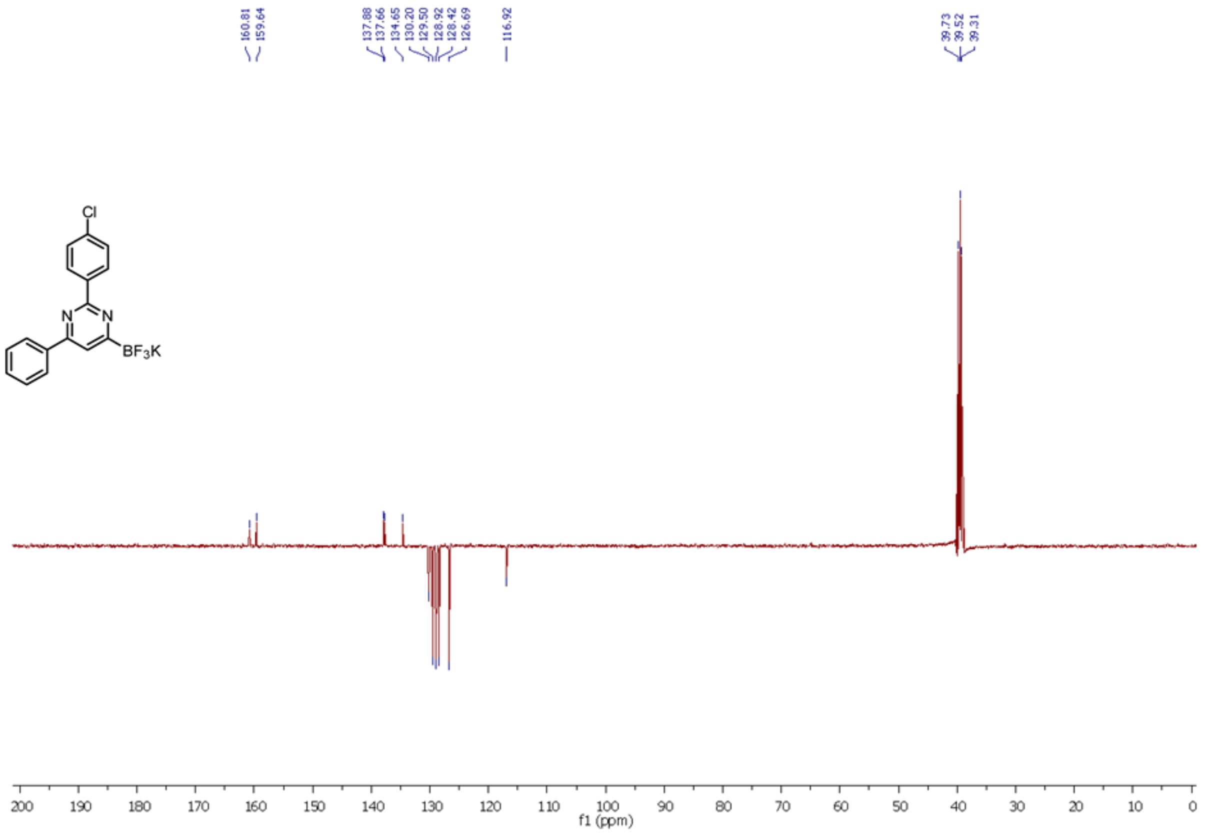
Cl
N
N
BF3K
160.81
159.64
137.88
137.66
134.65
130.20
129.50
128.92
128.42
126.69
116.92
39.73
39.52
39.31
f1 (ppm)

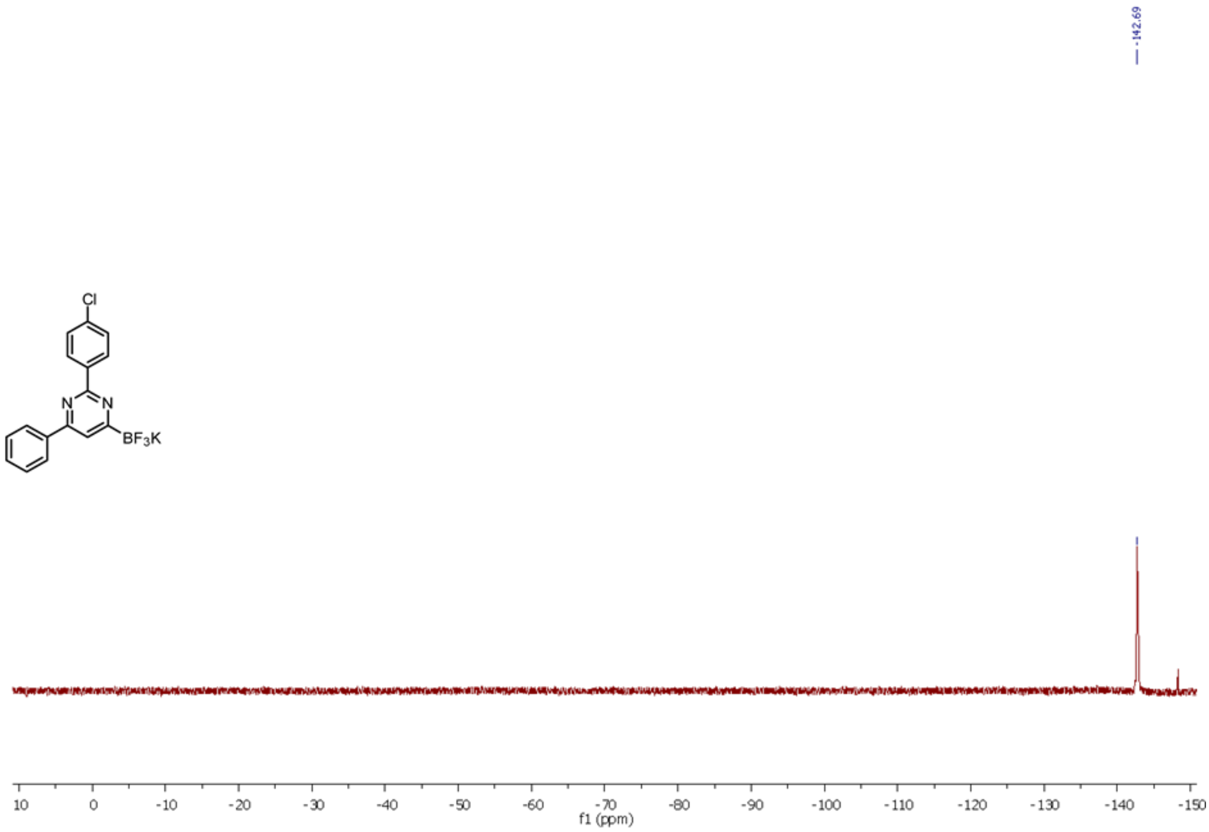
-142.69
Cl
N
N
BF3K
10 0 -10 -20 -30 -40 -50 -60 -70 -80 -90 -100 -110 -120 -130 -140 -150
f1 (ppm)

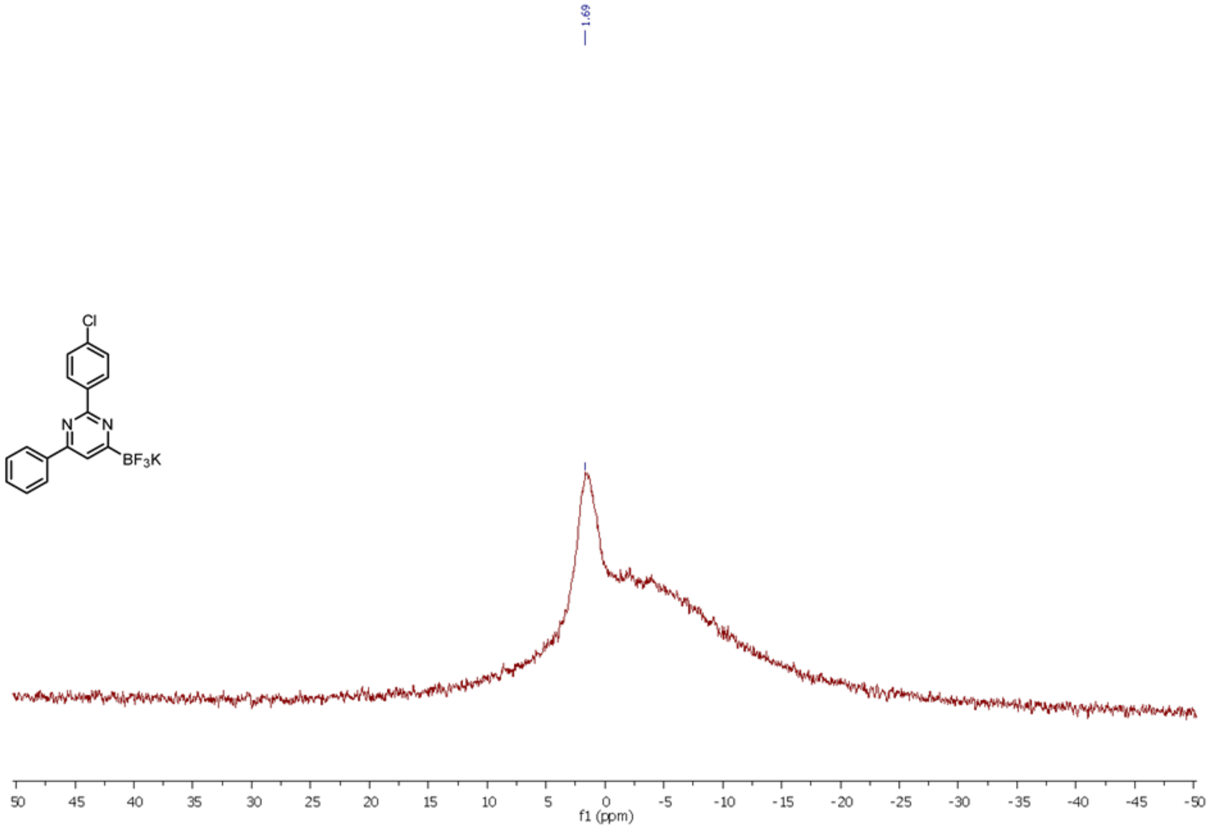
1.69
Cl
N
N
BF3K
50 45 40 35 30 25 20 15 10 5 0 -5 -10 -15 -20 -25 -30 -35 -40 -45 -50
f1 (ppm)

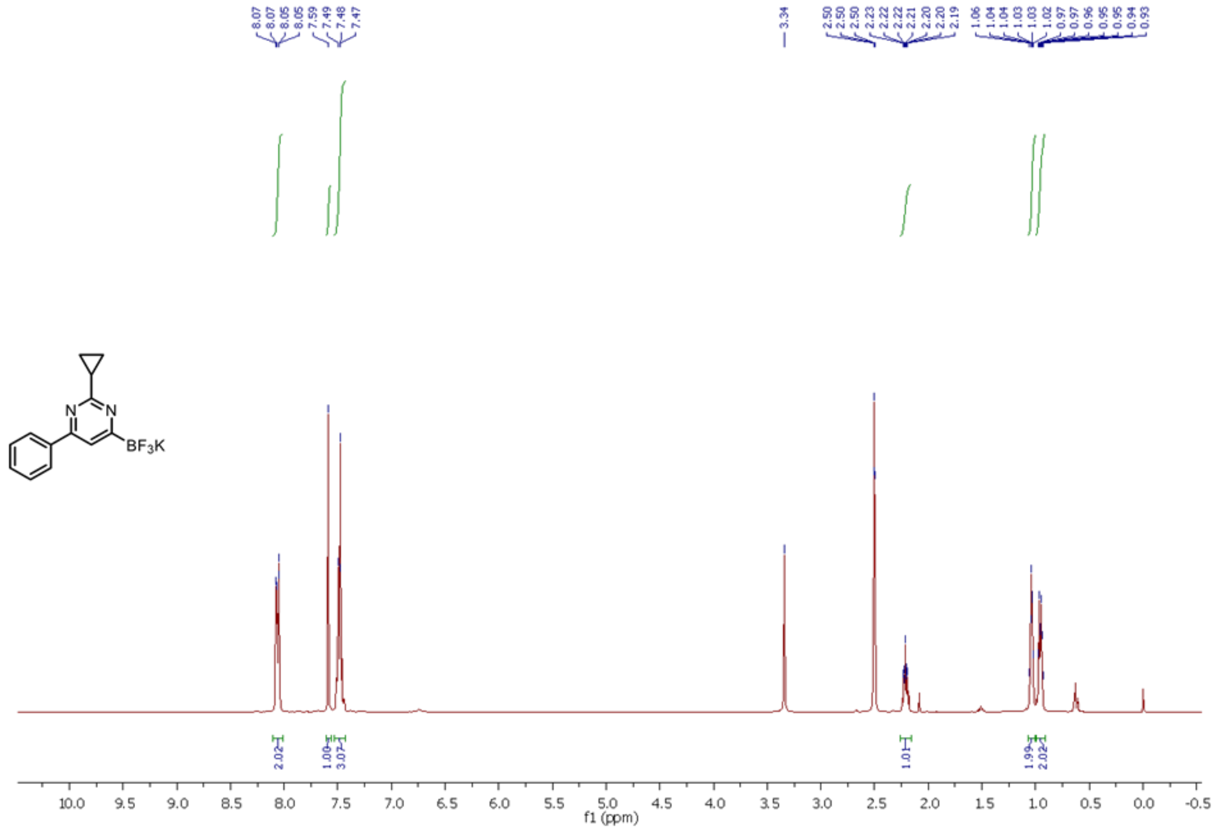

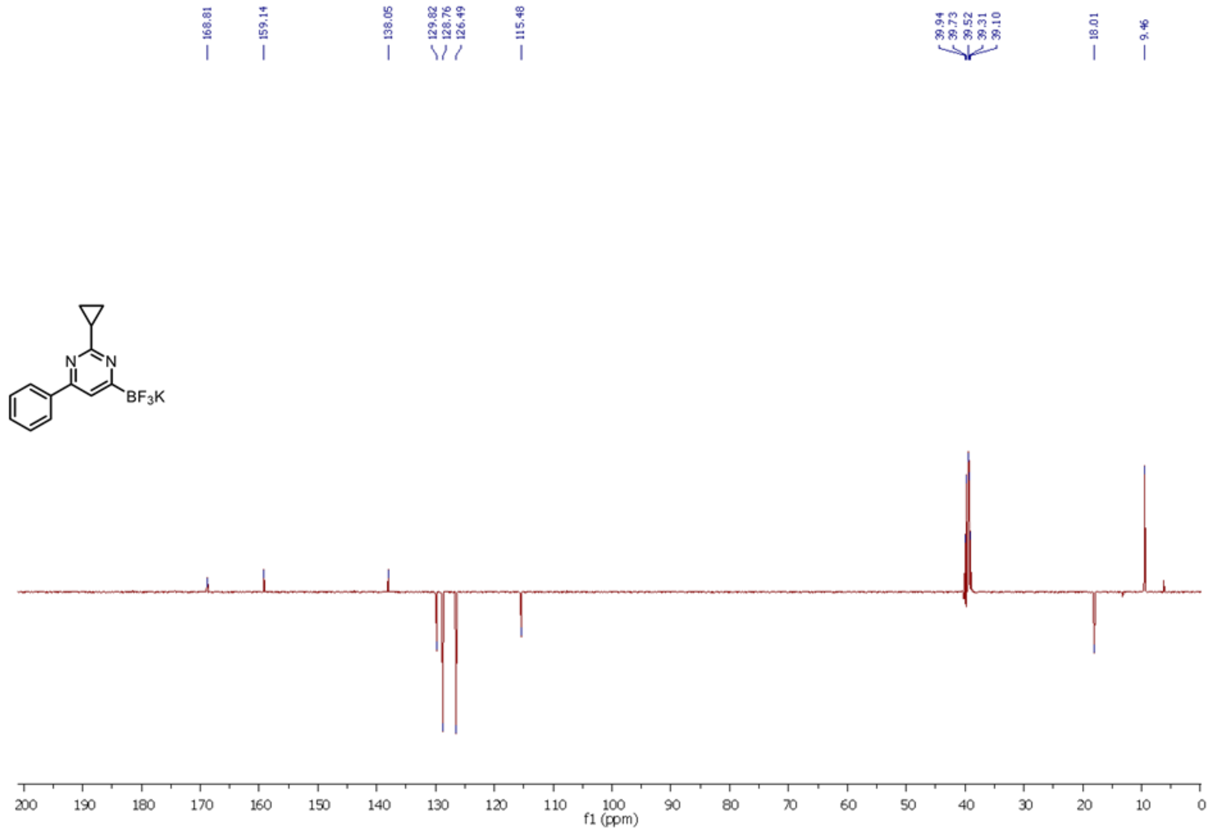

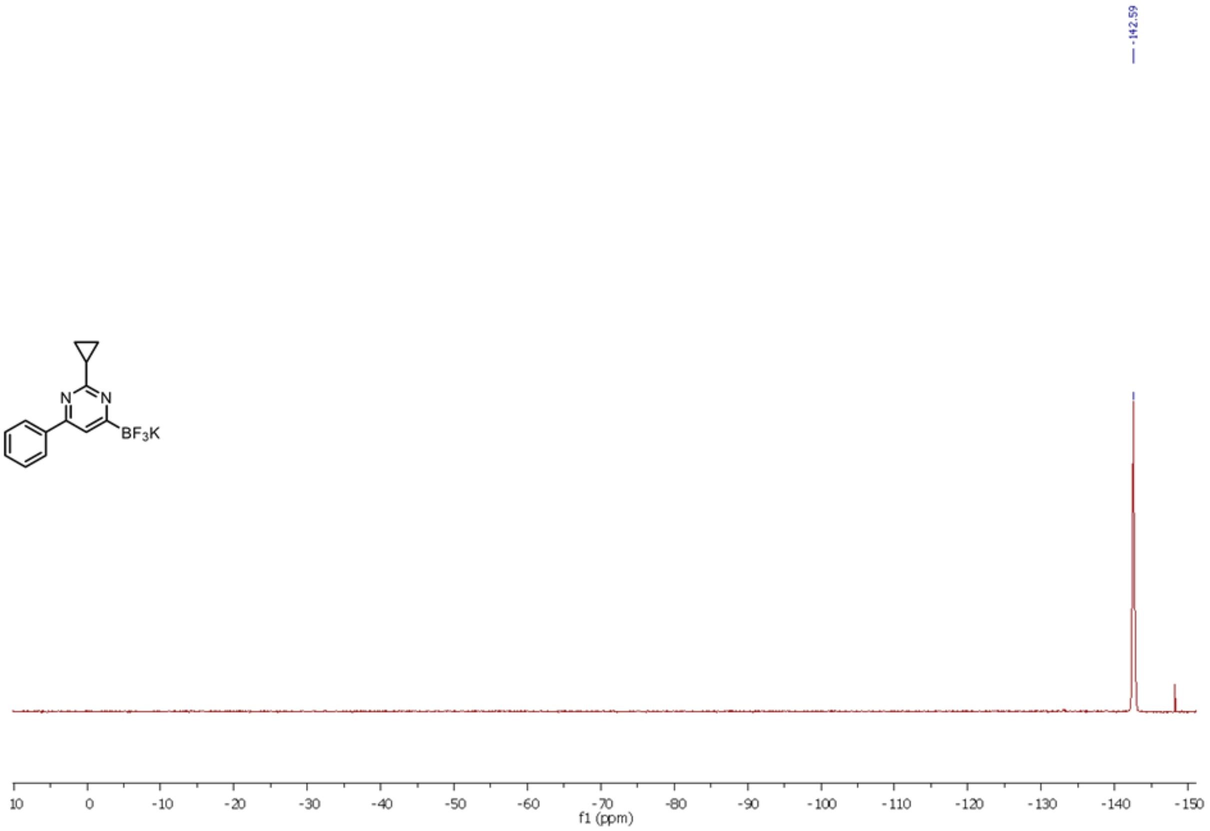
-142.59
N
N
BF3K
10 0 -10 -20 -30 -40 -50 -60 -70 -80 -90 -100 -110 -120 -130 -140 -150
f1 (ppm)

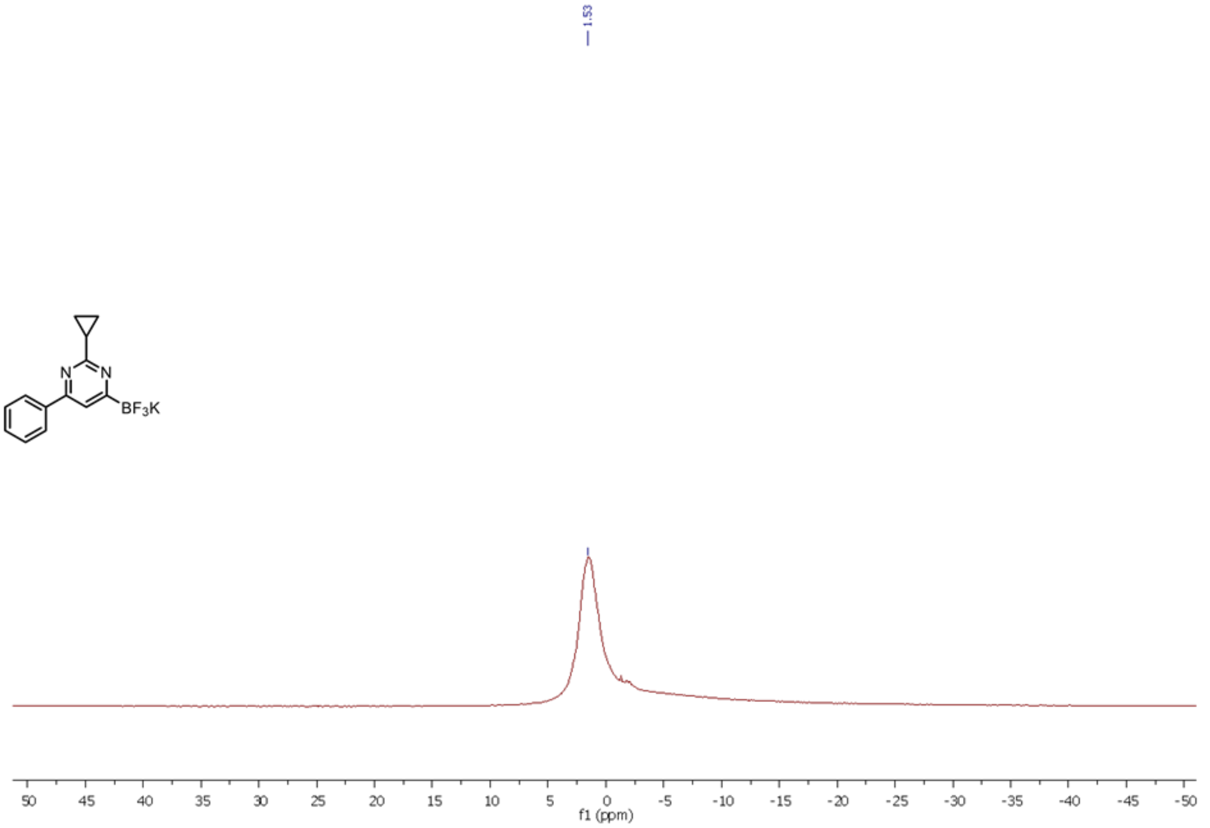
1.53
N
N
BF3K
50 45 40 35 30 25 20 15 10 5 0 -5 -10 -15 -20 -25 -30 -35 -40 -45 -50
f1 (ppm)

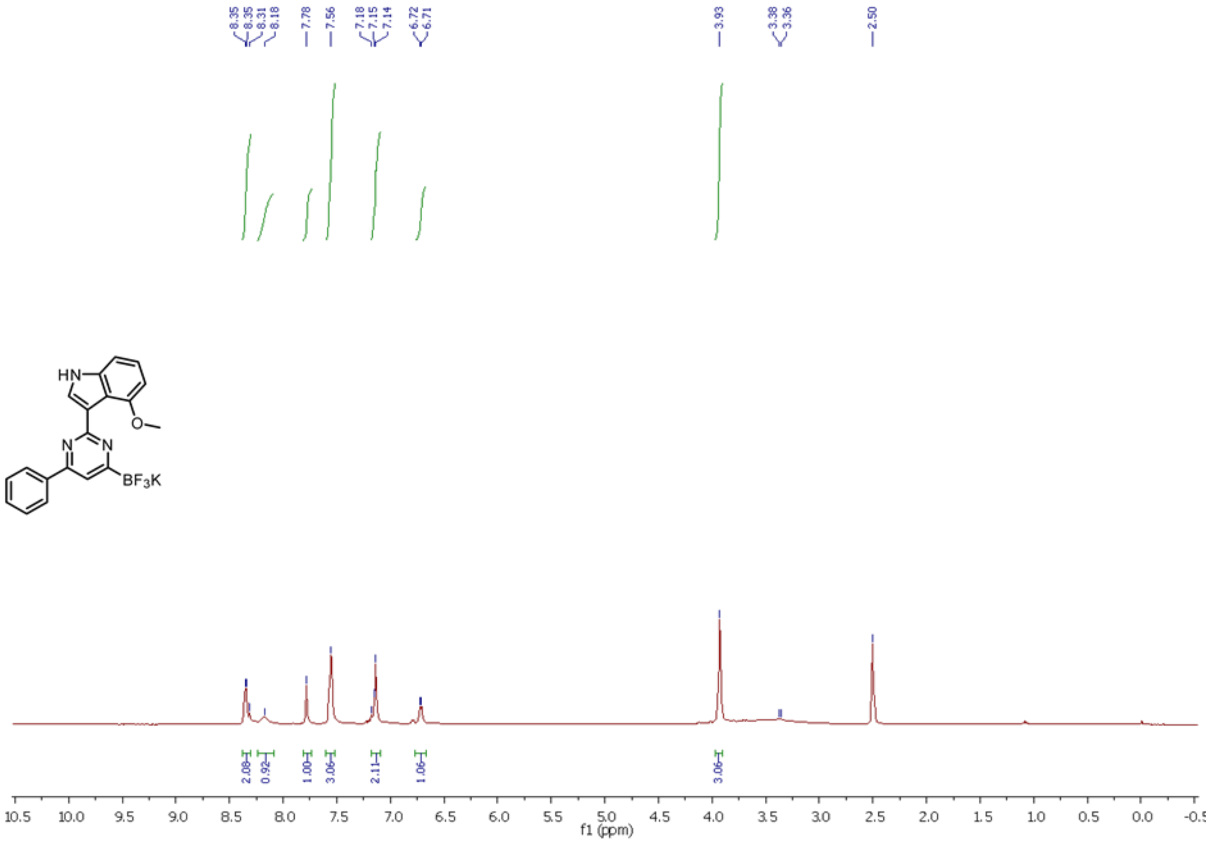

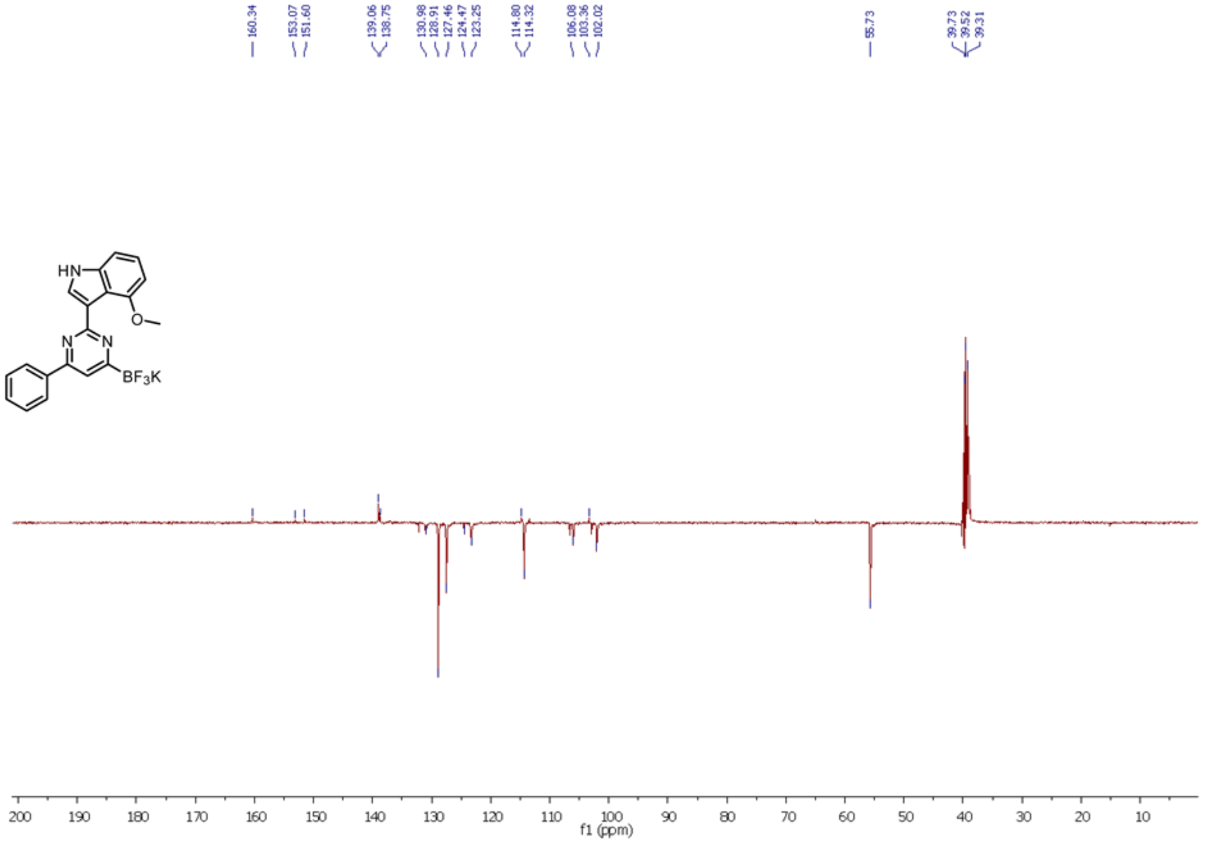

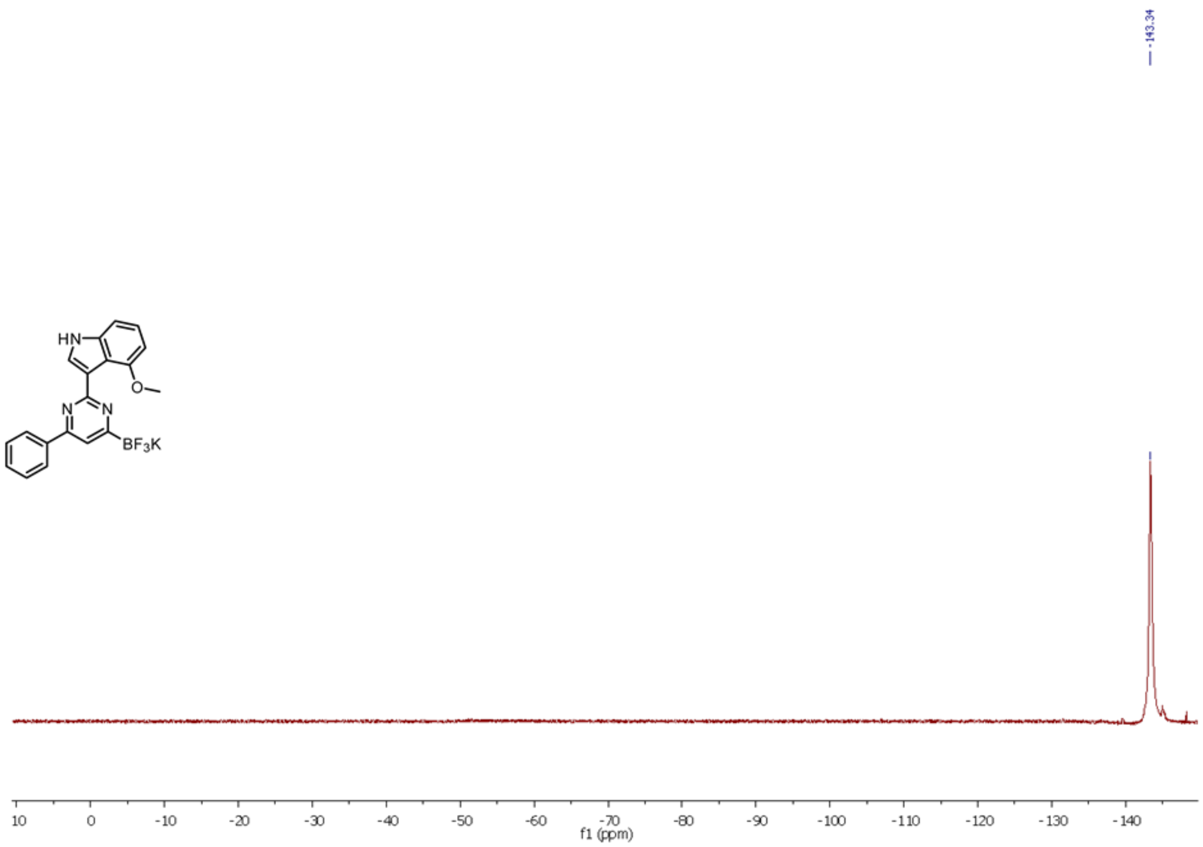
-143.34
HN
O
N
N
BF3K
10 0 -10 -20 -30 -40 -50 -60 -70 -80 -90 -100 -110 -120 -130 -140
f1 (ppm)

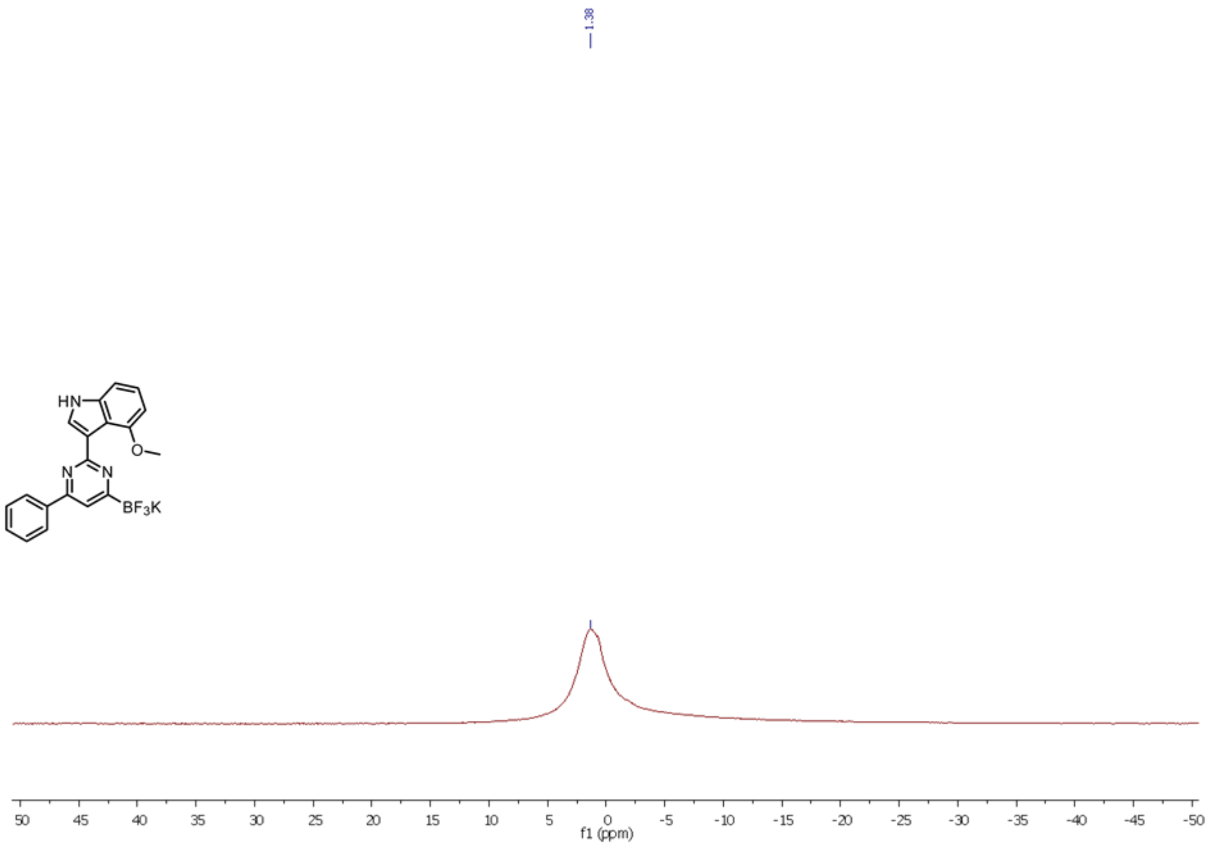
1.38
HN
O
N
N
BF3K
50 45 40 35 30 25 20 15 10 5 0 -5 -10 -15 -20 -25 -30 -35 -40 -45 -50
f1 (ppm)

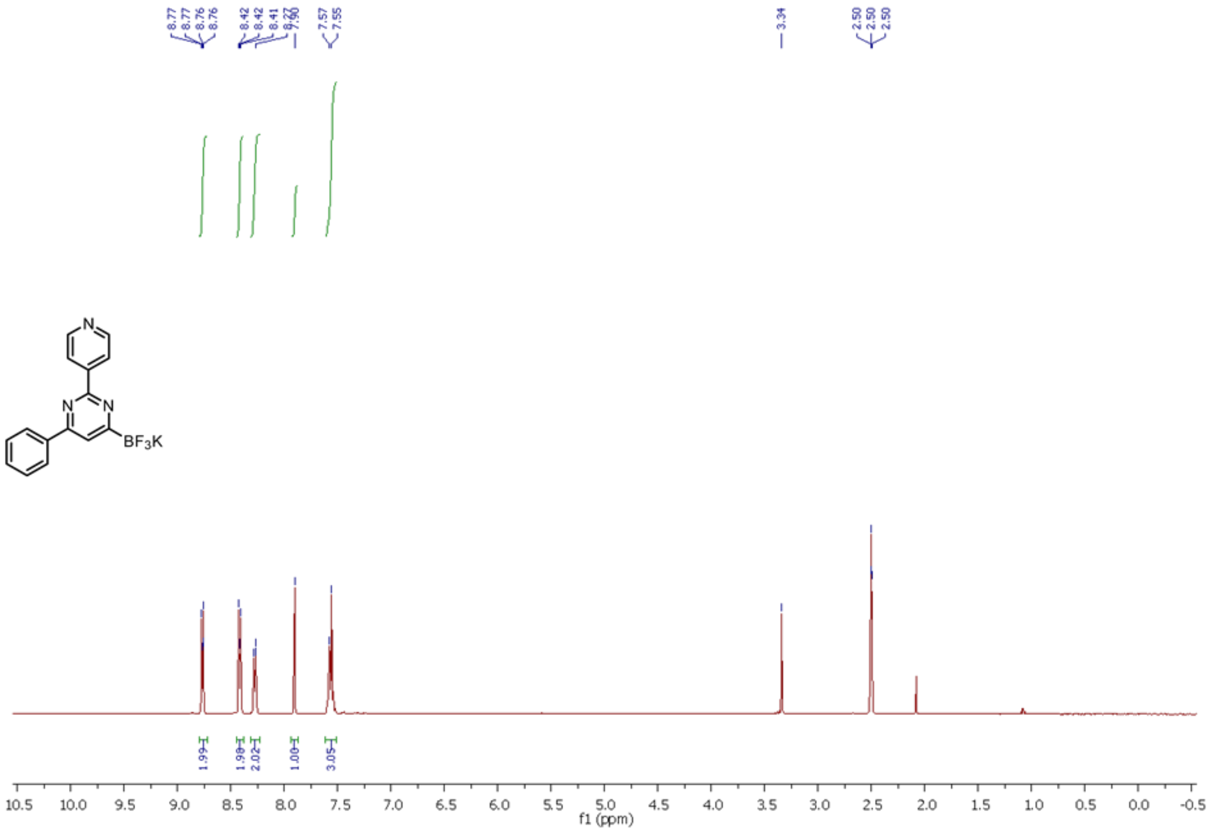
8.77
8.77
8.76
8.76
8.42
8.42
8.41
8.27
7.90
7.57
7.55
3.34
2.50
2.50
2.50
BF3K
1.99
1.98
2.02
1.00
3.05
f1 (ppm)

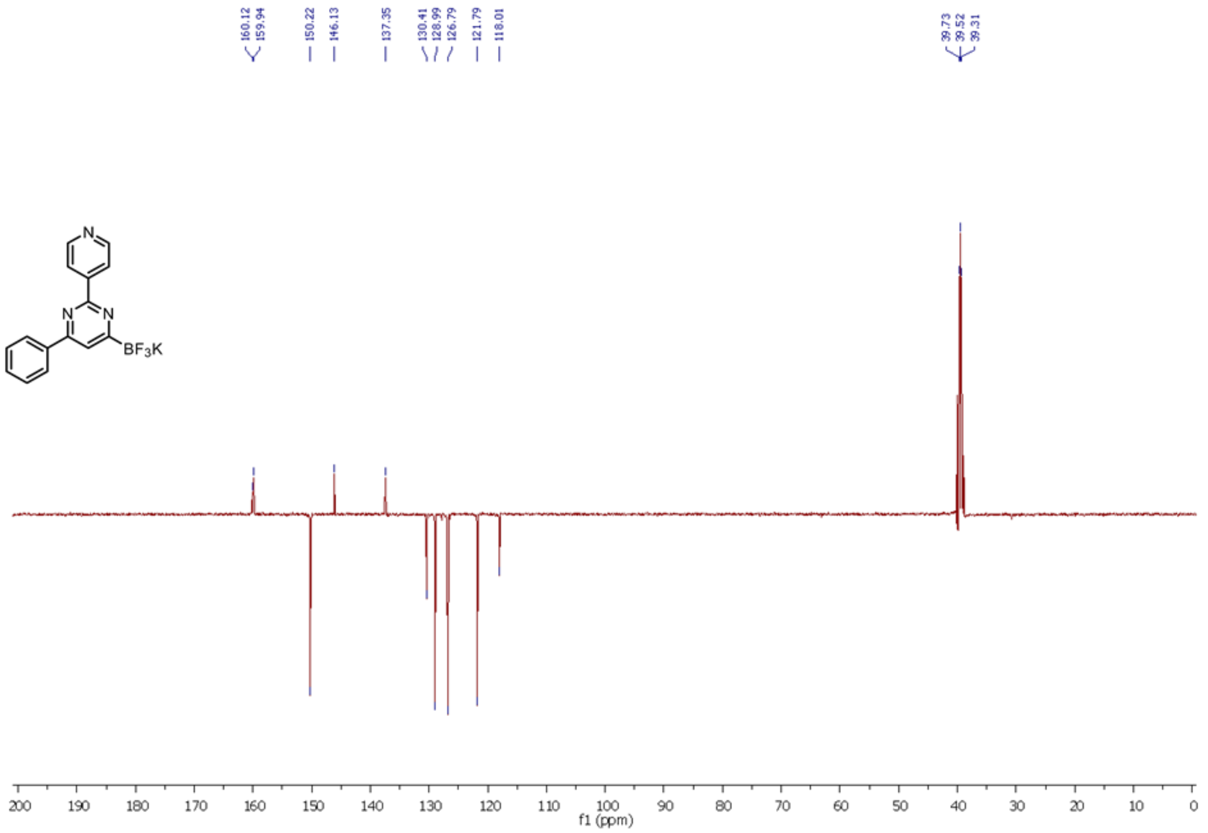
160.12
159.94
150.22
146.13
137.35
130.41
128.99
126.79
121.79
118.01
39.73
39.52
39.31
BF3K
f1 (ppm)

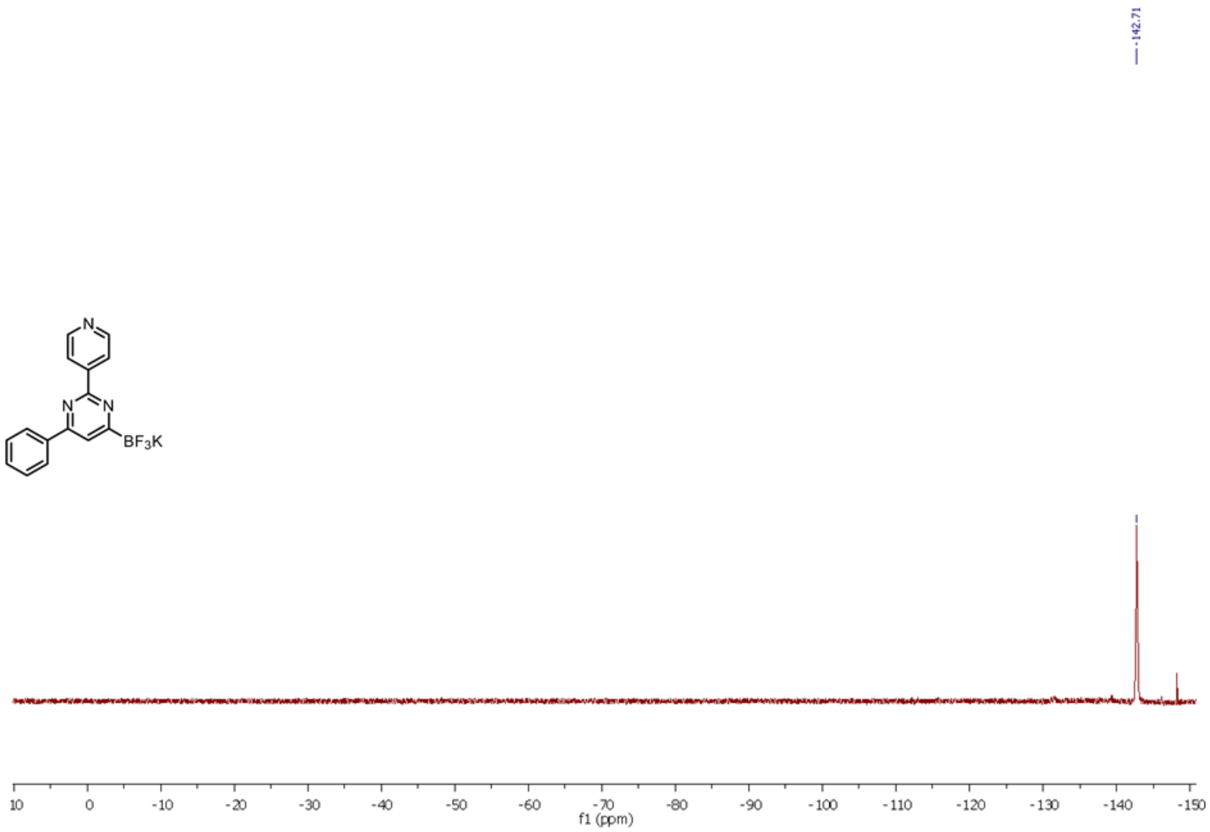
-142.71
BF3K
f1 (ppm)

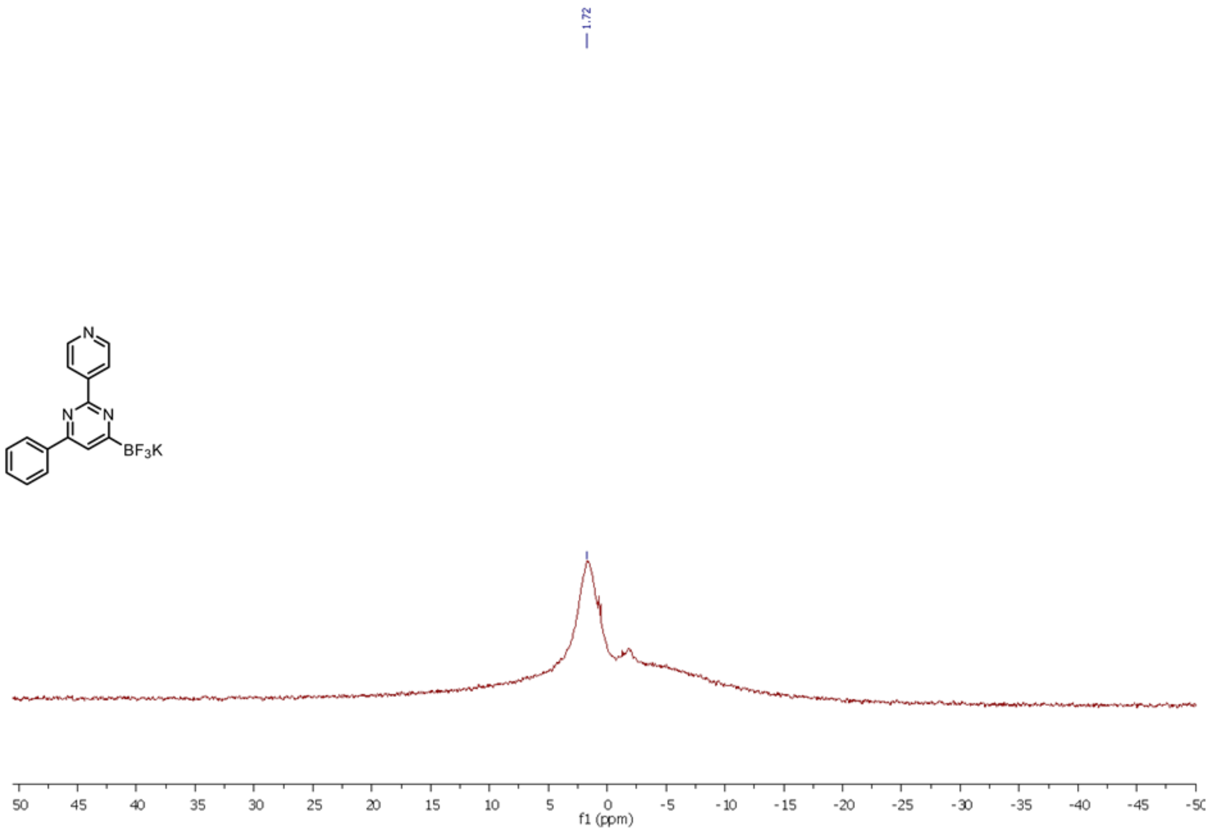
1.72
BF3K
f1 (ppm)

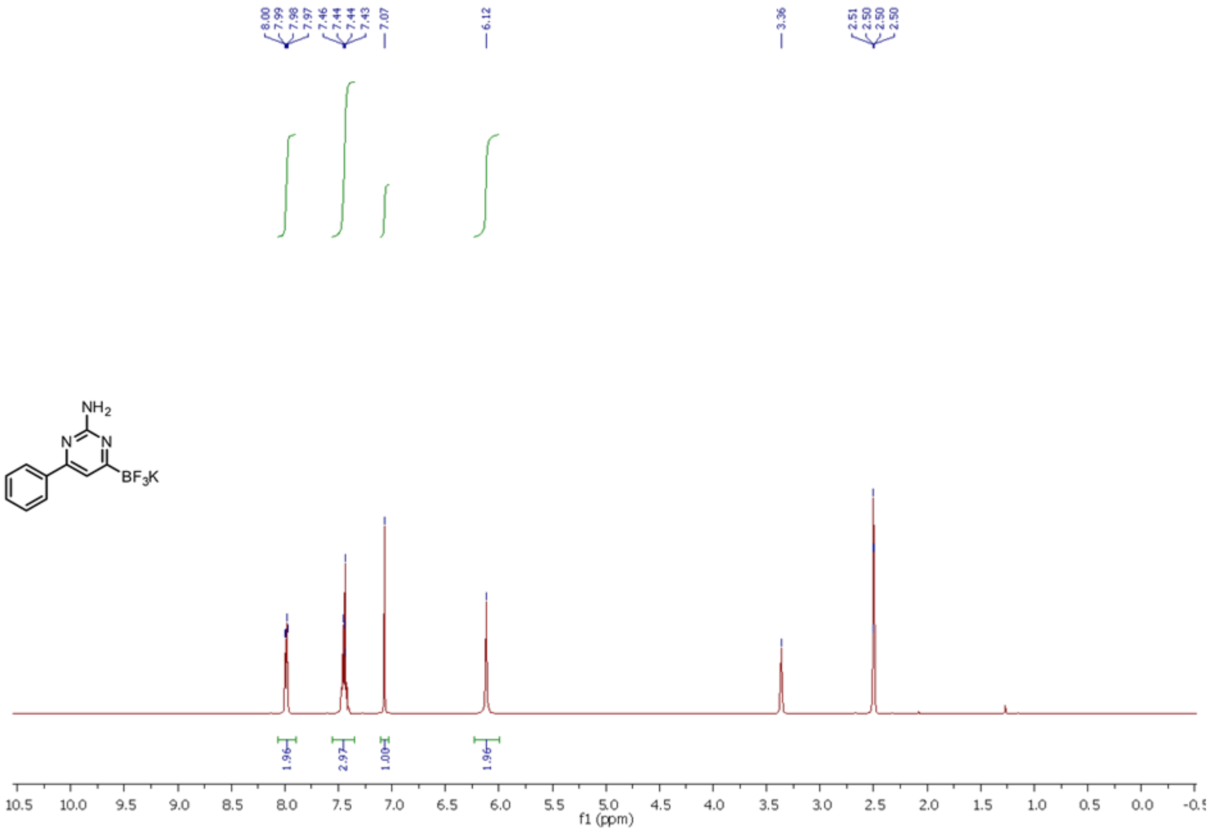
8.00
7.99
7.98
7.97
7.46
7.44
7.44
7.43
7.07
6.12
3.36
2.51
2.50
2.50
2.50
NH2
N
N
BF3K
1.96
2.97
1.00
1.96
f1 (ppm)

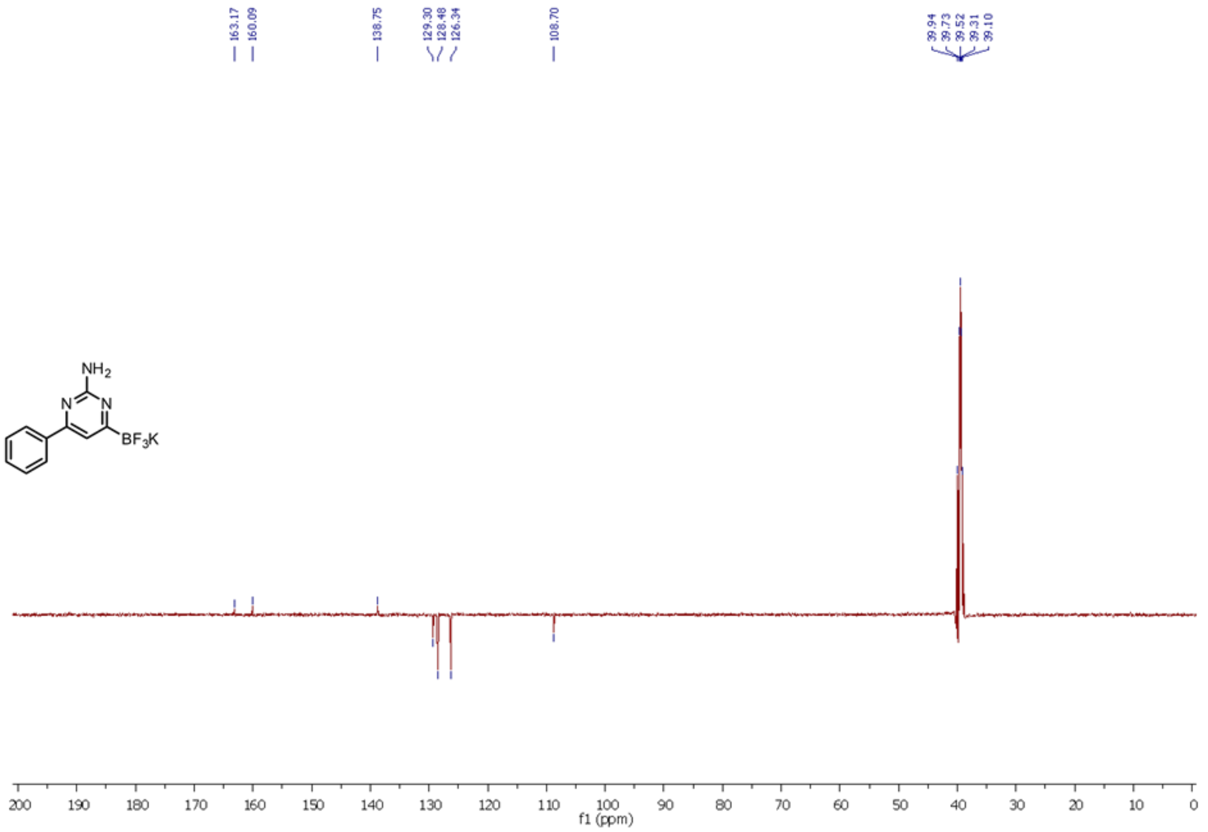
163.17
160.09
138.75
129.30
128.48
126.34
108.70
39.94
39.73
39.52
39.31
39.10
NH2
N
N
BF3K
f1 (ppm)

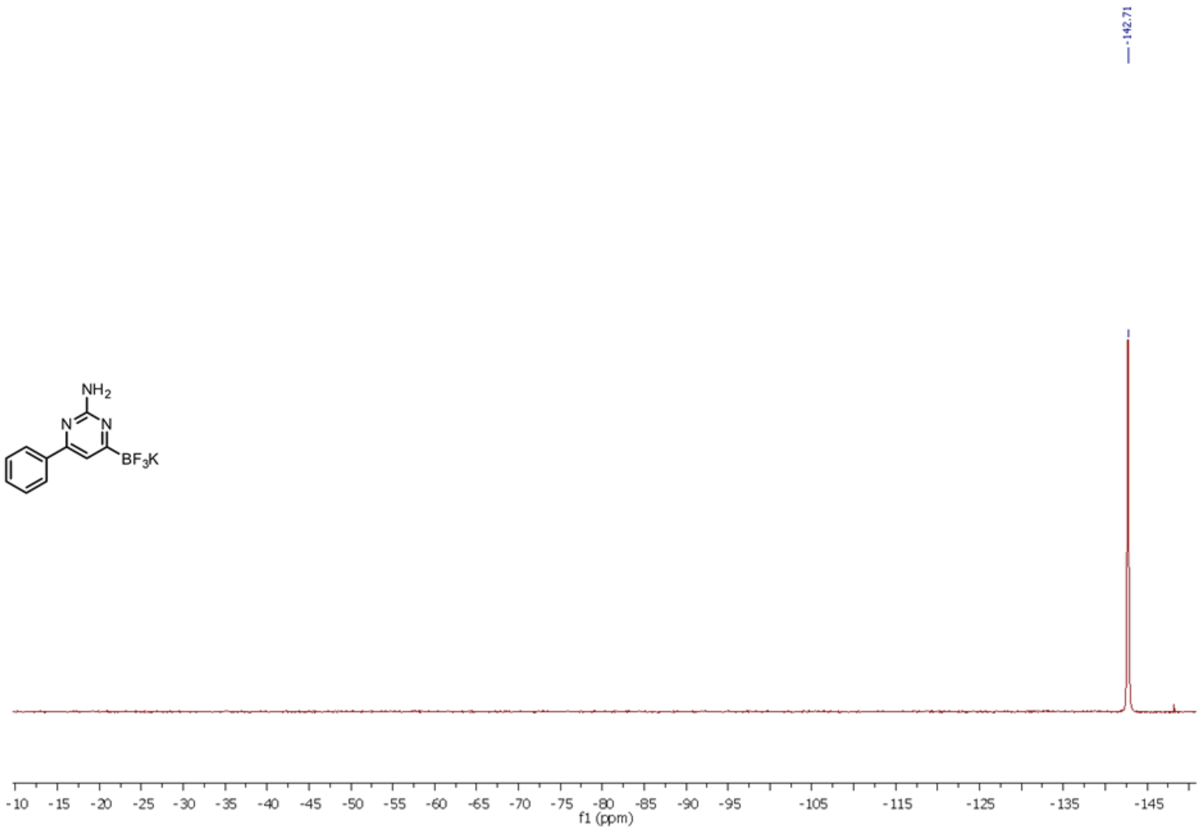
-142.71
NH2
N
N
BF3K
-10
-15
-20
-25
-30
-35
-40
-45
-50
-55
-60
-65
-70
-75
-80
-85
-90
-95
-105
-115
-125
-135
-145
f1 (ppm)

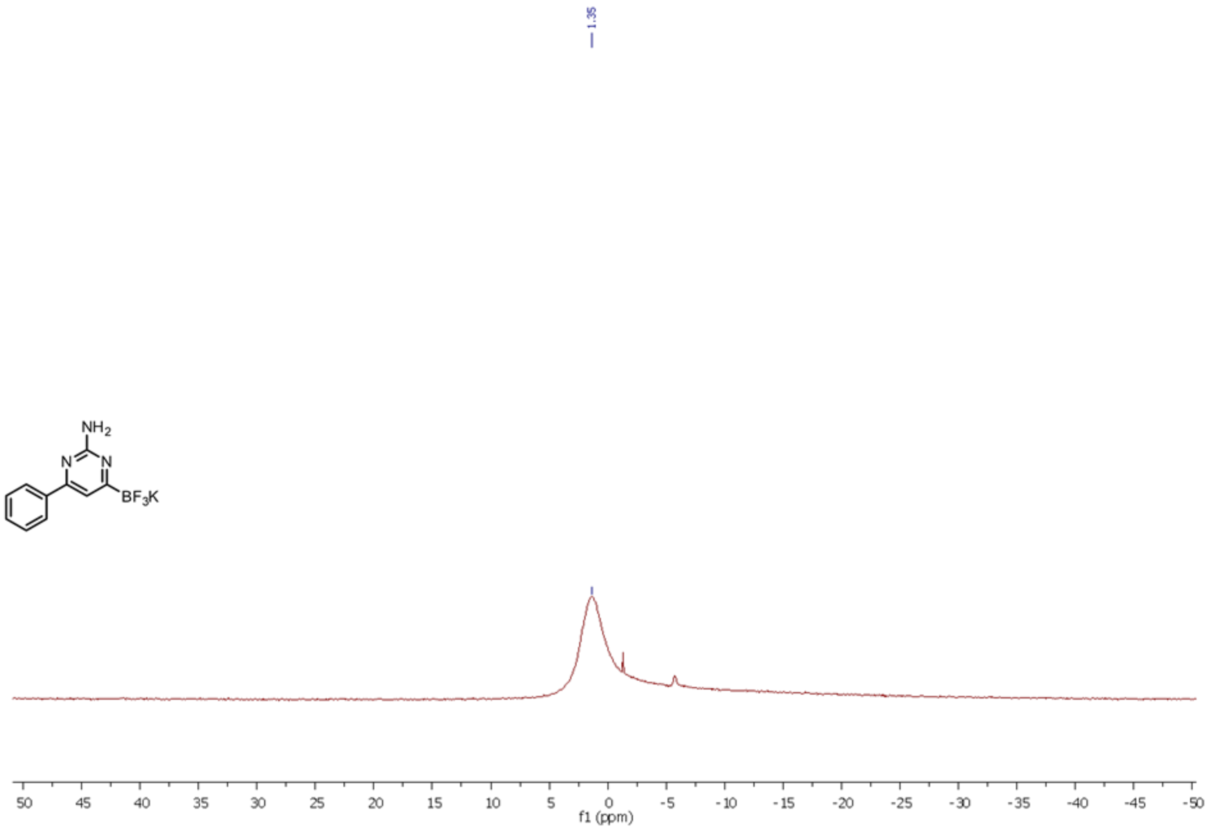
1.35
NH2
N
N
BF3K
50
45
40
35
30
25
20
15
10
5
0
-5
-10
-15
-20
-25
-30
-35
-40
-45
-50
f1 (ppm)

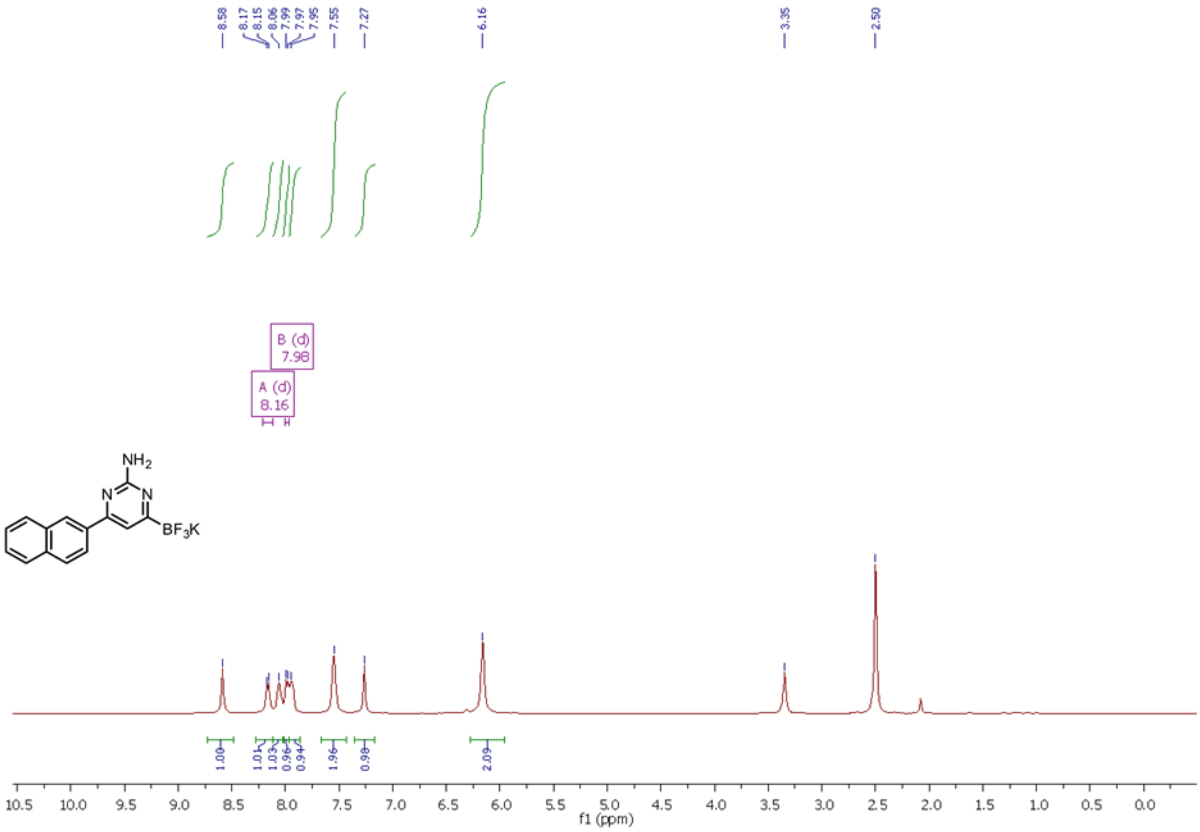
8.58
8.17
8.15
8.06
7.99
7.97
7.95
7.55
7.27
6.16
3.35
2.50
B (d) 7.98
A (d) 8.16
NH_2
N
N
BF_3K
1.00
1.01
1.03
0.96
0.94
1.96
0.98
2.09
f1 (ppm)

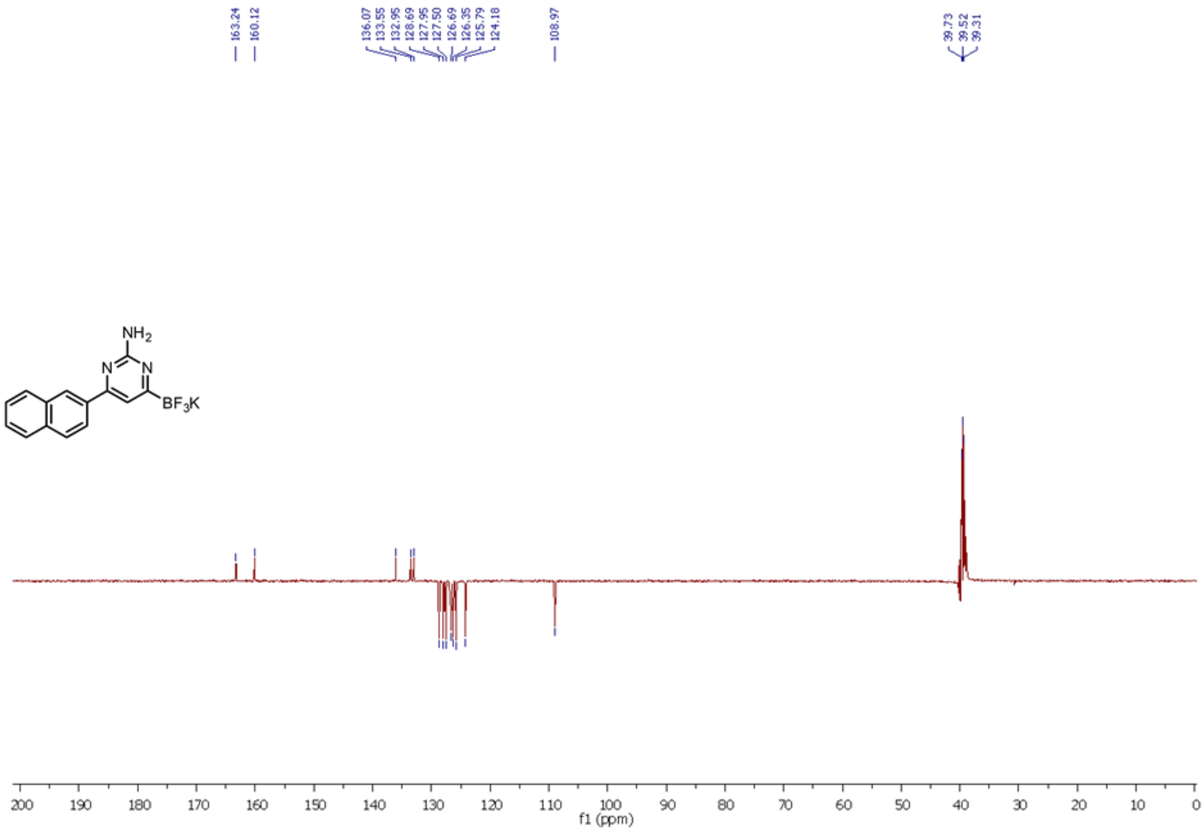
163.24
160.12
136.07
133.55
132.95
128.69
127.95
127.50
126.69
126.35
125.79
124.18
108.97
39.73
39.52
39.31
NH_2
N
N
BF_3K
f1 (ppm)

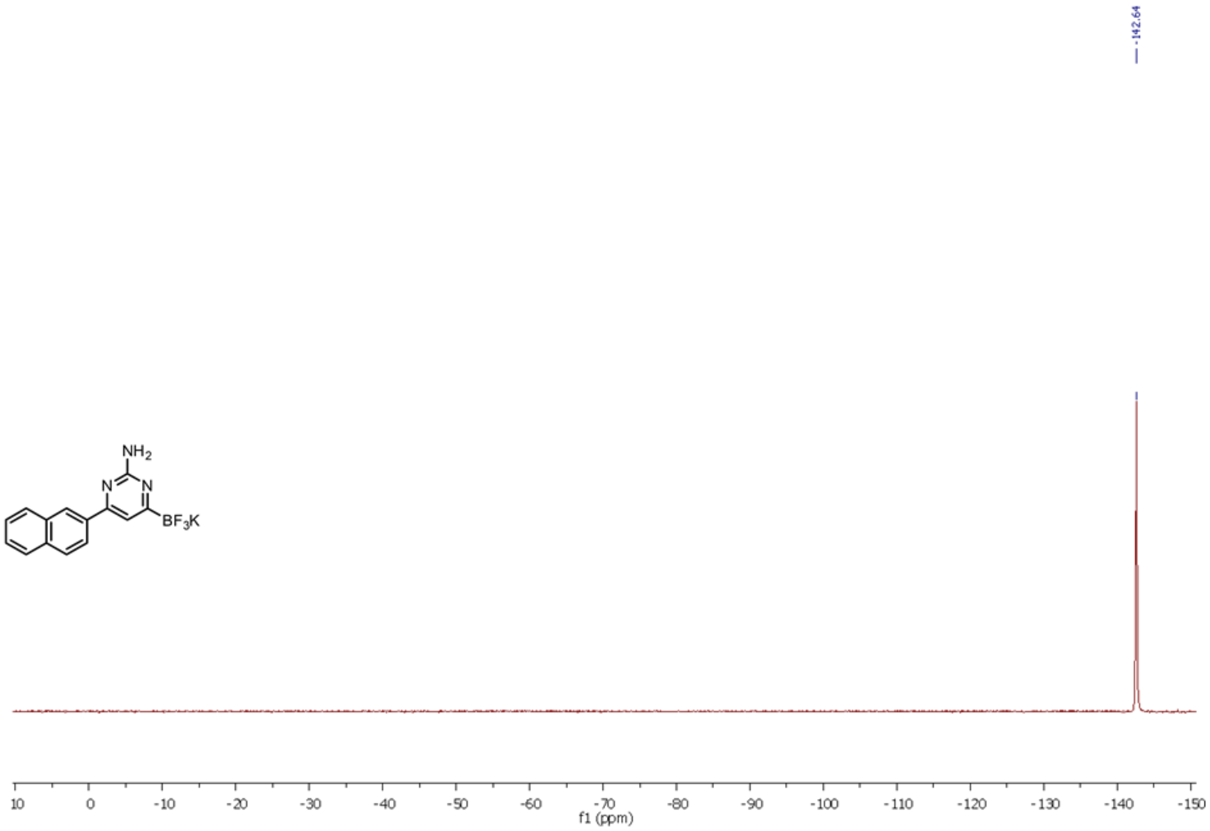
-142.64
NH2
N
N
BF3K
10 0 -10 -20 -30 -40 -50 -60 -70 -80 -90 -100 -110 -120 -130 -140 -150
f1 (ppm)

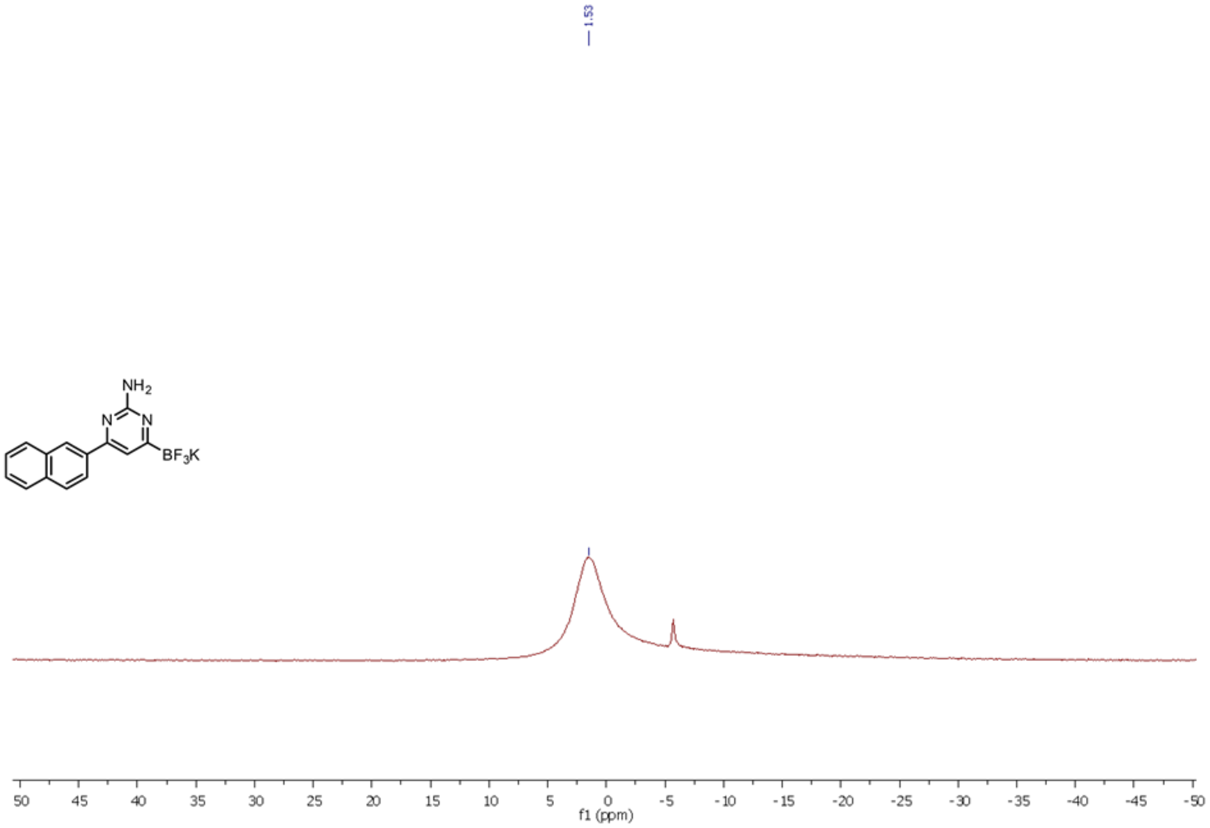
1.53
NH2
N
N
BF3K
50 45 40 35 30 25 20 15 10 5 0 -5 -10 -15 -20 -25 -30 -35 -40 -45 -50
f1 (ppm)

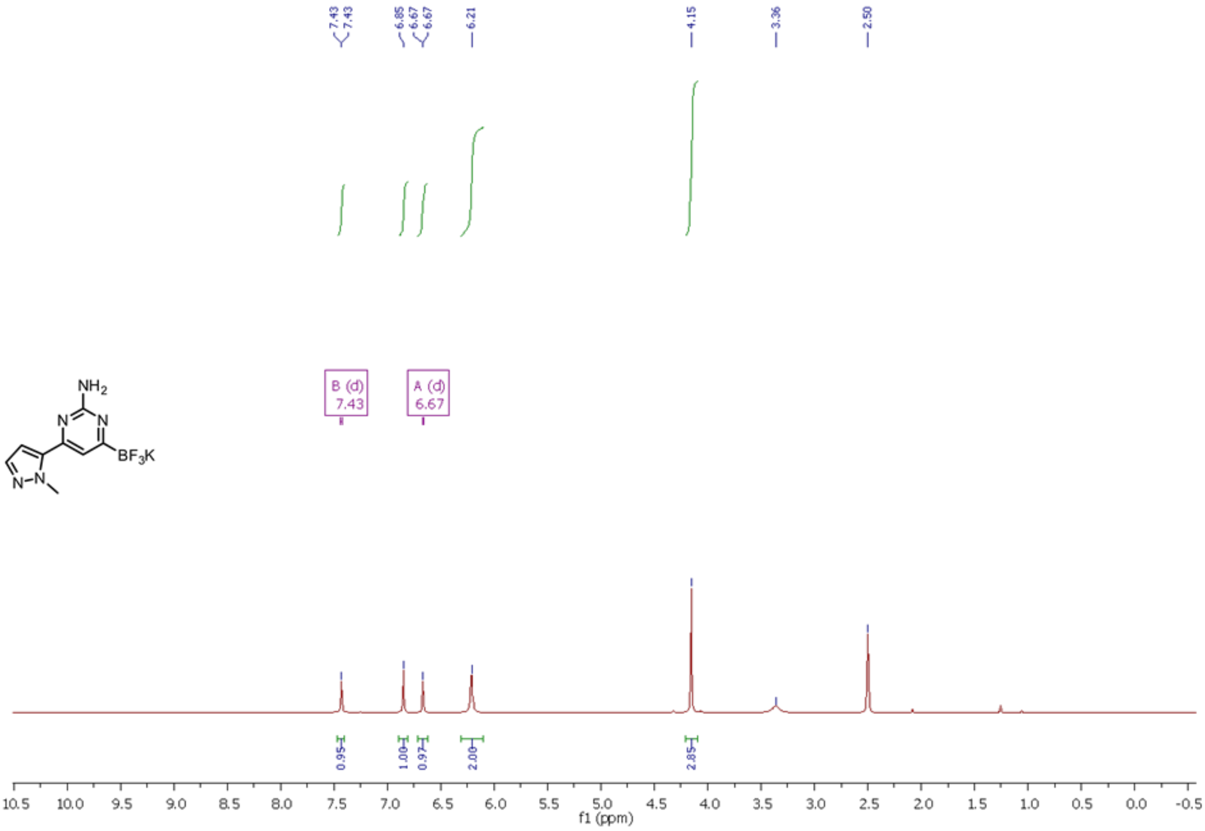
7.43
7.43
6.85
6.67
6.67
6.21
4.15
3.36
2.50
NH2
N
N
BF3K
N
N
B (d)
7.43
A (d)
6.67
0.95
1.00
0.97
2.00
2.85
10.5
10.0
9.5
9.0
8.5
8.0
7.5
7.0
6.5
6.0
5.5
5.0
4.5
4.0
3.5
3.0
2.5
2.0
1.5
1.0
0.5
0.0
-0.5
f1 (ppm)

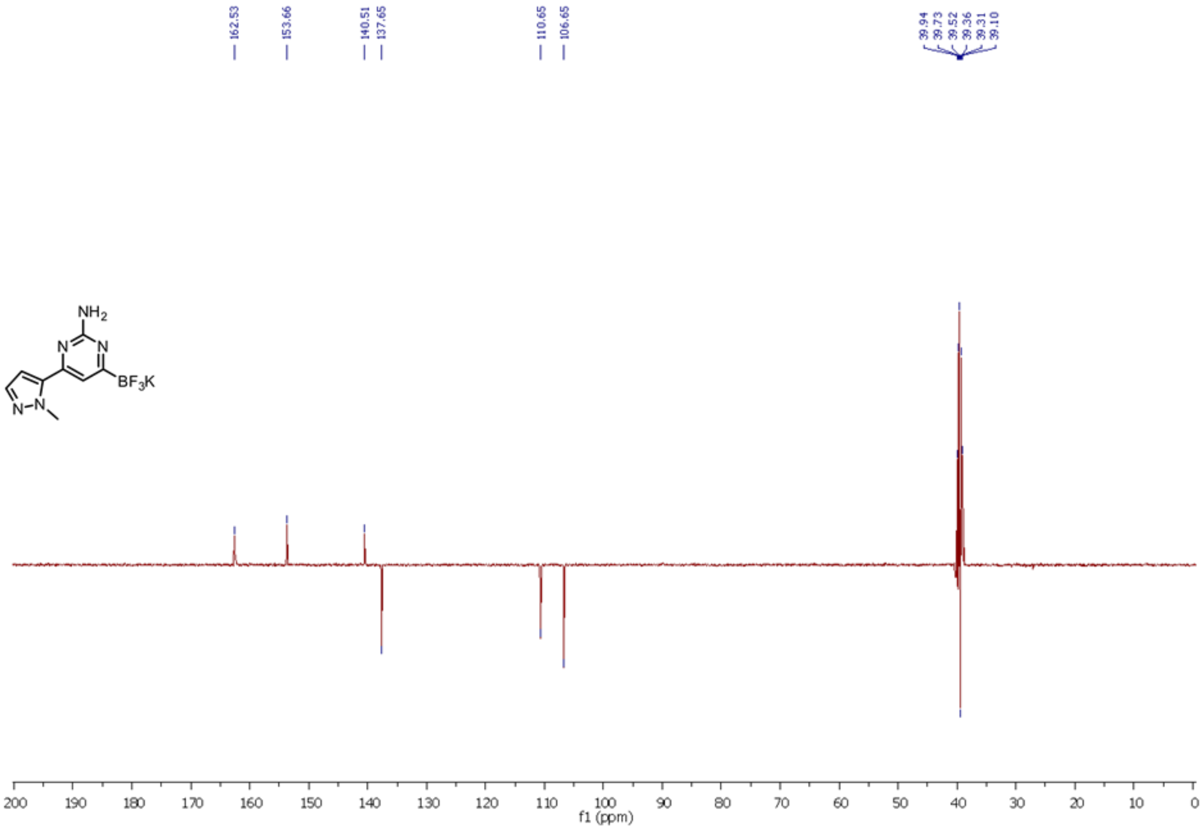
162.53
153.66
140.51
137.65
110.65
106.65
39.94
39.73
39.52
39.36
39.31
39.10
NH2
N
N
BF3K
N
N
200
190
180
170
160
150
140
130
120
110
100
90
80
70
60
50
40
30
20
10
0
f1 (ppm)

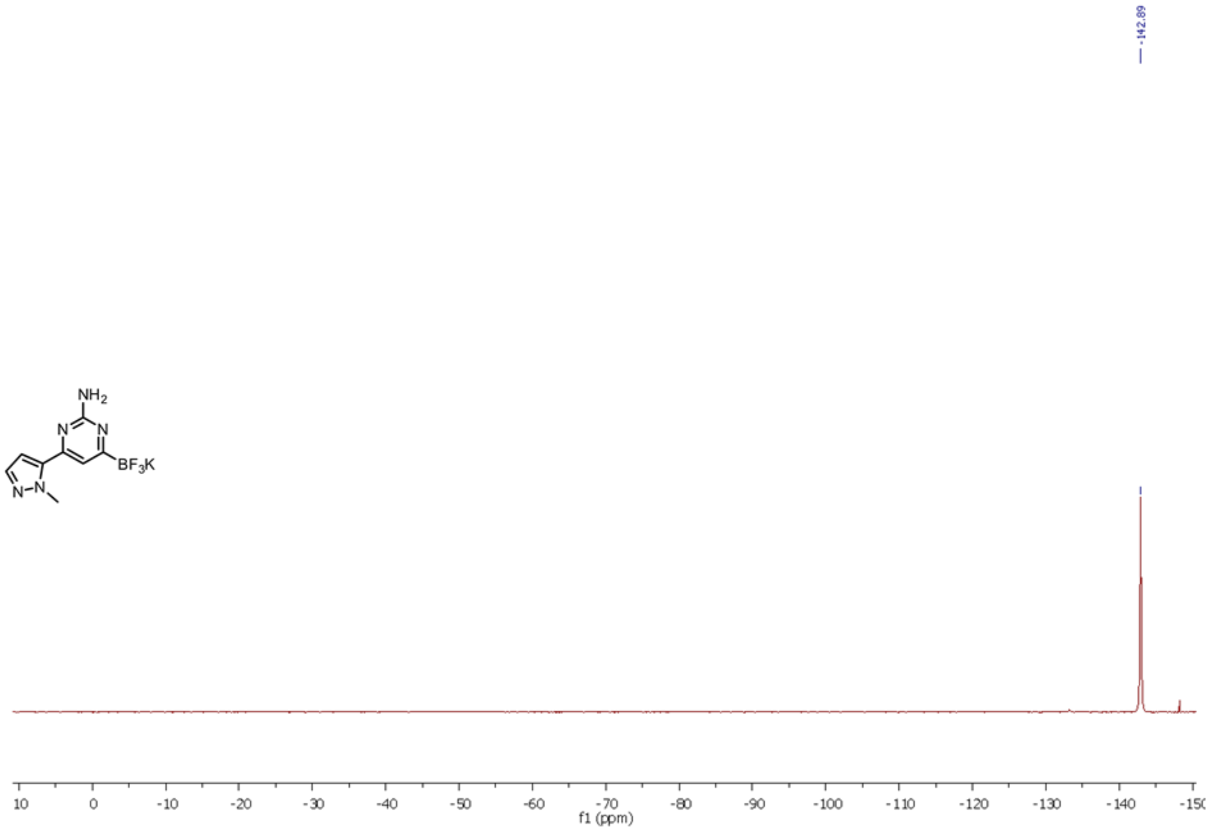
-142.89
NH2
N
N
BF3K
N-N
10
0
-10
-20
-30
-40
-50
-60
-70
-80
-90
-100
-110
-120
-130
-140
f1 (ppm)

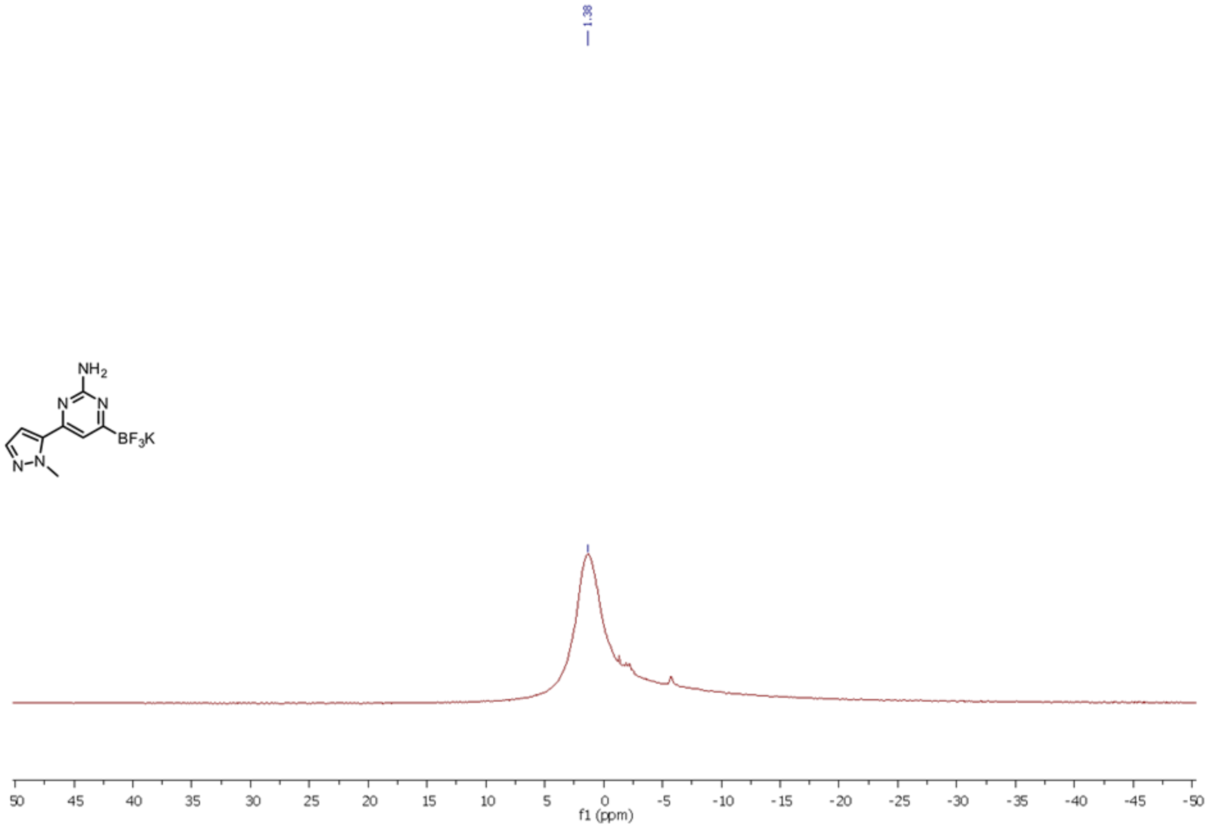
1.38
NH2
N
N
BF3K
N-N
50
45
40
35
30
25
20
15
10
5
0
-5
-10
-15
-20
-25
-30
-35
-40
-45
-50
f1 (ppm)

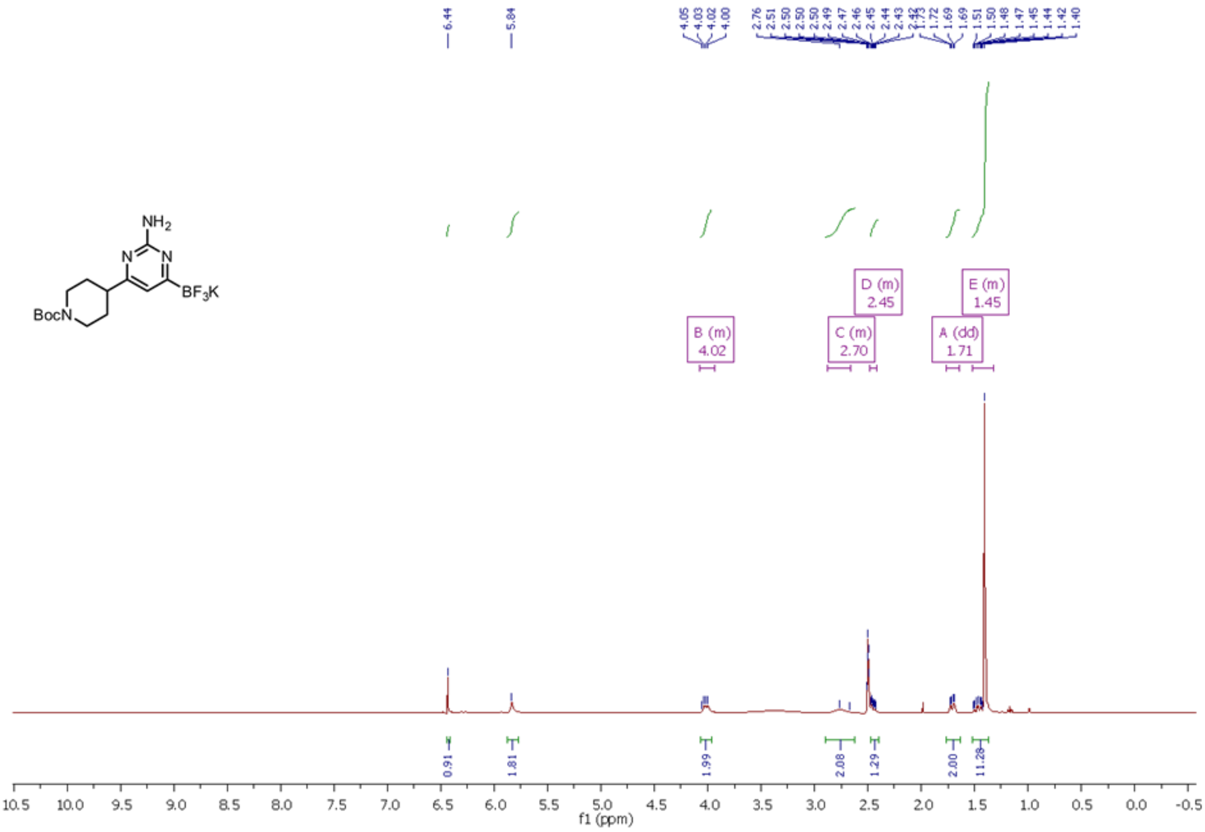

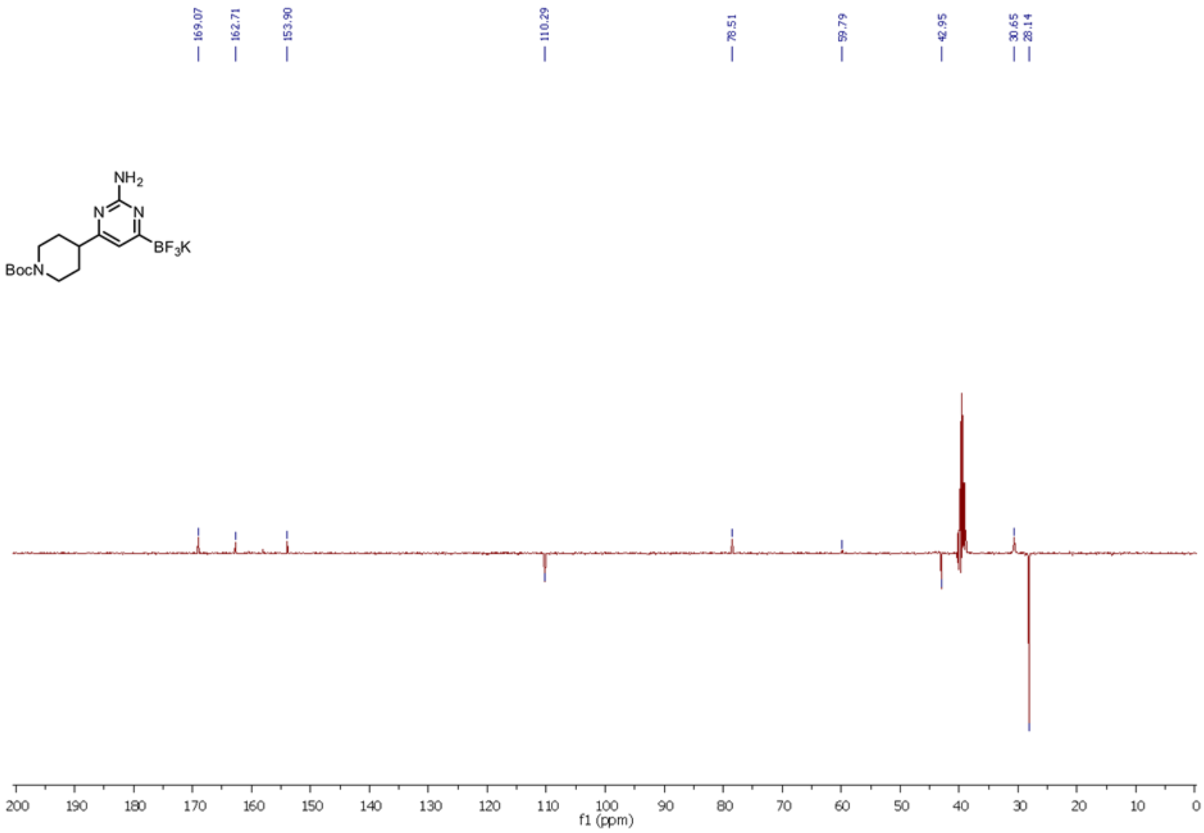

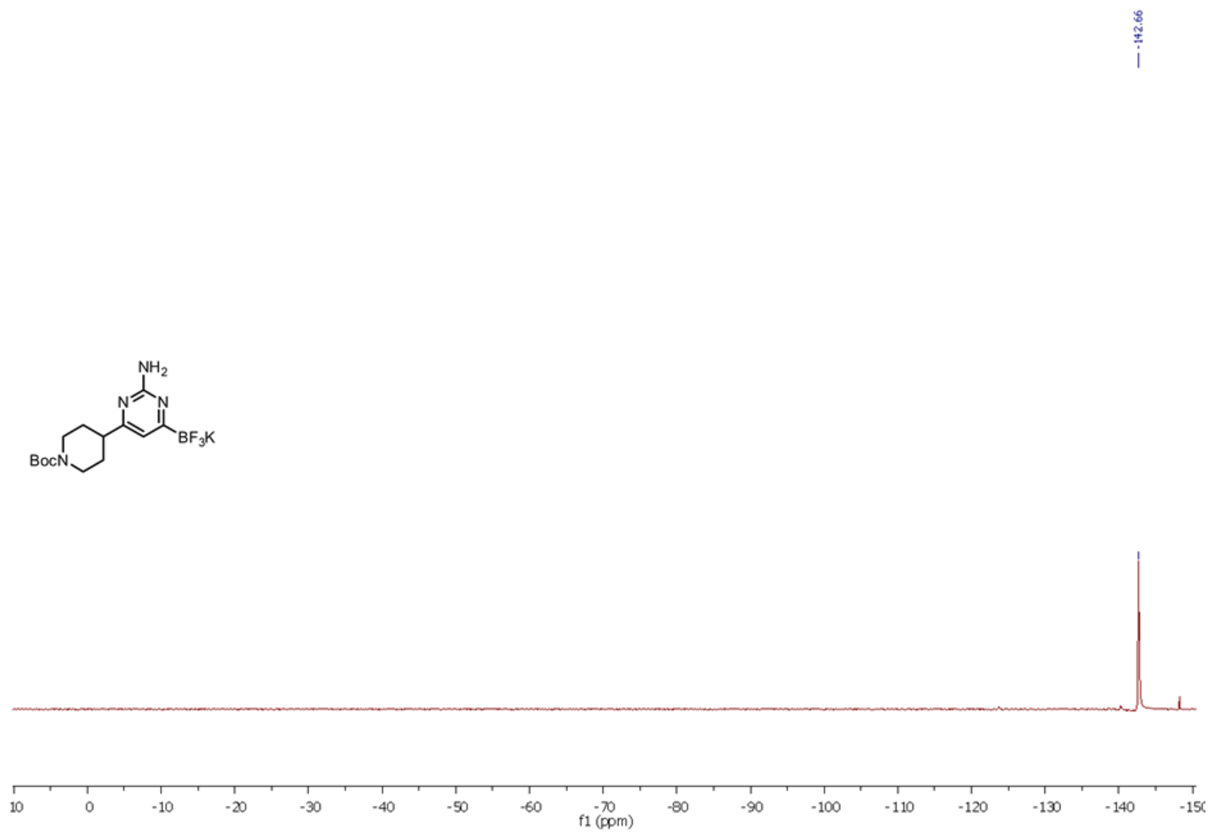
-142.66
NH2
N
N
BF3K
BocN
10
0
-10
-20
-30
-40
-50
-60
-70
-80
-90
-100
-110
-120
-130
-140
-150
f1 (ppm)

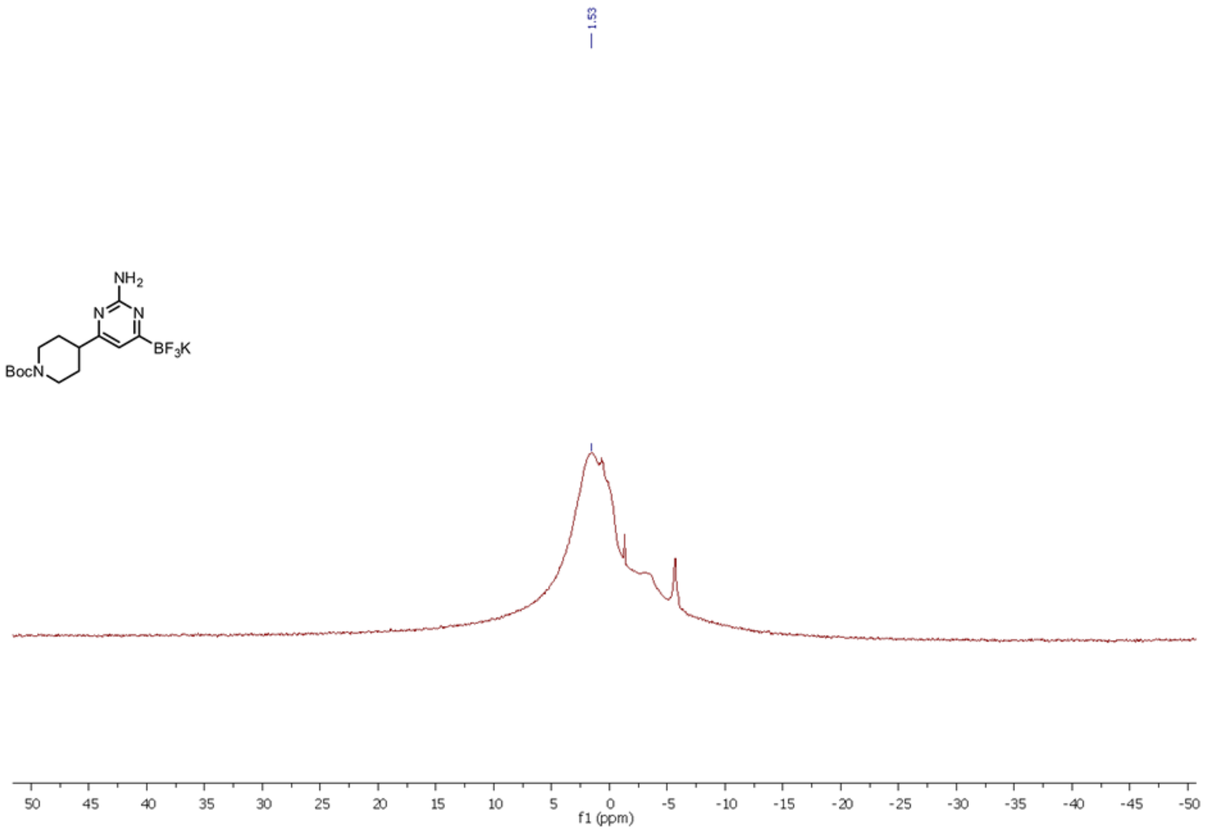
1.53
NH2
N
N
BF3K
BocN
50
45
40
35
30
25
20
15
10
5
0
-5
-10
-15
-20
-25
-30
-35
-40
-45
-50
f1 (ppm)

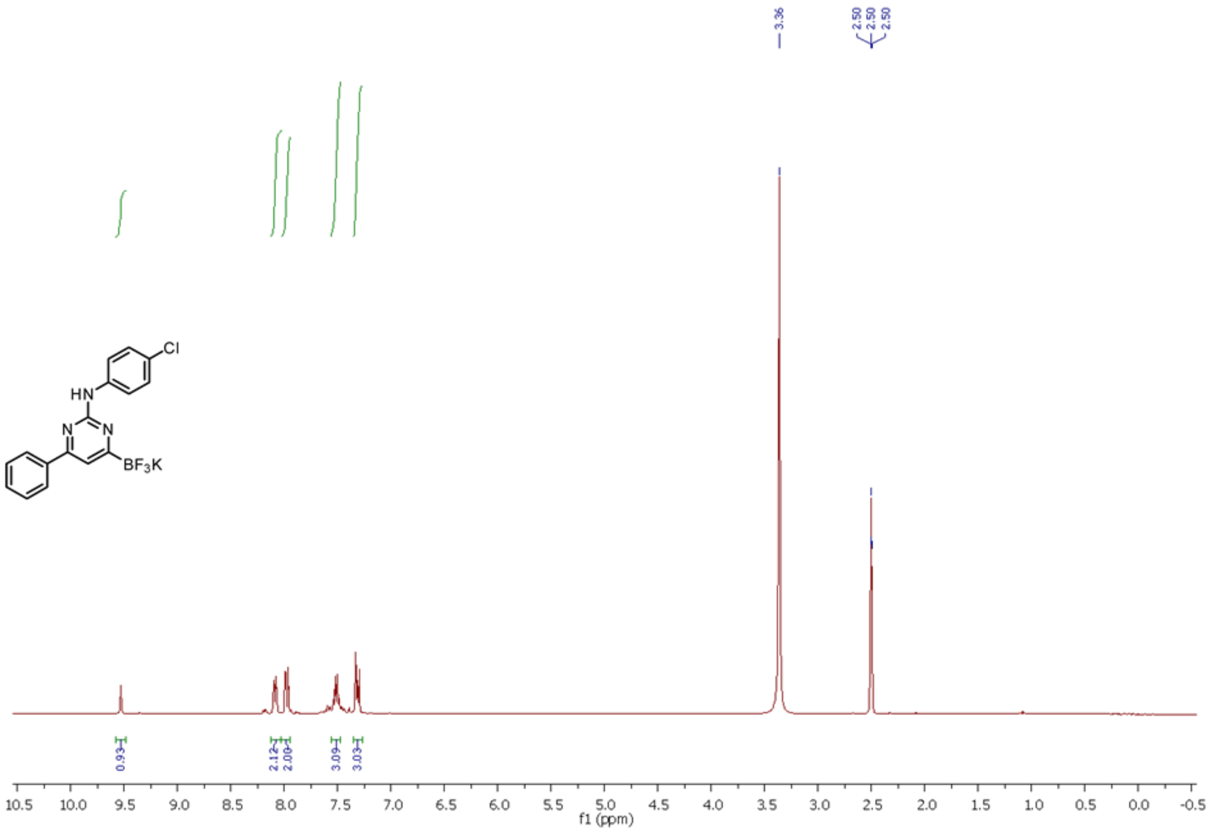
3.36
2.50
2.50
2.50
HN
Cl
N
N
BF3K
0.93
2.12
2.00
3.09
3.03
10.5
10.0
9.5
9.0
8.5
8.0
7.5
7.0
6.5
6.0
5.5
5.0
4.5
4.0
3.5
3.0
2.5
2.0
1.5
1.0
0.5
0.0
-0.5
f1 (ppm)

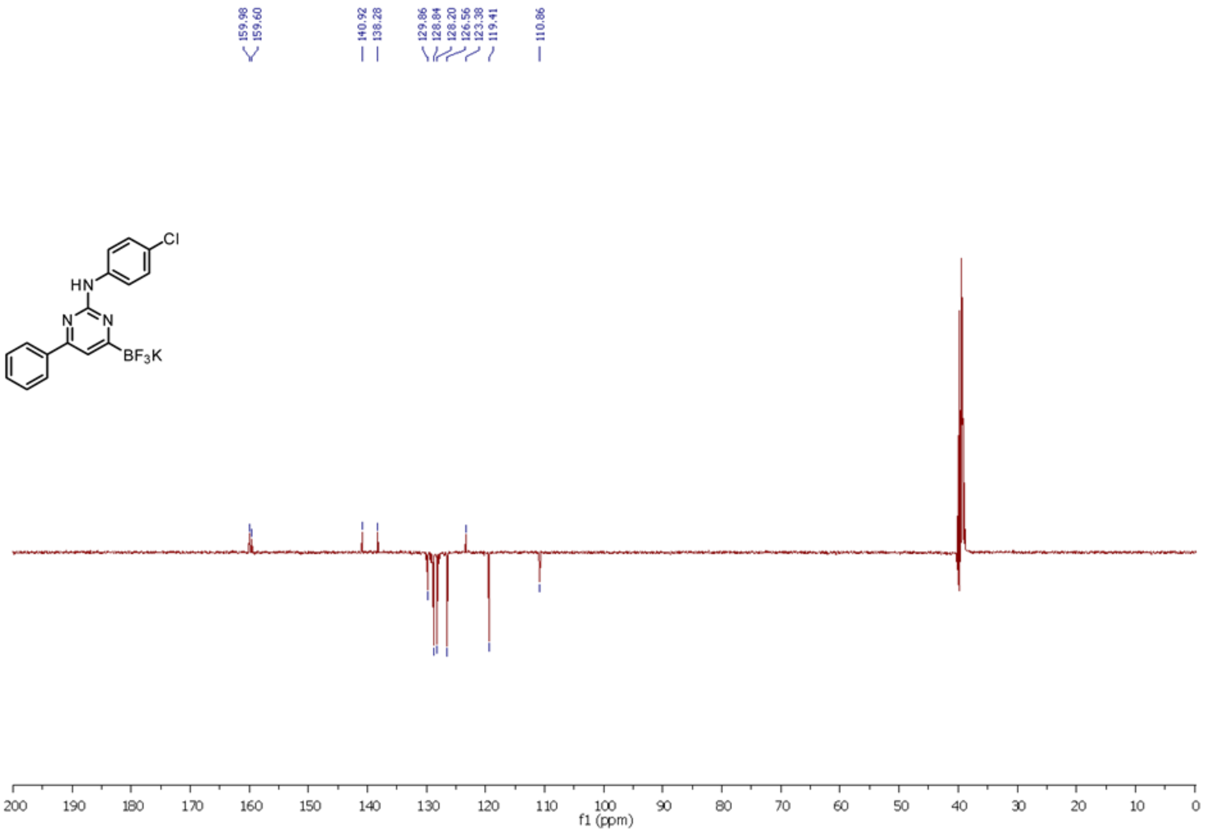
159.98
159.60
140.92
138.28
129.86
128.84
128.20
126.56
123.38
119.41
110.86
HN
Cl
N
N
BF3K
200
190
180
170
160
150
140
130
120
110
100
90
80
70
60
50
40
30
20
10
0
f1 (ppm)

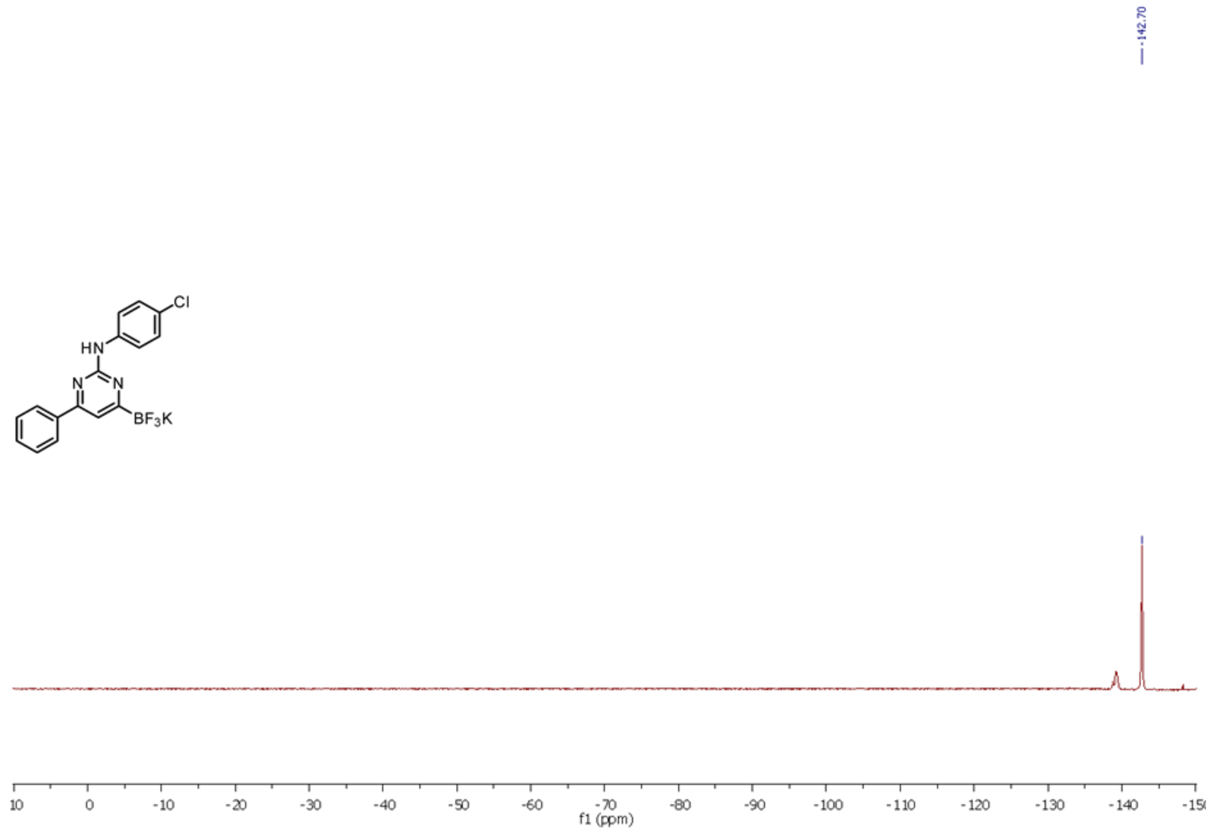
-142.70
Cl
HN
N
N
BF3K
10 0 -10 -20 -30 -40 -50 -60 -70 -80 -90 -100 -110 -120 -130 -140 -150
f1 (ppm)

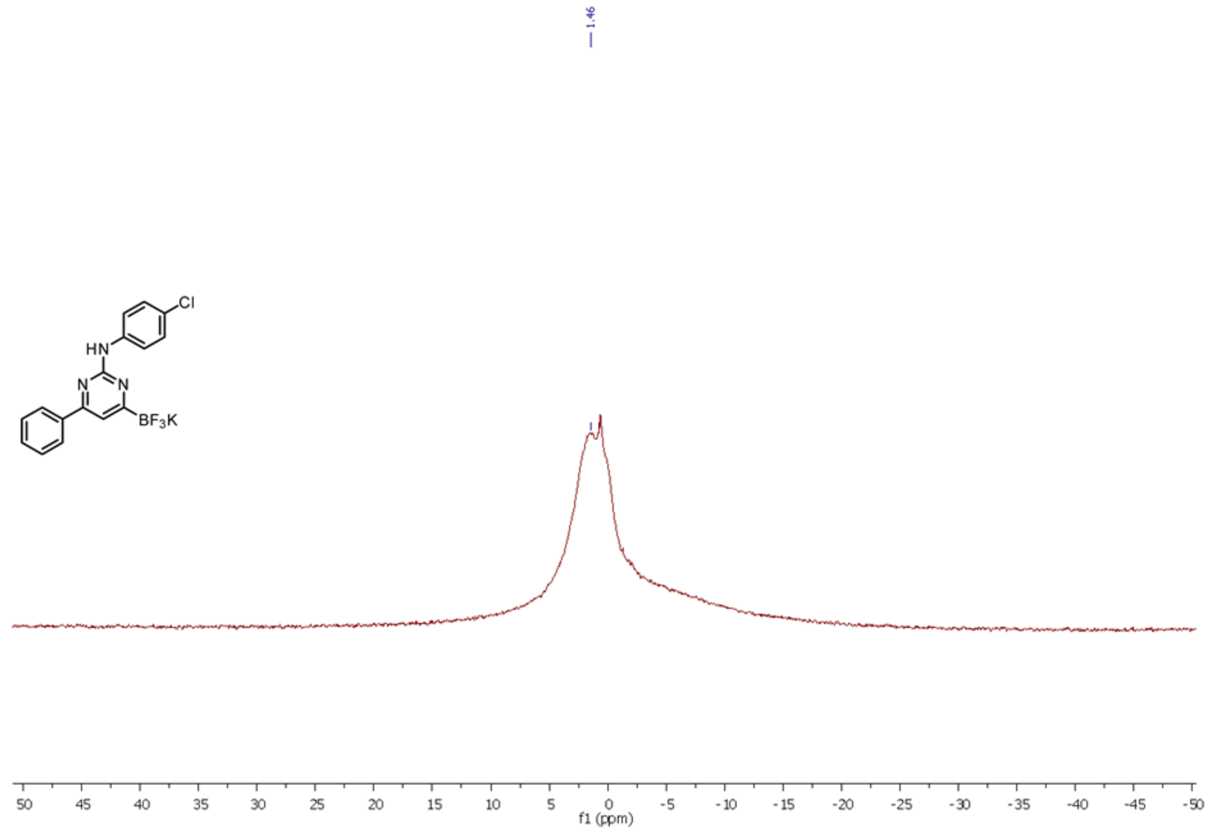
1.46
Cl
HN
N
N
BF3K
50 45 40 35 30 25 20 15 10 5 0 -5 -10 -15 -20 -25 -30 -35 -40 -45 -50
f1 (ppm)

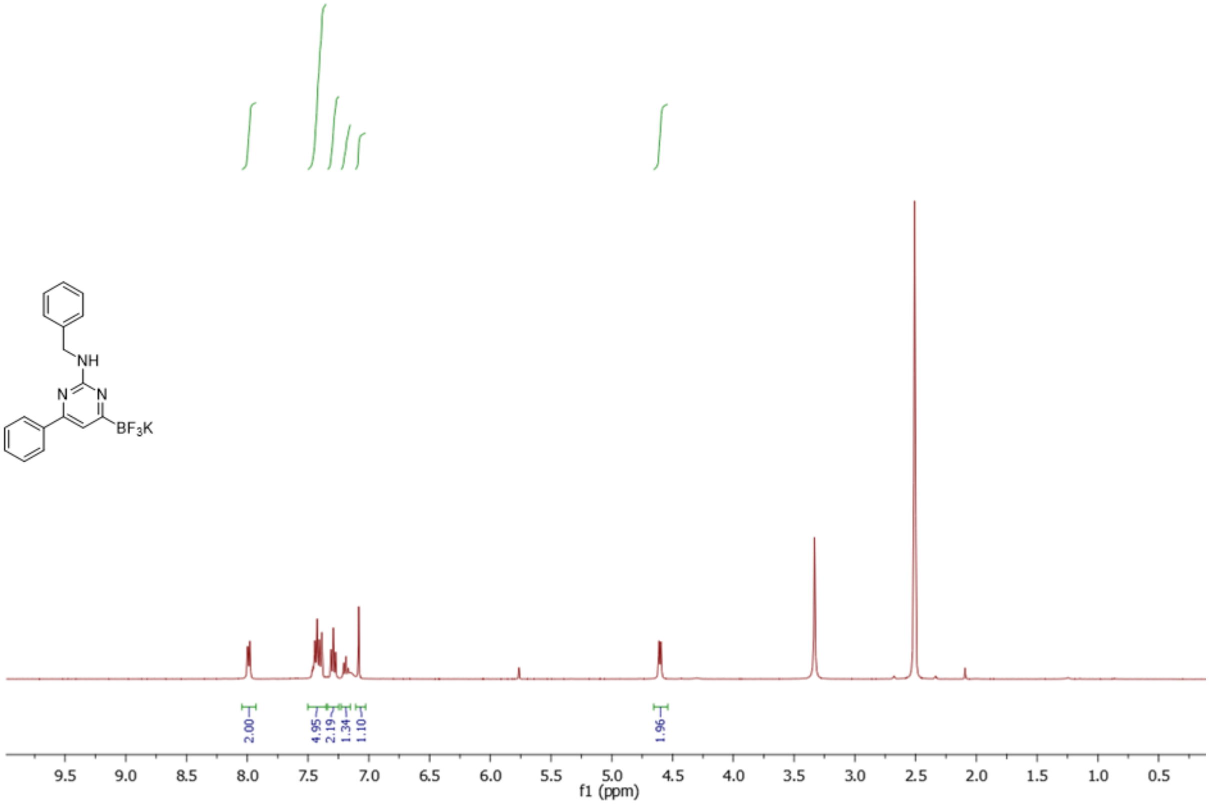

NH
N
N
BF3K
2.00
4.95
2.19
1.34
1.10
1.96
9.5 9.0 8.5 8.0 7.5 7.0 6.5 6.0 5.5 5.0 4.5 4.0 3.5 3.0 2.5 2.0 1.5 1.0 0.5
f1 (ppm)

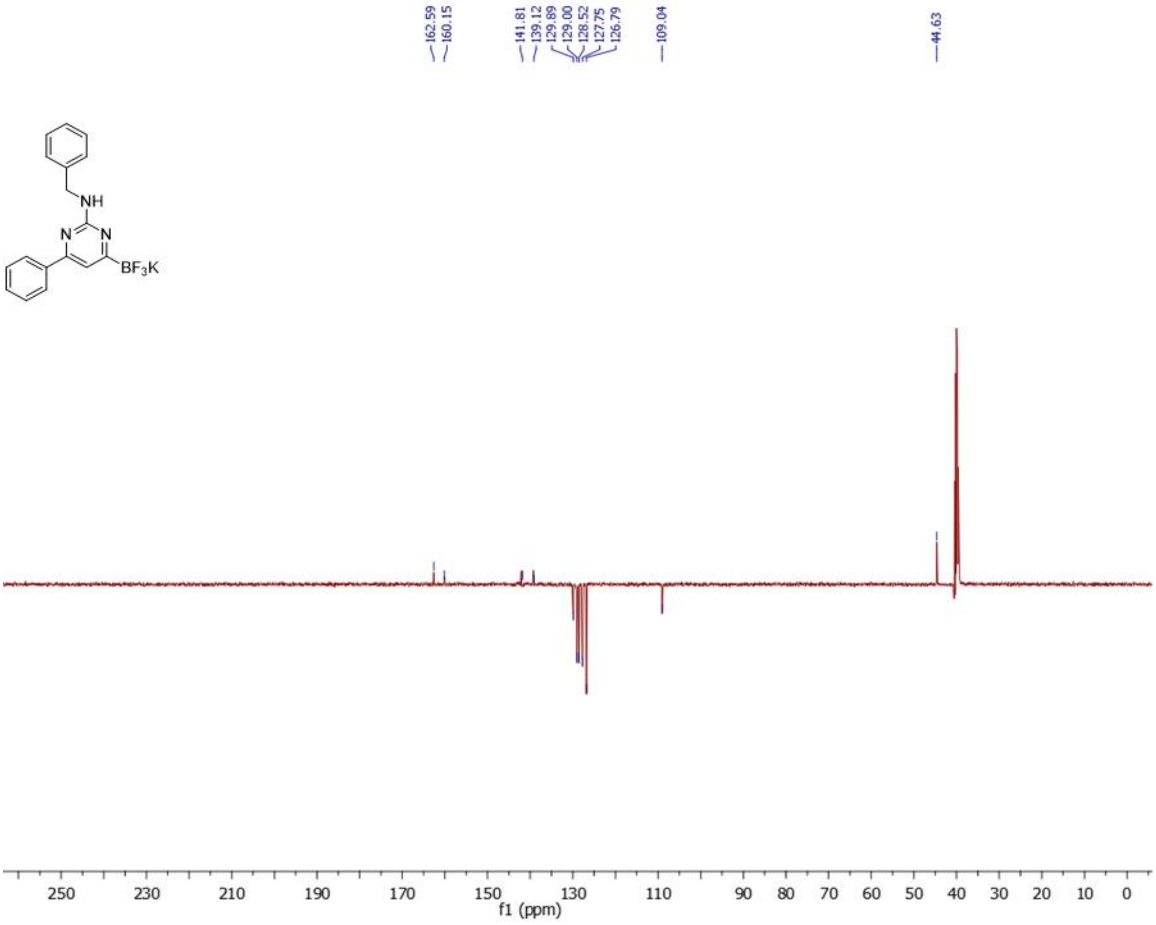

162.59
160.15
141.81
139.12
129.89
129.00
128.52
127.75
126.79
109.04
44.63
NH
N
N
BF3K
250 230 210 190 170 150 130 110 90 80 70 60 50 40 30 20 10 0
f1 (ppm)

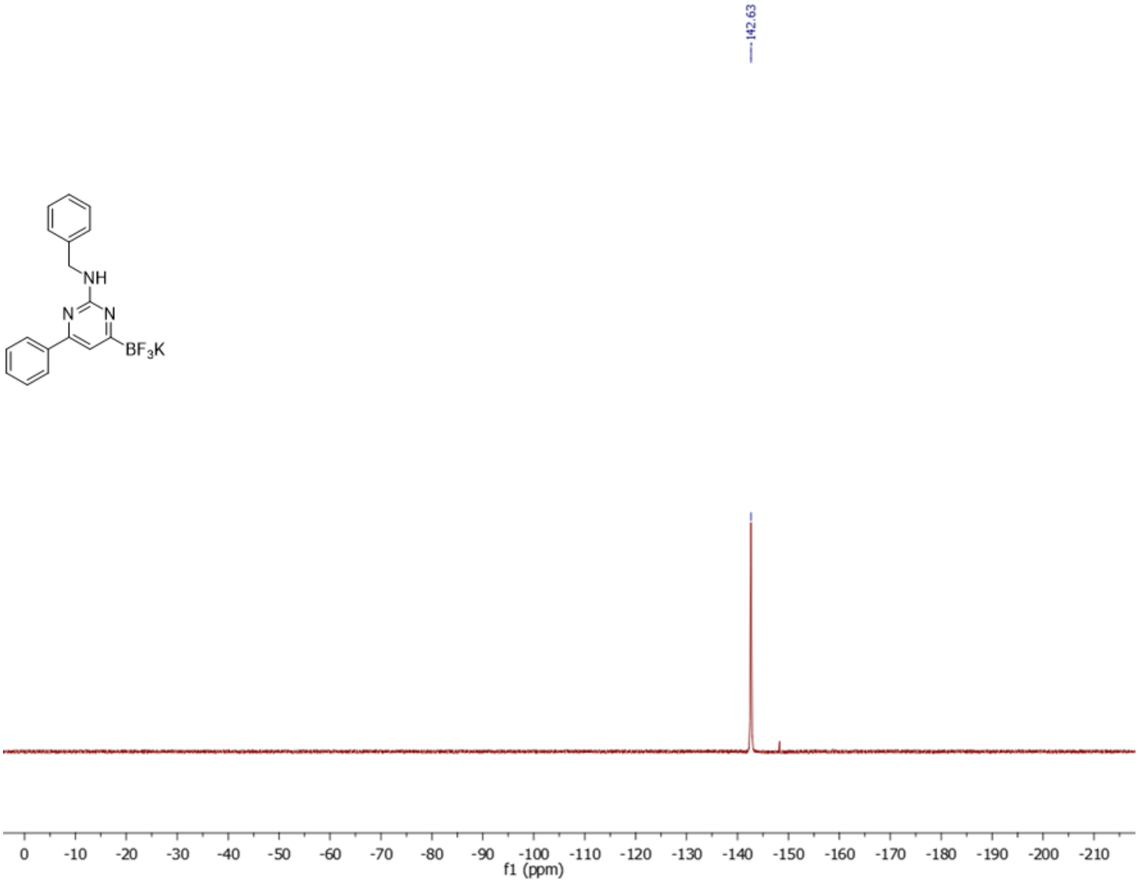
-142.63
NH
N
N
BF_3K
0 -10 -20 -30 -40 -50 -60 -70 -80 -90 -100 -110 -120 -130 -140 -150 -160 -170 -180 -190 -200 -210
f1 (ppm)

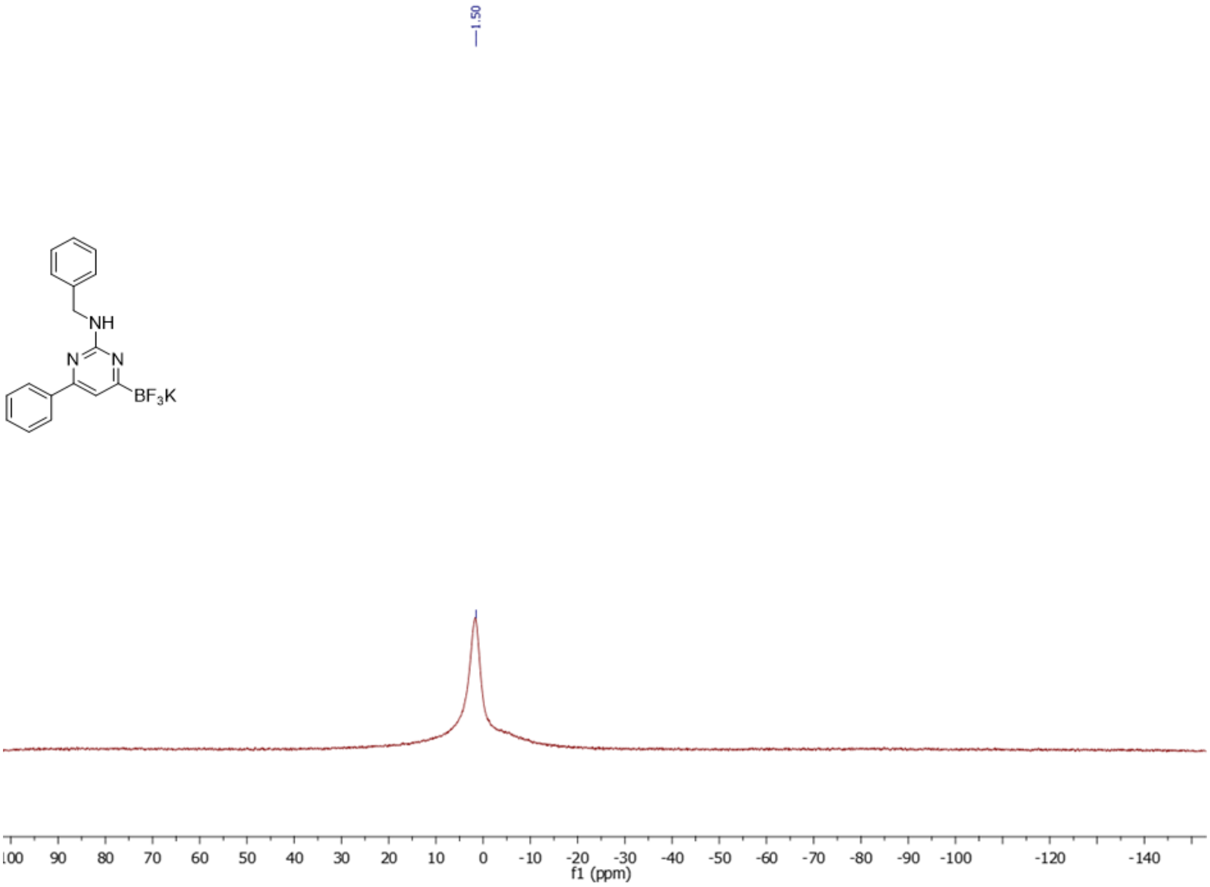
1.50
NH
N
N
BF_3K
100 90 80 70 60 50 40 30 20 10 0 -10 -20 -30 -40 -50 -60 -70 -80 -90 -100 -120 -140
f1 (ppm)

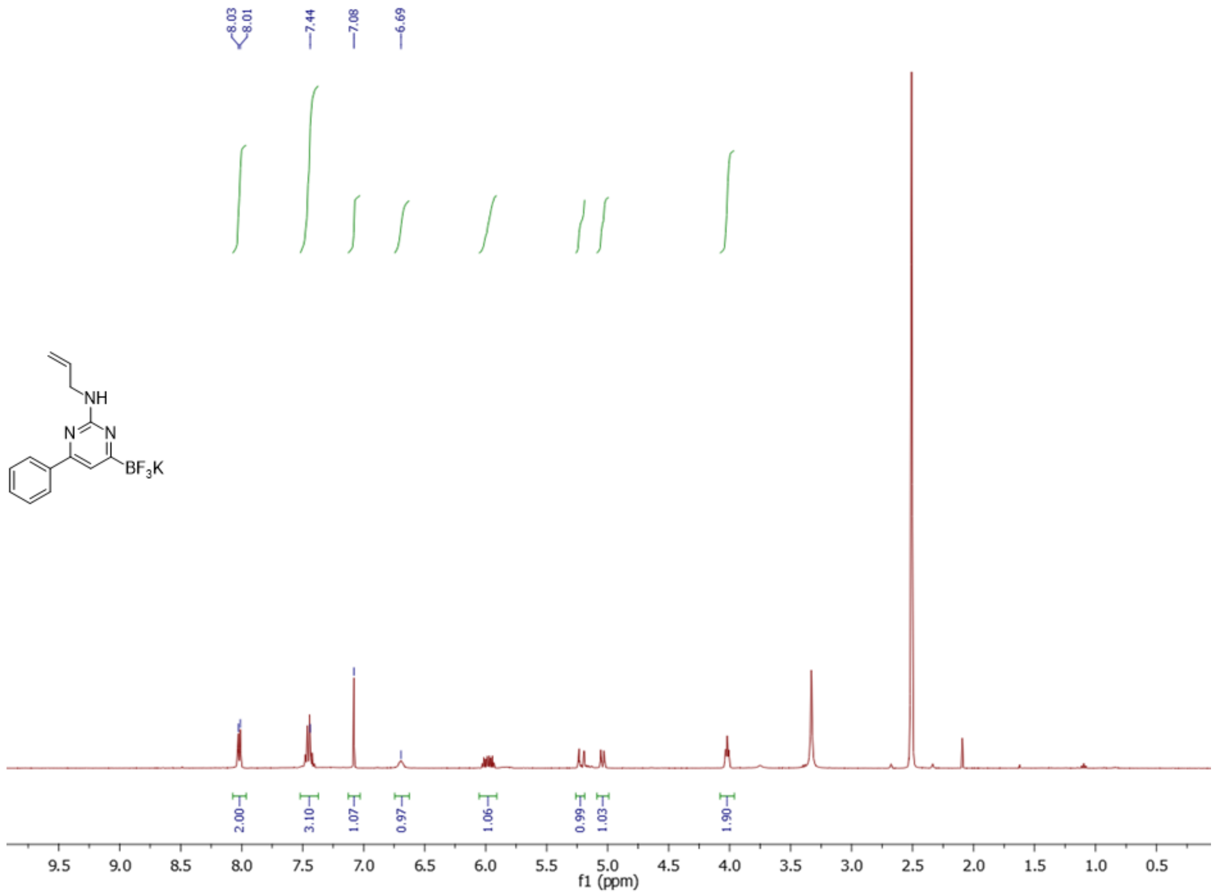
8.03
8.01
7.44
7.08
6.69
NH
N
N
BF3K
2.00
3.10
1.07
0.97
1.06
0.99
1.03
1.90
9.5 9.0 8.5 8.0 7.5 7.0 6.5 6.0 5.5 5.0 4.5 4.0 3.5 3.0 2.5 2.0 1.5 1.0 0.5
f1 (ppm)

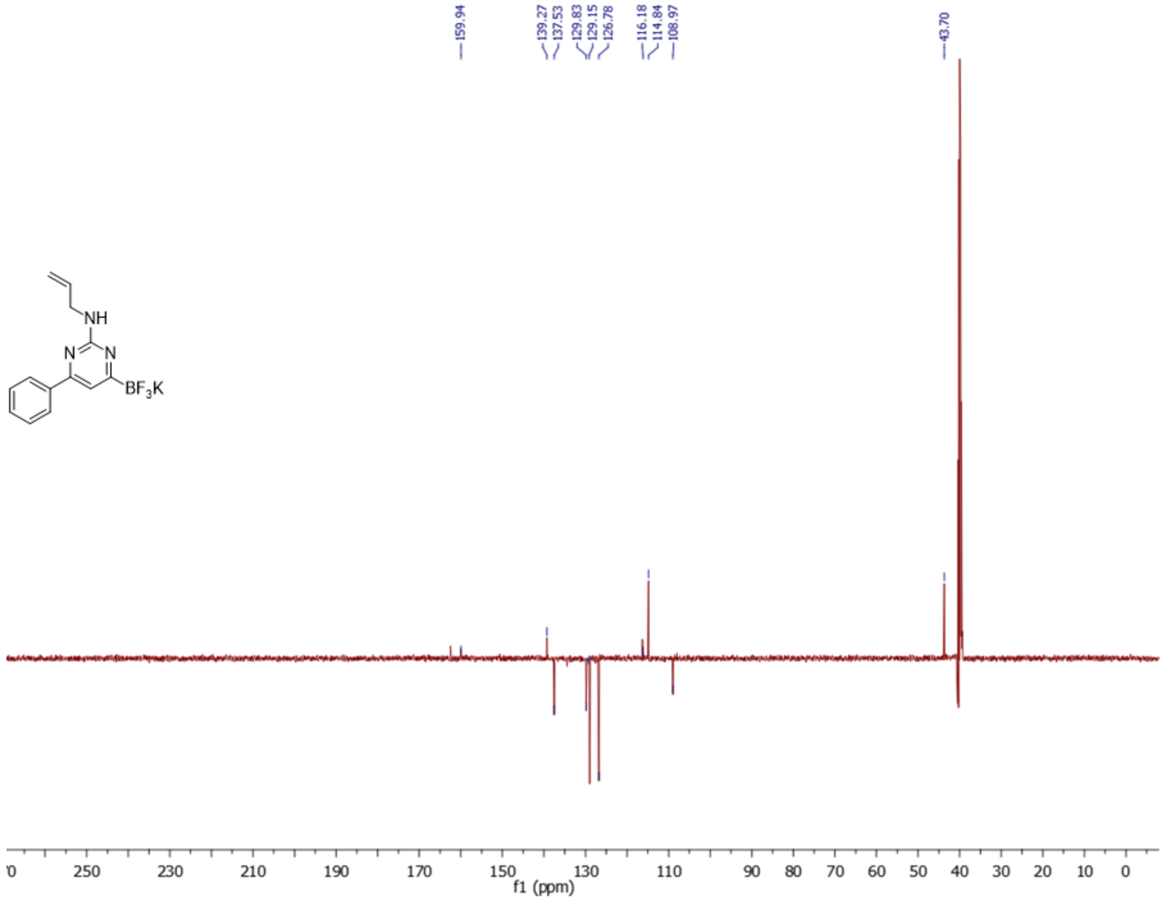
159.94
139.27
137.53
129.83
129.15
126.78
116.18
114.84
108.97
43.70
NH
N
N
BF3K
250 230 210 190 170 150 130 110 90 80 70 60 50 40 30 20 10 0
f1 (ppm)

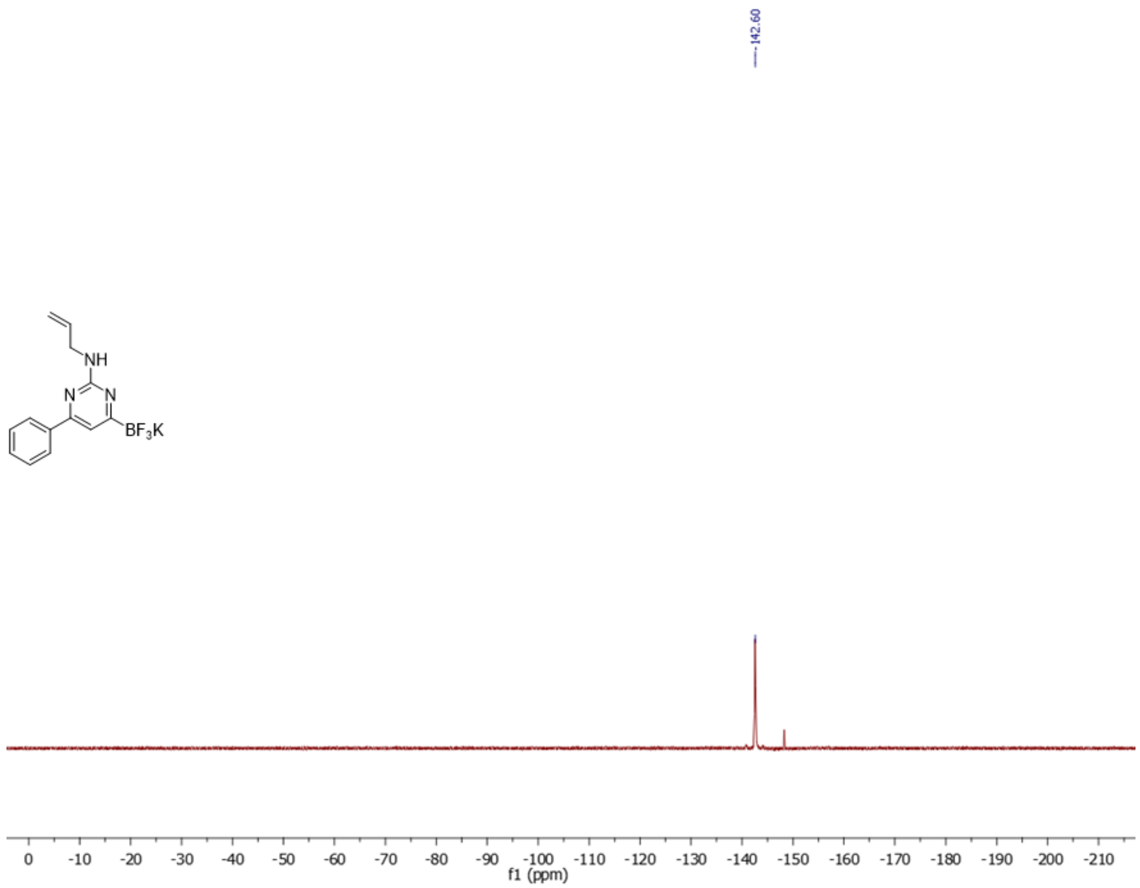

-142.60
NH
N
N
BF3K
0
-10
-20
-30
-40
-50
-60
-70
-80
-90
-100
-110
-120
-130
-140
-150
-160
-170
-180
-190
-200
-210
f1 (ppm)

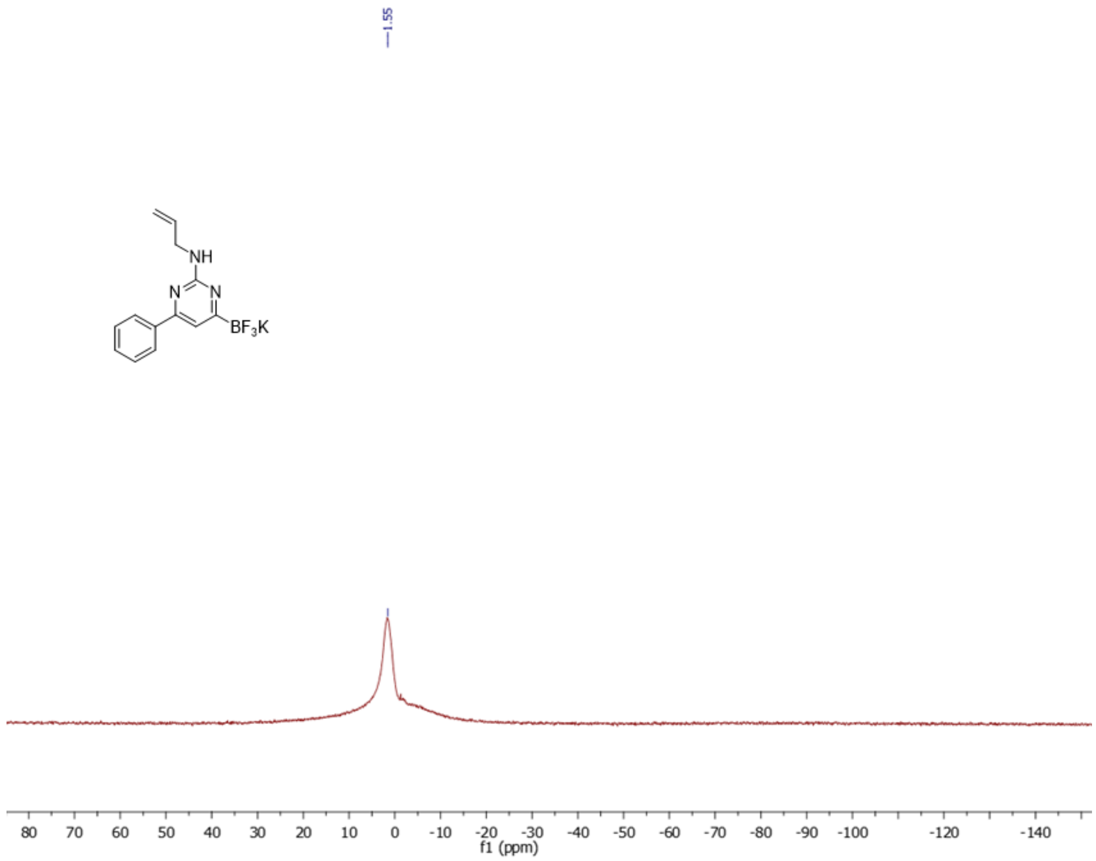

1.55
NH
N
N
BF3K
80
70
60
50
40
30
20
10
0
-10
-20
-30
-40
-50
-60
-70
-80
-90
-100
-120
-140
f1 (ppm)

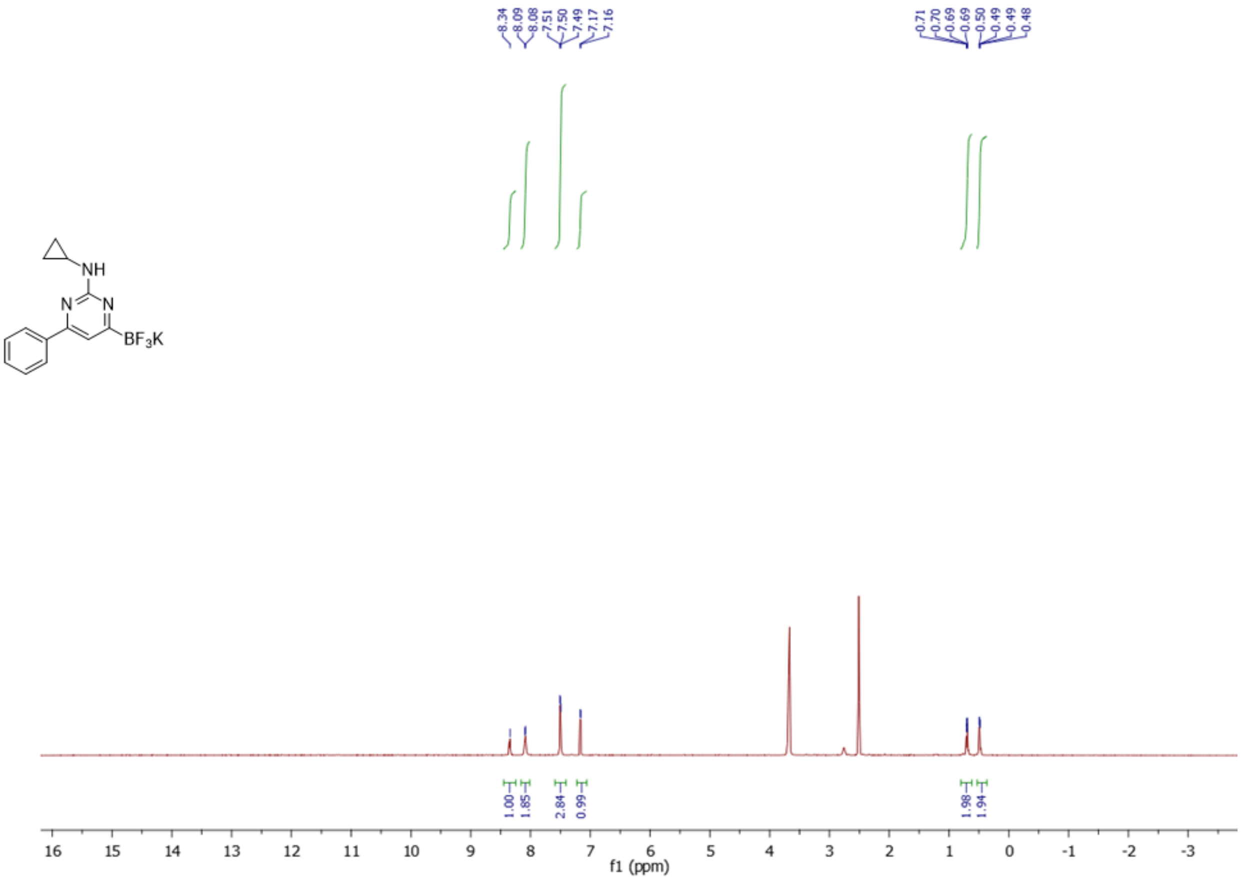
8.34
8.09
8.08
7.51
7.50
7.49
7.17
7.16
0.71
0.70
0.69
0.69
0.50
0.49
0.49
0.48
NH
N
N
BF3K
1.00
1.85
2.84
0.99
1.98
1.94
16 15 14 13 12 11 10 9 8 7 6 5 4 3 2 1 0 -1 -2 -3
f1 (ppm)

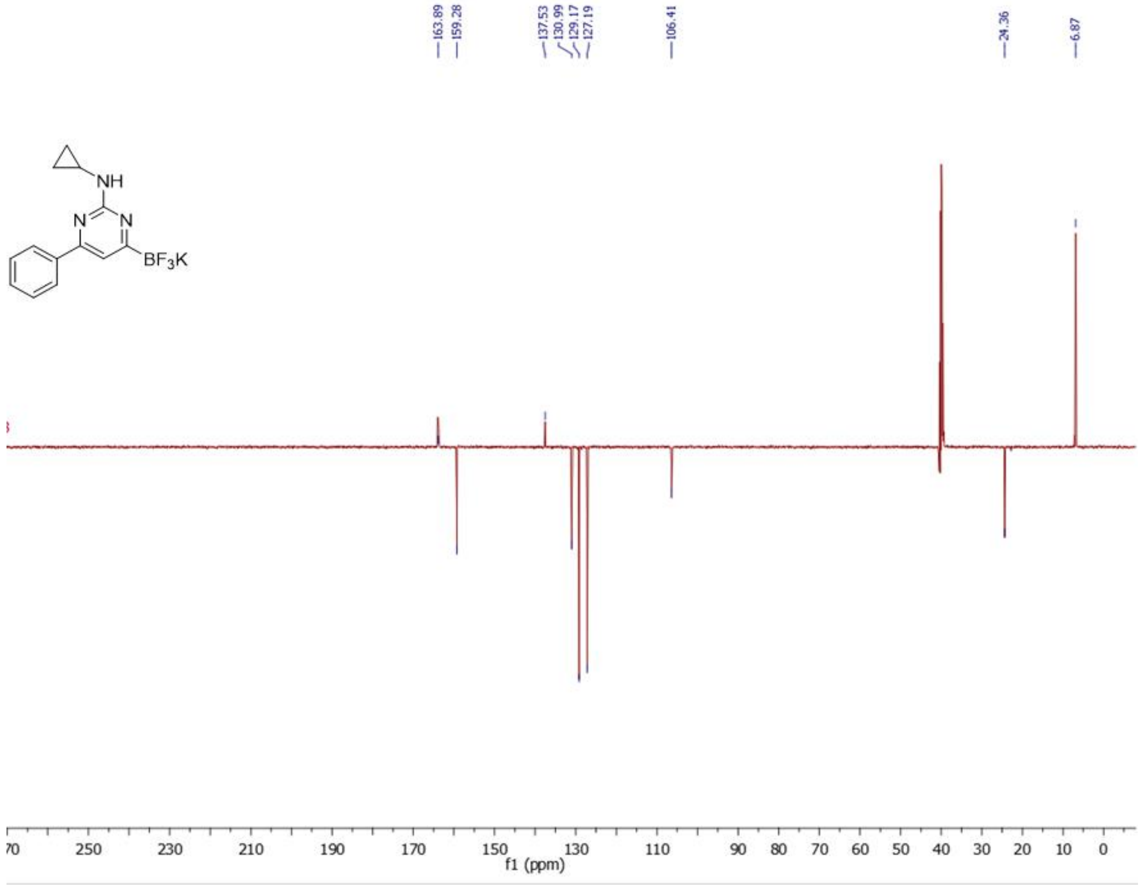
163.89
159.28
137.53
130.99
129.17
127.19
106.41
24.36
6.87
NH
N
N
BF3K
70 250 230 210 190 170 150 130 110 90 80 70 60 50 40 30 20 10 0
f1 (ppm)

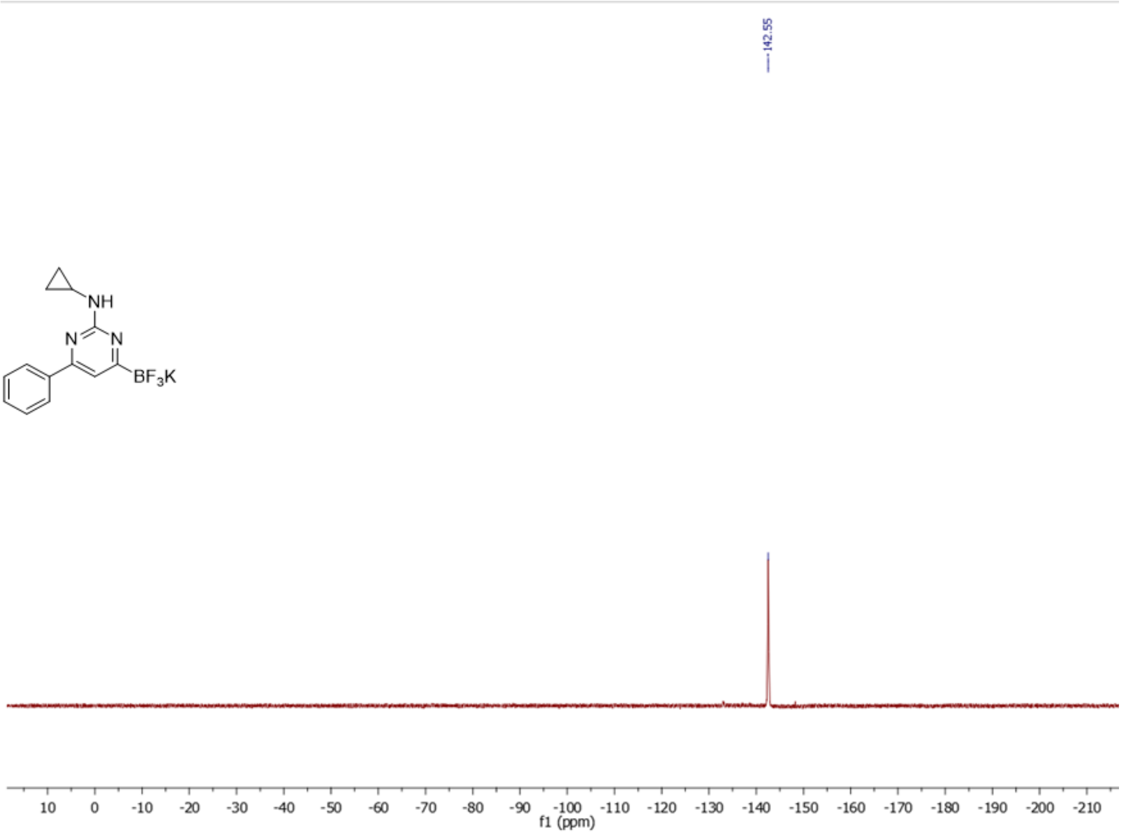
142.55
NH
N
N
BF$_3$K
10 0 -10 -20 -30 -40 -50 -60 -70 -80 -90 -100 -110 -120 -130 -140 -150 -160 -170 -180 -190 -200 -210
f1 (ppm)

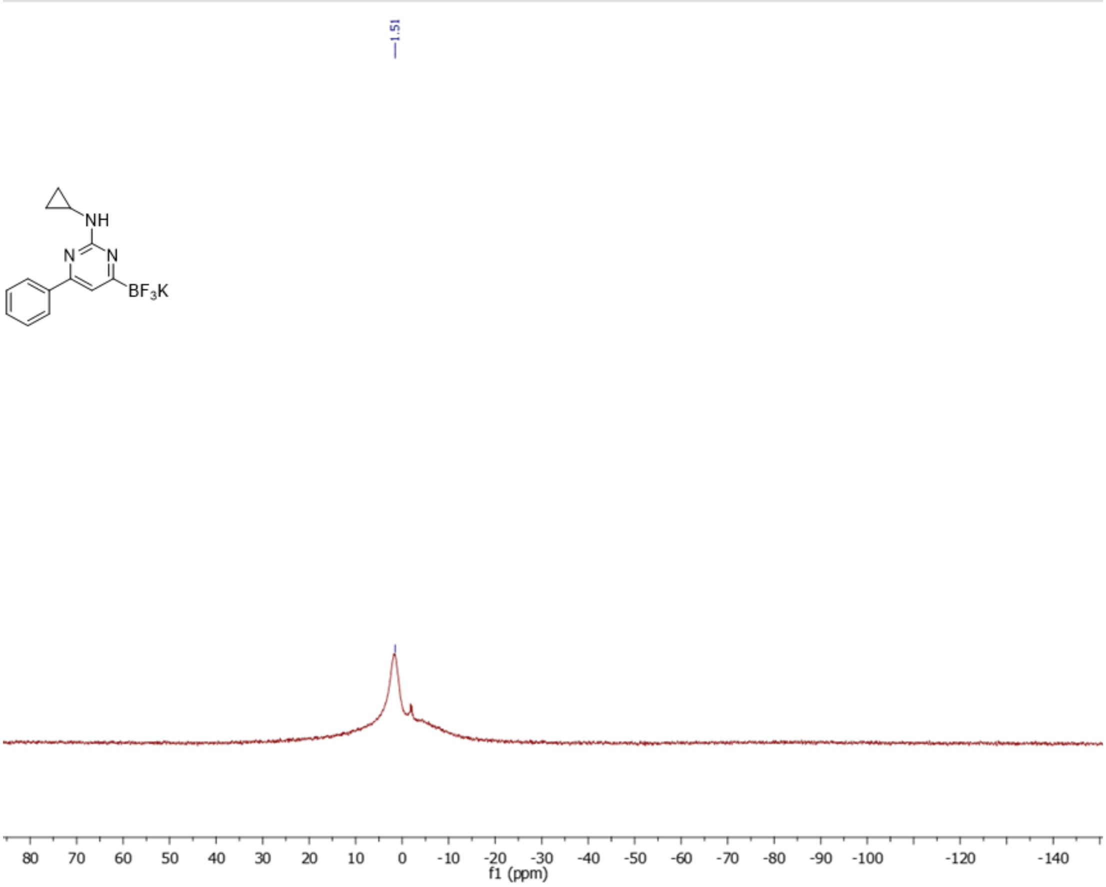
1.51
NH
N
N
BF$_3$K
80 70 60 50 40 30 20 10 0 -10 -20 -30 -40 -50 -60 -70 -80 -90 -100 -120 -140
f1 (ppm)

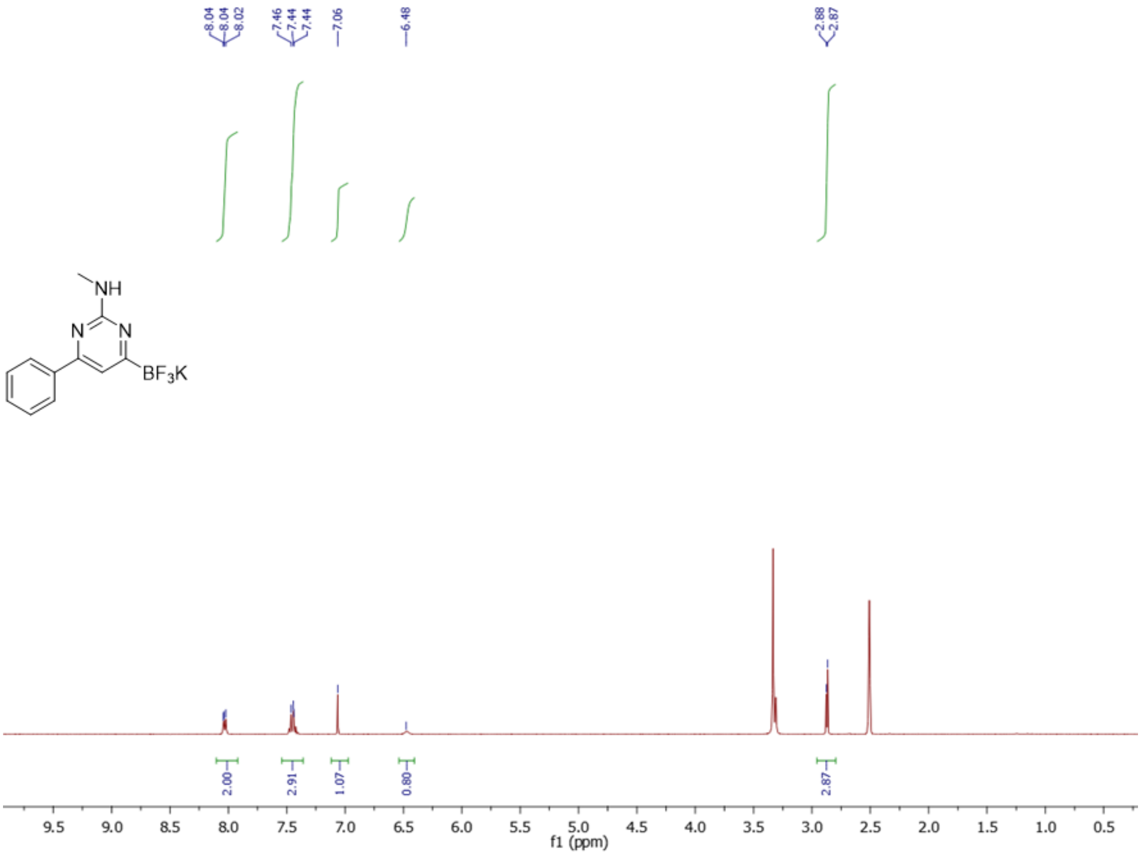
NH
N
N
BF3K
8.04
8.04
8.02
7.46
7.44
7.44
7.06
6.48
2.88
2.87
2.00
2.91
1.07
0.80
2.87
9.5
9.0
8.5
8.0
7.5
7.0
6.5
6.0
5.5
5.0
4.5
4.0
3.5
3.0
2.5
2.0
1.5
1.0
0.5
f1 (ppm)

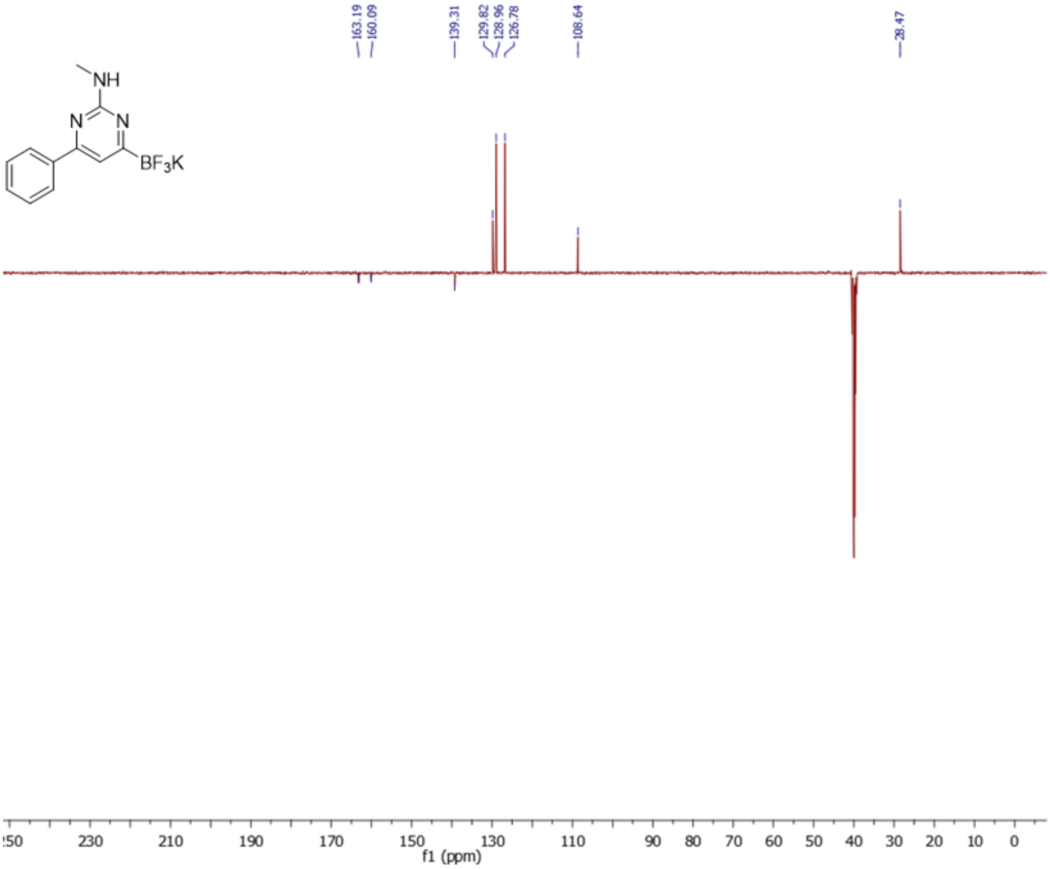
NH
N
N
BF3K
163.19
160.09
139.31
129.82
128.96
126.78
108.64
28.47
230
210
190
170
150
130
110
90
80
70
60
50
40
30
20
10
0
f1 (ppm)

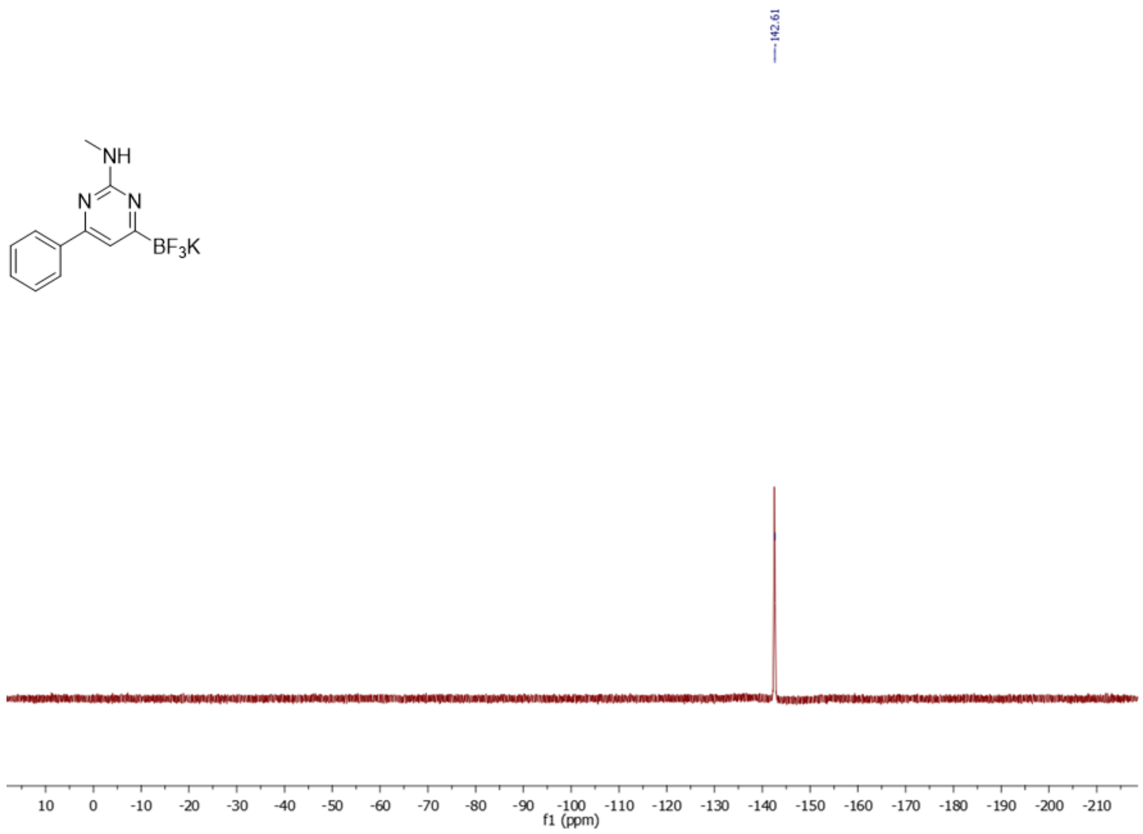
-142.61
NH
N
N
BF3K
10
0
-10
-20
-30
-40
-50
-60
-70
-80
-90
-100
-110
-120
-130
-140
-150
-160
-170
-180
-190
-200
-210
f1 (ppm)

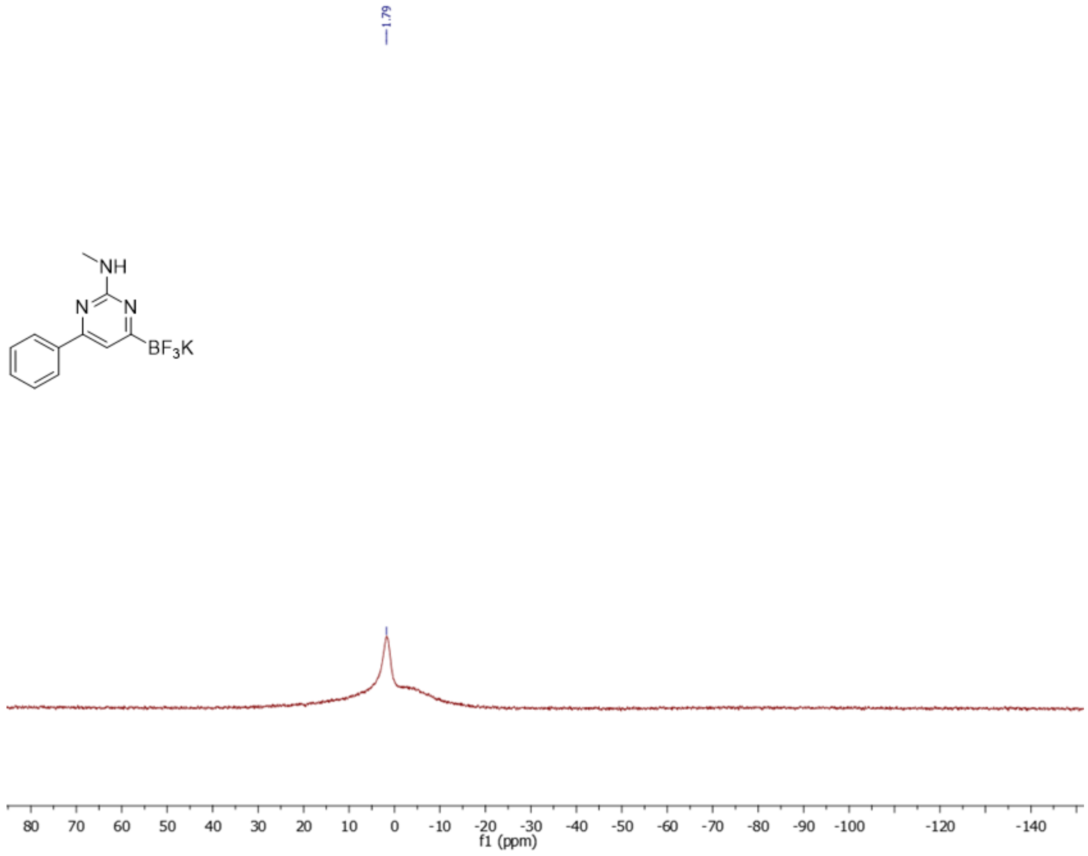
1.79
NH
N
N
BF3K
80
70
60
50
40
30
20
10
0
-10
-20
-30
-40
-50
-60
-70
-80
-90
-100
-120
-140
f1 (ppm)

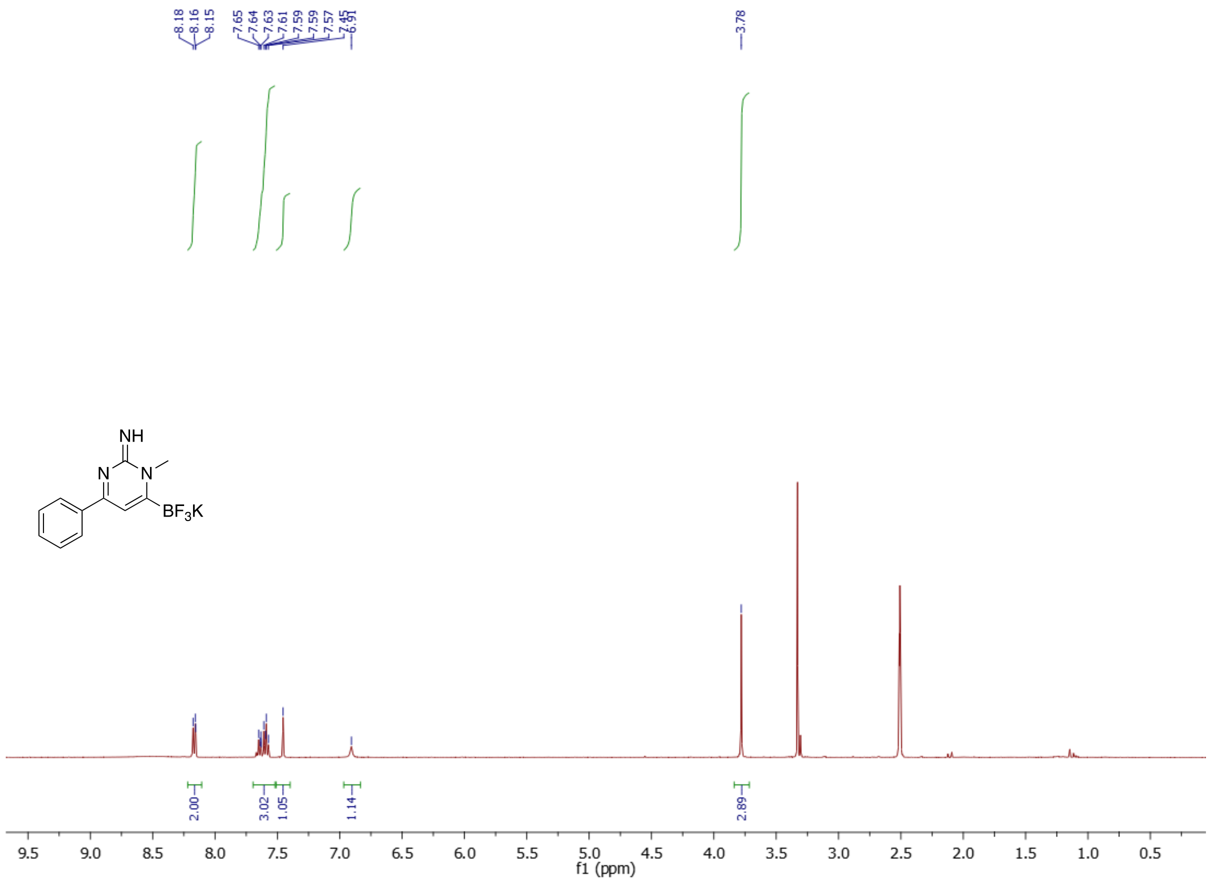
8.18
8.16
8.15
7.65
7.64
7.63
7.61
7.59
7.59
7.57
7.45
6.91
3.78
NH
N
N
BF3K
2.00
3.02
1.05
1.14
2.89
9.5
9.0
8.5
8.0
7.5
7.0
6.5
6.0
5.5
5.0
4.5
4.0
3.5
3.0
2.5
2.0
1.5
1.0
0.5
f1 (ppm)

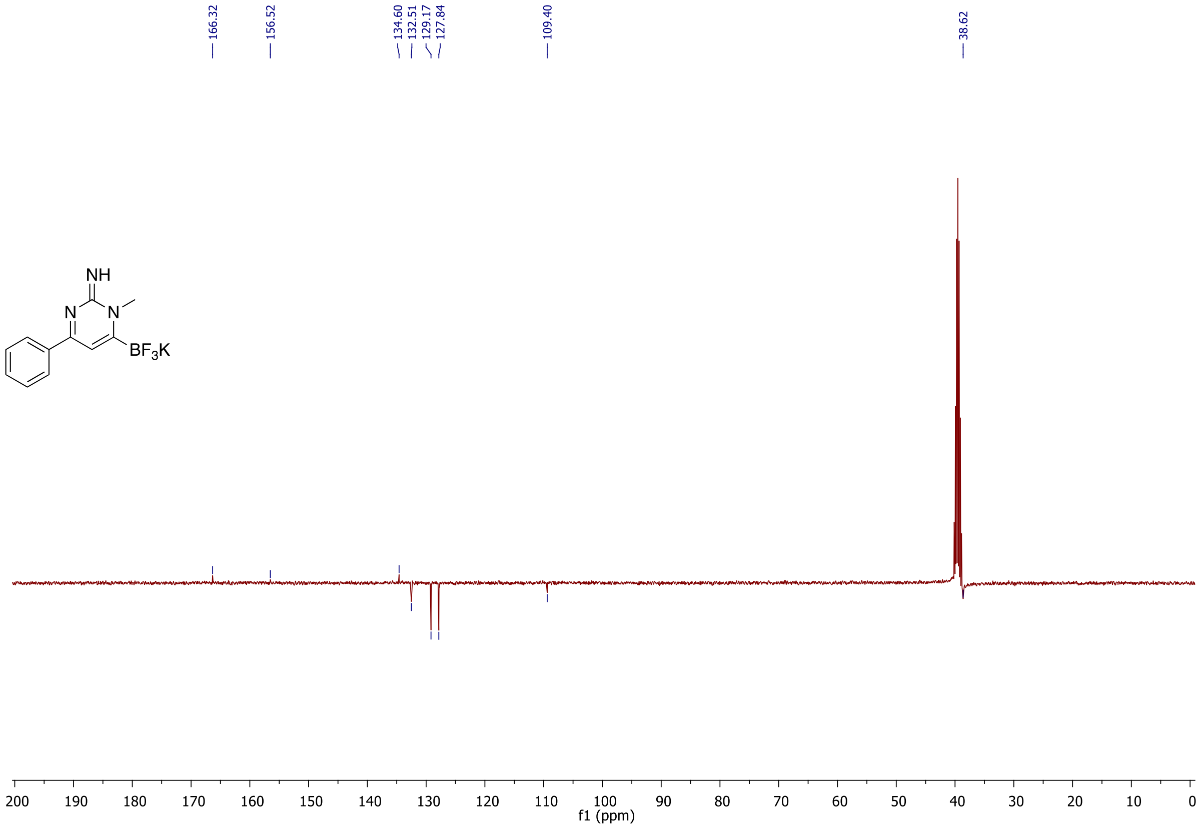
166.32
156.52
134.60
132.51
129.17
127.84
109.40
38.62
NH
N
N
BF3K
200
190
180
170
160
150
140
130
120
110
100
90
80
70
60
50
40
30
20
10
0
f1 (ppm)

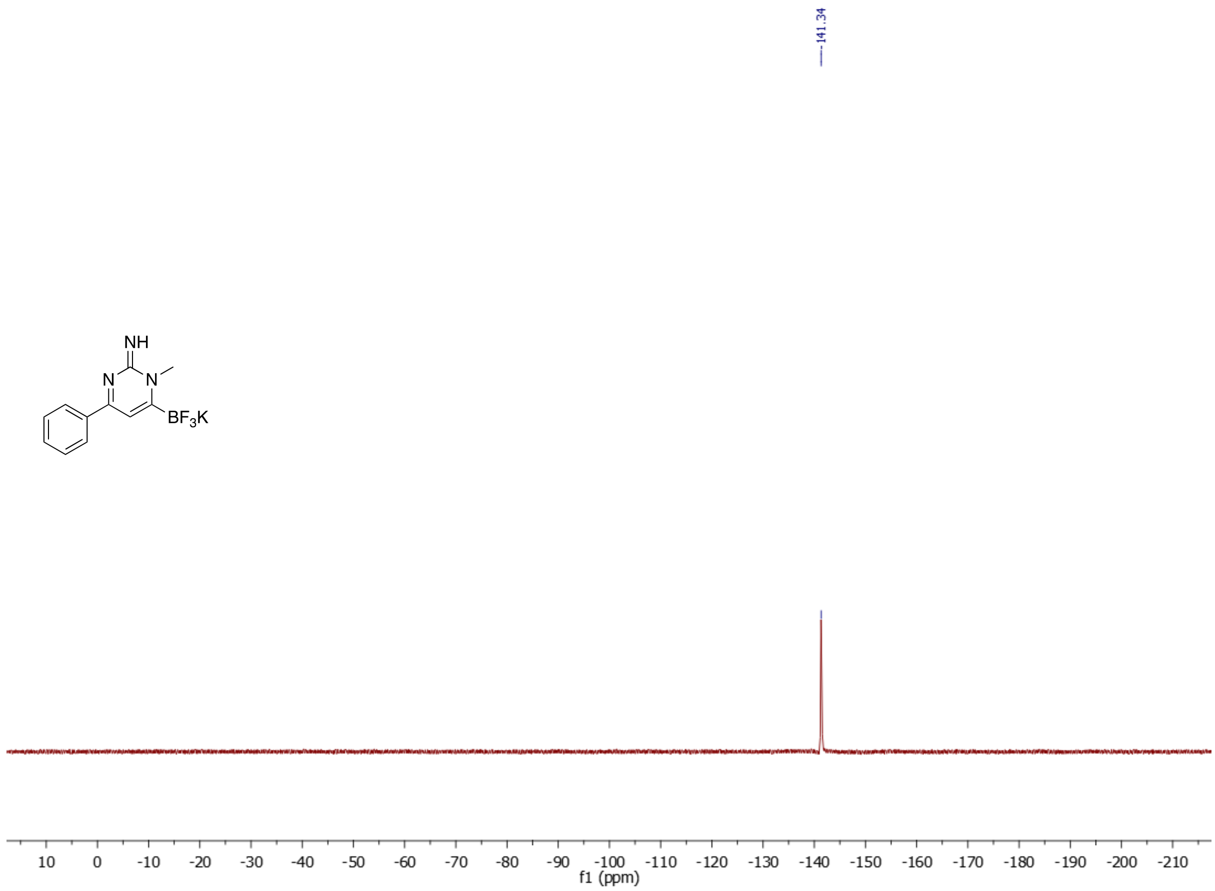
-141.34
NH
N
N
BF3K
10 0 -10 -20 -30 -40 -50 -60 -70 -80 -90 -100 -110 -120 -130 -140 -150 -160 -170 -180 -190 -200 -210
f1 (ppm)

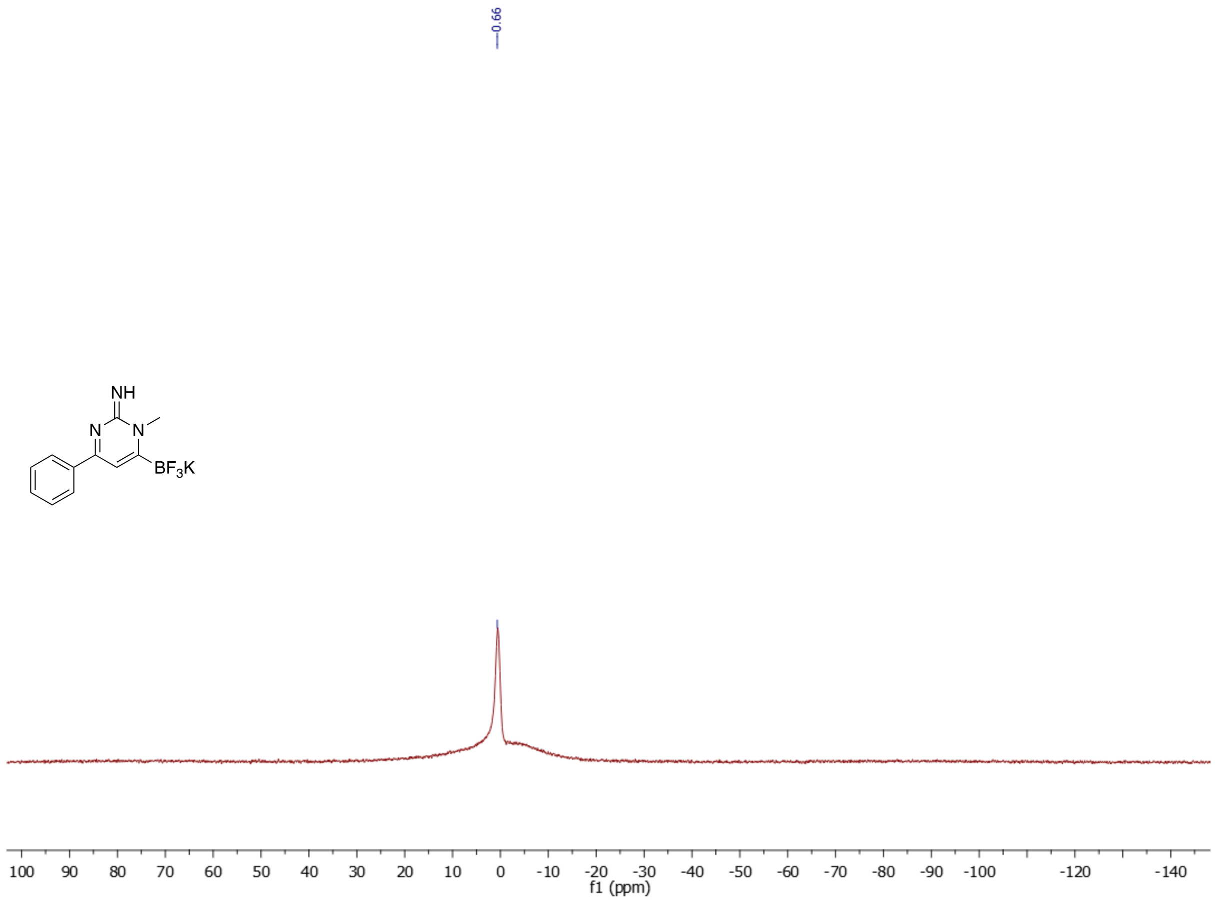
-0.66
NH
N
N
BF3K
100 90 80 70 60 50 40 30 20 10 0 -10 -20 -30 -40 -50 -60 -70 -80 -90 -100 -120 -140
f1 (ppm)

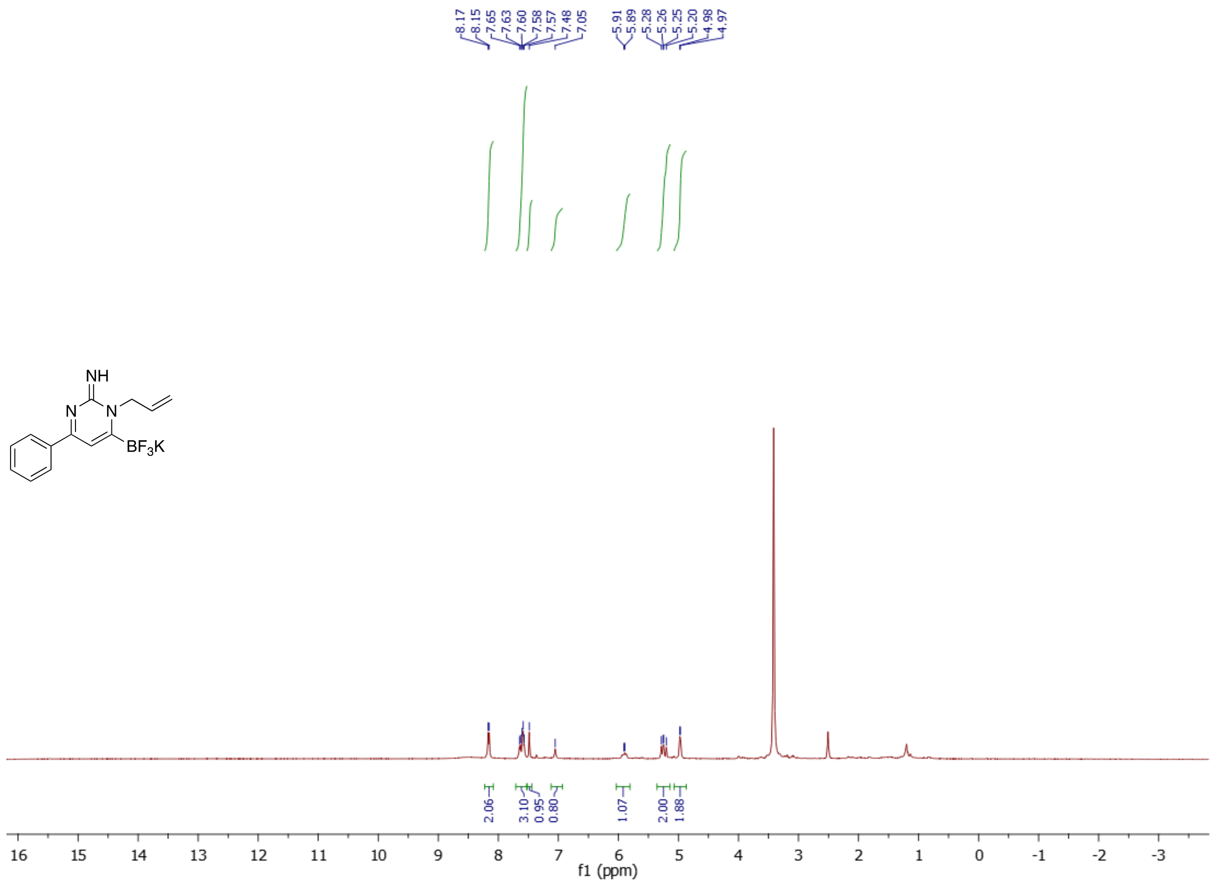
8.17
8.15
7.65
7.63
7.60
7.58
7.57
7.48
7.05
5.91
5.89
5.28
5.26
5.25
5.20
4.98
4.97
NH
N
N
BF3K
2.06
3.10
0.95
0.80
1.07
2.00
1.88
f1 (ppm)

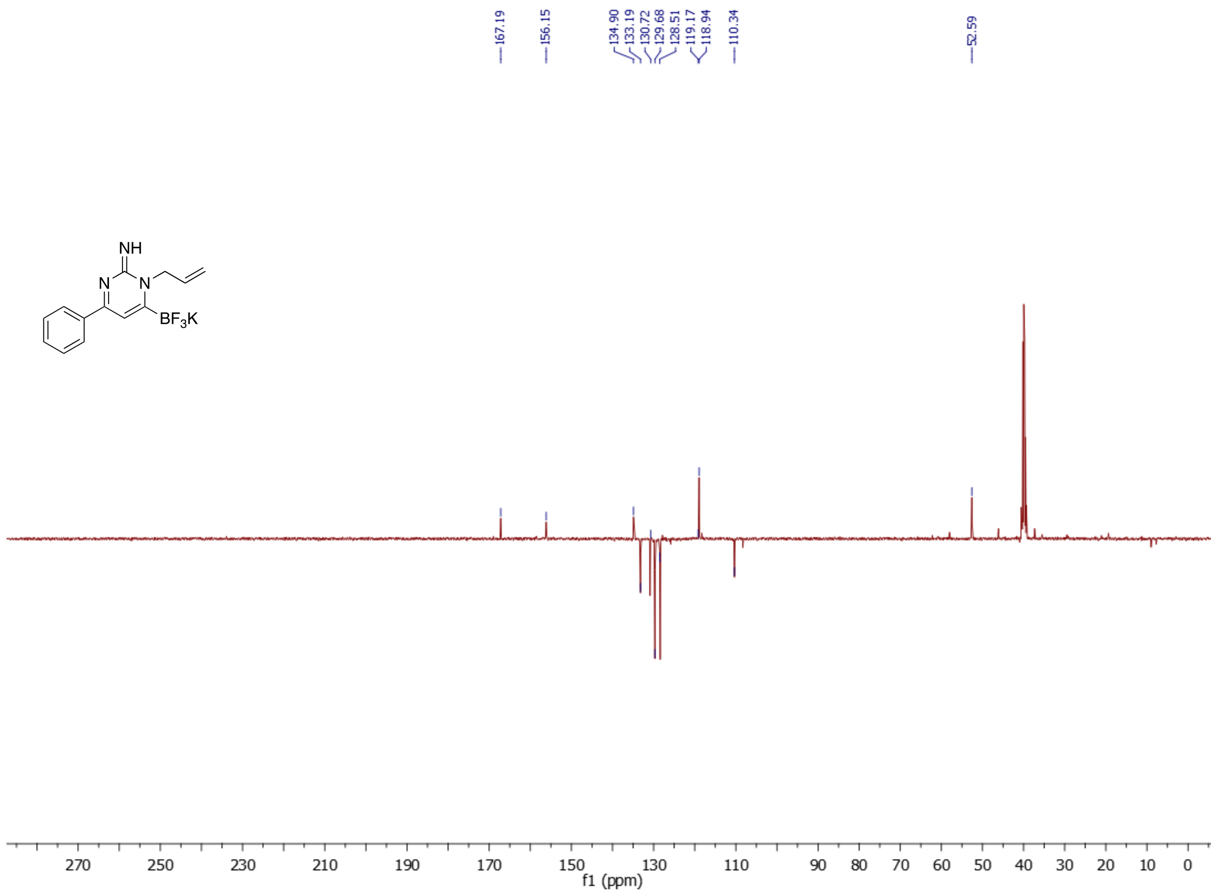
167.19
156.15
134.90
133.19
130.72
129.68
128.51
119.17
118.94
110.34
52.59
NH
N
N
BF3K
f1 (ppm)

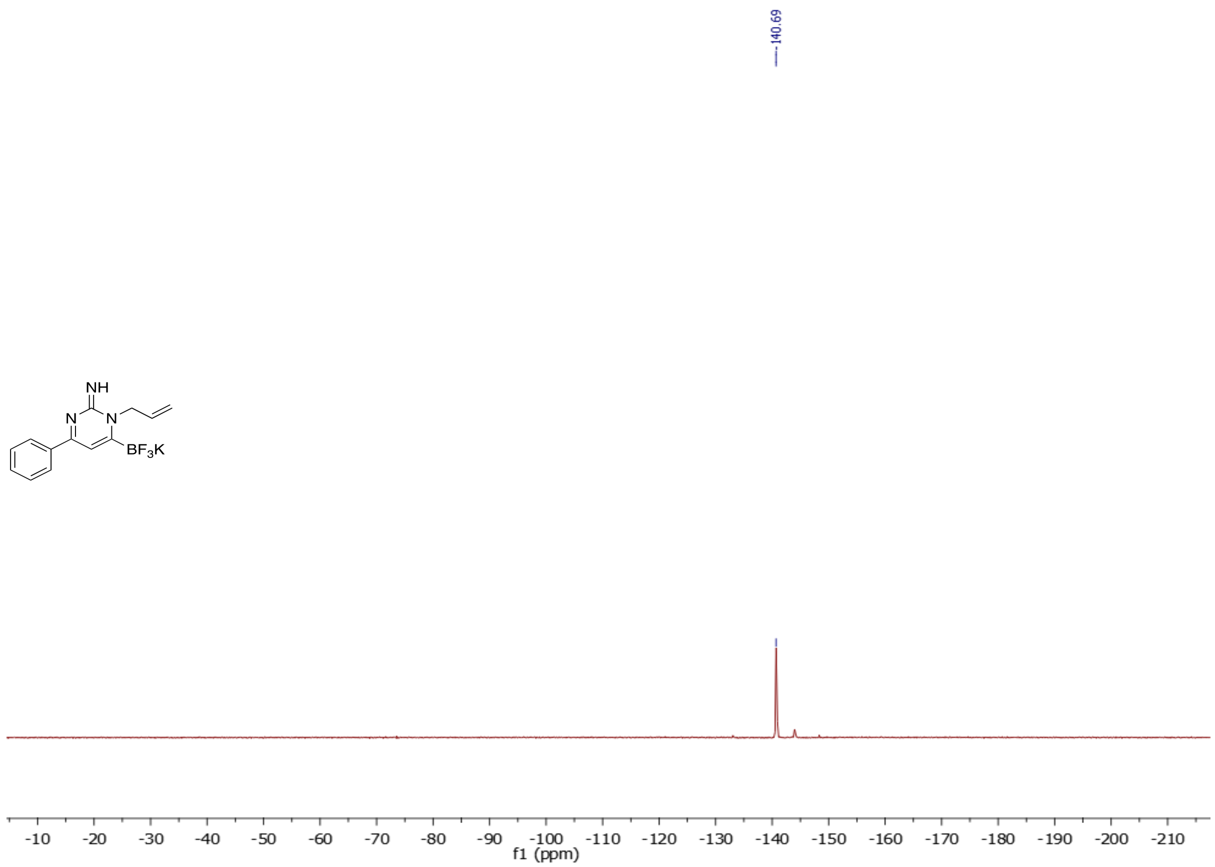
-140.69
NH
N
N
BF3K
-10 -20 -30 -40 -50 -60 -70 -80 -90 -100 -110 -120 -130 -140 -150 -160 -170 -180 -190 -200 -210
f1 (ppm)

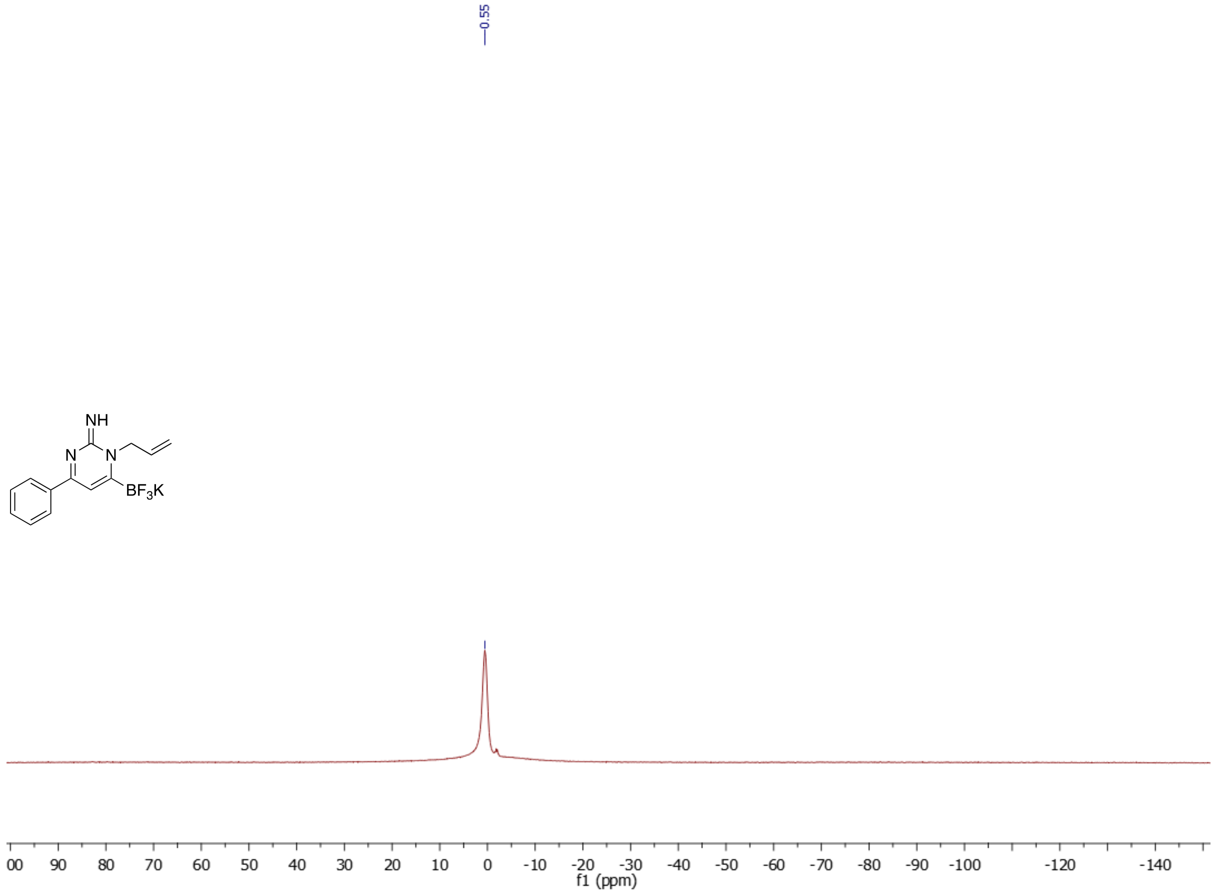
0.55
NH
N
N
BF3K
00 90 80 70 60 50 40 30 20 10 0 -10 -20 -30 -40 -50 -60 -70 -80 -90 -100 -120 -140
f1 (ppm)

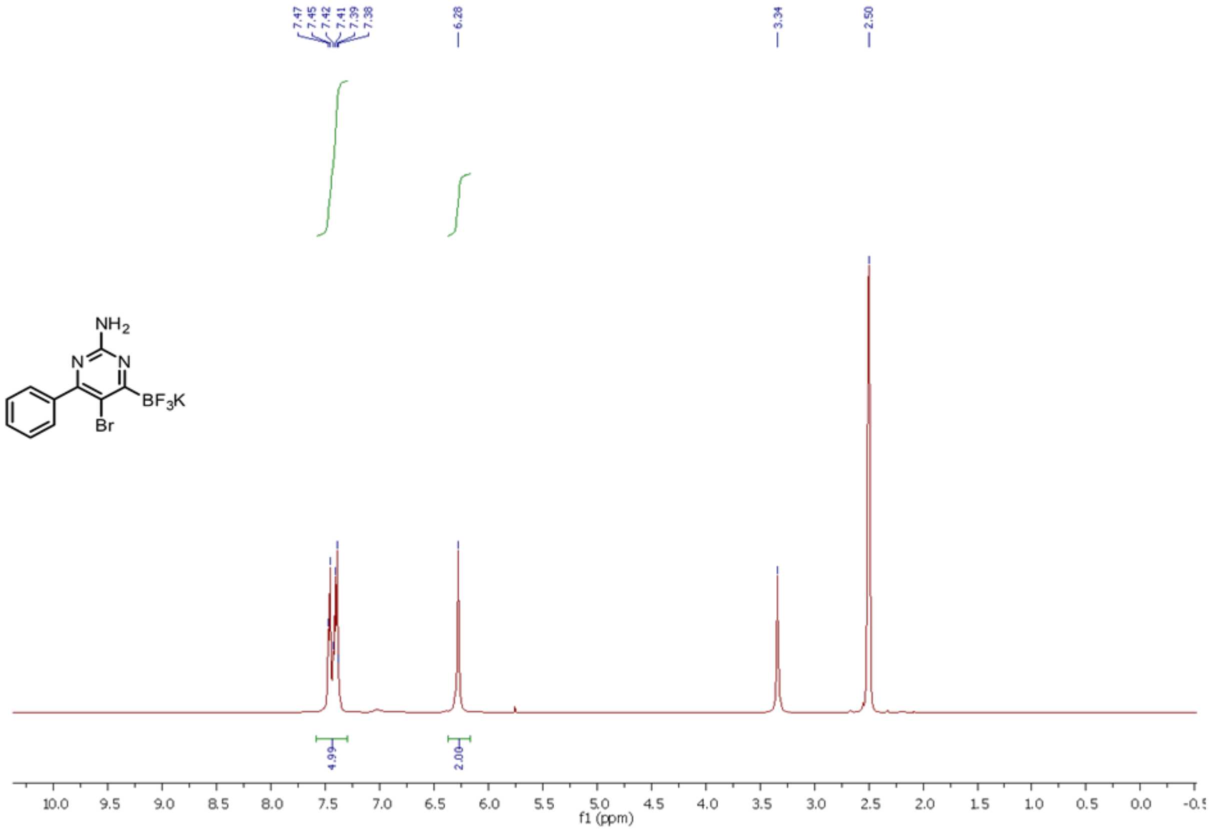
7.47
7.45
7.42
7.41
7.39
7.38
6.28
3.34
2.50
NH2
N
N
BF3K
Br
4.99
2.00
10.0 9.5 9.0 8.5 8.0 7.5 7.0 6.5 6.0 5.5 5.0 4.5 4.0 3.5 3.0 2.5 2.0 1.5 1.0 0.5 0.0
f1 (ppm)

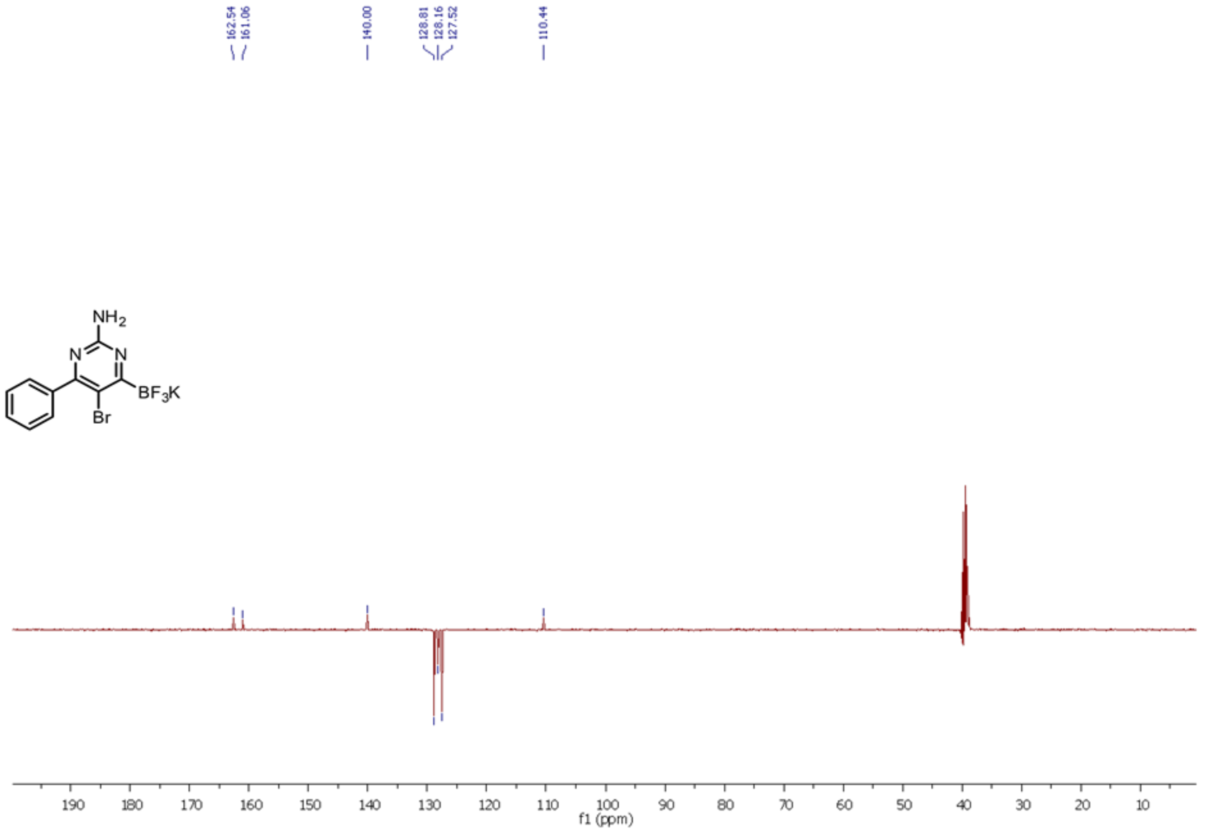
162.54
161.06
140.00
128.81
128.16
127.52
110.44
NH2
N
N
BF3K
Br
190 180 170 160 150 140 130 120 110 100 90 80 70 60 50 40 30 20 10
f1 (ppm)

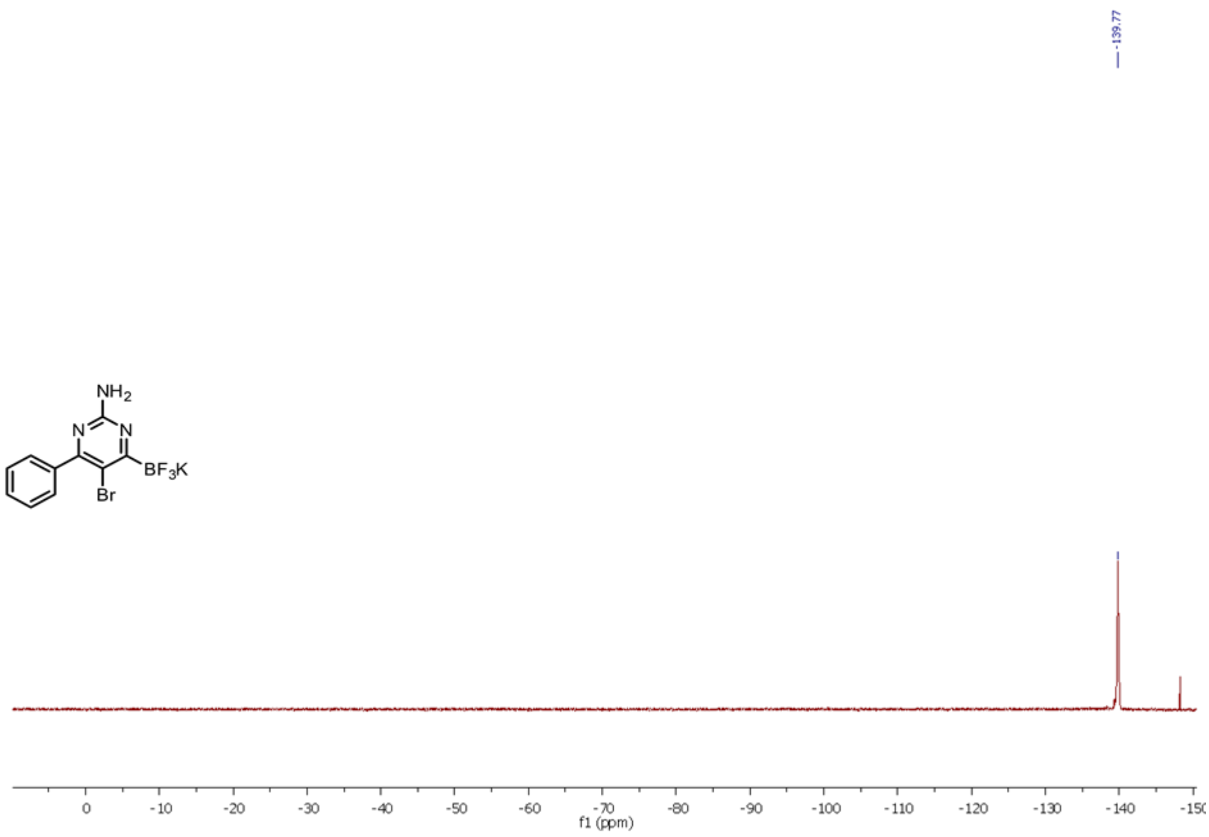
-139.77
NH2
N
N
BF3K
Br
0
-10
-20
-30
-40
-50
-60
-70
-80
-90
-100
-110
-120
-130
-140
f1 (ppm)

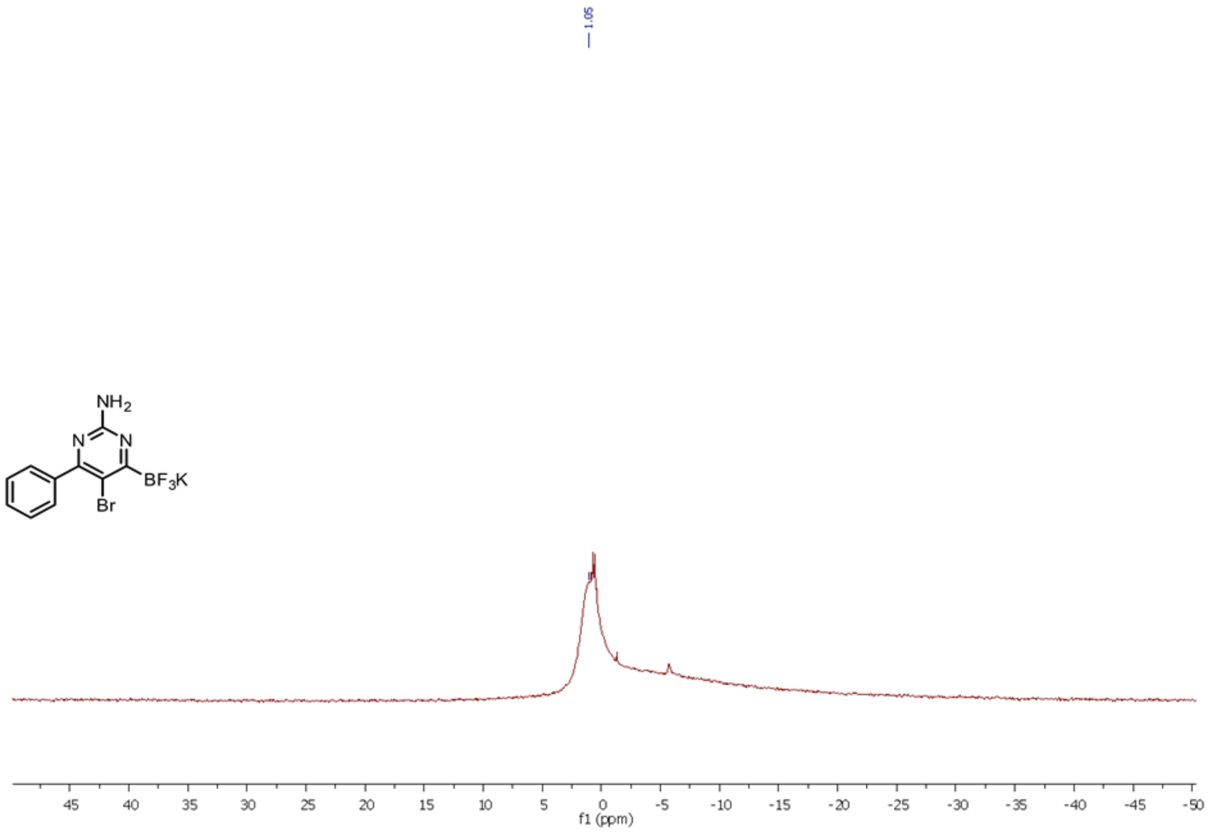
1.05
NH2
N
N
BF3K
Br
45
40
35
30
25
20
15
10
5
0
-5
-10
-15
-20
-25
-30
-35
-40
-45
-50
f1 (ppm)

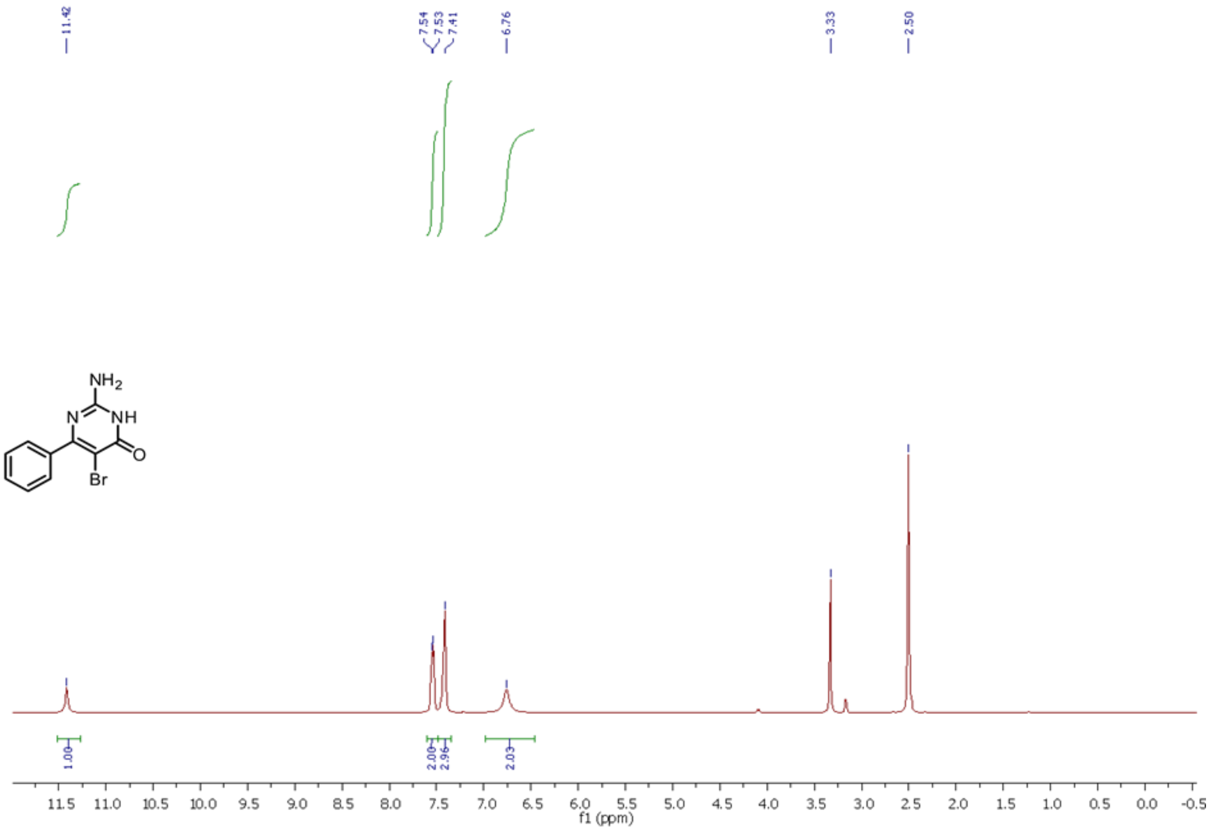
11.42
7.54
7.53
7.41
6.76
3.33
2.50
NH2
N
NH
O
Br
1.00
2.00
2.96
2.03
11.5 11.0 10.5 10.0 9.5 9.0 8.5 8.0 7.5 7.0 6.5 6.0 5.5 5.0 4.5 4.0 3.5 3.0 2.5 2.0 1.5 1.0 0.5 0.0 -0.5
f1 (ppm)

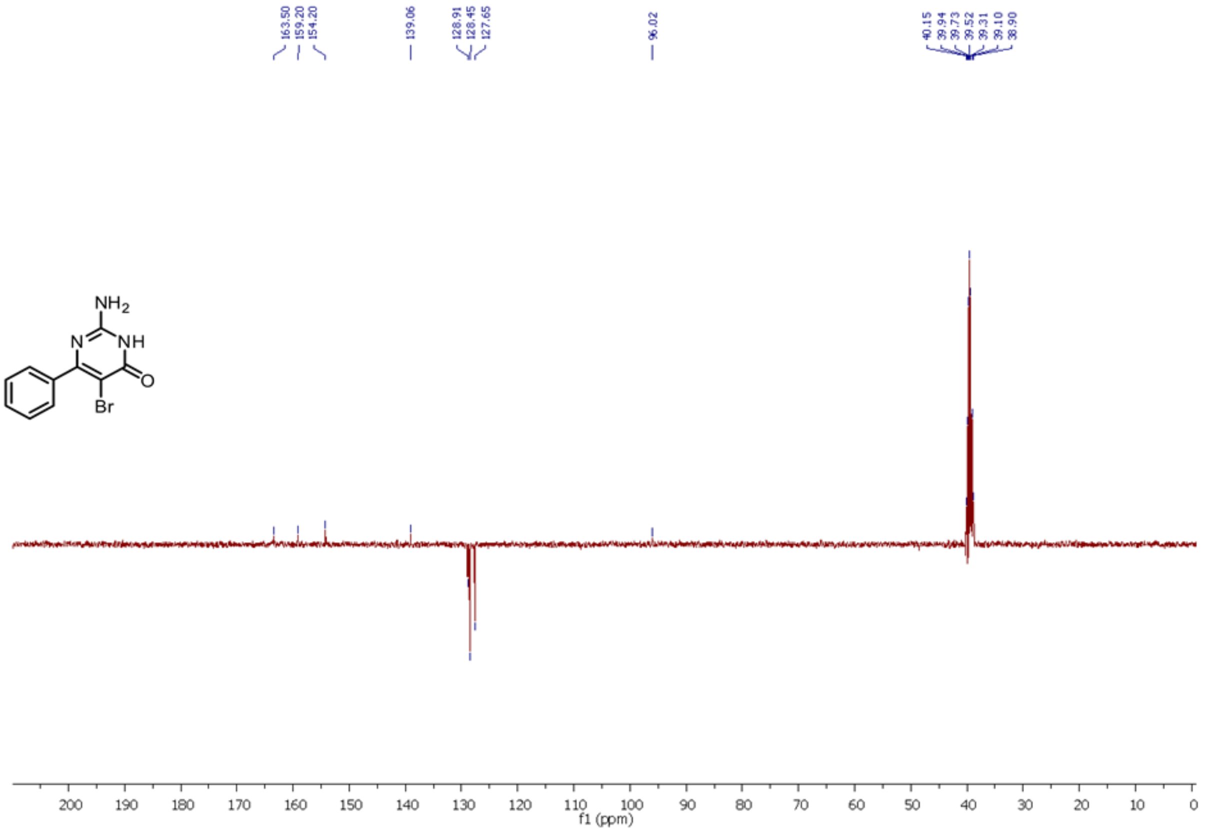
163.50
159.20
154.20
139.06
128.91
128.45
127.65
96.02
40.15
39.94
39.73
39.52
39.31
39.10
38.90
NH2
N
NH
O
Br
200 190 180 170 160 150 140 130 120 110 100 90 80 70 60 50 40 30 20 10 0
f1 (ppm)

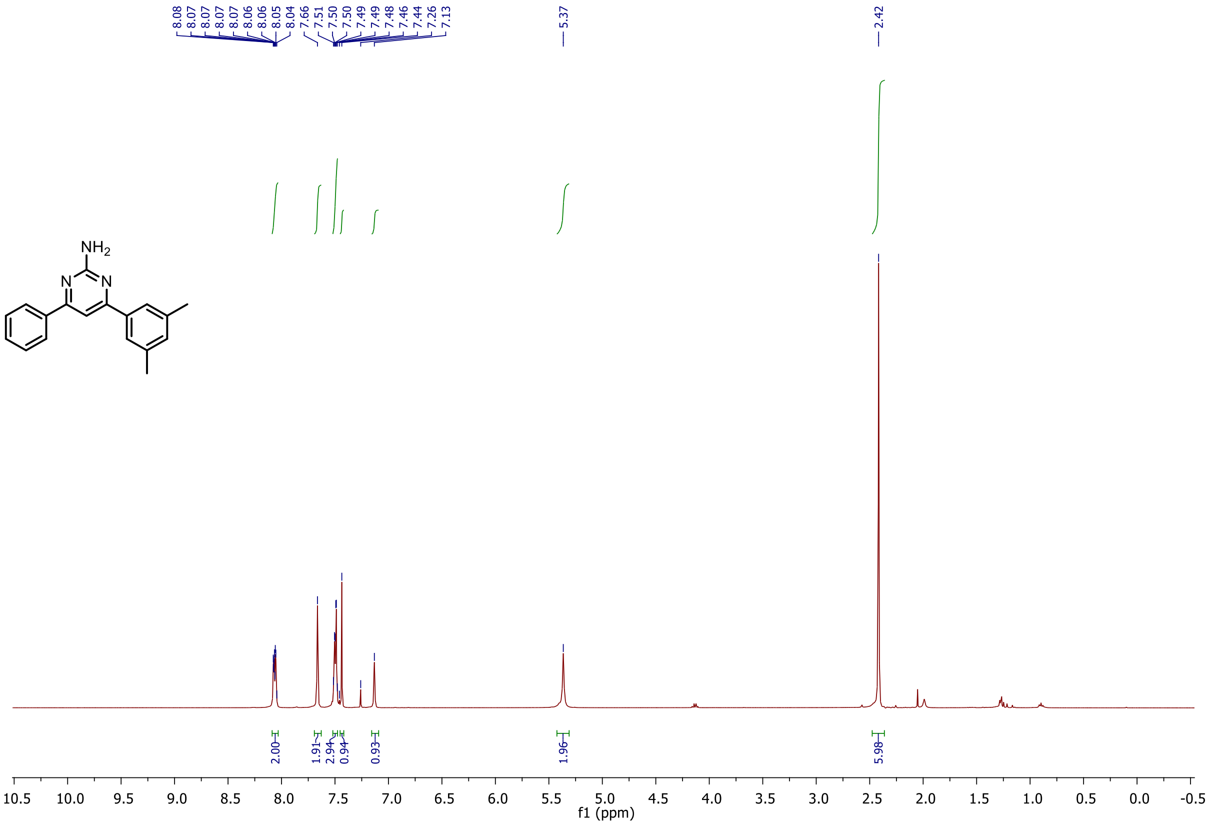
8.08
8.07
8.07
8.07
8.07
8.06
8.06
8.05
8.04
7.66
7.51
7.50
7.50
7.49
7.49
7.48
7.46
7.44
7.26
7.13
5.37
2.42
NH2
N
N
2.00
1.91
2.94
0.94
0.93
1.96
5.98
f1 (ppm)

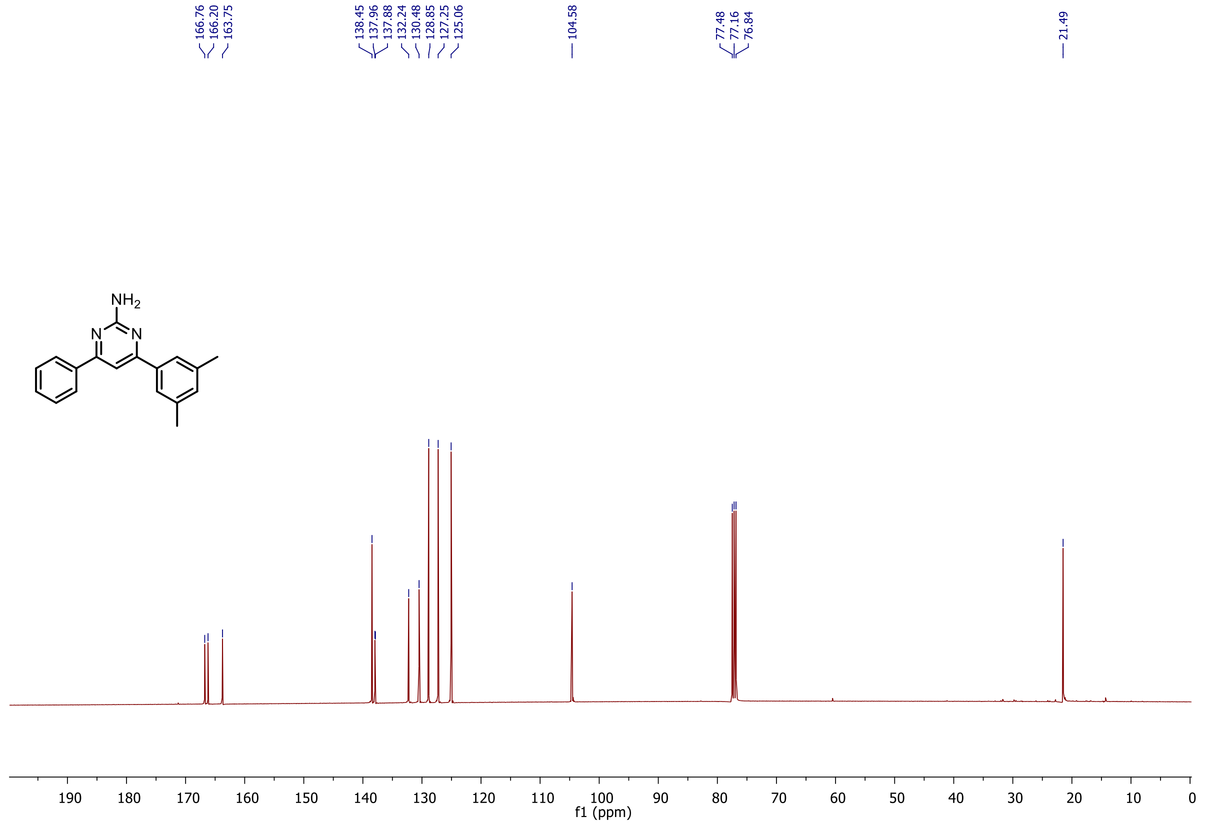
166.76
166.20
163.75
138.45
137.96
137.88
132.24
130.48
128.85
127.25
125.06
104.58
77.48
77.16
76.84
21.49
NH2
N
N
f1 (ppm)

References

[1] D. D. Perrin, W. L. F. Armarego, *Purification of laboratory materials*, 3rd ed.; Pergamon Press, Oxford, **1988**.

[2] a) P. Fricero, L. Bialy, A. W. Brown, W. Czechtizky, M. Méndez, J. P. A. Harrity, *J. Org. Chem*., **2017**, *82*, 1688-1696.; b) J. D. Kirkham, S. J. Edeson, S. Stokes, J. P. A. Harrity, *Org. Lett.*, **2012**, *14*, 5354-5357.

[3] H. Noth, B. Wrackmeyer, *Nuclear Magnetic Resonance Spectroscopy of Boron Compounds*, Springer Verlag, Berlin, **1978**, p. 6.

[4] R. Orii,N. Sakamoto, D. Fukami, S. Tsuda, M. Izumi, Y. Kajihara, R. Okamoto, *Chem. Eur. J.*, **2017**, *23*, 9253 – 9257.

[5] A. Patel, W. Lewis, M. S. Searle, M. F. G. Stevens, C. J. Moody, *Tetrahedron*, **2015**, *71*, 7339 – 7343.

[6] V. Yaziji, D. Rodriguez, H. Gutierrez-de-Teran, A. Coelho, O. Caamano, X. Garcia-Mera, J. Brea, M. I. Loza, M. I. Cadavid, E. Sotelo, *J. Med. Chem.*, **2011**, *54*, 457-471.

__X-ray crystallographic analysis for compound 2a__

Deposition number CCDC 2062198

ORTEP of 2a, thermal ellipsoids are shown at 50% probability.

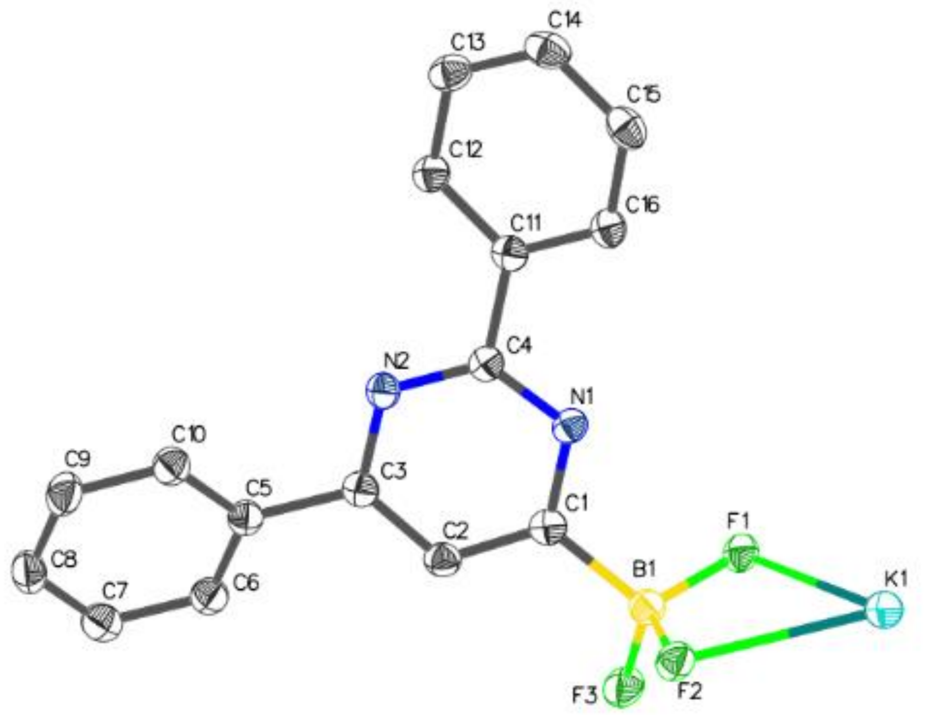

Crystal structure determination of 2a ($C_{16}H_{11}BF_3KN_2$).

Crystals of 2a were grown from a saturated acetone solution, allowing slow evaporation.

Crystal Data for $C_{16}H_{11}BF_3KN_2$ (*M* =338.18 g/mol): orthorhombic, space group Pccn (no. 56), a = 27.2800(4) Å, b = 14.9160(2) Å, c = 7.17560(10) Å, V = 2919.81(7) Å^3, Z = 8, T = 100(2) K, μ(CuKα) = 3.487 mm^{-1}, *Dcalc* = 1.539 g/cm^3, 14282 reflections measured (6.48° ≤ 2Θ ≤ 136.502°), 2674 unique (R_{int} = 0.0379, R_{sigma} = 0.0290) which were used in all calculations. The final R_1 was 0.0291 (I > 2σ(I)) and wR_2 was 0.0794 (all data).

Table 1 Crystal data and structure refinement for 2a.

Identification code	2018ncs0326s
Empirical formula	$C_{16}H_{11}BF_3KN_2$
Formula weight	338.18
Temperature/K	100(2)
Crystal system	orthorhombic
Space group	Pccn

a/Å	27.2800(4)
b/Å	14.9160(2)
c/Å	7.17560(10)
α/°	90
β/°	90
γ/°	90
Volume/Å^3	2919.81(7)
Z	8
ρ_{calc}g/cm^3	1.539
μ/mm^{-1}	3.487
F(000)	1376.0
Crystal size/mm^3	0.26 × 0.02 × 0.015
Radiation	CuKα (λ = 1.54184)
2Θ range for data collection/°	6.48 to 136.502
Index ranges	$-32 \le h \le 24$, $-17 \le k \le 17$, $-8 \le l \le 8$
Reflections collected	14282
Independent reflections	2674 [R_{int} = 0.0379, R_{sigma} = 0.0290]
Data/restraints/parameters	2674/0/208
Goodness-of-fit on F^2	1.023
Final R indexes [I>=2σ (I)]	R_1 = 0.0291, wR_2 = 0.0765
Final R indexes [all data]	R_1 = 0.0334, wR_2 = 0.0794
Largest diff. peak/hole / e Å^{-3}	0.34/-0.24

Table 2 Fractional Atomic Coordinates (×10^4) and Equivalent Isotropic Displacement Parameters (Å^2×10^3) for 2018ncs0326s. U_{eq} is defined as 1/3 of of the trace of the orthogonalised U_{IJ} tensor.

Atom	*x*	*y*	*z*	U(eq)
K1	8023.6(2)	3569.1(2)	6402.3(4)	20.94(11)
F1	7042.1(3)	3187.4(6)	5245.5(12)	22.6(2)
F2	7133.2(3)	4089.9(6)	7769.1(12)	25.3(2)
F3	6505.6(3)	3092.7(6)	7684.2(14)	30.0(2)
N1	6589.2(4)	4825.3(8)	4010.6(17)	19.1(3)
N2	5920.4(5)	5860.4(8)	4093.3(17)	19.3(3)
C1	6435.5(5)	4455.6(10)	5635(2)	19.6(3)
C2	6008.9(6)	4763.4(10)	6469(2)	20.9(3)
C3	5753.7(5)	5472.9(10)	5664(2)	19.4(3)
C4	6324.8(5)	5510.2(10)	3335(2)	18.3(3)
C5	5299.8(5)	5859.1(10)	6476(2)	20.0(3)
C6	4993.0(6)	5349.8(11)	7614(2)	22.4(3)
C7	4573.4(6)	5724.5(12)	8381(2)	24.9(4)
C8	4457.1(6)	6615.1(12)	8031(2)	27.0(4)
C9	4757.7(6)	7122.1(12)	6891(2)	28.1(4)
C10	5173.5(6)	6748.5(11)	6105(2)	23.9(3)

C11	6495.2(6)	5930.7(10)	1567(2)	19.6(3)
C12	6171.1(6)	6428.7(10)	470(2)	21.4(3)
C13	6335.4(6)	6869.2(11)	-1112(2)	25.5(4)
C14	6827.3(7)	6828.8(11)	-1610(2)	27.3(4)
C15	7150.7(6)	6330.8(10)	-538(2)	25.9(4)
C16	6985.3(6)	5869.4(10)	1019(2)	21.3(3)
B1	6776.6(7)	3697.0(12)	6579(2)	21.2(4)

Table 3 Anisotropic Displacement Parameters (Å^2×10^3) for 2018ncs0326s. The Anisotropic displacement factor exponent takes the form: $-2\pi^2[h^2a^{*2}U_{11}+2hka^*b^*U_{12}+\ldots]$.

Atom	U_{11}	U_{22}	U_{33}	U_{23}	U_{13}	U_{12}
K1	24.30(19)	17.04(19)	21.48(19)	-1.34(12)	-0.45(12)	-1.02(12)
F1	27.3(5)	16.9(4)	23.6(5)	-1.0(3)	-0.8(4)	4.1(3)
F2	28.1(5)	24.3(5)	23.5(5)	-3.1(4)	-5.2(4)	3.9(4)
F3	28.1(5)	25.9(5)	36.0(5)	13.4(4)	4.7(4)	2.2(4)
N1	21.9(6)	15.4(6)	20.0(6)	-0.3(5)	-1.6(5)	0.4(5)
N2	21.3(6)	16.3(6)	20.4(6)	-1.6(5)	0.3(5)	0.8(5)
C1	22.8(7)	15.6(7)	20.3(7)	-1.2(6)	-1.1(6)	-2.5(6)
C2	24.3(8)	19.1(8)	19.4(8)	1.0(6)	1.0(6)	-0.3(6)
C3	22.3(7)	16.4(7)	19.5(7)	-2.1(6)	-0.2(6)	-1.5(6)
C4	20.7(7)	14.7(7)	19.5(7)	-2.1(6)	-1.8(6)	-1.1(6)
C5	19.7(7)	20.9(8)	19.4(7)	-2.3(6)	-2.0(6)	0.4(6)
C6	24.0(8)	21.9(8)	21.4(7)	-1.7(6)	-1.7(6)	0.5(6)
C7	22.4(8)	30.2(9)	22.2(8)	-1.7(7)	-1.0(6)	-3.8(6)
C8	20.2(7)	33.9(9)	26.7(8)	-7.3(7)	-1.2(7)	4.6(7)
C9	29.6(8)	23.4(8)	31.4(8)	-1.4(7)	-1.5(7)	7.8(7)
C10	25.0(8)	21.8(8)	24.9(8)	1.1(6)	1.3(6)	1.3(6)
C11	25.4(8)	13.8(7)	19.4(7)	-2.7(6)	0.6(6)	-0.8(6)
C12	24.6(8)	17.0(7)	22.7(8)	-0.2(6)	-0.3(6)	-0.1(6)
C13	33.6(9)	19.3(8)	23.6(8)	1.6(6)	-2.9(7)	-0.1(6)
C14	39.6(9)	19.5(8)	22.9(8)	1.2(6)	6.1(7)	-4.4(7)
C15	27.7(8)	22.0(8)	27.9(9)	-4.2(6)	6.9(7)	-2.7(6)
C16	23.6(8)	16.9(8)	23.5(8)	-2.7(6)	0.5(6)	0.4(6)
B1	23.6(9)	19.1(9)	20.8(9)	1.2(7)	1.5(7)	0.6(7)

Table 4 Bond Lengths for 2018ncs0326s.

Atom	Atom	Length/Å	Atom	Atom	Length/Å
K1	F1[1]	2.8216(9)	N2	C3	1.3455(19)
K1	F1	2.8603(9)	N2	C4	1.3364(19)
K1	F1[2]	2.7542(9)	C1	C2	1.387(2)
K1	F2	2.7323(9)	C1	B1	1.614(2)

K1	F2^{3}	2.7537(9)	C2	C3	1.392(2)
K1	F3^{3}	3.0451(11)	C3	C5	1.485(2)
K1	F3^{2}	2.9394(10)	C4	C11	1.490(2)
K1	N1^{1}	2.8512(13)	C5	C6	1.395(2)
K1	C16^{1}	3.4422(16)	C5	C10	1.396(2)
K1	B1	3.4094(18)	C6	C7	1.388(2)
K1	B1^{2}	3.4261(18)	C7	C8	1.389(2)
K1	B1^{3}	3.5088(17)	C8	C9	1.383(2)
F1	K1^{3}	2.8216(9)	C9	C10	1.384(2)
F1	K1^{2}	2.7542(9)	C11	C12	1.398(2)
F1	B1	1.4206(19)	C11	C16	1.397(2)
F2	K1^{1}	2.7537(9)	C12	C13	1.386(2)
F2	B1	1.421(2)	C13	C14	1.390(2)
F3	K1^{1}	3.0451(11)	C14	C15	1.386(2)
F3	K1^{2}	2.9394(10)	C15	C16	1.388(2)
F3	B1	1.4101(19)	C16	K1^{3}	3.4422(16)
N1	K1^{3}	2.8512(13)	B1	K1^{2}	3.4261(18)
N1	C1	1.3556(19)	B1	K1^{1}	3.5088(17)
N1	C4	1.3413(19)			

^{1}3/2-X,+Y,1/2+Z; ^{2}3/2-X,1/2-Y,+Z; ^{3}3/2-X,+Y,-1/2+Z

Table 5 Bond Angles for 2018ncs0326s.

Atom	Atom	Atom	Angle/˚	Atom	Atom	Atom	Angle/˚
F1^{1}	K1	F1^{2}	95.65(2)	K1^{1}	F1	K1^{3}	118.84(3)
F1^{1}	K1	F1	70.26(3)	K1^{1}	F1	K1	99.37(3)
F1^{2}	K1	F1	100.60(2)	K1^{3}	F1	K1	107.64(3)
F1^{1}	K1	F3^{1}	47.20(2)	B1	F1	K1	100.09(8)
F1^{2}	K1	F3^{3}	146.72(3)	B1	F1	K1^{1}	105.83(8)
F1^{2}	K1	F3^{1}	63.37(3)	B1	F1	K1^{3}	121.20(8)
F1	K1	F3^{3}	95.40(3)	K1	F2	K1^{2}	113.44(3)
F1^{1}	K1	F3^{3}	62.71(3)	B1	F2	K1^{2}	110.26(8)
F1	K1	F3^{1}	109.39(3)	B1	F2	K1	106.00(8)
F1^{1}	K1	N1^{2}	147.90(3)	K1^{1}	F3	K1^{2}	106.66(3)
F1^{2}	K1	N1^{2}	60.96(3)	B1	F3	K1^{2}	97.03(9)
F1^{1}	K1	C16^{2}	157.47(3)	B1	F3	K1^{1}	97.70(8)
F1^{2}	K1	C16^{2}	106.19(3)	C1	N1	K1^{3}	114.30(9)
F1	K1	C16^{2}	99.71(3)	C4	N1	K1^{3}	117.55(9)
F1^{1}	K1	B1	89.97(4)	C4	N1	C1	117.01(12)
F1^{1}	K1	B1^{1}	23.51(3)	C4	N2	C3	116.87(13)
F1^{2}	K1	B1^{3}	170.03(4)	N1	C1	C2	119.74(13)
F1	K1	B1	24.22(3)	N1	C1	B1	117.89(13)
F1^{1}	K1	B1^{3}	76.38(3)	C2	C1	B1	122.30(13)
F1^{2}	K1	B1^{1}	76.99(3)	C1	C2	C3	119.45(14)

F1^2	K1	B1	84.93(3)	N2	C3	C2	120.35(13)
F1	K1	B1^3	82.53(3)	N2	C3	C5	116.35(13)
F1	K1	B1^1	87.90(4)	C2	C3	C5	123.28(14)
F2	K1	F1^1	108.71(3)	N1	C4	C11	117.43(13)
F2	K1	F1^2	69.51(3)	N2	C4	N1	126.48(14)
F2^3	K1	F1^1	88.45(3)	N2	C4	C11	116.09(13)
F2^3	K1	F1^2	166.50(3)	C6	C5	C3	121.27(14)
F2^3	K1	F1	68.66(3)	C6	C5	C10	118.76(14)
F2	K1	F1	47.83(3)	C10	C5	C3	119.97(14)
F2	K1	F2^3	96.98(2)	C7	C6	C5	120.52(15)
F2^3	K1	F3^3	45.72(3)	C6	C7	C8	120.14(15)
F2	K1	F3^3	139.10(3)	C9	C8	C7	119.64(15)
F2^3	K1	F3^1	127.02(3)	C8	C9	C10	120.44(15)
F2	K1	F3^1	121.06(3)	C9	C10	C5	120.48(15)
F2	K1	N1^2	84.66(3)	C12	C11	C4	120.39(14)
F2^3	K1	N1^2	119.58(3)	C16	C11	C4	120.74(14)
F2^3	K1	C16^2	69.02(3)	C16	C11	C12	118.80(14)
F2	K1	C16^2	74.87(3)	C13	C12	C11	120.57(15)
F2	K1	B1^1	114.12(4)	C12	C13	C14	120.15(15)
F2	K1	B1^3	118.47(4)	C15	C14	C13	119.68(15)
F2^3	K1	B1^1	109.76(4)	C14	C15	C16	120.35(15)
F2	K1	B1	23.62(3)	C11	C16	K1^3	92.06(9)
F2^3	K1	B1^3	22.33(3)	C15	C16	K1^3	124.13(10)
F2^3	K1	B1	82.21(3)	C15	C16	C11	120.38(15)
F3^1	K1	F3^3	83.86(2)	K1	B1	K1^2	83.03(4)
F3^3	K1	C16^2	99.53(3)	K1^1	B1	K1^2	87.60(4)
F3^1	K1	C16^2	150.30(3)	K1	B1	K1^1	77.57(4)
F3^3	K1	B1	117.81(4)	F1	B1	K1	55.69(7)
F3^1	K1	B1^3	106.66(4)	F1	B1	K1^1	50.66(7)
F3^1	K1	B1	118.13(4)	F1	B1	K1^2	123.79(10)
F3^1	K1	B1^1	24.07(3)	F1	B1	F2	106.05(13)
F3^3	K1	B1^3	23.50(3)	F1	B1	C1	112.72(12)
F3^3	K1	B1^1	74.64(3)	F2	B1	K1	50.39(7)
N1^2	K1	F1	131.92(3)	F2	B1	K1^1	108.68(9)
N1^2	K1	F3^3	124.90(3)	F2	B1	K1^2	47.41(7)
N1^2	K1	F3^1	100.77(3)	F2	B1	C1	110.97(13)
N1^2	K1	C16^2	53.13(4)	F3	B1	K1^1	58.23(7)
N1^2	K1	B1	107.96(4)	F3	B1	K1	120.55(10)
N1^2	K1	B1^3	123.66(4)	F3	B1	K1^2	59.47(7)
N1^2	K1	B1^1	124.41(4)	F3	B1	F1	107.71(13)
C16^2	K1	B1^3	82.42(4)	F3	B1	F2	106.54(12)
B1^1	K1	C16^2	170.92(4)	F3	B1	C1	112.46(13)
B1	K1	C16^2	86.57(4)	C1	B1	K1^2	122.81(10)
B1	K1	B1^1	102.27(4)	C1	B1	K1^1	140.15(10)
B1^1	K1	B1^3	93.75(4)	C1	B1	K1	126.78(10)
B1	K1	B1^3	100.87(4)				

[1]3/2-X,1/2-Y,+Z; [2]3/2-X,+Y,1/2+Z; [3]3/2-X,+Y,-1/2+Z

Table 6 Hydrogen Atom Coordinates (Å×10^4) and Isotropic Displacement Parameters (Å^2×10^3) for 2.

Atom	*x*	*y*	*z*	U(eq)
H2	5892	4492	7582	25
H6	5072	4741	7867	27
H7	4365	5371	9146	30
H8	4173	6875	8571	32
H9	4678	7731	6646	34
H10	5374	7100	5308	29
H12	5835	6466	812	26
H13	6111	7199	-1857	31
H14	6941	7141	-2679	33
H15	7487	6305	-871	31
H16	7207	5510	1716	26

X-ray crystallographic analysis for compound 6

Deposition number CCDC 2062205

ORTEP of 6, thermal ellipsoids are shown at 50% probability

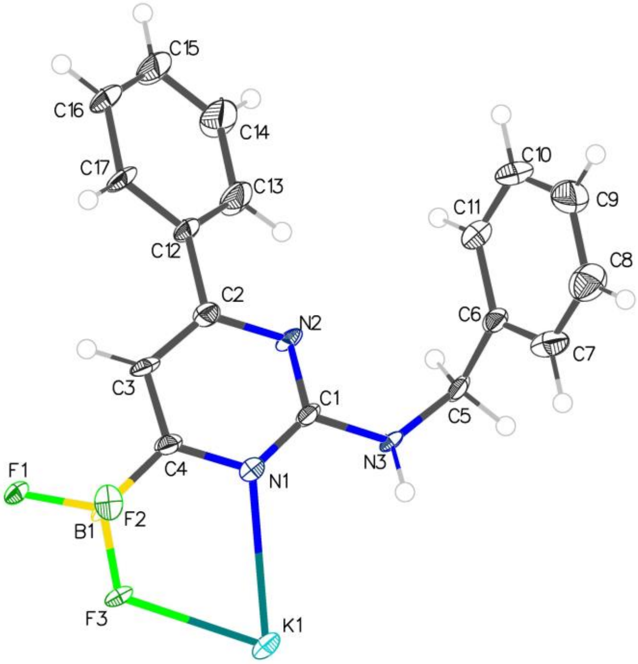

Crystal structure determination of compound 6

Crystals of 6 were grown from a saturated acetone solution, allowing slow evaporation.

Crystal Data for $C_{17}H_{14}BF_3KN_3$ (*M* =367.22 g/mol): monoclinic, space group P2$_1$/c (no. 14), *a* = 18.251(5) Å, *b* = 10.005(3) Å, *c* = 9.1105(18) Å, *β* = 94.150(16)°, *V* = 1659.2(7) Å^3, *Z* = 4, *T* = 100 K, μ(MoKα) = 0.355 mm^{-1}, *Dcalc* = 1.470 g/cm^3, 4270 reflections measured (4.476° ≤ 2Θ ≤ 46.598°), 2330 unique (R_{int} = 0.0897, R_{sigma} = 0.1525) which were used in all calculations. The final R_1 was 0.0678 (I > 2σ(I)) and wR_2 was 0.1452 (all data).

Table 1 Crystal data and structure refinement for ojH388k.

Identification code	ojH388k
Empirical formula	$C_{17}H_{14}BF_3KN_3$
Formula weight	367.22
Temperature/K	100
Crystal system	monoclinic
Space group	P2$_1$/c
a/Å	18.251(5)
b/Å	10.005(3)

c/Å	9.1105(18)
α/°	90
β/°	94.150(16)
γ/°	90
Volume/Å^3	1659.2(7)
Z	4
ρ_{calc}g/cm^3	1.470
μ/mm^{-1}	0.355
F(000)	752.0
Crystal size/mm^3	0.61 × 0.21 × 0.025
Radiation	MoKα (λ = 0.71073)
2Θ range for data collection/°	4.476 to 46.598
Index ranges	-14 ≤ h ≤ 20, -9 ≤ k ≤ 11, -10 ≤ l ≤ 10
Reflections collected	4270
Independent reflections	2330 [R_{int} = 0.0897, R_{sigma} = 0.1525]
Data/restraints/parameters	2330/199/226
Goodness-of-fit on F^2	1.021
Final R indexes [I>=2σ (I)]	R_1 = 0.0678, wR_2 = 0.1209
Final R indexes [all data]	R_1 = 0.1523, wR_2 = 0.1452
Largest diff. peak/hole / e Å^{-3}	0.44/-0.44

Table 2 Fractional Atomic Coordinates (×10^4) and Equivalent Isotropic Displacement Parameters (Å^2×10^3) for ojH388k. U_{eq} is defined as 1/3 of of the trace of the orthogonalised U_{IJ} tensor.

Atom	*x*	*y*	*z*	U(eq)
K1	4804.9(7)	2868.2(15)	5155.8(15)	20.5(4)
F1	5722.0(16)	4324(4)	727(3)	23.6(10)
N1	6075(3)	4493(5)	4782(5)	17.9(13)
C1	6653(3)	4283(6)	5805(6)	16.6(15)
B1	5562(4)	4735(8)	2153(8)	16.5(16)
F2	5350.8(17)	6104(4)	2087(4)	24.4(9)
N2	7360(3)	4100(6)	5533(5)	21.3(14)
C2	7517(3)	4156(7)	4114(7)	20.5(16)
F3	4923.1(17)	3994(4)	2490(3)	21.8(9)
N3	6485(2)	4228(5)	7237(5)	20.0(14)
C3	6962(3)	4334(6)	3002(7)	17.6(15)
C4	6240(3)	4500(6)	3351(6)	16.8(14)
C5	7052(3)	4058(7)	8443(6)	21.8(16)
C6	7437(3)	5366(7)	8943(6)	20.4(15)

Table 2 Fractional Atomic Coordinates (×10^4) and Equivalent Isotropic Displacement Parameters (Å²×10³) for ojH388k. U_{eq} is defined as 1/3 of of the trace of the orthogonalised U_{IJ} tensor.

Atom	*x*	*y*	*z*	U(eq)
C7	7084(4)	6267(7)	9760(7)	28.0(17)
C8	7439(4)	7439(8)	10294(8)	37.0(19)
C9	8161(4)	7658(9)	9991(8)	51(2)
C10	8512(4)	6751(10)	9158(8)	52(2)
C11	8157(4)	5597(9)	8628(7)	42(2)
C12	8301(3)	3998(8)	3825(7)	28.5(18)
C13	8770(3)	3262(8)	4785(7)	37(2)
C14	9508(4)	3134(10)	4535(8)	58(3)
C15	9791(4)	3781(10)	3362(8)	55(3)
C16	9328(3)	4536(9)	2420(8)	45(2)
C17	8585(3)	4647(8)	2643(7)	32(2)

Table 3 Anisotropic Displacement Parameters (Å²×10³) for ojH388k. The Anisotropic displacement factor exponent takes the form: $-2\pi^2[h^2a^{*2}U_{11}+2hka^*b^*U_{12}+\ldots]$.

Atom	U_{11}	U_{22}	U_{33}	U_{23}	U_{13}	U_{12}
K1	18.4(8)	25.5(9)	19.0(7)	1.5(8)	9.9(6)	-0.1(8)
F1	12.6(18)	44(3)	15.1(18)	-2.4(19)	6.6(15)	-1.8(18)
N1	16(3)	23(4)	16(2)	0(3)	5(2)	-3(3)
C1	16(3)	20(4)	15(3)	1(3)	8(2)	-3(3)
B1	15(4)	22(4)	13(3)	0(3)	10(3)	2(3)
F2	30(2)	23(2)	20(2)	-0.6(18)	0.3(17)	2.4(18)
N2	13(3)	32(4)	21(3)	-8(3)	12(2)	-1(3)
C2	16(3)	26(4)	20(3)	-5(3)	6(2)	-2(3)
F3	13.2(18)	34(3)	19.2(19)	1.0(19)	8.5(15)	-6.2(17)
N3	9(3)	36(4)	16(2)	-7(3)	9(2)	0(3)
C3	14(3)	16(4)	24(3)	-2(3)	11(2)	-6(3)
C4	14(3)	17(4)	20(3)	0(3)	6(2)	-5(3)
C5	17(3)	38(4)	12(3)	6(3)	8(3)	1(3)
C6	16(3)	31(4)	15(3)	8(3)	3(3)	-2(3)
C7	17(4)	31(4)	37(4)	8(3)	6(3)	1(3)
C8	41(4)	35(5)	36(4)	6(4)	8(3)	-4(3)
C9	46(4)	71(6)	35(5)	6(4)	-1(4)	-32(4)
C10	27(4)	93(7)	37(5)	2(5)	10(4)	-29(4)
C11	26(4)	80(6)	23(4)	0(4)	9(3)	-15(4)
C12	12(3)	57(5)	17(3)	-4(3)	7(3)	1(3)
C13	21(3)	66(6)	26(4)	2(4)	10(3)	13(4)
C14	21(4)	118(8)	34(4)	2(5)	6(3)	24(5)

Table 3 Anisotropic Displacement Parameters (Å^2×10^3) for ojH388k. The Anisotropic displacement factor exponent takes the form: $-2\pi^2[h^2a^{*2}U_{11}+2hka^*b^*U_{12}+\ldots]$.

Atom	U_{11}	U_{22}	U_{33}	U_{23}	U_{13}	U_{12}
C15	20(4)	113(8)	33(4)	-9(5)	14(3)	6(4)
C16	19(4)	91(7)	28(4)	-9(4)	16(3)	-5(4)
C17	15(3)	59(6)	23(4)	1(4)	13(3)	-1(4)

Table 4 Bond Lengths for ojH388k.

Atom	Atom	Length/Å	Atom	Atom	Length/Å
K1	K1^{1}	4.337(3)	B1	C4	1.607(9)
K1	K1^{2}	4.6144(10)	N2	C2	1.346(7)
K1	K1^{3}	4.6145(10)	C2	C3	1.390(8)
K1	F1^{2}	2.785(4)	C2	C12	1.483(8)
K1	N1^{1}	3.093(5)	N3	C5	1.464(7)
K1	N1	2.872(5)	C3	C4	1.387(7)
K1	B1^{2}	3.412(8)	C5	C6	1.538(9)
K1	B1^{1}	3.528(7)	C6	C7	1.361(9)
K1	F2^{4}	2.700(4)	C6	C11	1.384(8)
K1	F2^{1}	2.748(4)	C7	C8	1.408(9)
K1	F3^{2}	2.824(4)	C8	C9	1.383(9)
K1	F3	2.699(3)	C9	C10	1.372(11)
F1	B1	1.413(7)	C10	C11	1.394(11)
N1	C1	1.372(7)	C12	C13	1.389(9)
N1	C4	1.359(7)	C12	C17	1.389(9)
C1	N2	1.343(7)	C13	C14	1.388(8)
C1	N3	1.363(7)	C14	C15	1.381(10)
B1	F2	1.423(9)	C15	C16	1.383(10)
B1	F3	1.434(8)	C16	C17	1.390(8)

^{1}1-X,1-Y,1-Z; ^{2}+X,1/2-Y,1/2+Z; ^{3}+X,1/2-Y,-1/2+Z; ^{4}1-X,-1/2+Y,1/2-Z

Table 5 Bond Angles for ojH388k.

Atom	Atom	Atom	Angle/˚	Atom	Atom	Atom	Angle/˚
K1^{1}	K1	K1^{2}	94.63(5)	F3^{3}	K1	B1^{1}	86.16(15)
K1^{1}	K1	K1^{3}	103.49(5)	F3	K1	F2^{1}	133.34(12)
K1^{3}	K1	K1^{2}	161.62(7)	F3	K1	F2^{4}	66.60(11)
F1^{3}	K1	K1^{2}	90.91(8)	F3	K1	F3^{3}	161.09(10)
F1^{3}	K1	K1^{1}	133.43(9)	B1	F1	K1^{2}	103.8(4)
F1^{3}	K1	K1^{3}	74.50(7)	K1	N1	K1^{1}	93.22(13)
F1^{3}	K1	N1	89.35(13)	C1	N1	K1^{1}	119.5(4)

Table 5 Bond Angles for ojH388k.

Atom	Atom	Atom	Angle/˚	Atom	Atom	Atom	Angle/˚
F1^3	K1	N1^1	167.08(13)	C1	N1	K1	114.9(4)
F1^3	K1	B1^1	123.17(15)	C4	N1	K1^1	99.5(4)
F1^3	K1	B1^3	23.72(13)	C4	N1	K1	110.7(4)
F1^3	K1	F3^3	47.75(9)	C4	N1	C1	116.1(5)
N1	K1	K1^2	85.17(10)	N2	C1	N1	126.6(5)
N1^1	K1	K1^3	94.65(9)	N2	C1	N3	117.2(5)
N1^1	K1	K1^1	41.39(9)	N3	C1	N1	116.1(5)
N1	K1	K1^3	105.37(10)	K1^2	B1	K1^1	143.1(2)
N1	K1	K1^1	45.39(10)	F1	B1	K1^1	154.0(5)
N1^1	K1	K1^2	101.04(10)	F1	B1	K1^2	52.4(3)
N1	K1	N1^1	86.78(13)	F1	B1	F2	108.3(5)
N1^1	K1	B1^1	44.12(15)	F1	B1	F3	105.8(5)
N1	K1	B1^3	101.31(16)	F1	B1	C4	112.4(5)
N1^1	K1	B1^3	146.37(15)	F2	B1	K1^1	46.4(3)
N1	K1	B1^1	83.86(15)	F2	B1	K1^2	127.9(4)
B1^3	K1	K1^2	112.07(13)	F2	B1	F3	106.5(5)
B1^1	K1	K1^3	53.77(13)	F2	B1	C4	111.2(6)
B1^1	K1	K1^2	143.90(13)	F3	B1	K1^1	90.3(3)
B1^3	K1	K1^3	51.72(12)	F3	B1	K1^2	54.2(3)
B1^3	K1	K1^1	136.35(14)	F3	B1	C4	112.2(5)
B1^1	K1	K1^1	54.36(12)	C4	B1	K1^1	78.7(3)
B1^3	K1	B1^1	103.78(7)	C4	B1	K1^2	120.9(4)
F2^4	K1	K1^2	32.43(8)	K1^5	F2	K1^1	115.78(13)
F2^4	K1	K1^3	129.44(10)	B1	F2	K1^1	111.5(3)
F2^1	K1	K1^1	73.74(9)	B1	F2	K1^5	132.4(3)
F2^4	K1	K1^1	127.04(9)	C1	N2	C2	116.5(5)
F2^1	K1	K1^2	165.81(10)	N2	C2	C3	120.7(5)
F2^1	K1	K1^3	31.79(8)	N2	C2	C12	116.2(6)
F2^1	K1	F1^3	102.97(11)	C3	C2	C12	123.1(5)
F2^4	K1	F1^3	69.84(10)	K1	F3	K1^2	113.30(13)
F2^4	K1	N1	108.79(13)	B1	F3	K1	121.8(3)
F2^1	K1	N1^1	64.89(12)	B1	F3	K1^2	101.5(3)
F2^1	K1	N1	91.96(12)	C1	N3	C5	121.8(5)
F2^4	K1	N1^1	123.06(13)	C4	C3	C2	120.1(5)
F2^1	K1	B1^1	22.03(15)	N1	C4	B1	116.0(5)
F2^4	K1	B1^3	85.47(15)	N1	C4	C3	119.9(6)
F2^4	K1	B1^1	162.85(15)	C3	C4	B1	124.0(5)
F2^1	K1	B1^3	82.12(14)	N3	C5	C6	114.0(5)
F2^4	K1	F2^1	157.58(11)	C7	C6	C5	120.1(6)
F2^4	K1	F3^3	97.86(11)	C7	C6	C11	119.7(7)
F2^1	K1	F3^3	64.26(11)	C11	C6	C5	120.1(6)

Table 5 Bond Angles for ojH388k.

Atom	Atom	Atom	Angle/°	Atom	Atom	Atom	Angle/°
F3^{3}	K1	K1^{2}	129.25(9)	C6	C7	C8	121.1(6)
F3^{3}	K1	K1^{1}	133.96(9)	C9	C8	C7	118.9(7)
F3^{3}	K1	K1^{3}	32.50(7)	C10	C9	C8	119.7(8)
F3	K1	K1^{1}	60.44(8)	C9	C10	C11	121.0(7)
F3	K1	K1^{3}	163.80(10)	C6	C11	C10	119.5(7)
F3	K1	K1^{2}	34.20(8)	C13	C12	C2	120.2(6)
F3	K1	F1^{3}	114.24(10)	C13	C12	C17	119.2(6)
F3^{3}	K1	N1	116.39(13)	C17	C12	C2	120.4(6)
F3	K1	N1^{1}	74.68(12)	C14	C13	C12	120.4(6)
F3	K1	N1	62.58(12)	C15	C14	C13	120.4(8)
F3^{3}	K1	N1^{1}	124.11(11)	C14	C15	C16	119.4(7)
F3^{3}	K1	B1^{3}	24.32(12)	C15	C16	C17	120.7(7)
F3	K1	B1^{1}	111.90(15)	C12	C17	C16	119.9(7)
F3	K1	B1^{3}	137.96(14)				

^{1}1-X,1-Y,1-Z; ^{2}+X,1/2-Y,-1/2+Z; ^{3}+X,1/2-Y,1/2+Z; ^{4}1-X,-1/2+Y,1/2-Z; ^{5}1-X,1/2+Y,1/2-Z

Table 6 Torsion Angles for ojH388k.

A	B	C	D	Angle/°	A	B	C	D	Angle/°
K1^{1}	F1	B1	K1^{2}	-136.4(9)	N2	C1	N3	C5	3.7(9)
K1^{1}	F1	B1	F2	-124.2(4)	N2	C2	C3	C4	2.1(10)
K1^{1}	F1	B1	F3	-10.3(5)	N2	C2	C12	C13	27.9(10)
K1^{1}	F1	B1	C4	112.5(5)	N2	C2	C12	C17	-148.2(7)
K1	N1	C1	N2	132.0(5)	C2	C3	C4	N1	0.2(9)
K1^{2}	N1	C1	N2	-118.5(6)	C2	C3	C4	B1	179.0(6)
K1^{2}	N1	C1	N3	63.1(7)	C2	C12	C13	C14	-178.7(7)
K1	N1	C1	N3	-46.4(7)	C2	C12	C17	C16	177.3(6)
K1	N1	C4	B1	46.3(6)	F3	B1	F2	K1^{3}	-112.0(4)
K1^{2}	N1	C4	B1	-50.8(6)	F3	B1	F2	K1^{2}	74.0(5)
K1^{2}	N1	C4	C3	128.1(5)	F3	B1	C4	N1	-42.5(8)
K1	N1	C4	C3	-134.8(5)	F3	B1	C4	C3	138.7(6)
K1^{1}	B1	F2	K1^{2}	131.2(3)	N3	C1	N2	C2	179.9(6)
K1^{2}	B1	F2	K1^{3}	174.0(5)	N3	C5	C6	C7	74.9(7)
K1^{1}	B1	F2	K1^{3}	-54.8(6)	N3	C5	C6	C11	-108.5(6)
K1^{1}	B1	F3	K1	126.9(4)	C3	C2	C12	C13	-151.2(7)
K1^{2}	B1	F3	K1^{1}	169.27(12)	C3	C2	C12	C17	32.7(10)
K1^{2}	B1	F3	K1	-63.8(3)	C4	N1	C1	N2	0.7(10)
K1^{2}	B1	C4	N1	43.1(5)	C4	N1	C1	N3	-177.7(6)
K1^{1}	B1	C4	N1	-103.0(6)	C4	B1	F2	K1^{2}	-48.6(5)

Table 6 Torsion Angles for ojH388k.

A	B	C	D	Angle/˚	A	B	C	D	Angle/˚
K1[1]	B1	C4	C3	78.2(7)	C4	B1	F2	K1[3]	125.4(4)
K1[2]	B1	C4	C3	-135.7(6)	C4	B1	F3	K1	14.1(7)
F1	B1	F2	K1[2]	-172.6(3)	C4	B1	F3	K1[1]	-112.9(5)
F1	B1	F2	K1[3]	1.4(8)	C5	C6	C7	C8	176.7(6)
F1	B1	F3	K1[1]	10.0(5)	C5	C6	C11	C10	-176.4(6)
F1	B1	F3	K1	137.0(4)	C6	C7	C8	C9	-0.8(10)
F1	B1	C4	N1	-161.6(6)	C7	C6	C11	C10	0.2(10)
F1	B1	C4	C3	19.6(9)	C7	C8	C9	C10	1.3(11)
N1	C1	N2	C2	1.5(10)	C8	C9	C10	C11	-1.0(12)
N1	C1	N3	C5	-177.7(6)	C9	C10	C11	C6	0.3(11)
C1	N1	C4	B1	179.6(5)	C11	C6	C7	C8	0.1(10)
C1	N1	C4	C3	-1.6(9)	C12	C2	C3	C4	-178.9(6)
C1	N2	C2	C3	-2.9(10)	C12	C13	C14	C15	2.6(13)
C1	N2	C2	C12	178.0(6)	C13	C12	C17	C16	1.2(11)
C1	N3	C5	C6	82.9(7)	C13	C14	C15	C16	-1.3(13)
F2	B1	F3	K1[1]	125.1(4)	C14	C15	C16	C17	0.0(13)
F2	B1	F3	K1	-107.9(5)	C15	C16	C17	C12	0.1(12)
F2	B1	C4	N1	76.8(7)	C17	C12	C13	C14	-2.5(11)
F2	B1	C4	C3	-102.0(7)					

[1]+X,1/2-Y,-1/2+Z; [2]1-X,1-Y,1-Z; [3]1-X,1/2+Y,1/2-Z

Table 7 Hydrogen Atom Coordinates (Å×10^4) and Isotropic Displacement Parameters (Å^2×10^3) for ojH388k.

Atom	*x*	*y*	*z*	U(eq)
H3	6022.19	4295.73	7441.48	24
H3A	7077.71	4342.09	2002.16	21
H5A	7425.49	3422.76	8128.53	26
H5B	6824.36	3656.78	9294.03	26
H7	6590.36	6103.73	9974.37	34
H8	7186.3	8068.28	10852.51	44
H9	8412.32	8433.43	10358.26	61
H10	9005.74	6911.99	8938.58	62
H11	8406.14	4973.7	8055.25	51
H13	8584.13	2844.82	5618.07	45
H14	9819.85	2598.57	5173.73	69
H15	10297.86	3708.01	3202.93	66
H16	9520.79	4982.55	1611.93	54
H17	8272.35	5166.4	1989.25	39

X-ray crystallographic analysis for compound 10a

Deposition number CCDC 2062210

ORTEP of 10a, thermal ellipsoids are shown at 50% probability

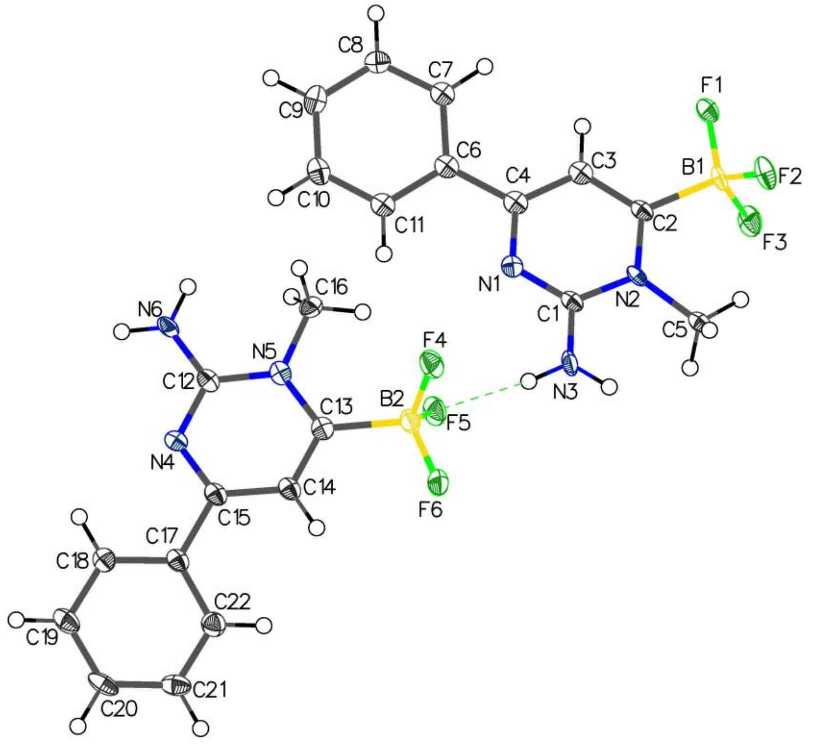

Crystal structure determination of compound 10a

Crystals of 10a were grown from a saturated acetone solution, allowing slow evaporation.

Crystal Data for $C_{11}H_{11}BF_3N_3$ (M =253.04 g/mol): monoclinic, space group $P2_1/n$ (no. 14), a = 17.8400(7) Å, b = 6.8724(3) Å, c = 19.3259(8) Å, β = 102.492(2)°, V = 2313.33(17) Å^3, Z = 8, T = 100.03 K, μ(CuKα) = 1.055 mm^{-1}, $Dcalc$ = 1.453 g/cm^3, 12247 reflections measured (6.116° ≤ 2Θ ≤ 133.71°), 3735 unique (R_{int} = 0.0706, R_{sigma} = 0.0683) which were used in all calculations. The final R_1 was 0.1587 (I > 2σ(I)) and wR_2 was 0.3409 (all data).

Table 1 Crystal data and structure refinement for OJH391v_0m.

Identification code	OJH391v_0m
Empirical formula	$C_{11}H_{11}BF_3N_3$
Formula weight	253.04
Temperature/K	100.03
Crystal system	monoclinic
Space group	$P2_1/n$

a/Å	17.8400(7)
b/Å	6.8724(3)
c/Å	19.3259(8)
α/°	90
β/°	102.492(2)
γ/°	90
Volume/Å^3	2313.33(17)
Z	8
ρ_{calc}g/cm^3	1.453
μ/mm^{-1}	1.055
F(000)	1040.0
Crystal size/mm^3	0.2 × 0.034 × 0.025
Radiation	CuKα (λ = 1.54178)
2Θ range for data collection/°	6.116 to 133.71
Index ranges	-21 ≤ h ≤ 21, -7 ≤ k ≤ 8, -22 ≤ l ≤ 22
Reflections collected	12247
Independent reflections	3735 [R_{int} = 0.0706, R_{sigma} = 0.0683]
Data/restraints/parameters	3735/282/279
Goodness-of-fit on F^2	1.419
Final R indexes [I>=2σ (I)]	R_1 = 0.1587, wR_2 = 0.3370
Final R indexes [all data]	R_1 = 0.1701, wR_2 = 0.3409
Largest diff. peak/hole / e Å^{-3}	0.58/-0.53

Table 2 Fractional Atomic Coordinates (×10^4) and Equivalent Isotropic Displacement Parameters (Å^2×10^3) for OJH391v_0m. U_{eq} is defined as 1/3 of of the trace of the orthogonalised U_{IJ} tensor.

Atom	*x*	*y*	*z*	U(eq)
F1	783(3)	4825(10)	4127(3)	31.3(15)
N1	3398(4)	3611(11)	3600(4)	15.2(15)
C1	3503(5)	3610(14)	4301(5)	16.7(11)
B1	1457(6)	4661(15)	4654(6)	15.0(18)
F2	1385(3)	3095(9)	5100(3)	27.9(14)
N2	2910(4)	3998(11)	4639(4)	13.7(15)
C2	2178(5)	4252(14)	4258(5)	16.7(11)
F3	1570(3)	6370(9)	5045(3)	27.0(14)
N3	4191(4)	3323(13)	4686(4)	21.9(18)
C3	2063(5)	4146(13)	3537(5)	16.1(8)
C4	2688(5)	3875(13)	3215(5)	16.1(8)
C5	3096(5)	4202(14)	5416(5)	16.7(11)
C6	2610(5)	3877(13)	2434(5)	16.1(8)
C7	1890(5)	3723(13)	1961(5)	16.1(8)

Table 2 Fractional Atomic Coordinates (×10⁴) and Equivalent Isotropic Displacement Parameters (Å²×10³) for OJH391v_0m. U_{eq} is defined as 1/3 of of the trace of the orthogonalised U_{IJ} tensor.

Atom	*x*	*y*	*z*	U(eq)
C8	1836(5)	3778(14)	1237(5)	17.9(19)
C9	2480(6)	3963(14)	958(5)	20.0(19)
C10	3193(6)	4094(14)	1417(5)	19.8(19)
C11	3267(5)	4061(13)	2146(5)	16.1(8)
B2	5799(6)	663(17)	3943(6)	19(2)
F4	5301(3)	-934(9)	3877(3)	27.7(14)
N4	6988(4)	972(11)	2157(4)	14.5(15)
F5	5386(3)	2399(9)	3956(3)	25.9(14)
N5	5838(4)	889(11)	2584(4)	15.4(15)
F6	6340(3)	487(11)	4562(3)	33.2(16)
N6	5824(4)	1077(12)	1378(4)	19.1(17)
C12	6220(5)	968(14)	2043(5)	16.3(17)
C13	6248(5)	744(13)	3276(5)	16.3(17)
C14	7024(5)	723(13)	3387(5)	17.2(11)
C15	7385(5)	873(14)	2814(5)	17.2(11)
C16	5001(5)	1022(15)	2411(5)	20(2)
C17	8231(5)	935(14)	2913(5)	17.2(11)
C18	8564(5)	786(14)	2328(5)	18.7(19)
C19	9352(5)	891(16)	2395(6)	25(2)
C20	9819(5)	1133(14)	3065(6)	22(2)
C21	9502(6)	1247(15)	3651(6)	24(2)
C22	8719(6)	1124(15)	3585(5)	23(2)

Table 3 Anisotropic Displacement Parameters (Å²×10³) for OJH391v_0m. The Anisotropic displacement factor exponent takes the form: -2π²[h²a*²U₁₁+2hka*b*U₁₂+…].

Atom	U_{11}	U_{22}	U_{33}	U_{23}	U_{13}	U_{12}
F1	13(3)	56(4)	26(3)	-5(3)	7(2)	3(3)
N1	20(3)	8(4)	19(3)	-1(3)	6(3)	-3(3)
C1	12(2)	16(3)	23(2)	-1(2)	5.5(18)	0(2)
B1	15(4)	13(4)	21(5)	0(3)	13(3)	0(4)
F2	30(3)	27(3)	33(3)	7(3)	20(3)	-1(3)
N2	11(3)	10(4)	21(3)	3(3)	7(3)	-2(3)
C2	12(2)	16(3)	23(2)	-1(2)	5.5(18)	0(2)
F3	28(3)	24(3)	34(3)	-6(2)	16(3)	4(3)
N3	16(3)	36(5)	17(4)	3(4)	10(3)	3(4)
C3	19.6(19)	6.4(17)	22.5(18)	0.9(16)	4.9(15)	0.2(16)
C4	19.6(19)	6.4(17)	22.5(18)	0.9(16)	4.9(15)	0.2(16)
C5	12(2)	16(3)	23(2)	-1(2)	5.5(18)	0(2)
C6	19.6(19)	6.4(17)	22.5(18)	0.9(16)	4.9(15)	0.2(16)

Table 3 Anisotropic Displacement Parameters (Å^2×10^3) for OJH391v_0m. The Anisotropic displacement factor exponent takes the form: -2π^2[h^{2}a*^{2}U$_{11}$+2hka*b*U$_{12}$+…].

Atom	U_{11}	U_{22}	U_{33}	U_{23}	U_{13}	U_{12}
C7	19.6(19)	6.4(17)	22.5(18)	0.9(16)	4.9(15)	0.2(16)
C8	17(4)	14(5)	22(4)	0(4)	1(3)	4(4)
C9	32(4)	10(4)	19(4)	5(4)	8(3)	5(4)
C10	25(4)	11(4)	26(4)	3(4)	11(3)	0(4)
C11	19.6(19)	6.4(17)	22.5(18)	0.9(16)	4.9(15)	0.2(16)
B2	19(5)	20(5)	20(4)	-1(4)	9(3)	-3(4)
F4	28(3)	29(3)	28(3)	2(3)	12(3)	-7(3)
N4	15(3)	8(4)	21(3)	0(3)	4(3)	-1(3)
F5	24(3)	27(3)	30(3)	-5(3)	13(3)	4(2)
N5	18(3)	8(4)	21(3)	0(3)	8(3)	2(3)
F6	21(3)	61(5)	19(3)	-4(3)	8(2)	1(3)
N6	9(4)	25(4)	24(3)	-3(3)	6(3)	2(3)
C12	16(4)	12(4)	21(4)	3(4)	6(3)	0(4)
C13	21(4)	7(4)	22(4)	-4(4)	8(3)	-1(3)
C14	18(2)	12(2)	23(2)	0(2)	7.2(18)	-3(2)
C15	18(2)	12(2)	23(2)	0(2)	7.2(18)	-3(2)
C16	19(4)	23(5)	19(5)	-7(4)	3(4)	-2(4)
C17	18(2)	12(2)	23(2)	0(2)	7.2(18)	-3(2)
C18	20(4)	15(5)	22(4)	-2(4)	7(3)	1(4)
C19	16(4)	26(6)	34(5)	-2(5)	9(4)	3(4)
C20	11(4)	15(5)	41(5)	4(4)	6(3)	3(4)
C21	15(4)	21(5)	32(5)	-5(4)	-1(4)	2(4)
C22	22(4)	23(5)	25(4)	-3(4)	7(3)	-1(4)

Table 4 Bond Lengths for OJH391v_0m.

Atom	Atom	Length/Å	Atom	Atom	Length/Å
F1	B1	1.402(13)	B2	F4	1.401(12)
N1	C1	1.327(12)	B2	F5	1.405(13)
N1	C4	1.335(12)	B2	F6	1.370(13)
C1	N2	1.384(11)	B2	C13	1.658(13)
C1	N3	1.304(12)	N4	C12	1.339(12)
B1	F2	1.403(12)	N4	C15	1.315(12)
B1	C2	1.657(13)	N5	C12	1.368(11)
B1	F3	1.387(12)	N5	C13	1.383(12)
N2	C2	1.364(12)	N5	C16	1.460(12)
N2	C5	1.474(11)	N6	C12	1.328(12)
C2	C3	1.365(13)	C13	C14	1.355(13)
C3	C4	1.402(13)	C14	C15	1.399(13)
C4	C6	1.486(13)	C15	C17	1.481(13)

Table 4 Bond Lengths for OJH391v_0m.

Atom	Atom	Length/Å	Atom	Atom	Length/Å
C6	C7	1.410(13)	C17	C18	1.390(13)
C6	C11	1.408(12)	C17	C22	1.405(14)
C7	C8	1.383(13)	C18	C19	1.385(13)
C8	C9	1.377(13)	C19	C20	1.389(15)
C9	C10	1.386(14)	C20	C21	1.373(14)
C10	C11	1.386(13)	C21	C22	1.377(14)

Table 5 Bond Angles for OJH391v_0m.

Atom	Atom	Atom	Angle/˚	Atom	Atom	Atom	Angle/˚
C1	N1	C4	118.5(8)	F4	B2	F5	110.0(8)
N1	C1	N2	121.8(8)	F4	B2	C13	111.0(8)
N3	C1	N1	119.3(8)	F5	B2	C13	109.2(8)
N3	C1	N2	118.8(8)	F6	B2	F4	108.9(9)
F1	B1	F2	109.3(8)	F6	B2	F5	109.4(8)
F1	B1	C2	107.8(7)	F6	B2	C13	108.3(8)
F2	B1	C2	109.7(8)	C15	N4	C12	118.4(8)
F3	B1	F1	109.2(8)	C12	N5	C13	119.7(8)
F3	B1	F2	109.7(8)	C12	N5	C16	118.5(8)
F3	B1	C2	111.1(8)	C13	N5	C16	121.8(7)
C1	N2	C5	118.1(7)	N4	C12	N5	122.4(8)
C2	N2	C1	120.7(8)	N6	C12	N4	118.1(8)
C2	N2	C5	121.1(7)	N6	C12	N5	119.4(8)
N2	C2	B1	121.3(8)	N5	C13	B2	120.7(8)
N2	C2	C3	117.3(8)	C14	C13	B2	121.8(8)
C3	C2	B1	121.4(8)	C14	C13	N5	117.5(8)
C2	C3	C4	120.1(9)	C13	C14	C15	120.2(9)
N1	C4	C3	121.3(8)	N4	C15	C14	121.7(8)
N1	C4	C6	115.8(8)	N4	C15	C17	116.3(8)
C3	C4	C6	122.8(8)	C14	C15	C17	122.0(8)
C7	C6	C4	122.0(8)	C18	C17	C15	119.7(8)
C11	C6	C4	119.9(8)	C18	C17	C22	118.0(9)
C11	C6	C7	118.0(8)	C22	C17	C15	122.2(8)
C8	C7	C6	120.6(9)	C19	C18	C17	121.5(9)
C9	C8	C7	121.1(9)	C18	C19	C20	119.1(9)
C8	C9	C10	118.9(9)	C21	C20	C19	120.3(9)
C11	C10	C9	121.5(9)	C20	C21	C22	120.6(9)
C10	C11	C6	119.9(9)	C21	C22	C17	120.4(9)

Table 6 Torsion Angles for OJH391v_0m.

A	B	C	D	Angle/°	A	B	C	D	Angle/°
F1	B1	C2	N2	-179.0(8)	B2	C13	C14	C15	-177.4(9)
F1	B1	C2	C3	0.7(12)	F4	B2	C13	N5	59.5(12)
N1	C1	N2	C2	5.0(14)	F4	B2	C13	C14	-122.3(10)
N1	C1	N2	C5	-172.7(8)	N4	C15	C17	C18	-10.0(13)
N1	C4	C6	C7	166.2(8)	N4	C15	C17	C22	170.6(9)
N1	C4	C6	C11	-14.6(13)	F5	B2	C13	N5	-62.0(11)
C1	N1	C4	C3	0.0(13)	F5	B2	C13	C14	116.3(10)
C1	N1	C4	C6	179.5(8)	N5	C13	C14	C15	0.9(13)
C1	N2	C2	B1	178.4(8)	F6	B2	C13	N5	179.0(8)
C1	N2	C2	C3	-1.3(13)	F6	B2	C13	C14	-2.8(13)
B1	C2	C3	C4	177.5(8)	C12	N4	C15	C14	1.4(14)
F2	B1	C2	N2	-60.1(12)	C12	N4	C15	C17	-178.4(8)
F2	B1	C2	C3	119.6(10)	C12	N5	C13	B2	179.5(8)
N2	C2	C3	C4	-2.9(14)	C12	N5	C13	C14	1.2(13)
C2	C3	C4	N1	3.6(14)	C13	N5	C12	N4	-2.2(13)
C2	C3	C4	C6	-175.8(9)	C13	N5	C12	N6	179.0(9)
F3	B1	C2	N2	61.4(12)	C13	C14	C15	N4	-2.3(15)
F3	B1	C2	C3	-118.9(10)	C13	C14	C15	C17	177.5(9)
N3	C1	N2	C2	-178.1(9)	C14	C15	C17	C18	170.2(9)
N3	C1	N2	C5	4.2(13)	C14	C15	C17	C22	-9.2(15)
C3	C4	C6	C7	-14.3(14)	C15	N4	C12	N5	0.8(14)
C3	C4	C6	C11	165.0(9)	C15	N4	C12	N6	179.7(9)
C4	N1	C1	N2	-4.3(13)	C15	C17	C18	C19	178.2(9)
C4	N1	C1	N3	178.9(9)	C15	C17	C22	C21	-177.6(9)
C4	C6	C7	C8	178.4(8)	C16	N5	C12	N4	175.9(9)
C4	C6	C11	C10	-179.0(9)	C16	N5	C12	N6	-2.9(13)
C5	N2	C2	B1	-4.0(13)	C16	N5	C13	B2	1.5(13)
C5	N2	C2	C3	176.4(8)	C16	N5	C13	C14	-176.8(8)
C6	C7	C8	C9	0.7(14)	C17	C18	C19	C20	0.6(16)
C7	C6	C11	C10	0.3(14)	C18	C17	C22	C21	2.9(15)
C7	C8	C9	C10	0.0(14)	C18	C19	C20	C21	0.7(16)
C8	C9	C10	C11	-0.6(15)	C19	C20	C21	C22	-0.1(16)
C9	C10	C11	C6	0.5(14)	C20	C21	C22	C17	-1.8(16)
C11	C6	C7	C8	-0.8(14)	C22	C17	C18	C19	-2.3(15)

Table 7 Hydrogen Atom Coordinates (Å×10^4) and Isotropic Displacement Parameters (Å^2×10^3) for OJH391v_0m.

Atom	*x*	*y*	*z*	U(eq)
H3A	4578.98	3132.98	4479.7	26
H3B	4266.38	3319.69	5150.93	26
H3	1558.68	4255.6	3253.3	19

Table 7 Hydrogen Atom Coordinates (Å×10^4) and Isotropic Displacement Parameters (Å^2×10^3) for OJH391v_0m.

Atom	*x*	*y*	*z*	U(eq)
H5A	3282.07	2954.72	5632.71	25
H5B	2635.13	4594.65	5578.72	25
H5C	3495.71	5193.5	5553.59	25
H7	1438.5	3580.37	2141.99	19
H8	1345.28	3686.14	926.16	22
H9	2437.64	4000.07	459.86	24
H10	3639.56	4208.7	1227.71	24
H11	3760.74	4162.97	2450.26	19
H6A	6065.43	1149.91	1027.74	23
H6B	5318.42	1076.72	1288.56	23
H14	7325.26	606.44	3854.59	21
H16A	4843.67	2161.79	2109.77	31
H16B	4812.27	1150.73	2848.92	31
H16C	4785.55	-155.97	2158.59	31
H18	8243.4	608.61	1871.02	22
H19	9569.43	797.36	1989.38	29
H20	10359.54	1220.86	3116.87	27
H21	9826.58	1411.6	4106.63	28
H22	8508.62	1167.24	3995.75	27
